# Enzymatic synthesis of hypermodified DNA polymers for sequence-specific display of four different hydrophobic groups

**DOI:** 10.1093/nar/gkaa999

**Published:** 2020-11-05

**Authors:** Marek Ondruš, Veronika Sýkorová, Lucie Bednárová, Radek Pohl, Michal Hocek

**Affiliations:** Institute of Organic Chemistry and Biochemistry, Czech Academy of Sciences, Flemingovo nam. 2, CZ-16000 Prague 6, Czech Republic; Department of Organic Chemistry, Faculty of Science, Charles University in Prague, Hlavova 8, CZ-12843 Prague 2, Czech Republic; Institute of Organic Chemistry and Biochemistry, Czech Academy of Sciences, Flemingovo nam. 2, CZ-16000 Prague 6, Czech Republic; Institute of Organic Chemistry and Biochemistry, Czech Academy of Sciences, Flemingovo nam. 2, CZ-16000 Prague 6, Czech Republic; Institute of Organic Chemistry and Biochemistry, Czech Academy of Sciences, Flemingovo nam. 2, CZ-16000 Prague 6, Czech Republic; Institute of Organic Chemistry and Biochemistry, Czech Academy of Sciences, Flemingovo nam. 2, CZ-16000 Prague 6, Czech Republic; Department of Organic Chemistry, Faculty of Science, Charles University in Prague, Hlavova 8, CZ-12843 Prague 2, Czech Republic

## Abstract

A set of modified 2′-deoxyribonucleoside triphosphates (dNTPs) bearing a linear or branched alkane, indole or phenyl group linked through ethynyl or alkyl spacer were synthesized and used as substrates for polymerase synthesis of hypermodified DNA by primer extension (PEX). Using the alkyl-linked dNTPs, the polymerase synthesized up to 22-mer fully modified oligonucleotide (ON), whereas using the ethynyl-linked dNTPs, the enzyme was able to synthesize even long sequences of >100 modified nucleotides in a row. In PCR, the combinations of all four modified dNTPs showed only linear amplification. Asymmetric PCR or PEX with separation or digestion of the template strand can be used for synthesis of hypermodified single-stranded ONs, which are monodispersed polymers displaying four different substituents on DNA backbone in sequence-specific manner. The fully modified ONs hybridized with complementary strands and modified DNA duplexes were found to exist in B-type conformation (B- or C-DNA) according to CD spectral analysis. The modified DNA can be replicated with high fidelity to natural DNA through PCR and sequenced. Therefore, this approach has a promising potential in generation and selection of hypermodified aptamers and other functional polymers.

## INTRODUCTION

Targeting protein-protein interactions is one of the most important challenges of current medicinal chemistry and one of the promising approaches is the use of nucleic acids aptamers (1,2), which can bind proteins (or other targets) with high affinities and specificities. Aptamers are typically selected using *in vitro* systematic evolution of ligand by exponential enrichment (SELEX). Although there are many known aptamers including clinically used drugs based on non-modified RNA or DNA (3,4), the absence of hydrophobic functional groups in natural nucleic acids limits the potential for targeting hydrophobic proteins. Although there were several recent reviews summarizing chemically modified aptamers (5–7) and outlining the potential of the use of modified nucleotides, there were only a few real studies of selection of base-modified aptamers using one modified nucleotide (mostly modified uracil) (8–18). More recent works confirmed that the use of two modified nucleotides can be even more beneficial in the aptamer selection (19,20). Alternative approaches to introduce functionality to aptamers are the use of click-reaction (21–27), extended genetic alphabet (28–31) or ligation of short modified oligonucleotides (32–37). Obviously, if we can use all-four modified nucleotides each bearing a different functional group, we could dramatically increase the potential of modified aptamers to target many undruggable biomolecular targets and such hypermodified DNA would sequence-specifically display four different functional groups as a special case of sequence-defined functional polymers (38–42).

Polymerase synthesis of nucleobase-functionalized nucleic acids using base-modified nucleoside triphosphates (dNTPs) is now an established method competing with chemical synthesis (43,44). 5-Substituted pyrimidine or 7-substituted 7-deazapurine dNTPs are very good substrates (45–49) for DNA polymerases and can be used in primer extension (PEX) (43), PCR (43), nicking enzyme amplification reaction (NEAR) (50,51), terminal deoxynucleotidyl transferase 3′-end labelling (52) or rolling-cycle amplification (RCA) (53). In this way, number of hydrophobic modifications have been attached to DNA, e.g. diamondoids (54), oligoethyleneglycols (55), grafted polymers (56), steroids (57), carboranes (58), aromatic hydrocarbons (59) etc. However, in most cases, only one modified dNTP was used in presence of three natural dNTPs. Several studies used two or three functionalized dNTPs at the same time showing that even the PEX reaction becomes more difficult and PCR typically did not work (60–64). Only few papers published (65–68) in 2001–2005 had reported to achieve simultaneous incorporation of all four functionalized dNTPs in PEX and even PCR using specific set of dNTPs (two hydrophobic and two polar) and short 59- or 79-mer sequences and these works have never been replicated or followed up by other groups. A special case of an incorporation of four different (fluorescently) modified nucleotides is reversible terminator synthesis used in next generation sequencing (NGS) but major part of the modification is always removed after each step (69–71). A more recent study has reported PCR and even *in vivo* replication of DNA composed of all four modified bases, but the set of modified dNTPs was composed of halogenated pyrimidines and unsubstituted 7-deazapurines (72). Another recent work has achieved the synthesis of longer fully modified duplexes through extension of self-priming ‘oligoseeds’ (73). For possible applications in generation of hypermodified DNA polymers or in selection of hypermodified aptamers, there is a great need of a robust enzymatic method for synthesis of hypermodified DNA, its amplification and sequencing and we report here methodology for incorporation of four different nucleotides bearing four different hydrophobic groups and properties of the hypermodified hydrophobic oligonucleotide polymers.

## MATERIALS AND METHODS

Complete experimental part, procedures and characterization of all compounds is given in Supporting Information. Only most important typical procedures are given below:

### Ethynyl-modified nucleosides synthesis by cross-coupling reaction

1:2 mixture of AN/H_2_O (2 ml) was added through a septum to an argon-purged flask containing **dN^I^** (1 equiv.), TPPTS (11 mol.%), CuI (8 mol.%) and Pd(OAc)_2_ (7 mol.%) followed by addition of excess of terminal alkyne **1a**–**1d** (Supplementary Table S1) and TEA (6 equiv.) (Supplementary Scheme S1). The reaction mixture was stirred at room temperature overnight (48 h in case of **dG^I^**) and then evaporated under vacuum. The product was purified by FLC chromatography using DCM/MeOH (0–30%) as eluent followed by evaporation under vacuum to get solid product.

### Alkyl-modified nucleosides synthesis by catalytic hydrogenation

MeOH (5 ml) was added through a septum to an argon-purged flask containing **dN^ER^** (1 equiv.), 10% Pd/C (10 mol.%) followed by vacuuming and fulfilling with H_2_ atmosphere (balloon) (Supplementary Scheme S2). The reaction mixture was stirred at r.t. (reflux in case of **dU^EPh^**) for desired time (Supplementary Table S2) until complete consumption of the starting material and then evaporated under vacuum. The product was purified by FLC chromatography using DCM/MeOH (0–30%) as eluent followed by evaporation under vacuum to get solid product.

### Modified dN^R^TPs synthesis by phosphorylation

PO(OMe)_3_ (1 mL) was added through a septum to an argon-purged flask containing modified nucleosides **dN^ER^** or **dN^AR^** (1 equiv.) followed by dropwise addition of POCl_3_ (1.2 equiv.) at –10°C (ice bath + NaCl) and the reaction mixture was stirred for 2 h at –10°C. Content of ice-cooled mixture containing solution of (NHBu_3_)_2_H_2_P_2_O_7_ (5 equiv.) and Bu_3_N (4 equiv.) in dry DMF (1 mL) was added dropwise and the reaction mixture was stirred for another 1 h at –10°C. The reaction was quenched by addition of aqueous 2 M TEAB (triethylammonium bicarbonate) (5 ml). Solvents were evaporated under vacuum and co-distilled with water three times. The product was purified by HPLC on a C18 column with use of linear gradient from 0.1 M TEAB in H_2_O to 0.1 M TEAB in H_2_O/MeOH (1:1) as eluent. Conversion to sodium salt by ion exchange resin Dowex 50WX8 followed by freeze-drying from water gave solid product (Supplementary Table S3).

### Multiple incorporation of four modified dN^R^TPs by PEX

Reaction mixture (10 μl) contained prb4basII template (3 μM, 0.75 μl), 5′-(6-FAM)-labelled prim248short primer (3 μM, 0.5 μl), mix of four modified **dN^R^TPs** (2 mM, 1 μl), Vent (exo-) DNA polymerase (2 U) and reaction buffer (10×, 1 μl) as supplied by the manufacturer. Positive control contained 0.5 U of Vent (exo-) DNA polymerase and mix of natural dNTPs (1 mM, 1 μl). The reaction mixture was incubated for 60 min at 60°C, stopped by addition of PAGE stop solution (10 μl) and denaturated for 3 min at 95°C. Samples were analysed by 12.5% PAGE and visualised using fluorescence imaging.

### Multiple incorporation of four modified dN^E^TPs by aPCR

Reaction mixture (20 μl) contained one of MO77/FVL-A/MO120 / MO150 template (5 μM, 1 μl), 5′-(6-FAM)-labelled LT25TH primer (10 μM, 4 μl), mix of ethynyl-modified **dN^ER^TPs** (2 μl), one of DNA polymerase (5–10 U) and reaction buffer (10×, 2 μl) as supplied by the manufacturer (Supplementary Table S10 and S11). All reaction mixtures were under cycling protocol: 95°C for 1 min, followed by 50 cycles at 95°C for 1 min, 50°C for 1 min, and 70°C for 2 min, followed by a final elongation step at 70°C for 5 min. Samples were analysed on 3% agarose gel as well as by 12.5% PAGE and visualised using fluorescence imaging.

### Re-PCR of modified ssONs to natural DNA

Reaction mixture (20 μl) contained modified **97ON_N^ER^**as template (7.5 μM, 1 μl), 5′-(6-FAM)-labeled L20 and Flank primers (10 μM, 4 μl, each), mix of natural dNTPs (2 μl, 4 mM), Vent (exo-) DNA polymerase (6 U) and reaction buffer (10×, 2 μl) as supplied by the manufacturer. Reaction mixtures were under cycling protocol: 95°C for 1 min, followed by 30 cycles at 95°C for 1 min, 55°C for 1 min, and 70°C for 2 min, followed by a final elongation step at 70°C for 5 min. Samples were analysed on 3% agarose gel as well as by 12.5% PAGE and visualised using fluorescence imaging.

## RESULTS AND DISCUSSION

**Scheme 1.**
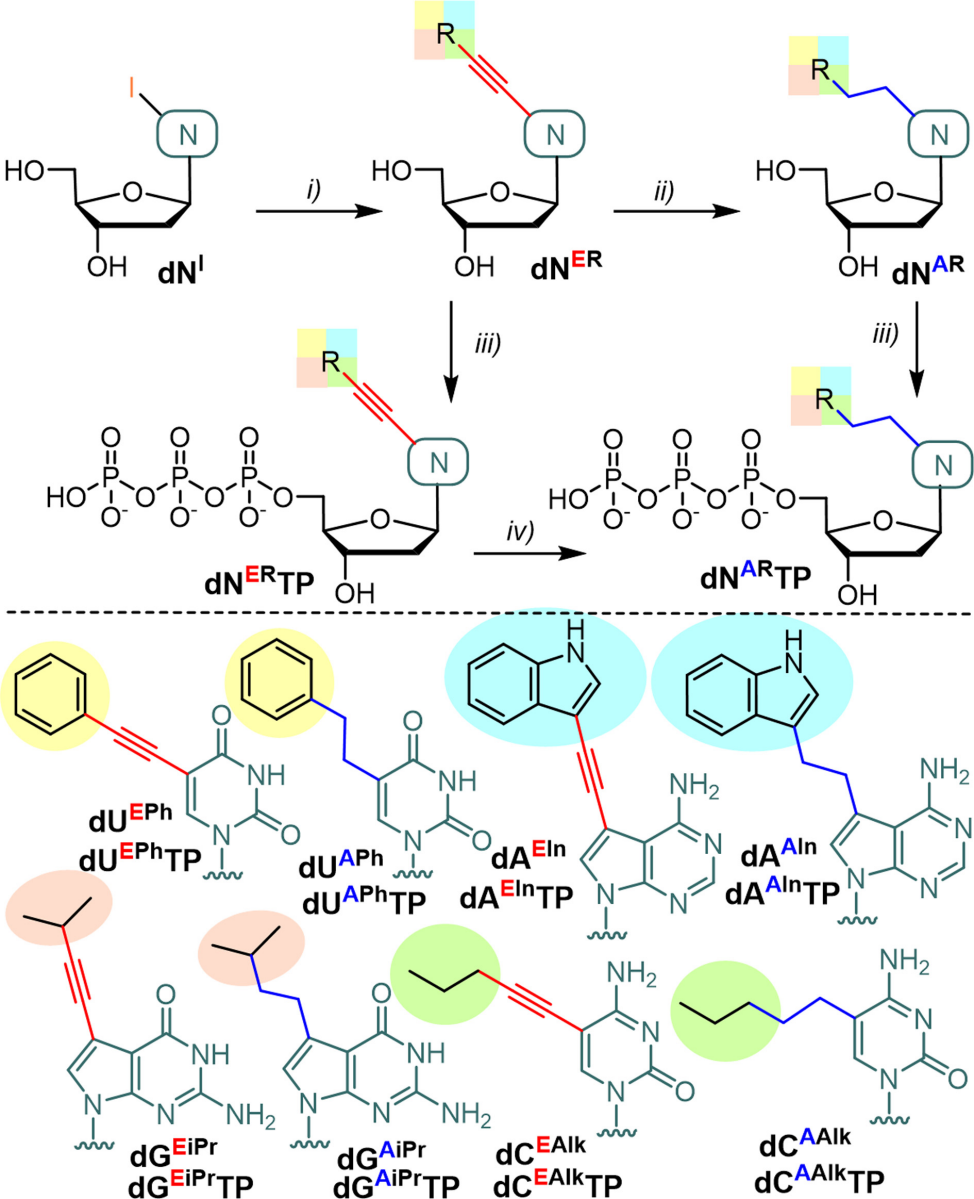
Design and synthesis of modified **dN^R^TP**s. Reagents and conditions: (i) R-C≡CH (excess), Pd(OAc)_2_ (7 mol.%), TPPTS (11 mol. %), CuI (8 mol.%), TEA (6 equiv.), AN/H_2_O 1:2, r.t., 16–48 h, under Ar, 69–98%; (ii) H_2_ (1 atm.), 10% Pd/C (10 mol. %), MeOH, r.t., 75°C, overnight, 93–98%; (iii) 1. POCl_3_ (1.2 equiv.), PO(OMe)_3_, -10°C, 2 h, under Ar; 2. (NHBu_3_)_2_H_2_P_2_O_7_ (5 equiv.), Bu_3_N (4 equiv.), DMF, -10°C, 1 h, under Ar; 3. 2 M TEAB; 18–28%; (iv) H_2_ (1 atm.), 10% Pd/C (10 mol. %), H_2_O, r.t., 4 h, 25%.

The design of the four different hydrophobic modifications of the dNTP building blocks was inspired by hydrophobic amino acid side-chains to enhance the chance for interactions of the hypermodified polymers with proteins. Thus, we have chosen phenyl and indol-3-yl groups as aromatic substituents and propyl and isopropyl groups as aliphatic substituents and envisaged to link them to position 5 of pyrimidines or to position 7 of 7-deazapurines either through rigid acetylene linker or through flexible ethane tether (Scheme 1). The synthesis of the ethynyl-linked nucleosides was performed through the aqueous Sonogashira cross-coupling reactions of iodinated 2′-deoxyribonucleosides (**dN^I^**s) with the corresponding terminal alkynes in presence of Pd(OAc)_2_, triphenylphosphine-3,3′,3″-trisulfonate (TPPTS), CuI and Et_3_N in mixture of acetonitrile (AN) and water. The reactions gave the desired ethynyl-linked nucleosides **dN^ER^** in good to excellent yields (69–98%). Undesired Cu(I)-mediated cyclization of 5-alkylethynyl uracil derivatives to furopyrimidines (74–77) was observed during the coupling reactions performed at higher temperatures (>75°C), whereas no cyclization occurred when performing the reaction at r.t.. Transformation of ethynyl-linked nucleosides (**dN^ER^**, E-series) to alkyl-linked ones (**dN^AR^**, A-series) was accomplished by catalytic hydrogenation using Pd/C in MeOH in excellent 93–98% yields. The triphosphorylation of nucleosides **dN^ER^** or **dN^AR^** was performed using standard protocol (78) to give the desired triphosphates **dN^ER^TP** or **dN^AR^TP** in acceptable yields of 18–28% after HPLC purification and conversion to sodium salts by ion exchange chromatography. An alternative synthesis of alkyl-linked triphosphate **dC^AAlk^TP** was also performed through catalytic hydrogenation (79) of ethynyl-linked nucleotide **dC^EAlk^TP** but the yield was significantly decreased (25% yield) by partial hydrolysis of the triphosphate and partial reduction of the pyrimidine base. Therefore, it was more efficient to perform the catalytic hydrogenation on nucleosides followed by triphosphorylation. For detailed summary of the synthesis of the nucleosides and nucleotides, see Schemes S1–S3 and Tables S1–S3 in the Supporting Information.

**Figure 1.**
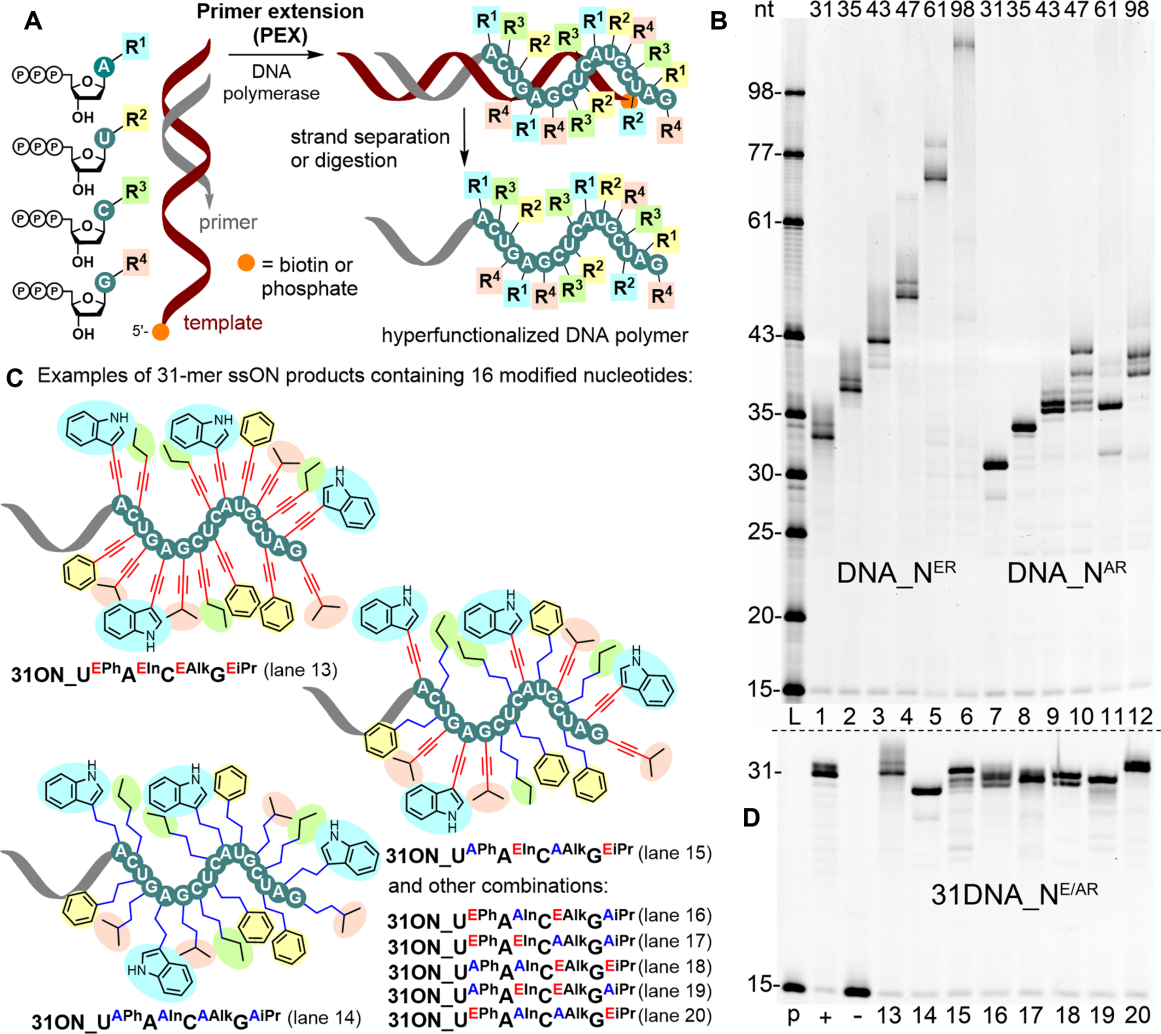
(**A**) Simultaneous incorporation of four modified **dN^R^TP**s using sequence specific templates in primer extension reaction. (**B**) Denaturing PAGE analysis of PEX reactions with set of modified **dN^ER^TP**s and **dN^AR^TP**s performed on 31-mer (lanes 1 and 7), 35-mer (lanes 2 and 8) 43-mer (lanes 3 and 9), 47-mer (lanes 4 and 10), 61-mer (lanes 5 and 11) and 98-mer (lanes 6 and 12) template using Vent (exo-) DNA polymerase. Lane L shows ssON ladder in corresponding sizes. (**C**) 31-mer ssONs achieved after PEX in various combinations of modified **dN^ER^TP**s and **dN^AR^TP**s followed by magnetoseparation. (**D**) Denaturing PAGE analysis of 31-mer PEX products in various combinations of **dN^ER^TP**s and **dN^AR^TP**s with negative (no dNTPs) and positive control (natural dNTPs) which serves as a size marker. Lanes are marked according to 1C.

Then we tested all the eight modified nucleotides (**dU^EPh^TP**, **dA^EIn^TP**, **dG^EiPr^TP**, **dC^EAlk^TP**, **dU^APh^TP**, **dA^AIn^TP**, **dG^AiPr^TP** and **dC^AAlk^TP**) as substrates for DNA polymerases (KOD XL and Vent (exo-)). We started with simple primer extension (PEX) experiments with 19-mer and 31-mer template with 15-mer primer using one modified nucleotide in presence of the other three natural dNTPs (the lists of all oligonucleotide sequences are given in Tables S4 and S5 in SI). In all cases we obtained full-length products (Supplementary Figures S1, S2 and Tables S6 and S7 in SI). Simultaneous incorporation of all four modified nucleotides is much more challenging (Figure 1A). We tested PEX reactions with four different modified nucleotides using templates of different length (31, 35, 43, 47, 61, 98-mer) coding for 16–83 modified nucleotides in a row again with Vent (exo-) and KOD XL (a mixture of KOD polymerase from *Thermococcus kodakaraensis* and its exonuclease deficient mutant) (80) DNA polymerases. In most cases with shorter templates, we observed formation of hypermodified full-length PEX products on polyacrylamide gel (PAGE) (Figure 1B, Scheme S4 and Supplementary Figures S3 and S4 in SI). The electrophoretic mobility of modified PEX products often differed significantly compared to a native DNA of the same size which is caused by the increased hydrophobic character and higher mass to charge ratio of the modified ONs (54). The smears that were observed in some of the PEX product bands may arise from secondary structures with different stability or higher aggregates that cannot be resolved under the conditions applied in standard denaturing PAGE electrophoretic analysis. Using 5′-biotinylated templates, the modified ssONs were separated from template strand by incubating the reaction mixture with streptavidin-coated magnetic beads followed by capturing of the beads with a magnet, washing and release of the modified ON through denaturation in hot water (63). The modified ssONs were characterized by MALDI-TOF analysis (Supplementary Table S8 in SI). Modified ssONs were also prepared by preferential digestion of 5′-phosphorylated templates with λ exonuclease (81,82) (Supplementary Figures S5 in SI).

The results of the PEX reaction differed depending on the linker. When using the rigid ethynyl-linked **dN^ER^TP**s, the PEX reactions gave full-length products even with 83 modified nucleotides (98-mer template). On the other hand, the PEX reactions using flexible alkyl-linked **dN^AR^TP**s typically stopped after incorporation of 22 modified nucleotides in a row. We assume, that synthesis with alkyl-linked nucleotides stops due to strong duplex destabilisation (which was proven by thermal denaturation study, vide infra) and increased hydrophobicity which may result in limited solubility of the PEX product in the aqueous reaction mixture. This difference also confirms previous reports (83,84) showing that ethynyl-substituted dNTPs are better substrates for polymerases than alkyl-linked nucleotides. We also observed that the DNA polymerase using ethynyl-linked **dN^ER^TP**s in the PEX often added an additional nucleotide in an untemplated fashion at the 3′-end of primer extension resulting in 3′-overhanged ON (mostly containing an additional **dA^EIn^**) as confirmed by MALDI-TOF (Supplementary Figures S23–S51 in SI). Similar non-templated additions have been reported frequently and their formation can be mitigated by using bulky 5′-TINA-labelled templates (85).

The successful PEX reactions using all four modified dNTPs can be easily used for generation of large libraries of sequence-specifically hypermodified polymers. Even in the relatively short 31-mer PEX products, the library of all possible 16-mer modified sequences would theoretically generate 4^16^ permutations that is over 4.3 billion different compounds. However, the diversity can be even further increased. Using the 31-mer template, we also performed a series of PEX reactions using different combinations of two ethynyl-linked and two alkyl-linked **dN^R^TP**s and we obtained a small library of eight full-length 31-mer PEX products containing different combinations of 16 modified nucleotides from one single template sequence (Figure 1C, D and Supplementary Figures S3, S4 in SI). This gives another level of complexity and diversity of the modified polymers we can generate by this simple approach.

In order to synthesize short <20-mer fully modified ssONs which can serve as modified primers for either PEX or PCR reactions, nicking enzyme amplification reaction (NEAR) (50) was studied (Scheme S5 in SI). Under optimized conditions, linear amplification of 17-mer fully modified ON was observed when using the set of all four modified **dN^ER^TPs**. The fully modified **17ON_N^ER^** was detected on PAGE by radiolabeling and also confirmed by MALDI-TOF measurement (Supplementary Figure S18 in SI). On the other hand, using modified **dN^AR^TPs**, fully modified ON was prematurely nicked by nicking enzyme during the primer extension before the DNA polymerase was able to synthesize full-length product resulting in inseparable mixture of oligonucleotides.

**Figure 2.**
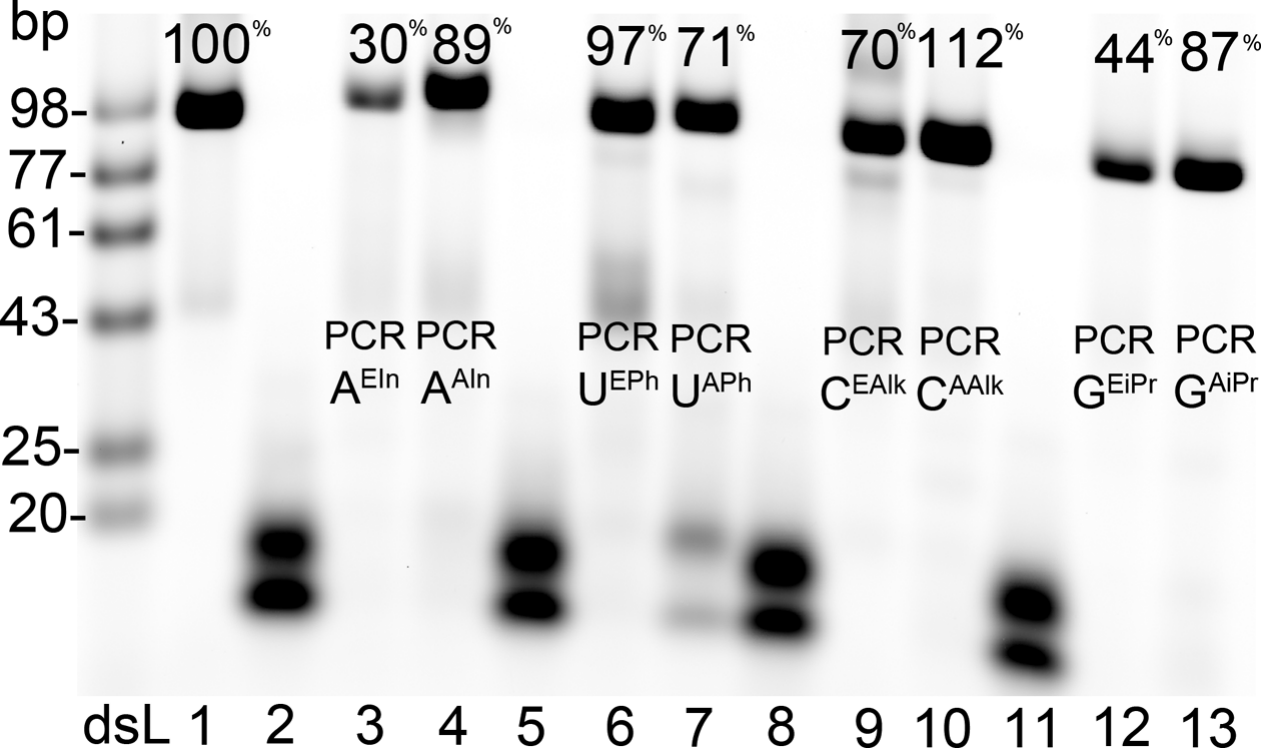
Agarose gel of 5′-(6-FAM)-labelled PCR products using one modified **dN^R^TP**: modified **dA^EIn^** (lane 3), **dA^AIn^** (lane 4), **dU^EPh^** (lane 6), **dU^APh^** (lane 7), **dC^EAlk^** (lane 9), **dC^AAlk^** (lane 10), **dG^EiPr^** (lane 12), **dG^AiPr^** (lane 13) or natural dNTPs (lane 1). Lanes 2, 5, 8 and 11 are control lanes with an absence of the particular modified **dN^R^TP** under study. PCR efficiencies were obtained relative to the PCR product with natural dNTPs (lane 1); (ds) double-stranded ladder. Fluorescence quantification was carried out using ImageJ.

The PEX reactions confirmed that the modified **dN^R^TP**s are suitable substrates for DNA polymerases but for the applications in selection experiments, we needed to investigate their use in the PCR amplification. In PCR, not only the modified **dN^R^TP**s need to be good substrates (even in longer sequences) but also the polymerase needs to be able to read fully-modified template. At first, we studied each of the **dN^ER^TP**s in PCR reactions in the presence of the other three natural dNTPs using Vent (exo-) DNA polymerase (Figure 2). After 30 cycles, generation of full-length PCR amplicons were observed in 30–112% relative efficiency compared to PCR with natural dNTPs.

**Figure 3.**
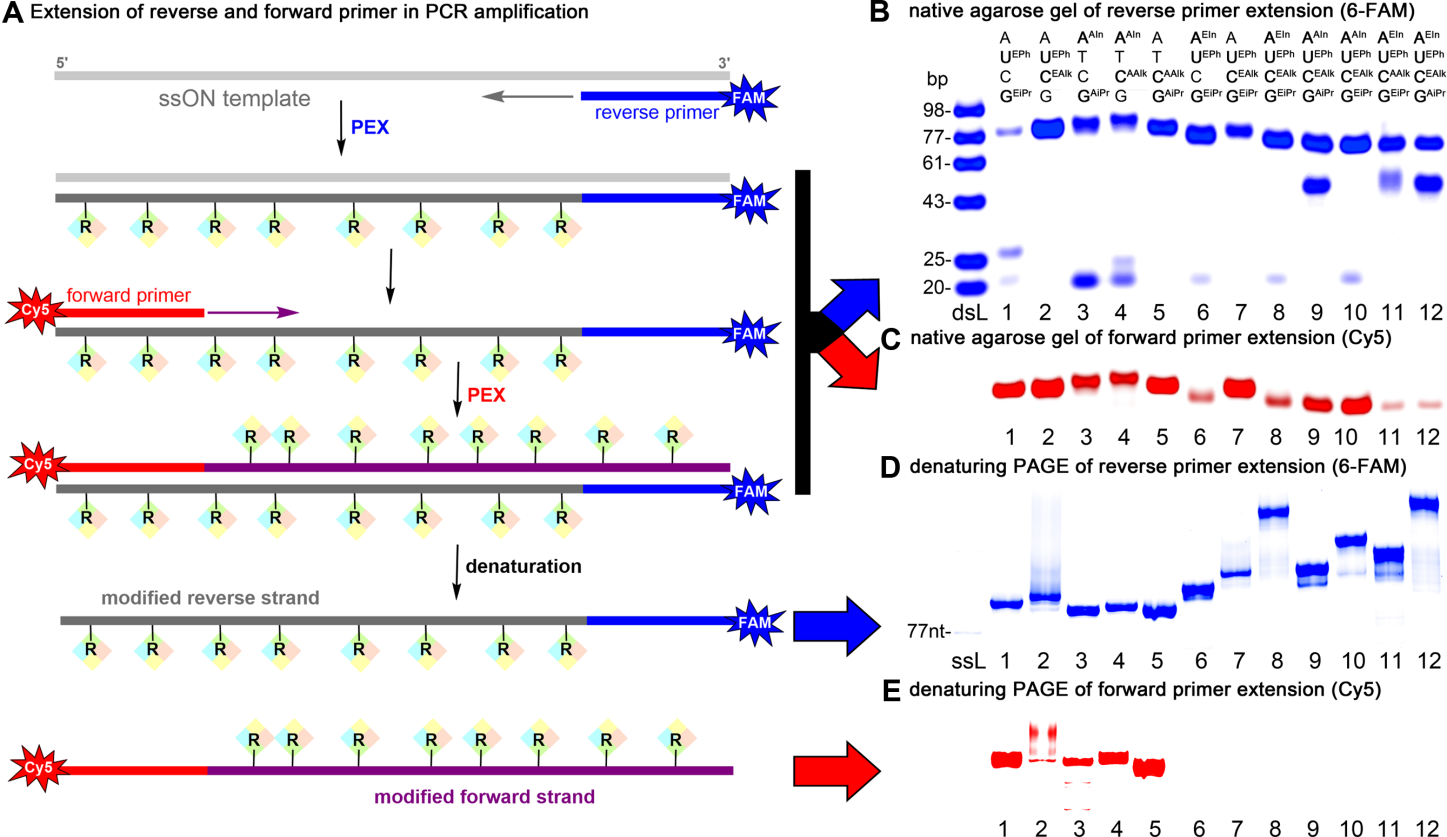
The final combinatorial screen of modified **dN^R^TP**s in PCR. (**A**) Scheme of reverse and forward primers extension in PCR; (**B**) native agarose gel of PCR products with 5′-(6-FAM)-labelled reverse primer, Vent (exo-) DNA polymerase, 77-mer template and respective combination of two (lanes 1–5), three (lanes 6, 7) or four (lanes 8–12) modified **dN^R^TP**s; (**C**) cutout of native agarose gel of corresponding PCR products with Cy5-labelled reverse primer; (**D**) denaturing PAGE of extended 5′-(6-FAM)-labelled reverse primer in corresponding PCR products; (**E**) denaturing PAGE of extended Cy5-labelled forward primer in corresponding PCR products; (dsL) double-stranded ladder; (ssL) single-stranded ladder.

PCR reactions using multiple modified **dN^R^TP**s are much more challenging and the previous literature examples (66–68) were only performed with short sequences and without a conclusive proof of the exponential amplification. We have performed a systematic study of PCR reactions with different combinations of two, three and four modified **dN^R^TP**s. In most cases, we observed an amplified DNA product but the intensity varied and the primers were not consumed in the same level suggesting that only linear amplification might have occurred (Supplementary Table S9, Supplementary Figure S6 and S7 in SI). To verify if the exponential amplification with extension of both primers and replication of modified templates took place, we designed a series of experiments using differently labelled primers (Figure 3A). The reverse primer was labelled with 6-FAM while the forward primer with Cy5. Then we performed the PCR reactions using 77-nt ssON template, differently labelled primers and different combinations of two, three or four modified **dN^R^TP**s. In all cases, we observed formation of amplified modified DNA products with the appropriate electrophoretic mobility in native agarose gels using either detection of 6-FAM (Figure 3B) or Cy5 (Figure 3C, for unmodified original gels, see Supplementary Figure S8 in SI). However, these gels do not sufficiently distinguish between fully extended labelled strand or labelled primer just hybridized to the complementary modified strand. Therefore, we also made denaturing PAGE analyses of the PCR products which separate the strands and visualize only one or the other strand based on the label. Figure 3D shows that in all cases we obtained modified ON using reverse primer complementary to the unmodified ssON template. On the other hand, visualization of the Cy5 label (Figure 3E) shows the presence of fully extended modified strand using forward primer only in case of some combinations of two modified **dN^R^TP**s (lanes 2–5), whereas when using any combinations of three or four modified **dN^R^TP**s the forward PEX using modified ON as template did not proceed. In particular, the combination of **dU^APh^TP** or **dU^EPh^TP** with of **dA^EIn^TP** in the set of modified **dN^R^TPs** are problematic and do not give the exponential amplification even for a combinations of two modified **dN^R^TP**s (Supplementary Figure S7 in SI). This clearly shows that for the combinations of three or four modified **dN^R^TP**s, only linear amplification occurs and the heavily modified ONs do not serve as templates for PCR with modified **dN^R^TP**s. When using unmodified dsDNA as template, we obtained DNA products modified in both strands as products of linear amplifications with both forward and reverse primers (Supplementary Figure S11 in SI).

**Figure 4.**
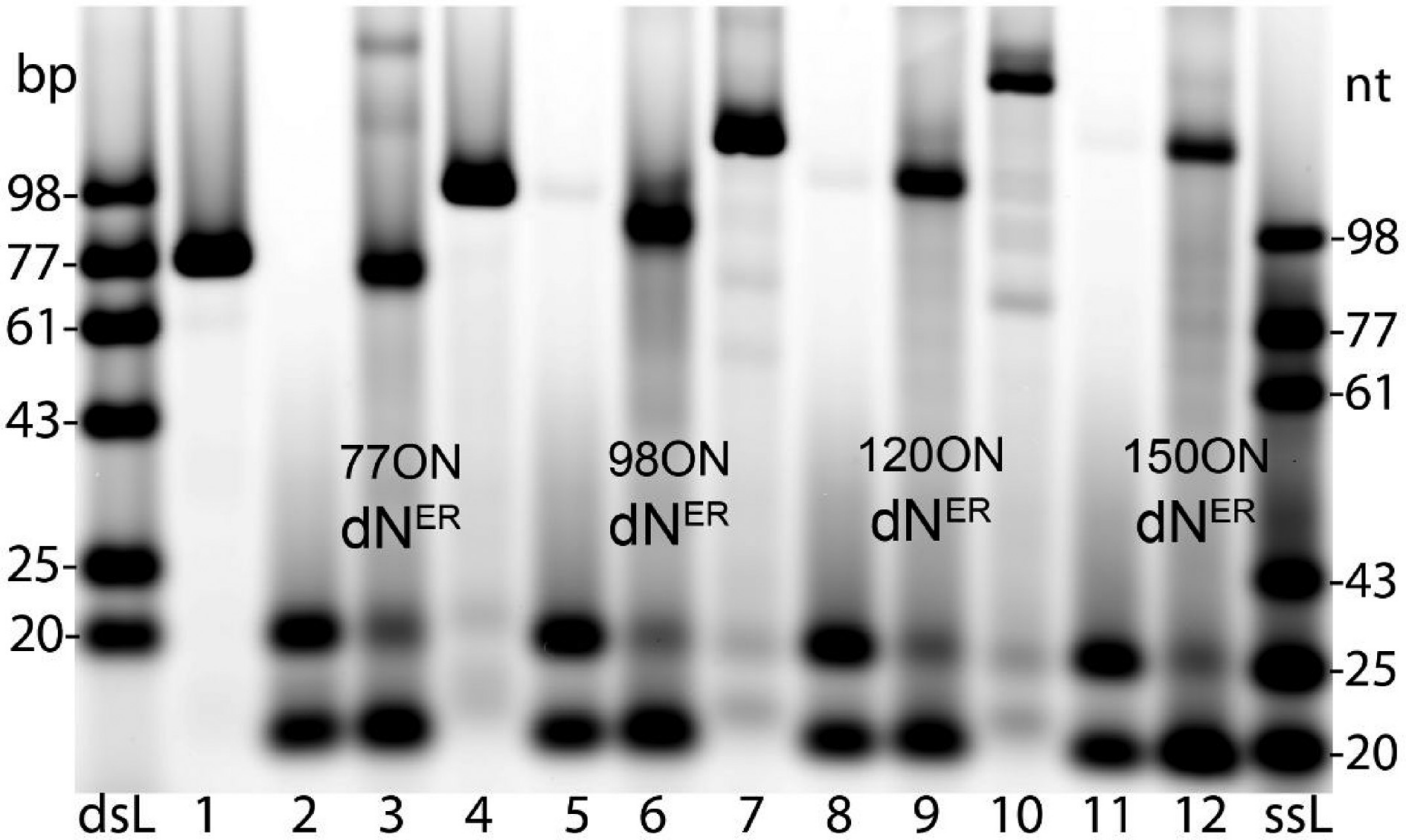
Native agarose gel of 5′-(6-FAM)-labelled aPCR products using four modified ethynyl-linked **dN^ER^TP**s, Vent (exo-) and 77-nt (lane 3), 98-nt (lane 6), 120-nt (lane 9) and 150-nt (lane 12) templates. For each template, positive (lanes 1, 4, 7, 10, natural dNTPs) and negative controls (lanes 2, 5, 8, 11, no dNTPs) were carried out; (dsL) double-stranded ladder; (ssL) single-stranded ladder.

Since the alkyl-linked nucleotides **dN^AR^TP**s could be used only for synthesis of short ONs with 22 nucleotides in a row, we further optimized the PCR synthesis of fully-modified DNA with the set of four ethynyl-linked **dN^ER^TP**s. For optimization of demanding templates, we screened various thermostable B-family DNA polymerases (KOD XL, LongAmp, Pwo, Deep Vent (exo-) and Vent (exo-)). However, even extensive optimization did not lead to exponential amplification of templates under PCR conditions. In all cases, we observed the formation of modified ssONs and consumption of only forward primer suggesting that only asymmetric PCR (aPCR) (86) proceeded with linear amplification (Figure 4). The use of four-component set of additives (DMSO, formamide, betaine and TMAC) slightly increased the yield of the amplification but still only, the linear aPCR proceeded. Unfortunately, the previous literature examples (66–68) did not present relevant proofs (see above) to distinguish whether they achieved a linear or exponential amplification, so no direct comparison could be done. Nevertheless, using the optimized conditions, we were able to perform the aPCR using 77, 98, 120 or 150-mer template generating ssONs containing 52, 73, 95 or 125 modified nucleotides in a row (linked to a 25-mer natural primer, Supplementary Table S10 and Supplementary Figures S9 and S10 in SI). There was also significant sequence dependence: A- and T-rich sequences were synthesized more easily than the C- and G-rich modified oligonucleotides (Supplementary Figure S12 in SI). In all cases, the resulting modified ssONs showed higher electrophoretic mobility compared to natural PCR products (which are dsDNA).

**Scheme 2.**
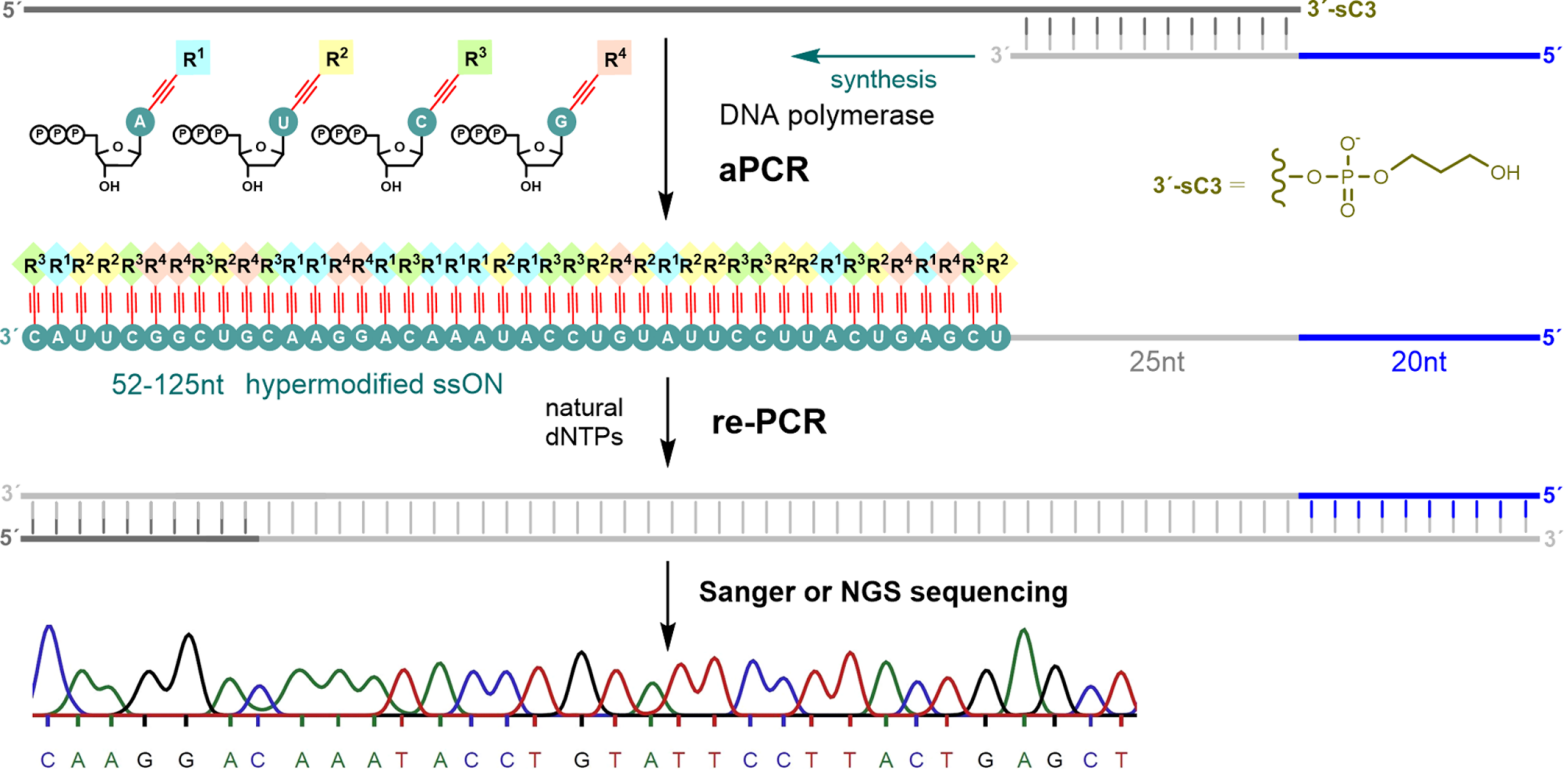
Asymmetric PCR synthesis using set of four modified **dN^ER^TP**s, followed by re-PCR with natural dNTPs to prepare dsDNA for Sanger sequencing and NGS.

In order to be useful for selection experiments, the hypermodified ON polymers need to be sequencable by one of the DNA sequencing methods. In the previous works (66–68), the authors performed PEX or PCR on biotinylated template, separated the template with streptavidin-linked agarose, and the remaining ONs were re-PCRed with natural nucleotides and submitted for Sanger sequencing—however, in that experimental set up, one cannot rule out that they may have amplified traces of the original unmodified template (which might not have been completely removed with streptavidin) and then sequenced it. To unequivocally avoid any possible bias through re-PCR of the original unmodifed template in sequencing, we designed a new construct (Scheme 2). We extended the 5′-end of the primer with additional twenty nucleotide sequence which served as a new primer region for re-PCR. The template was modified at 3′-end with three carbon spacer (sC3) preventing any extension during aPCR. Modified ssDNA strand produced in aPCR was purified by HPLC and then used as a template for re-PCR reaction in presence of Vent (exo-) (Supplementary Figure S13 in SI) or Kappa HiFi DNA Polymerase (Supplementary Figure S16 in SI) within the NGS processing. The PCR product was then subjected to Sanger sequencing and NGS confirming the full fidelity of the replication of hypermodified ON (Supplementary Figures S14, S15, S17). Apparently, the fully-modified ONs are relatively difficult templates but the thermostable polymerases still can use them as templates for PCR with natural dNTPs (but not with modified **dN^R^TP**s). These results show the promising potential of the long hypermodified ONs in selection experiments where the successfully binding sequences can be re-PCRed into natural DNA for amplification followed by sequencing identification.

Modifications attached at positions C5 of pyrimidines and C7 of 7-deazapurines do not interfere with the Watson-Crick base pairing and presumably do not disturb helical structure of B-DNA. However, the situation can be dramatically different if the DNA is modified with hydrophobic groups in aqueous solutions which may in principle lead to alternative secondary structures due to hydrophobic effect of the substituents. To understand how the hydrophobic modifications influence the structure and properties of DNA, thermal denaturation has been studied as well as UV—vis absorption and circular dichroism (CD) spectroscopy for the short 31bp DNA duplexes containing 16 ethynyl- or alkyl-modified nucleotides (**31DNA_N^ER^** or **31DNA_N^AR^)** or longer 77bp DNA duplex containing 52 ethynyl-linked modified bases (**77DNA_N^ER^, 77DNA_dsN^ER^)**.

Significant duplex stabilization was previously reported for oligonucleotides containing 7-propynyl derivatives of 8-aza-7-deazapurines (87,88) or pyrimidines (89,90). Also in our hands, the 31bp ethynyl-modified **31DNA_N^ER^** showed slight duplex stabilization manifested by increase of *T*_m_ by 1.8 °C (Table 1, Supplementary Figure S19 in SI). Presumably, it is caused by the combination of electronic effect on Watson—Crick base-pairing and enhanced polarizability and π-π-stacking of the ethynyl-modified nucleobases compared to natural counterpart (87,88). In the case of DNA duplex bearing flexible alkyl substituents **31DNA_N^AR^**, the *T*_m_ value decreased by 11.1°C showing a strong destabilization of duplex. This destabilization may be caused by increased hydrophobicity and perturbation of the hydration network surrounding the DNA duplex. However, both **31DNA_N^ER^** and **31DNA_N^AR^** can be denaturated and re-hybridized repeatedly without changing the *T*_m_ values even though the modified ssON may hypothetically fold to some micelle-type structure hiding the hydrophobic groups inside. In presence of the complementary strand, it can adopt the duplex structure again. The repeated denaturation and re-hybridization of the longer heavily modified **77DNA_N^ER^** was also studied. Analogous hysteresis (difference between *T*_m_ and annealing temperature *T*_a_) of **77DNA_N^ER^** compared to its natural counterpart **77DNA** indicates that the re-hybridization kinetics of the presumably folded hypermodified ssON is comparable to re-hybridization of a natural DNA of the same length.

**Table 1.** Melting temperatures (*T*_m_) and hysteresis of natural and modified DNA determined by UV spectroscopy

DNA title	*T*_m_ (°C)	Hysteresis = *T*_m_−*T*_a_ (°C)	Δ*T*_m_ / modification
**31DNA**	71.0	3.4	-
**31DNA_N^ER^**	72.8	4.0	+0.113
**31DNA_N^AR^**	61.7	3.2	−0.694
**77DNA**	74.4	3.9	-
**77DNA_N^ER^**	77.0	3.8	+0.050

**Figure 5.**
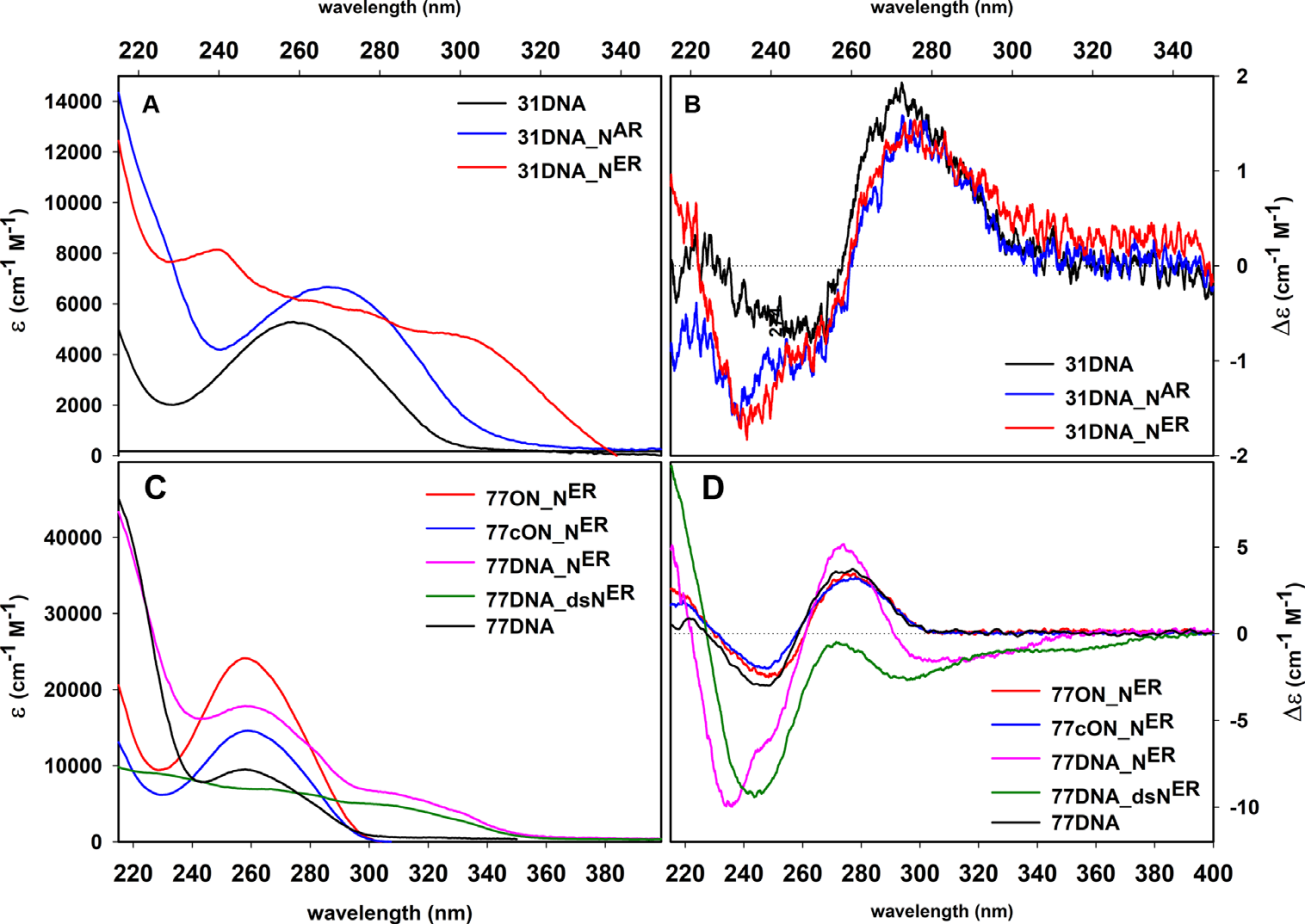
UV absorption spectra (**A**) and CD spectra (**B**) of **31DNA**, **31DNA_N^ER^** and **31DNA_N^AR^**; UV absorption spectra (**C**) and CD spectra (**D**) of sequence-heterogeneous **77DNA**, **77DNA_N^ER^** and **77DNA_dsN^ER^**and their corresponding modified single strands (**77ON_N^ER^**, **77cON_N^ER^**); all measurements were carried out in TrisHCl buffer (10 mM, 1 mM EDTA, 65 mM NaCl, pH 8.0).

In order to correctly interpret the absorption and CD spectra of modified DNA, we first measured the UV and CD spectra of the modified **dN^R^TPs** (Supplementary Figure S20, S21 in SI). While the non-conjugated alkyl-linked substituents had little effect on the absorption spectra of the modified nucleotides, the conjugated ethynyl-linked nucleotides showed a significant bathochromic shift of absorption maxima to 300—324 nm. Next, we measured the absorption spectra of model 31bp DNA modified with 16 nucleotides attached either via ethynyl (**31DNA_N^ER^**) or via alkyl (**31DNA_N^AR^**) spacers. The UV—vis absorption spectrum of alkyl-modified DNA **31DNA_N^AR^** was very similar to natural **31DNA** with only small shift of absorption maximum from 260 to 270 nm. On the other hand, absorption of ethynyl-linked **31DNA_N^ER^** exerted a new distinct band at ca. 310 nm (Figure 5A). CD spectroscopy was measured with both types of shorter hypermodified 31bp **31DNA_N^ER^** and **31DNA_N^AR^**. The CD spectra of **31DNA_N^ER^** and **31DNA_N^AR^** possess similar CD patterns as natural DNA duplex **31DNA** characterized by CD couplet with positive and negative maxima at ∼274 nm (+)/∼247 nm (−) indicating B-type DNA conformation, but exerted a new distinct CD minimum at 235 nm for both modifications (Figure 5B).

Absorption and CD spectra were also measured for longer 77-bp ethynyl-modified DNA. We have prepared two complementary modified ssONs (**77ON_N^ER^** and **77cON_N^ER^**) through aPCR. Then we annealed **77ON_N^ER^** with either non-modified complementary ssON (MO77) to give dsDNA modified in one strand (**77DNA_N^ER^**) or we annealed it with complementary modified strand **77cON_N^ER^** to give duplex **77DNA_dsN^ER^** fully modified in both strands (Supplementary Figure S10A in SI). CD spectra of modified ssONs (**77ON_N^ER^** and **77cON_N^ER^**) were not significantly altered by modifications and CD spectra of the longer natural **77DNA** and modified **77DNA_N^ER^** showed similar couplet typical for B-type DNA conformation (Figure 5C, D). The additional negative high intensity bands at 235 nm and low intensity band around 310 nm may have resulted from exciton chirality coupling of the extended π-electron system containing phenyl and indole residues reflecting the possible helical arrangement of the phenyl and indole moieties. Spectral changes at 235 nm and 310 nm can also serve as unambiguous markers of structural changes and can be used for an alternative temperature stability (*T*_m_) measurement (Supplementary Figure S22 in SI). The CD spectrum of the duplex modified in both strands (**77DNA_dsN^ER^**) showed greatly decreased positive band at 275 nm. This spectral change was accompanied by an intensity increase of negative spectral band at 300 nm whereas the other negative band was shifted to 244 nm with comparable intensity as observed for **77DNA_N^ER^**. Lowering intensity of positive band around 275 nm corresponds to DNA duplex changes which reflect the same trend as global changes within DNA wounding known as C-DNA conformation with more tight packing of parallel DNA threads and greater wounding angle (θ) between the adjacent base pairs (91,92).

**Figure 6.**
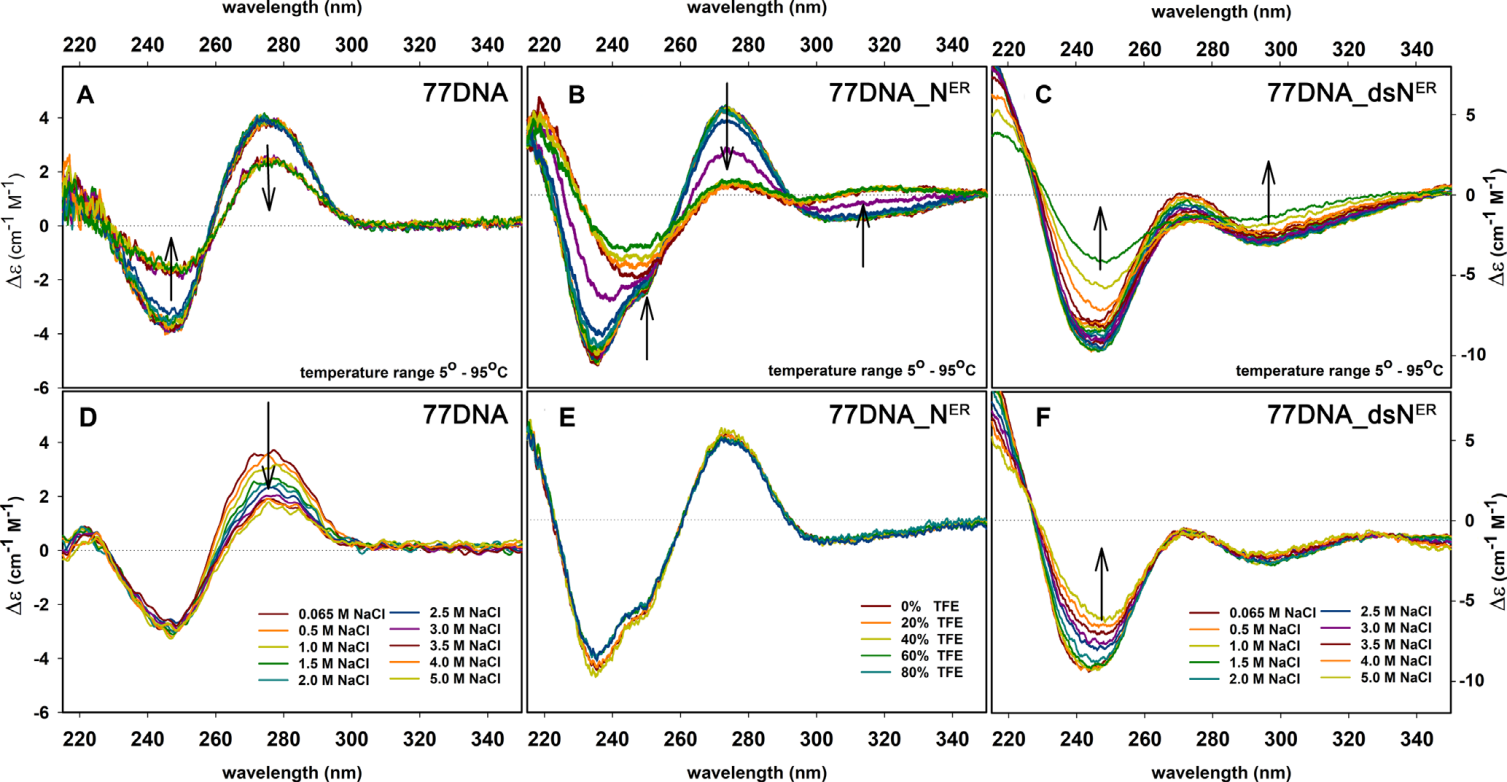
CD spectra reflecting changes within natural **77DNA** and modified **77DNA_N**^ER^, **77DNA_dsN^ER^** duplexes with increasing temperature (**A-C**), concentration of NaCl (**D,F**) and percentage of TFE (**E**).

For further investigation of modified duplexes, we also studied temperature dependence of CD spectra which showed that the helical arrangement of modifications is retaining the comparable temperature stability as DNA backbone (Figure 6B, C). Other types of DNA heavily modified in both strands (made by PCR using modified dNTPs) have been previously reported (85,93,94) to exhibit inverted CD spectrum suggesting transition of right-handed B-type DNA to left-handed Z-type DNA. The secondary structure conversions within DNA polymorphism can be also induced by changing the environment (91).

To show whether modified **77DNA_N^ER^**and **77DNA_dsN^ER^** could change its conformation depending on its environment, we further carried out measurements under greater ionic strength by NaCl titration (B-DNA to C-DNA transition) (Figure 6D, F) and under anhydrous conditions by trifluoroethanol (TFE) titration (B-DNA to Z-DNA transition) (Figure 6E). As expected, CD magnitude of natural **77DNA** at 275 nm decreases gradually with increasing Na^+^ concentration forcing natural DNA to more wound conformation. Addition of NaCl to already tightly packed modified **77DNA_dsN^ER^** duplex does not alter so significantly than modifications themselves and rather causes opposite changes in 245 nm region. There were also no significant changes observed with TFE titration also suggesting no conformational change out of B-family DNA structures.

All these experiments confirmed that our sequence-heterogeneous DNA duplexes composed of one fully-modified and one non-modified strand (**77DNA_N^ER^**) and even fully modified DNA duplex (**77DNA_dsN^ER^**) do not undergo any cooperative transition and still belong to B-DNA conformational class.

## CONCLUSIONS

We have designed and synthesized a small library of all four dNTPs bearing hydrophobic substituents linked either through ethynyl or alkyl tether. These **dN^R^TP**s served as substrates for DNA polymerases in the synthesis of hypermodified DNA. Using PEX, we were able to generate dsDNA composed of one natural strand and one hypermodified strand. The modified ssON can be easily isolated either using magnetoseparation or digestion of the template by λ exonuclease. Using flexible alkyl-linked **dN^AR^TP**s, we were able to synthesize hypermodified oligonucleotides with up to 22 modified nucleotides in a row and then the extension is terminated (probably due to limited solubility and stability of the modified duplex). However, when using the more rigid alkyne-linked **dN^ER^TP**s, the polymerase can synthesize even longer modified ONs (up to at least 125 modified nucleotides in a row). The exponential amplification in PCR occurred in case of various combinations of two modified **dN^R^TP**s. On the other hand, when using different sets of three and four modified **dN^R^TP**s, exponential amplification did not proceed but we were still able to use aPCR for linear amplification and synthesis of long fully-modified ssONs. Thus, the PEX or aPCR can be used for synthesis of monodispersed polymers based on DNA backbone displaying four different substituents in sequence-specific manner. This robust approach can be used for generation of large libraries of modified polymers. Using four different modified **dN^R^TP**s, only with short 16-mer ONs, there are theoretically 4.3 × 10^9^ possible permutations, whereas with a longer 52-mer ssONs (which we can still easily make using the ethynyl-modified **dN^ER^TP**s), there are 5 × 10^33^ theoretically possible sequences, each displaying the substituents in a unique way. Most importantly, we have designed and developed a new construct for bias-free re-PCR of the modified ONs and demonstrated that the long hypermodified DNA polymers can be replicated with good fidelity and amplified by PCR using natural dNTPs to form natural DNA which could be then sequenced using Sanger or NGS method. Despite of previous studies reporting Z conformation of fully-modified duplexes, here we demonstrated that incorporation of all four hydrophobic modified **dN^R^TPs** into sequence heterogeneous DNA duplex do not significantly alter the overall B-DNA structure. Fully-modified ssONs (prepared by strand separation or aPCR) can presumably adopt different folding depending on the sequence and modification. Therefore, this type of hypermodified ON polymers has a promising potential in generation and selection of aptamers or other functional polymers. Possible combining of these new hydrophobic dNTPs with some previously reported (68,83) or newly designed polar dNTPs should lead to production of hypermodified DNA bearing even wider diversity of functional groups for prospective targeting of wider diversity of (bio)molecules. Although there are still many technical issues with the selections and sequencing which need to be solved, these applications will be further pursued in our labs.

## Supplementary Material

gkaa999_Supplemental_File
