# Supplementary material for: Enzymatic synthesis of hypermodified DNA polymers for sequence-specific display of four different hydrophobic groups
Source: Nucleic Acids Res. 2020 Nov 5;48(21):11982–93. doi: 10.1093/nar/gkaa999 (PMC7708046; doi:10.1093/nar/gkaa999)
Supplement: gkaa999_Supplemental_File [file gkaa999_supplemental_file.pdf]

Supporting Information

Enzymatic Synthesis of Hypermodified DNA for Sequence-Specific Display of Four Different Hydrophobic Groups

Marek Ondruš,[ab] Veronika Sýkorová,[a] Lucie Bednárová,[a] Radek Pohl,[a] and Michal Hocek[ab*]

Supporting information

Table of contents

1) Experimental section – organic chemistry 3
1.1 Synthesis of modified nucleosides – Sonogashira cross-coupling 3
1.2 Synthesis of modified nucleosides – Catalytic hydrogenation 4
1.3 Synthesis of modified nucleotides – 5´-triphosphorylation 6
1.4 Spectral characteristics of prepared compounds: 7
2) Experimental section - biochemistry 18
2.1 PEX - Single incorporation (one modified $dN^{R}TP$) 22
2.2 PEX - Multiple incorporation (one modified $dN^{R}TP$) 23
2.3 PEX - Multiple incorporation (four modified $dN^{R}TPs$ in various combinations) 25
2.4 PEX - Multiple incorporation (four modified $dN^{R}TPs$ in various template length) 26
2.5 General procedure for ssDNA generation via magnetoseparation 27
2.6 General procedure for ssDNA generation via λ-exonuclease digestion 28
2.7 MALDI-TOF measurements 29
2.8 PCR – single incorporation (one modified $dN^{R}TP$) 30
2.9 PCR – multiple incorporation (two, three and four modified $dN^{R}TPs$) 31
2.10 aPCR – multiple incorporation (four modified $dN^{R}TPs$) 33
2.11 Re-PCR of modified ssONs 37
2.12 Sanger sequencing 38
2.13 High-throughput Next-generation Sequencing (NGS) 39
2.14 NEAR - Nicking Enzyme Amplification Reaction 41
3) Experimental section – UV-VIS absorption and CD spectroscopy 43
3.1 UV-VIS spectroscopy 45
3.2 Circular dichroism (CD) spectroscopy 46
4) Copies of MALDI-TOF mass spectra 47
5) Copies of ^{1}H, ^{13}C and ^{31}P NMR spectra of prepared compounds 62
6) References 82

1) Experimental section – organic chemistry

General remarks

NMR spectra were recorded on Bruker Avance III 500 MHz spectrometer (500.0 MHz for ^{1}H, 125.7 MHz for ^{13}C and 202.4 for ^{31}P) from sample solutions in D_2O and DMSO*d*$_6$. Chemical shifts (in ppm, δ scale) were referenced as follows: D_2O (referenced to dioxane as internal standard in 1 mm coaxial capillary; 3.75 ppm for ^{1}H NMR and 69.3 ppm for ^{13}C NMR); DMSO-*d*$_6$ (referenced to solvent signal: 2.50 ppm for ^{1}H NMR and 39.7 ppm for ^{13}C NMR). ^{31}P chemical shifts were referenced to H_3PO_4 (0 ppm) as external reference. Coupling constants (*J*) are given in Hz. Complete assignment of all NMR signals was achieved by using a combination of H,H-COSY, H,C-HSQC, and H,C-HMBC experiments. Mass spectra were measured on LCQ classic (Thermo-Finnigan) spectrometer using ESI or Q-Tof Micro (Waters, ESI source, internal calibration with lockspray). Preparative HPLC separations were performed on a column packed with 10 µm C18 reversed phase (Phenomenex, Luna C18). High-resolution mass spectra were measured on a LTQ Orbitrap XL (Hermo Fischer Scientific) spectrometer using ESI ionization technique. Chemicals were of analytical grade.

1.1 Synthesis of modified nucleosides – Sonogashira cross-coupling

Method A: 1:2 mixture of AN/H_2O (2 mL) was added through a septum to an argon-purged flask containing **dN^{I}** (1 equiv.), TPPTS (11 mol. %), CuI (8 mol. %) and $Pd(OAc)_2$ (7 mol. %) followed by addition of excess of terminal alkyne **1a**-**1d** (Table S1) and TEA (6 equiv.) (Scheme S1). The reaction mixture was stirred at room temperature overnight (48 h in case of **dG^{I}**) and then evaporated under vacuum. The product was purified by FLC chromatography using DCM/MeOH (0-30%) as eluent followed by evaporation under vacuum to get solid product.

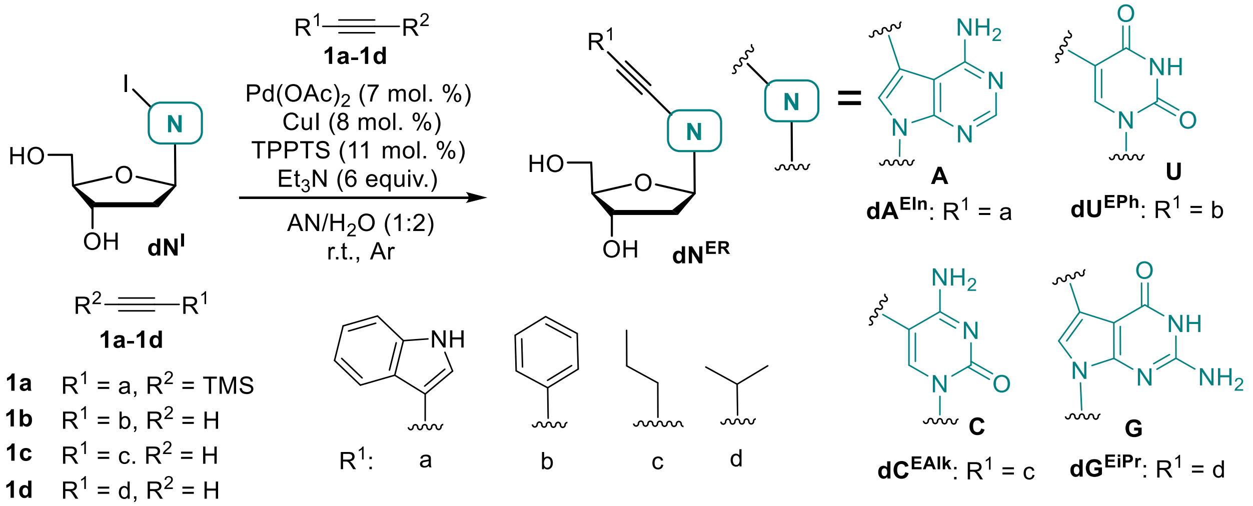

Scheme S1. Reaction scheme of Sonogashira cross-coupling reaction described by method A.

Table S1. Reaction conditions of Sonogashira cross-coupling reaction.

Entry	Starting dN^I	Alkyne	R^1	R^2	Alkyne excess	Additive	Product	Yield (%)
1	**dA^I**	**1a**	3-indolyl	TMS	1.3	NH_4F (5 equiv.)	**dA^{EIn}**	98
2	**dU^I**	**1b**	phenyl	H	10	-	**dU^{EPh}**	88
3	**dC^I**	**1c**	propyl	H	10	-	**dC^{EAlk}**	84
4	**dG^I**	**1d**	isopropyl	H	25	-	**dG^{EiPr}**	69

1.2 Synthesis of modified nucleosides – Catalytic hydrogenation

Method B1: MeOH (5 mL) was added through a septum to an argon-purged flask containing **dN^{ER}** (1 equiv.), 10% Pd/C (10 mol. %) followed by vacuuming and fulfilling with H_2 atmosphere (balloon) (Scheme S2). The reaction mixture was stirred at r.t. (reflux in case of **dU^{EPh}**) for desired time (Table S2) until complete consumption of the starting material and then evaporated under vacuum. The product was purified by FLC chromatography using DCM/MeOH (0-30%) as eluent followed evaporation under vacuum to get solid product.

Method B2: H_2O (2 mL) was added through a septum to an argon-purged flask containing **dC^{EAlk}TP** (1 equiv.), 10% Pd/C (10 mol. %) followed by vacuuming and fulfilling with H_2

atmosphere (balloon). The reaction mixture was stirred at r.t. for 4 h until complete consumption of the starting material and then evaporated under vacuum. The product was isolated by HPLC on a C18 column with use of linear gradient from 0.1 M TEAB in H_2O to 0.1 M TEAB in H_2O/MeOH (1:1) as eluent.

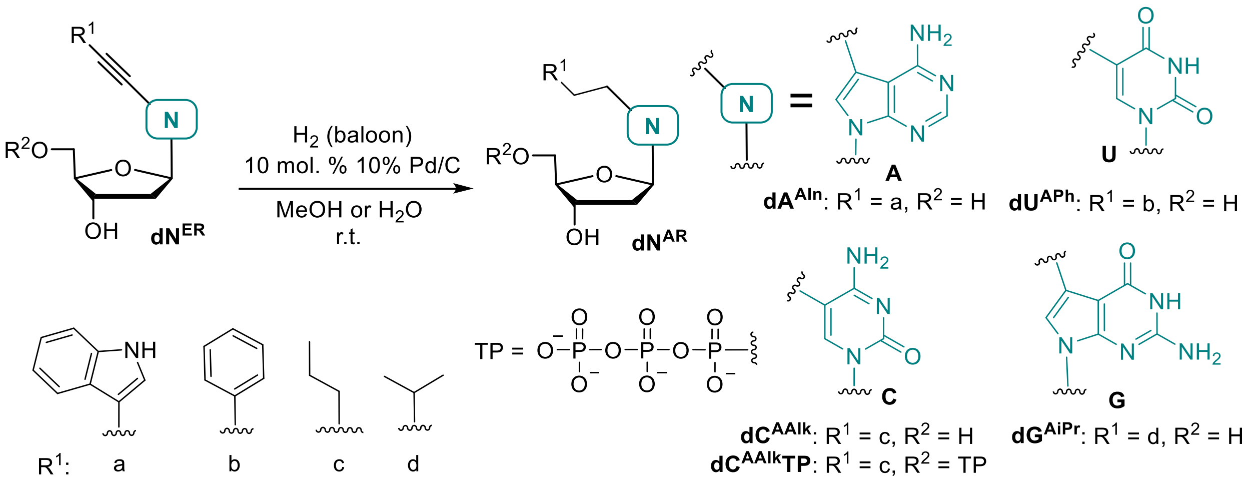

Scheme S2. Reaction scheme of catalytic hydrogenation reaction described by Method B1 and B2.

Table S2. Reaction conditions of catalytic hydrogenation reaction.

Entry	Starting dN^{ER}	R^1	R^2	Solvent	Reaction time	Product	Yield (%)
1	**dU^{EPh}**	3-indolyl	H	MeOH	8 h	**dU^{APh}**	93
2	**dA^{EIn}**	phenyl	H	MeOH	overnight	**dA^{AIn}**	94
3	**dC^{EAlk}**	propyl	H	MeOH	4 h	**dC^{AAlk}**	95
4	**dG^{EiPr}**	isopropyl	H	MeOH	overnight	**dG^{AiPr}**	98
5	**$dC^{EAlk}TP$**	propyl	TP	H_2O	4h	**$dC^{AAlk}TP$**	25

1.3 Synthesis of modified nucleotides – 5´-triphosphorylation

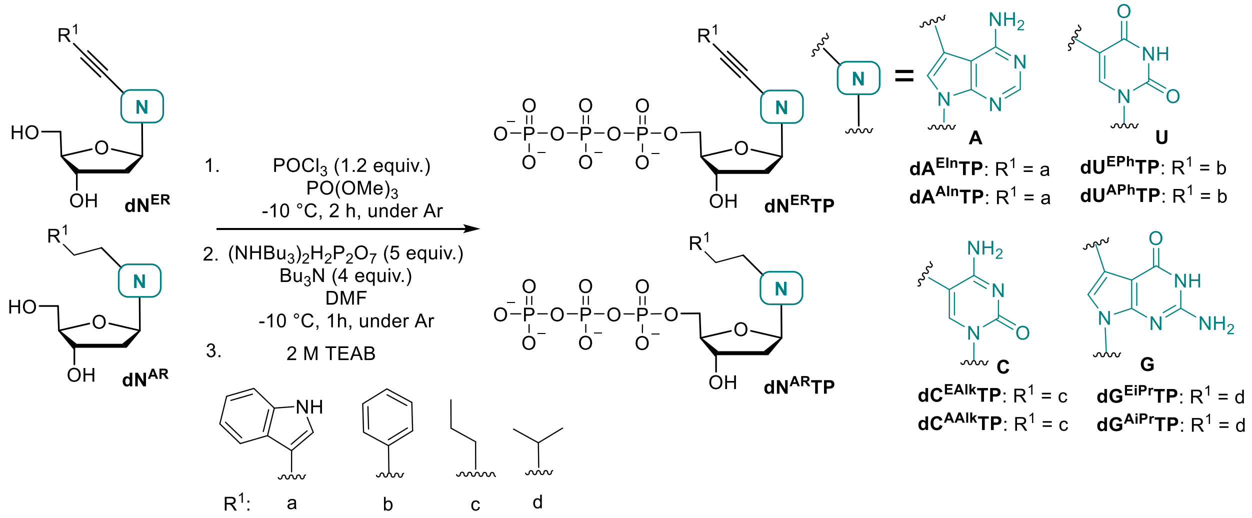

Scheme S3. Reaction scheme of phosphorylation reaction described by method C.

Method C: $PO(OMe)_3$ (1 mL) was added through a septum to an argon-purged flask containing modified nucleosides $\mathbf{dN^{ER}}$ or $\mathbf{dN^{AR}}$ (1 equiv.) followed by dropwise addition of $POCl_3$ (1.2 equiv.) at -10 °C (ice bath + NaCl) and the reaction mixture was stirred for 2 h at -10 °C (Scheme S3). Content of ice-cooled mixture containing solution of $(NHBu_3)_2H_2P_2O_7$ (5 equiv.) and Bu_3N (4 equiv.) in dry DMF (1 mL) was added dropwise and the reaction mixture and stirred for another 1 h at -10 °C. The reaction was quenched by addition of aqueous 2 M TEAB (triethylammonium bicarbonate) (5 mL). Solvents were evaporated under vacuum and co-distilled with water three times. The product was purified by HPLC on a C18 column with use of linear gradient from 0.1 M TEAB in H_2O to 0.1 M TEAB in H_2O/MeOH (1:1) as eluent. Conversion to sodium salt by ion exchange resin Dowex 50WX8 followed by freeze-drying from water gave solid product (Table S3).

Table S3. Starting materials, products and yields of 5´-triphosphorylation reaction.

Entry	Starting dN^{R}	Product	Yield (%)
1	$\mathbf{dU^{EPh}}$	$\mathbf{dU^{EPh}TP}$	20
2	$\mathbf{dU^{APh}}$	$\mathbf{dU^{APhe}TP}$	28
3	$\mathbf{dA^{EIn}}$	$\mathbf{dA^{EIn}TP}$	22
4	$\mathbf{dA^{AIn}}$	$\mathbf{dA^{AIn}TP}$	21
5	$\mathbf{dC^{EAlk}}$	$\mathbf{dC^{EAlk}TP}$	21
6	$\mathbf{dC^{AAlk}}$	$\mathbf{dC^{AAlk}TP}$	28
7	$\mathbf{dG^{EiPr}}$	$\mathbf{dG^{EiPr}TP}$	18
8	$\mathbf{dG^{AiPr}}$	$\mathbf{dG^{AiPr}TP}$	13

1.4 Spectral characteristics of prepared compounds:

5-(2-Phenyl-1-ethyn-1-yl)-2´-deoxyuridine (dU$^{\mathbf{EPh}}$)

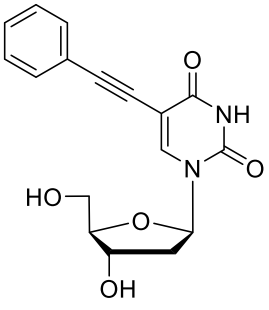

dU$^{\mathbf{EPh}}$

Compound **dU$^{\mathbf{EPh}}$** was prepared from **dU$^{\mathbf{I}}$** using Method A. The spectral data were in accordance with literature.[1] ^{1}H NMR (500.0 MHz, DMSO-d_6): 2.14 (ddd, 1H, J_{gem} = 13.3, $J_{2'b,1'}$ = 6.3, $J_{2'b,3'}$ = 4.0, H-2'b); 2.18 (ddd, 1H, J_{gem} = 13.3, $J_{2'a,1'}$ = 7.0, $J_{2'a,3'}$ = 5.8, H-2'b); 3.59, 3.66 (2 × ddd, 2 × 1H, J_{gem} = 11.8, $J_{5',OH}$ = 4.8, $J_{5',4'}$ = 3.4, H-5'); 3.81 (q, 1H, $J_{4',3'}$ = $J_{4',5'}$ = 3.4, H-4'); 4.26 (dddd, 1H, $J_{3',2'}$ = 5.8, 4.0, $J_{3',OH}$ = 4.3, $J_{3',4'}$ = 3.4 , H-3'); 5.17 (t, 1H, $J_{OH,5'}$ = 4.8, OH-5'); 5.27 (d, 1H, $J_{OH,3'}$ = 4.3, OH-3'); 6.13 (dd, 1H, $J_{1',2'}$ = 7.0, 6.3, H-1'); 7.37 – 7.44 (m, 3H, H-*m,p*-Ph); 7.44 – 7.49 (m, 2H, H-*o*-Ph); 8.39 (s, 1H, H-6); 11.70 (s, 1H, NH).

^{13}C NMR (125.7 MHz, DMSO-d_6): 40.39 (CH_2-2'); 61.01 (CH_2-5'); 70.11 (CH-3'); 82.67 (C5-**C**≡C-Ph); 85.02 (CH-1'); 87.77 (CH-4'); 91.95 (C5-C≡**C**-Ph); 98.33 (C-5); 122.58 (C-*i*-Ph); 128.84 (CH-*p*-Ph); 128.93 (CH-*m*-Ph); 131.34 (CH-*o*-Ph); 144.10 (CH-6); 149.62 (C-2); 161.62 (C-4).
MS (ESI): *m/z*: 351.1 [M+Na].
HRMS (ESI): *m/z*: [M+Na] calcd for: $C_{17}H_{16}O_5N_2Na$: 351.09514; found: 351.09531.
m.p.: 173-175°C.

5-(2-Phenylethyl)-2´-deoxyuridine (dU$^{\mathbf{APh}}$)

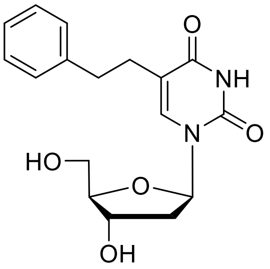

dU$^{\mathbf{APh}}$

Compound **dU$^{\mathbf{APh}}$** was prepared from **dU$^{\mathbf{EPh}}$** using Method B1. The spectral data in $CDCl_3$ and D_2O are published in literature.[2] ^{1}H NMR (500.0 MHz, DMSO-d_6): 1.94 (ddd, 1H, J_{gem} = 13.3, $J_{2'b,1'}$ = 7.6, $J_{2'b,3'}$ = 6.0, H-2'b); 2.02 (ddd, 1H, J_{gem} = 13.3, $J_{2'a,1'}$ = 6.2, $J_{2'a,3'}$ = 3.3, H-2'a); 2.41 – 2.58 (m, 2H, U-C**H$_2$**CH_2-Ph); 2.68 – 2.79 (m, 2H, U-CH_2C**H$_2$**-Ph); 3.50, 3.55 (2 × ddd, 2 × 1H, J_{gem} = 11.8, $J_{5',OH}$ = 5.1, $J_{5',4'}$ = 3.8, H-5'); 3.74 (td, 1H, $J_{4',5'}$ = 3.8, $J_{4',3'}$ = 3.0, H-4'); 4.21 (dddd, 1H, $J_{3',2'}$ = 6.0, 3.3, $J_{3',OH}$ = 4.3, $J_{3',4'}$ = 3.0 , H-3'); 5.03 (t, 1H, $J_{OH,5'}$ = 5.1, OH-5'); 5.22 (d, 1H, $J_{OH,3'}$ = 4.3, OH-

3'); 6.14 (dd, 1H, $J_{1',2'}$ = 7.6, 6.2, H-1'); 7.15 – 7.20 (m, 3H, H-*o,p*-Ph); 7.25 – 7.30 (m, 2H, H-*m*-Ph); 7.59 (s, 1H, H-6); 11.30 (s, 1H, NH).

^{13}C NMR (125.7 MHz, DMSO-d_6): 28.33 (U-**C**$H_2$$CH_2$-Ph); 34.04 (U-$CH_2$**C**$H_2$-Ph); 39.67 ($CH_2$-2'); 61.44 ($CH_2$-5'); 70.55 (CH-3'); 83.96 (CH-1'); 87.44 (CH-4'); 112.89 (C-5); 126.04 (CH-*p*-Ph); 128.47 (CH-*m*-Ph); 128.52 (CH-*o*-Ph); 136.75 (CH-6); 141.38 (C-*i*-Ph); 150.45 (C-2); 163.49 (C-4).

MS (ESI): *m/z*: 355.1 [M+Na].
HRMS (ESI): *m/z*: [M+Na] calcd for: $C_{17}H_{20}O_5N_2Na$: 355.12644; found: 355.12653.
m.p.:191-194°C.

5-(2-Phenyl-1-ethyn-1-yl)-2´-deoxyuridine triphosphate **(dU^{EPh}TP)**

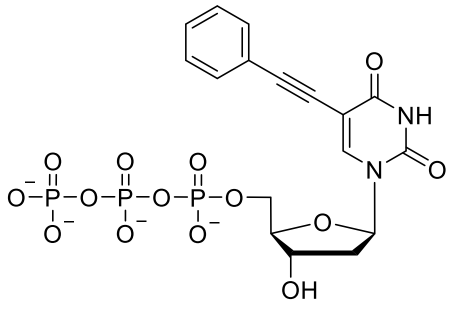

dU^{EPh}TP

Compound **dU^{EPh}TP** was prepared from **dU^{EPh}** using Method C. ^{1}H NMR (500.0 MHz, D_2O): 2.41 (ddd, 1H, J_{gem} = 14.3, $J_{2'b,1'}$ = 7.1, $J_{2'b,3'}$ = 5.9, H-2'b); 2.44 (ddd, 1H, J_{gem} = 14.3, $J_{2'a,1'}$ = 6.6, $J_{2'a,3'}$ = 4.6, H-2'b); 4.17 – 4.28 (m, 3H, H-4',5'); 4.67 (ddd, 1H, $J_{3',2'}$ = 5.9, 4.6, $J_{3',4'}$ = 3.1 , H-3'); 6.31 (dd, 1H, $J_{1',2'}$ = 7.1, 6.6, H-1'); 7.41 – 7.46 (m, 3H, H-*m,p*-Ph); 7.61 – 7.66 (m, 2H, H-*o*-Ph); 8.21 (s, 1H, H-6).

^{13}C NMR (125.7 MHz, D_2O): 41.34 (CH_2-2'); 67.95 (d, $J_{C,P}$ = 5.4, CH_2-5'); 73.08 (CH-3'); 82.66 (C5-**C**≡C-Ph); 88.35 (d, $J_{C,P}$ = 8.9, CH-4'); 88.44 (CH-1'); 96.72 (C5-C≡**C**-Ph); 102.56 (C-5); 124.57 (C-*i*-Ph); 131.36 (CH-*m*-Ph); 131.77 (CH-*p*-Ph); 134.32 (CH-*o*-Ph); 146.85 (CH-6); 153.43 (C-2); 167.45 (C-4).

^{31}P{^{1}H} NMR (202.4 MHz, D_2O): -22.44 (bt, J = 19.7, P_β); -11.20 (d, J = 19.7, P_α); -7.82 (bs, P_γ).

MS (ESI): *m/z*: 487.0 [M-PO_3Na-2Na+H]$^-$; 509.0 [M-PO_3Na-Na]$^-$; 567.0 [M-3Na+2H]$^-$; 589.0 [M-2Na+H]$^-$; 611.0 [M-Na]$^-$.

HRMS (ESI): *m/z*: calcd for $C_{17}H_{18}O_{14}N_2P_3$ [M-3Na+2H]$^-$: 566.99764; found: 566.99740. calcd for $C_{17}H_{17}O_{14}N_2P_3Na$ [M-2Na+H]$^-$: 588.97958; found: 588.97928.

5-(2-Phenylethyl)-2´-deoxyuridine triphosphate **(dU$^{\mathbf{APh}}$TP)**

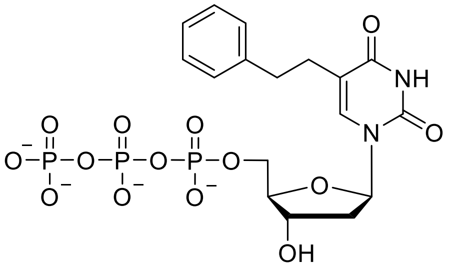

dU$^{\mathbf{APh}}$TP

Compound **dU$^{\mathbf{APh}}$TP** was prepared from **dU$^{\mathbf{APh}}$** using Method C. ^{1}H NMR (500.0 MHz, D_2O): 2.08 (dt, 1H, J_{gem} = 14.2, $J_{2'b,1'}$ = $J_{2'b,3'}$ = 6.9, H-2'b); 2.25 (ddd, 1H, J_{gem} = 14.2, $J_{2'a,1'}$ = 6.5, $J_{2'a,3'}$ = 4.2, H-2'a); 2.66 – 2.77 (m, 2H, Ph-CH_2C**H$_2$**-B); 2.80 - 2.93 (m, 2H, Ph-C**H$_2$**CH_2-B); 4.02 – 4.17 (m, 3H, H-4´, 5'); 4.44 (ddd, 1H, $J_{3',2'}$ = 6.5, 4.2, $J_{3',4'}$ = 3.4, H-3'); 6.22 (dd, 1H, $J_{1',2'}$ = 6.9, 6.5, H-1'); 7.22 – 7.33 (m, 4H, H-6, H-*o*,*p*-Ph); 7.38 (m, 2H, H-*m*-Ph).

^{13}C NMR (125.7 MHz, D_2O): 30.42 (Ph-**C**H_2CH_2-B); 36.31 (Ph-CH_2**C**H_2-B); 41.08 (CH_2-2'); 67.88 (d, $J_{C,P}$ = 5.5, CH_2-5'); 72.93 (CH-3'); 87.24 (CH-1'); 87.96 (d, $J_{C,P}$ = 8.9, CH-4'); 116.77 (C-5); 128.95 (CH-*p*-Ph); 131.43 (CH-*m*-Ph); 131.79 (CH-*o*-Ph); 141.14 (CH-6); 143.77 (C-*i*-Ph); 154.21 (C-2); 168.67 (C-4).

^{31}P NMR (202.4 MHz, D_2O): -21.70 (m, 1P, P_β); -10.83 (d, 1P, $J_{\alpha,\beta}$ = 19.8, P_α); -7.23 (m, 1P, P_γ).

MS (ESI): *m/z*: 411.1 [M-2PO_3Na-Na]$^-$; 491.1 [M-PO_3Na-2Na+H]$^-$; 513.0 [M-PO_3Na-Na]$^-$; 571.0 [M-3Na+2H]$^-$; 593.0 [M-2Na+H]$^-$; 615.0 [M-Na]$^-$; 637.0 [M-H]$^-$.
HRMS (ESI): *m/z*: calcd for $C_{17}H_{22}O_{14}N_2P_3$ [M-3Na+2H]$^-$: 571.02894; found: 571.02895.

7-(2-(1H-Indol-3-yl)-1-ethyn-1-yl)-2´-deoxyadenosine **(dA$^{\mathbf{EIn}}$)**

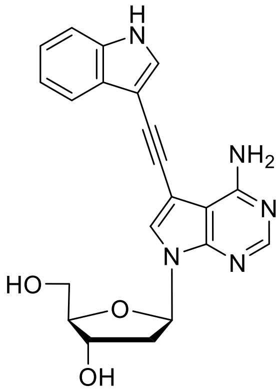

dA$^{\mathbf{EIn}}$

Compound **dA$^{\mathbf{EIn}}$** was prepared from **dA$^{\mathbf{I}}$** using Method A. ^{1}H NMR (500.0 MHz, DMSO-d_6): 2.22 (ddd, 1H, J_{gem} = 13.1, $J_{2'b,1'}$ = 6.0, $J_{2'b,3'}$ = 2.8, H-2'b); 2.52 (ddd, 1H, J_{gem} = 13.1, $J_{2'a,1'}$ = 8.1, $J_{2'a,3'}$ = 5.8, H-2'b); 3.53, 3.60 (2 × dd, 2 × 1H, J_{gem} = 11.7, $J_{5',4'}$ = 4.4, H-5'); 3.85 (td, 1H, $J_{4',5'}$ = 4.4, $J_{4',3'}$ = 2.5, H-4'); 4.37 (ddd, 1H, $J_{3',2'}$ = 5.8, 2.8, $J_{3',4'}$ = 2.5 , H-3'); 4.97 – 5.32 (bm, 2H, OH-3',5'); 6.52 (dd, 1H, $J_{1',2'}$ = 8.1, 6.0, H-1'); 6.90 (bs, 2H, NH_2); 7.13 (ddd, 1H, $J_{5,4}$ = 8.0, $J_{5,6}$ = 7.0, $J_{5,7}$ = 1.1, H-5-ind); 7.20 (ddd, 1H, $J_{6,7}$ = 8.2, $J_{6,5}$ = 7.0, $J_{6,4}$ = 1.3, H-6-ind); 7.46 (dt, 1H, $J_{7,6}$ = 8.1, $J_{7,4}$ = $J_{7,5}$ = 1.1, H-7-ind); 7.61 (ddt, 1H, $J_{4,5}$ = 8.0, $J_{4,6}$ =

1.3, $J_{4,1}$ = $J_{4,7}$ = 1.1, H-4-ind); 7.81 (d, 1H, $J_{2,1}$ = 2.7, H-2-ind); 7.85 (s, 1H, H-6); 8.17 (s, 1H, H-2); 11.57 (bd, 1H, $J_{1,2}$ = 2.7, NH-1-ind).

^{13}C NMR (125.7 MHz, DMSO-d_6): 39.87 (CH_2-2'); 62.11 (CH_2-5'); 71.18 (CH-3'); 83.33 (CH-1'); 83.63 (deazaA-**C**≡C-ind); 85.97 (deazaA-C≡**C**-ind); 87.71 (CH-4'); 96.07, 96.08 (C-3-ind, C-5); 102.46 (C-4a); 112.41 (CH-7-ind); 118.90 (CH-4-ind); 120.29 (CH-5-ind); 122.51 (CH-6-ind); 125.60 (CH-6); 128.19 (C-3a-ind); 129.96 (CH-2-ind); 135.57 (C-7a-ind); 149.41 (C-7a); 152.89 (CH-2); 157.90 (C-4).

MS (ESI): *m/z*: 390.2 [M+H]; 412.2 [M+Na].

HRMS (ESI): *m/z*: [M+H] calcd for $C_{21}H_{20}O_3N_5$: 390.15607; found: 390.15615. [M+Na] calcd for: $C_{21}H_{19}O_3N_5Na$: 412.13801; found: 412.13804.

m.p.: 143-145°C.

7-(2-(1H-Indol-3-yl)-ethyl)-2´-deoxyadenosine (dA^{AIn})

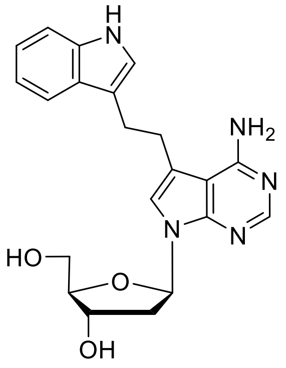

dA^{AIn}

Compound **dA^{AIn}** was prepared from **dA^{EIn}** using Method B1. ^{1}H NMR (500.0 MHz, DMSO-d_6): 2.09 (ddd, 1H, J_{gem} = 13.0, $J_{2'b,1'}$ = 5.9, $J_{2'b,3'}$ = 2.5, H-2'b); 2.46 (ddd, 1H, J_{gem} = 13.0, $J_{2'a,1'}$ = 8.5, $J_{2'a,3'}$ = 5.8, H-2'b); 2.95 – 3.06 (m, 2H, ind-C**H$_2$**CH$_2$-deazaA); 3.08 – 3.19 (m, 2H, ind-CH$_2$C**H$_2$**-deazaA); 3.48 (ddd, 1H, J_{gem} = 11.6, $J_{5'b,OH}$ = 6.1, $J_{5'b,4'}$ = 4.4, H-5'b); 3.55 (ddd, 1H, J_{gem} = 11.6, $J_{5'a,OH}$ = 5.2, $J_{5'a,4'}$ = 4.8, H-5'b); 3.79 (ddd, 1H, $J_{4',5'}$ = 4.8, 4.4, $J_{4',3'}$ = 2.5, H-4'); 4.32 (ddt, 1H, $J_{3',2'}$ = 5.8, 2.5, $J_{3',OH}$ = 4.1, $J_{3',4'}$ = 2.5 , H-3'); 5.10 (dd, 1H, $J_{OH,5'}$ = 6.1, 5.2, OH-5'); 5.22 (d, 1H, $J_{OH,3'}$ = 4.1, OH-3'); 6.48 (dd, 1H, $J_{1',2'}$ = 8.5, 5.9, H-1'); 6.55 (bs, 2H, NH_2); 6.96 (ddd, 1H, $J_{5,4}$ = 8.0, $J_{5,6}$ = 7.0, $J_{5,7}$ = 1.1, H-5-ind); 7.05 (ddd, 1H, $J_{6,7}$ = 8.1, $J_{6,5}$ = 7.0, $J_{6,4}$ = 1.1, H-6-ind); 7.85 (t, 1H, $J_{6,CH2}$ = 1.1, H-6); 7.81 (dt, 1H, $J_{2,1}$ = 2.4, $J_{2,CH2}$ = 1.1, H-2-ind); 7.33 (dt, 1H, $J_{7,6}$ = 8.1, $J_{7,4}$ = $J_{7,5}$ = 1.1, H-7-ind); 7.52 (ddt, 1H, $J_{4,5}$ = 8.0, $J_{4,1}$ = $J_{4,6}$ = $J_{4,7}$ = 1.1, H-4-ind); 8.02 (s, 1H, H-2); 10.78 (bd, 1H, $J_{1,2}$ = 2.4, NH-1-ind).

^{13}C NMR (125.7 MHz, DMSO-d_6): 25.62 (ind-**C**H$_2$CH$_2$-deazaA); 26.85 (ind-CH$_2$**C**H$_2$-deazaA); 39.70 (CH_2-2'); 62.34 (CH_2-5'); 71.31 (CH-3'); 82.97 (CH-1'); 87.30 (CH-4'); 102.48 (C-4a); 111.44 (CH-7-ind); 114.35 (C-3-ind); 115.52 (C-5); 118.25 (CH-5-ind);

118.52 (CH-4-ind); 118.80 (CH-6); 120.97 (CH-6-ind); 122.70 (CH-2-ind); 127.42 (C-3a-ind); 136.35 (C-7a-ind); 150.51 (C-7a); 151.45 (CH-2); 157.94 (C-4).

MS (ESI): *m/z*: 394.2 [M+H]; 416.2 [M+Na].

HRMS (ESI): *m/z*: [M+H] calcd for $C_{21}H_{24}O_3N_5$: 394.18737; found: 394.18738. [M+Na] calcd for: $C_{21}H_{23}O_3N_5Na$: 416.16931; found: 416.16934.

m.p.: 179-181°C.

***7-(2-(1H-Indol-3-yl)-1-ethyn-1-yl)-2´-deoxyadenosine triphosphate* (dA^{EIn}TP)**

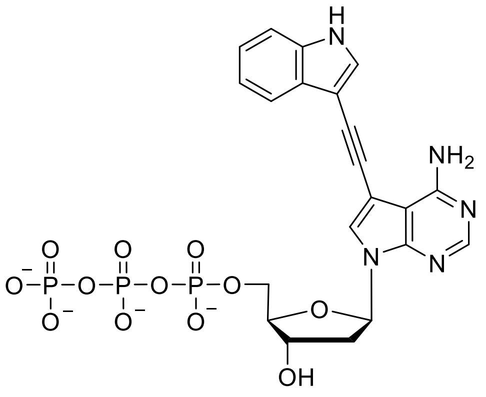

dA^{EIn}TP

Compound **dA^{EIn}TP** was prepared from **dA^{EIn}** using Method C. ^{1}H NMR (500.0 MHz, D_2O): 2.40 (ddd, 1H, J_{gem} = 14.0, $J_{2'b,1'}$ = 6.2, $J_{2'b,3'}$ = 3.4, H-2'b); 2.59 (ddd, 1H, J_{gem} = 14.1, $J_{2'a,1'}$ = 7.9, $J_{2'a,3'}$ = 6.4, H-2'a); 4.07 – 4.19 (m, 2H, H-5'); 4.21 (m, 1H, H-4'); 4.71 (dt, 1H, $J_{3',2'}$ = 6.4, 3.4, $J_{3',4'}$ = 3.4, H-3'); 6.37 (bdd, 1H, $J_{1',2'}$ = 7.9, 6.2, H-1'); 7.14 (ddd, 1H, $J_{5,4}$ = 8.0, $J_{5,6}$ = 7.1, $J_{5,7}$ = 1.1, H-5-ind); 7.21 (ddd, 1H, $J_{6,7}$ = 8.2, $J_{6,5}$ = 7.1, $J_{6,4}$ = 1.2, H-6-ind); 7.41 (bd, 1H, $J_{7,6}$ = 8.2, H-7-ind); 7.51 – 7.54 (m, 2H, H-2-ind, H-6); 7.60 (bd, 1H, $J_{4,5}$ = 8.0, H-4-ind); 7.93 (bs, 1H, H-2).

^{13}C NMR (125.7 MHz, D_2O): 40.91 (CH_2-2'); 68.19 (d, $J_{C,P}$ = 5.8, CH_2-5'); 73.67 (CH-3'); 85.46 (CH-1'); 85.80 (ind-C≡**C**-deazaA); 87.85 (d, $J_{C,P}$ = 8.9, CH-4'); 89.28 (ind-**C**≡C-deazaA); 98.34 (C-3-ind); 100.37 (C-5); 105.70 (C-4a); 114.70 (CH-7-ind); 121.50 (CH-5-ind); 123.27 (CH-4-ind); 125.41 (CH-6-ind); 127.08 (CH-6); 130.16 (C-3a-ind); 132.51 (CH-2-ind); 137.71 (C-7a-ind); 150.94 (C-7a); 154.41 (CH-2); 160.09 (C-4).

^{31}P NMR (202.4 MHz, D_2O): -21.40 (t, 1P, $J_{\beta,\alpha} = J_{\beta,\gamma}$= 19.9, P_β); -10.27 (d, 1P, $J_{\alpha,\beta}$ = 19.5, P_α); -5.97 (d, 1P, $J_{\gamma,\beta}$ = 20.4, P_γ).

MS (ESI): *m/z*: 313.5 [(M-3Na+H)/2]$^{2-}$; 468.1 [M-2PO$_3$Na-Na]$^-$; 548.1 [M-PO$_3$Na-2Na+H]$^-$; 570.1 [M-PO$_3$Na-Na]$^-$; 628.0 [M-3Na+2H]$^-$; 650.0 [M-2Na+H]$^-$; 672.0 [M-Na]$^-$.

HRMS (ESI): *m/z*: calcd for $C_{21}H_{21}O_{12}N_5P_3$ [M-3Na+2H]$^-$: 628.04050; found: 628.04095. Calcd for $C_{21}H_{20}O_{12}N_5P_3Na$ [M-2Na+H]$^-$: 650.02245; found: 650.02277.

7-(2-(1H-Indol-3-yl)-ethyl)-2´-deoxyadenosine triphosphate **(dA^{AIn}TP)**

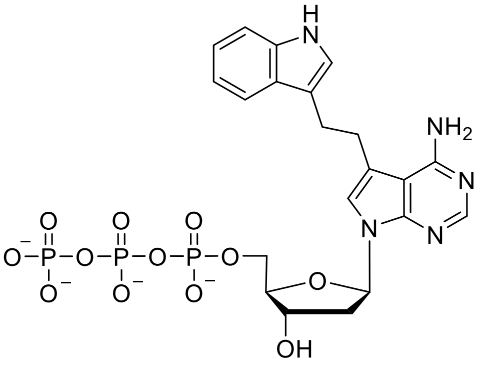

dA^{AIn}TP

Compound **dA^{AIn}TP** was prepared from **dA^{AIn}** using Method C. ^{1}H NMR (500.0 MHz, D_2O): 2.18 – 2.28 (m, 2H, H-2'); 2.96 – 3.10 (m, 4H, ind-C**H$_2$**C**H$_2$**-deazaA); 3.96 -4.04 (m, 2H, H-5'); 4.13 (btd, 1H, $J_{4',5'}$ = 4.5, $J_{4',3'}$ = 3.6, H-4'); 4.45 (btd, 1H, $J_{3',2'}$ = 5.0, $J_{3',4'}$ = 3.6, H-3'); 6.41 (t, 1H, $J_{1',2'}$ = 6.8, H-1'); 6.87 (s, 1H, H-6); 6.90 (bdd, 1H, $J_{5,4}$ = 8.0, $J_{5,6}$ = 7.5, H-5-ind); 7.09 (s, 1H, H-2-ind); 7.11 (bdd, 1H, $J_{6,7}$ = 8.2, $J_{6,5}$ = 7.5, H-6-ind); 7.17 (bd, 1H, $J_{4,5}$ = 8.0, H-4-ind); 7.43 (bd, 1H, $J_{7,6}$ = 8.2, H-4-ind); 7.96 (s, 1H, H-2).

^{13}C NMR (125.7 MHz, D_2O): 28.11 (ind-**C**H$_2$CH$_2$-deazaA); 29.81 (ind-CH$_2$**C**H$_2$-deazaA); 40.69 (CH$_2$-2'); 68.29 (d, $J_{C,P}$ = 5.4, CH$_2$-5'); 73.62 (CH-3'); 85.14 (CH-1'); 87.42 (d, $J_{C,P}$ = 8.5, CH-4'); 105.62 (C-4a); 114.35 (CH-7-ind); 116.59 (C-3-ind); 119.31 (C-5); 120.95 (CH-4-ind); 121.30 (CH-5-ind); 122.41 (CH-6); 124.16 (CH-6-ind); 126.08 (CH-2-ind); 129.89 (C-3a-ind); 138.72 (C-7a-ind); 151.83 (C-7a); 152.60 (CH-2); 159.47 (C-4).

^{31}P NMR (202.4 MHz, D_2O): -21.08 (m, 1P, P_β); -10.29 (d, 1P, $J_{\alpha,\beta}$ = 19.2, P_α); -6.43 (m, 1P, P_γ).

MS (ESI): *m/z*: 326.5 [(M-2Na)/2]$^{2-}$; 472.1 [M-2PO$_3$Na-Na]$^-$; 552.1 [M-PO$_3$Na-2Na+H]$^-$; 574.1 [M-PO$_3$Na-Na]$^-$.

HRMS (ESI): *m/z*: calcd for $C_{21}H_{25}O_{12}N_5P_3$ [M-3Na+2H]$^-$: 632.07180; found: 632.07125.

5-(Pent-1-yn-1-yl)-2´-deoxycytidine **(dC$^{\mathbf{EAlk}}$)**

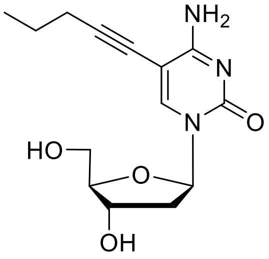

dC$^{\mathbf{EAlk}}$

Compound **dC$^{\mathbf{EAlk}}$** was prepared from **dC$^{\mathbf{I}}$** using Method A. The spectral data were in accordance with literature.[3] ^{1}H NMR (500.0 MHz, DMSO-*d*$_6$): 0.96 (t, 3H, J_{vic} = 7.4, C**H$_3$**CH$_2$CH$_2$); 1.54 (qt, 2H, J_{vic} = 7.4, 7.1, CH$_3$C**H$_2$**CH$_2$); 1.97 (ddd, 1H, J_{gem} = 13.2, $J_{2'b,1'}$ = 7.3, $J_{2'b,3'}$ = 6.0, H-2'b); 2.12 (ddd, 1H, J_{gem} = 13.2, $J_{2'a,1'}$ = 6.0, $J_{2'a,3'}$ = 3.5, H-2'a); 2.37 (t, 2H, J_{vic} = 7.1, CH$_3$CH$_2$C**H$_2$**); 3.54, 3.60 (2 × bd, 2 × 1H, J_{gem} = 11.7, H-5'); 3.77 (q, 1H, $J_{4',3'}$ = $J_{4',5'}$ = 3.5, H-4'); 4.20 (dt, 1H, $J_{3',2'}$ = 6.0, 3.5, $J_{3',4'}$ = 3.5 , H-3'); 5.05 (bs, 1H, OH-5'); 5.20 (bs, 1H, OH-3'); 6.11 (dd, 1H, $J_{1',2'}$ = 7.3, 6.0, H-1'); 6.69, 7.67 (2 × bs, 2 × 1H, NH$_2$); 8.07 (s, 1H, H-6).

^{13}C NMR (125.7 MHz, DMSO-*d*$_6$): 13.69 (**C**H$_3$CH$_2$CH$_2$); 21.21 (CH$_3$CH$_2$**C**H$_2$); 21.72 (CH$_3$**C**H$_2$CH$_2$); 40.92 (CH$_2$-2'); 61.21 (CH$_2$-5'); 70.32 (CH-3'); 72.35 (C5-**C**≡C-nPr); 85.39 (CH-1'); 87.56 (CH-4'); 90.59 (C-5); 95.70 (C5-C≡**C**-*n*Pr); 143.68 (CH-6); 153.69 (C-2); 164.57 (C-4).

MS (ESI): *m/z*: 294.1 [M+H]; 316.1 [M+Na].

HRMS (ESI): *m/z*: [M+H] calcd for $C_{14}H_{20}O_4N_3$: 294.14483; found: 294.14491.

m.p.: 148-150°C.

5-(Pentyl)-2´-deoxycytidine **(dC$^{\mathbf{AAlk}}$)**

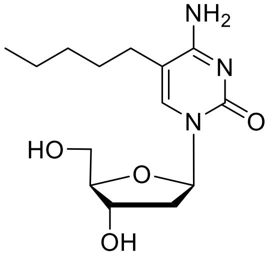

dC$^{\mathbf{AAlk}}$

Compound **dC$^{\mathbf{AAlk}}$** was prepared from **dC$^{\mathbf{EAlk}}$** using Method B1. ^{1}H NMR (500.0 MHz, DMSO-*d*$_6$): 0.86 (t, 3H, J_{vic} = 7.0, C**H$_3$**CH$_2$CH$_2$CH$_2$CH$_2$); 1.21 – 1.35 (m, 4H, CH$_3$C**H$_2$**C**H$_2$**CH$_2$CH$_2$); 1.37 – 1.44 (m, 4H, CH$_3$CH$_2$CH$_2$C**H$_2$**CH$_2$); 1.96 (ddd, 1H, J_{gem} = 13.2, $J_{2'b,1'}$ = 7.4, $J_{2'b,3'}$ = 6.0, H-2'b); 2.08 (ddd, 1H, J_{gem} = 13.2, $J_{2'a,1'}$ = 6.1, $J_{2'a,3'}$ = 3.4, H-2'a); 2.15 – 2.28 (m, 2H, CH$_3$CH$_2$CH$_2$CH$_2$C**H$_2$**); 3.53, 3.58 (2 × ddd, 2 × 1H, J_{gem} = 11.8, $J_{5',OH}$ = 5.2, $J_{5',4'}$ = 3.8, H-5'); 3.75 (q, 1H, $J_{4',3'}$ = $J_{4',5'}$ = 3.8, H-4'); 4.21 (dddd, 1H, $J_{3',2'}$ = 6.0, 3.4, $J_{3',OH}$ = 4.2, $J_{3',4'}$ = 3.8 , H-3'); 4.99 (t, 1H, $J_{OH,5'}$ = 5.2, OH-5'); 5.20 (d, 1H, $J_{OH,3'}$ = 4.2, OH-3'); 6.17 (dd, 1H, $J_{1',2'}$ = 7.4, 6.1, H-1'); 6.80, 7.22 (2 × bs, 2 × 1H, NH$_2$); 7.61 (s, 1H, H-6).

^{13}C NMR (125.7 MHz, DMSO-d_6): 14.18 (**C**$H_3CH_2CH_2CH_2CH_2$); 22.12 (CH_3**C**$H_2CH_2CH_2CH_2$); 26.72 ($CH_3CH_2CH_2CH_2$**C**H_2); 27.68 ($CH_3CH_2CH_2$**C**H_2CH_2); 30.88 (CH_3CH_2**C**$H_2CH_2CH_2$); 40.52 (CH_2-2'); 61.52 (CH_2-5'); 70.59 (CH-3'); 84.95 (CH-1'); 87.30 (CH-4'); 105.63 (C-5); 138.24 (CH-6); 155.09 (C-2); 164.91 (C-4).

MS (ESI): *m/z*: 298.2 [M+H]; 320.2 [M+Na].

HRMS (ESI): *m/z*: [M+H] calcd for $C_{14}H_{24}O_4N_3$: 298.17613; found: 298.17620. [M+Na] calcd for: $C_{14}H_{23}O_4N_3Na$: 320.15808; found: 320.15810.

m.p.: 170-172°C.

5-(Pent-1-yn-1-yl)-2´-deoxycytidine triphosphate **(dC^{EAlk}TP)**

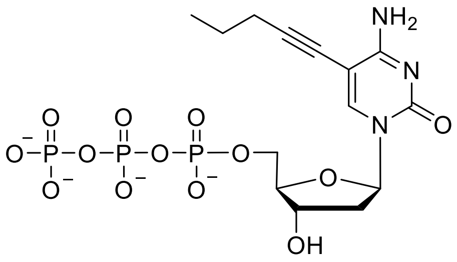

dC^{EAlk}TP

Compound **dC^{EAlk}TP** was prepared from **dC^{EAlk}** using Method C. ^{1}H NMR (500.0 MHz, D_2O): 1.00 (t, 3H, J = 7.4, C**H$_3$**CH_2CH_2); 1.61 (sextet, 2H, J = 7.3, CH_3C**H$_2$**CH_2); 2.31 (dt, 1H, J_{gem} = 14.1, $J_{2'b,1'}$ = $J_{2'b,3'}$ = 6.8, H-2'b); 2.43 (t, 2H, J = 7.2, CH_3CH_2C**H$_2$**); 2.43 (ddd, 1H, J_{gem} = 14.1, $J_{2'a,1'}$ = 6.3, $J_{2'a,3'}$ = 4.0, H-2'a); 4.13 – 4.28 (m, 3H, H-4´,5'); 4.62 (bdt, 1H, $J_{3',2'}$ = 6.8, 4.0, $J_{3',4'}$ = 4.0, H-3'); 6.28 (dd, 1H, $J_{1',2'}$ = 6.8, 6.3, H-1'); 8.03 (s, 1H, H-6).

^{13}C NMR (125.7 MHz, D_2O): 15.71 (**C**$H_3CH_2CH_2$); 23.57 (CH_3CH_2**C**H_2); 24.20 (CH_3**C**H_2CH_2); 41.83 (CH_2-2'); 67.93 (d, $J_{C,P}$ = 5.6, CH_2-5'); 73.06 (CH-3'); 73.08 (CH_2C≡**C**-B); 88.15 (d, $J_{C,P}$ = 8.8, CH-4'); 88.84 (CH-1'); 96.34 (C-5); 101.14 (CH_2**C**≡C-B); 146.22 (CH-6); 158.88 (C-2); 168.05 (C-4).

^{31}P NMR (202.4 MHz, D_2O): -21.66 (t, 1P, $J_{\beta,\alpha} = J_{\beta,\gamma}$= 19.8, P_β); -10.44 (d, 1P, $J_{\alpha,\beta}$ = 19.8, P_α); -7.56 (m, 1P, P_γ).

MS (ESI): *m/z*: 452.1 [M-PO_3Na-2Na+H]$^-$; 474.0 [M-PO_3Na-Na]$^-$; 532.0 [M-3Na+2H]$^-$; 554.0 [M-2Na+H]$^-$; 576.0 [M-Na]$^-$.

HRMS (ESI): *m/z*: calcd for $C_{14}H_{21}O_{13}N_3P_3$ [M-3Na+2H]$^-$: 532.02927; found: 532.02832.

5-(Pentyl)-2´-deoxycytidine triphosphate (dC^{AAlk}TP)

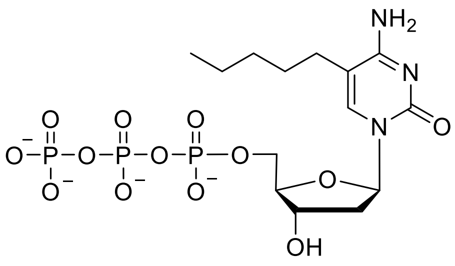

dC^{AAlk}TP

Compound **dC^{AAlk}TP** was prepared from **dC^{AAlk}** using Method C or from **dC^{EAlk}TP** using method B2. ^{1}H NMR (500.0 MHz, D_2O): 0.87 (t, 3H, *J* = 7.0, C**H**$_3$(CH$_2$)$_4$); 1.24 – 1.36 (m, 4H, CH$_3$C**H$_2$**C**H$_2$**(CH$_2$)$_2$); 1.53 (pent, 2H, *J* = 7.4, CH$_3$(CH$_2$)$_2$C**H$_2$**CH$_2$); 2.29 – 2.42 (m, 2H, H-2'); 2.41 (t, 2H, *J* = 7.4, CH$_3$(CH$_2$)$_3$C**H$_2$**); 4.15 – 4.28 (m, 3H, H-4',5'); 4.66 (bdt, 1H, $J_{3',2'}$ = 6.1, 3.3, $J_{3',4'}$ = 3.3, H-3'); 6.35 (t, 1H, $J_{1',2'}$ = 6.9, H-1'); 7.70 (s, 1H, H-6).

^{13}C NMR (125.7 MHz, D_2O): 16.13 (**C**H$_3$CH$_2$CH$_2$); 24.64 (CH$_3$CH$_2$**C**H$_2$(CH$_2$)$_2$); 29.34 and 29.66 (CH$_3$(CH$_2$)$_2$**C**H$_2$**C**H$_2$);, 33.17 (CH$_3$**C**H$_2$(CH$_2$)$_3$); 41.75 (CH$_2$-2'); 68.06 (d, $J_{C,P}$ = 5.5, CH$_2$-5'); 73.41 (CH-3'); 88.18 (d, $J_{C,P}$ = 9.0, CH-4'); 88.39 (CH-1'); 111.97 (C-5); 141.35 (CH-6); 160.01 (C-2); 168.16 (C-4).

^{31}P NMR (202.4 MHz, D_2O): -21.61 (t, 1P, $J_{\beta,\alpha} = J_{\beta,\gamma}$ = 19.7, P$_\beta$); -10.76 (d, 1P, $J_{\alpha,\beta}$ = 19.7, P$_\alpha$); -7.10 (d, 1P, $J_{\gamma,\beta}$ = 19.7, P$_\gamma$).

MS (ESI): *m/z*: 376.1 [M-2PO$_3$Na-Na]$^-$; 456.1 [M-PO$_3$Na-2Na+H]$^-$; 478.1 [M-PO$_3$Na-Na]$^-$; 536.1 [M-3Na+2H]$^-$; 558.0 [M-2Na+H]$^-$; 580.0 [M-Na]$^-$.

HRMS (ESI): *m/z*: calcd for $C_{14}H_{25}O_{13}N_3P_3$ [M-3Na+2H]$^-$: 536.06057; found: 536.06004.

7-(3-methylbut-1-yn-1-yl)-2´-deoxyguanosine (dG^{EiPr})

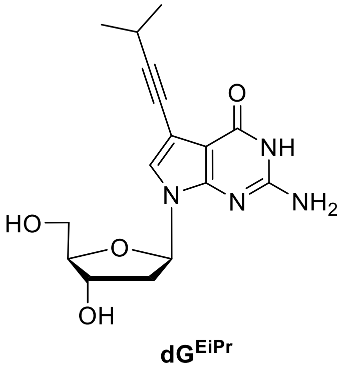

dG^{EiPr}

Compound **dG^{EiPr}** was prepared from **dG^{I}** using Method A. ^{1}H NMR (500.0 MHz, DMSO-*d*$_6$): 1.17 (d, 6H, J_{vic} = 6.9, (C**H$_3$**)$_2$CH); 2.05 (ddd, 1H, J_{gem} = 13.0, $J_{2'b,1'}$ = 5.8, $J_{2'b,3'}$ = 2.4, H-2'b); 2.30 (ddd, 1H, J_{gem} = 13.0, $J_{2'a,1'}$ = 8.5, $J_{2'a,3'}$ = 5.6, H-2'b); 2.73 (sep, 1H, J_{vic} = 6.9, C**H**(CH$_3$)$_2$); 3.46 (ddd, 1H, J_{gem} = 11.7, $J_{5'b,OH}$ = 5.5, $J_{5'b,4'}$ = 4.5, H-5'); 3.50 (ddd, 1H, J_{gem} = 11.7, $J_{5'a,OH}$ = 5.5, $J_{5'a,4'}$ = 5.0, H-5'); 3.74 (ddd, 1H, $J_{4',5'}$ = 5.0, 4.5, $J_{4',3'}$ = 2.4, H-4'); 4.26 (ddt, 1H, $J_{3',2'}$ = 5.6, 2.4, $J_{3',OH}$ = 3.7, $J_{3',4'}$ = 2.4 , H-3'); 4.90 (t, 1H,

$J_{OH,5'}$ = 5.5, OH-5'); 5.20 (d, 1H, $J_{OH,3'}$ = 3.7, OH-3'); 6.26 (d, 1H, $J_{1',2'}$ = 8.5, 5.8, H-1'); 6.29 (bs, 2H, NH_2); 7.11 (s, 1H, H-6); 10.38 (s, 1H, NH).
^{13}C NMR (125.7 MHz, DMSO-d_6): 20.85 (**C**H(CH_3)$_2$); 23.24 ((**C**H_3)$_2$CH); 39.75 (CH_2-2'); 62.11 (CH_2-5'); 70.16 (CH-3'); 73.98 (C5-**C**≡C-*i*Pr); 82.35 (CH-1'); 87.28 (CH-4'); 95.33 (C5-C≡**C**-*i*Pr); 99.58 (C-4a,5); 121.11 (CH-6); 150.33 (C-7a); 153.23 (C-2); 158.05 (C-4).
MS (ESI): *m/z*: 331.2 [M-H]; 333.2 [M+H]; 355.2 [M+Na].
HRMS (ESI): *m/z*: [M+H] calcd for $C_{16}H_{21}O_4N_4$: 333.15573; found: 333.15576. [M+Na] calcd for: $C_{16}H_{20}O_4N_4Na$: 355.13768; found: 355.13770.
m.p.: 126-128°C.

7-(isopentyl)-2´-deoxyguanosine (dG^{AiPr})

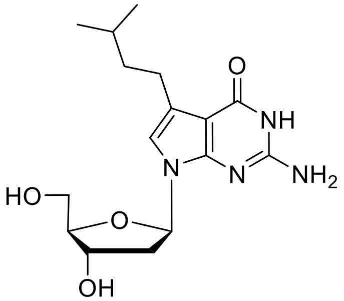

dG^{AiPr}

Compound **dG^{AiPr}** was prepared from **dG^{EiPr}** using Method B1.
^{1}H NMR (500.0 MHz, DMSO-d_6): 0.89 (d, 6H, J_{vic} = 6.6, (C**H$_3$**)$_2$CHCH_2CH_2); 1.42 – 1.50 (m, 2H, (CH_3)$_2$CHC**H$_2$**CH_2); 1.54 (non, 1H, J_{vic} = 6.6, (CH_3)$_2$C**H**CH_2CH_2); 2.01 (ddd, 1H, J_{gem} = 13.0, $J_{2'b,1'}$ = 5.8, $J_{2'b,3'}$ = 2.4, H-2'b); 2.29 (ddd, 1H, J_{gem} = 13.0, $J_{2'a,1'}$ = 8.7, $J_{2'a,3'}$ = 5.7, H-2'b); 2.52 – 2.57 (m, 2H, (CH_3)$_2$CHCH_2C**H$_2$**); 3.41 – 3.51 (m, 2H, H-5'); 3.71 (td, 1H, $J_{4',5'}$ = 4.9, $J_{4',3'}$ = 2.4, H-4'); 4.25 (ddt, 1H, $J_{3',2'}$ = 5.7, 2.4, $J_{3',OH}$ = 3.8, $J_{3',4'}$ = 2.4 , H-3'); 4.85 (t, 1H, $J_{OH,5'}$ = 5.5, OH-5'); 5.17 (d, 1H, $J_{OH,3'}$ = 3.8, OH-3'); 6.16 (bs, 2H, NH_2); 6.26 (d, 1H, $J_{1',2'}$ = 8.7, 5.8, H-1'); 6.61 (t, 1H, ^4J = 1.1, H-6); 10.20 (s, 1H, NH).
^{13}C NMR (125.7 MHz, DMSO-d_6): 22.71, 22.72 ((**C**H_3)$_2$CHCH_2CH_2); 24.04 ((CH_3)$_2$CHCH_2**C**H_2); 27.53 ((CH_3)$_2$**C**HCH_2CH_2); 39.29 ((CH_3)$_2$CH**C**H_2CH_2); 39.45 (CH_2-2'); 62.29 (CH_2-5'); 71.19 (CH-3'); 82.00 (CH-1'); 86.92 (CH-4'); 99.58 (C-4a); 112.96 (CH-6); 119.79 (C-5); 150.86 (C-7a); 152.57 (C-2); 159.08 (C-4).
MS (ESI): *m/z*: 337.2 [M+H]; 359.2 [M+Na].
HRMS (ESI): *m/z*: [M+H] calcd for $C_{16}H_{25}O_4N_4$: 337.18703; found: 337.18712. [M+Na] calcd for: $C_{16}H_{24}O_4N_4Na$: 359.16898; found: 359.16907.
m.p.: 140-145°C.

7-(3-methylbut-1-yn-1-yl)-2´-deoxyguanosine triphosphate (**dG^{EiPr}TP**)

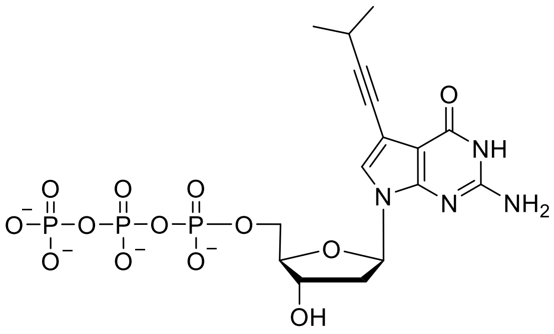

dG^{EiPr}TP

Compound **dG^{EiPr}TP** was prepared from **dG^{EiPr}** using Method C. ^{1}H NMR (500.0 MHz, D_2O): 1.24 (d, 6H, J_{vic} = 6.9, (C**H$_3$**)$_2$CH); 2.39 (ddd, 1H, J_{gem} = 14.0, $J_{2'b,1'}$ = 6.3, $J_{2'b,3'}$ = 3.3, H-2'b); 2.64 (ddd, 1H, J_{gem} = 14.0, $J_{2'a,1'}$ = 7.9, $J_{2'a,3'}$ = 6.4, H-2'a); 2.82 (septet, 1H, J_{vic} = 6.9, $(CH_3)_2$C**H**); 4.09 -4.24 (m, 3H, H-5', 4'); 4.74 (dt, 1H, $J_{3',2'}$ = 6.4, 3.3, $J_{3',4'}$ = 3.3, H-3'); 6.40 (dd, 1H, $J_{1',2'}$ = 7.9, 6.3, H-1'); 7.23 (s, 1H, H-6).

^{13}C NMR (125.7 MHz, D_2O): 23.57 and 24.83 ((**C**H_3)$_2$CH); 40.87 (CH_2-2'); 68.17 (d, $J_{C,P}$ = 5.5, CH_2-5'); 73.70 (CH-3'); 75.00 (CHC≡**C**); 85.63 (CH-1'); 87.86 (d, $J_{C,P}$ = 8.7, CH-4'); 101.36 (CH**C**≡C); 102.33 and 102.93 (C-4a,5); 125.09 (CH-6); 153.49 (C-7a); 156.30 (C-2); 164.13 (C-4).

^{31}P NMR (202.4 MHz, D_2O): -21.23 (t, 1P, $J_{\beta,\alpha} = J_{\beta,\gamma}$ = 19.9, P_β); -10.19 (d, 1P, $J_{\alpha,\beta}$ = 19.5, P_α); -5.27 (d, 1P, $J_{\gamma,\beta}$ = 20.3, P_γ).

MS (ESI): *m/z*: 411.1 [M-2PO_3Na-Na]$^-$; 491.1 [M-PO_3Na-2Na+H]$^-$; 513.1 [M-PO_3Na-Na]$^-$; 571.0 [M-3Na+2H]$^-$; 593.0 [M-2Na+H]$^-$; 615.0 [M-Na]$^-$.
HRMS (ESI): *m/z*: calcd for $C_{16}H_{22}O_{13}N_4P_3$ [M-3Na+2H]$^-$: 571.04017; found: 571.04032. Calcd for $C_{16}H_{21}O_{13}N_4P_3Na$ [M-2Na+H]$^-$: 593.02211; found: 593.02197.

7-(isopentyl)-2´-deoxyguanosine triphosphate (**dG^{AiPr}TP**)

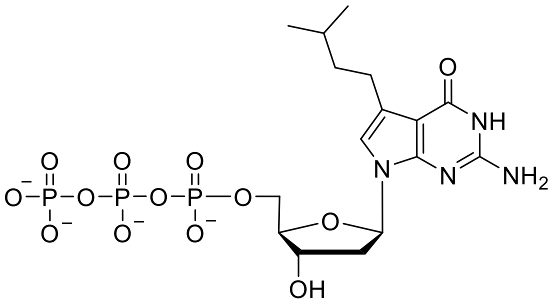

dG^{AiPr}TP

Compound **dG^{AiPr}TP** was prepared from **dG^{AiPr}** using Method B1. ^{1}H NMR (500.0 MHz, D_2O): 0.90 and 0.91 (2×d, 2×3H, J_{vic} = 6.5, (C**H$_3$**)$_2$CHCH_2CH_2); 1.46 – 1.62 (m, 3H, $(CH_3)_2$C**H**C**H$_2$**CH_2); 2.34 (ddd, 1H, J_{gem} = 14.0, $J_{2'b,1'}$ = 6.2, $J_{2'b,3'}$ = 3.2, H-2'b); 2.64 (ddd, 1H, J_{gem} = 14.0, $J_{2'a,1'}$ = 8.3, $J_{2'a,3'}$ = 6.3, H-2'a); 2.63

– 2.70 (m, 2H, $(CH_3)_2CHCH_2C\mathbf{H_2}$); 4.06 -4.22 (m, 2H, H-5'); 4.19 (m, 1H, H-4'); 4.72 (dt, 1H, $J_{3',2'}$ = 6.3, 3.1, $J_{3',4'}$ = 3.1, H-3'); 6.41 (dd, 1H, $J_{1',2'}$ = 8.3, 6.1, H-1'); 6.86 (s, 1H, H-6). ^{13}C NMR (125.7 MHz, D_2O): 24.56 and 24.63 ($(\mathbf{C}H_3)_2CHCH_2CH_2$); 26.27 ($(CH_3)_2CHCH_2\mathbf{C}H_2$); 29.79 ($(CH_3)_2\mathbf{C}HCH_2CH_2$); 40.47 ($CH_2$-2'); 41.74 ($(CH_3)_2CH\mathbf{C}H_2CH_2$-B); 68.34 (d, $J_{C,P}$ = 5.7, CH_2-5'); 73.97 (CH-3'); 85.28 (CH-1'); 87.53 (d, $J_{C,P}$ = 8.6, CH-4'); 102.34 (C-4a); 117.42 (CH-6); 124.27 (C-5); 154.26 (C-7a); 155.41 (C-2); 164.24 (C-4). ^{31}P NMR (202.4 MHz, D_2O): -21.44 (t, 1P, $J_{\beta,\alpha} = J_{\beta,\gamma}$ = 19.6, P_β); -10.30 (d, 1P, $J_{\alpha,\beta}$ = 19.6, P_α); -6.11 (m, 1P, P_γ).

MS (ESI): *m/z*: 415.1 $[M\text{-}2PO_3Na\text{-}Na]^-$; 495.1 $[M\text{-}PO_3Na\text{-}2Na+H]^-$; 517.1 $[M\text{-}PO_3Na\text{-}Na]^-$; 597.1 $[M\text{-}2Na+H]^-$.
HRMS (ESI): *m/z*: calcd for $C_{16}H_{25}O_{13}N_4P_3Na$ $[M\text{-}2Na+H]^-$: 597.05341; found: 597.05207.

2) Experimental section - biochemistry

General remarks

All agarose and PAGE gels were analysed by fluorescence or phosphorous imaging using Typhoon FLA 9500 (GE Healthcare). The MALDI-TOF spectra of modified oligonucleotides were measured on UltrafleXtreme MALDI-TOF/TOF (Bruker) mass spectrometer with 1 kHz smartbeam II laser technology. The matrix consisted of 3-hydroxypicolinic acid (HPA)/picolinic acid (PA)/ ammonium tartrate in ratio 9/1/1. The matrix (1 μL) was applied to the target (ground steel) and dried down at room temperature. UV-Vis spectra were measured at room temperature in a NanoDrop1000 (ThermoScientific). Synthetic oligonucleotides (unmodified, 5´-biotinylated, 5´-phosphorylated or 3´-sC3-modified templates; unmodified, 5´-(6-FAM) or Seq+-labelled primers, for sequences see Table S4) were purchased from Generi Biotech (Czech Republic). Natural nucleoside triphosphates (dATP, TTP, dCTP, dGTP) were purchased from ThermoScientific. KOD XL DNA polymerase was purchased from Merck (Sigma Aldrich); Pwo, Deep Vent (exo-), Vent (exo-) and LongAmp DNA polymerases as well as

T4 PNK were purchased from New England Biolabs. KAPA HiFi Hotstart Master Mix was purchased from Roche. [γ-^{32}P]-ATP was purchased from M.G.P. spol.s.r.o. Streptavidin magnetic particles (Roche) were obtained from Sigma Aldrich and AMPure XP from Beckman Coulter. Nuclease-free water was used for all experiments. PAGE stop solution used after PEX reactions contains: 95% [v/v] formamide, 0.5 mM EDTA, 0.025% [w/v] bromophenol blue and 0.025%, [w/v] xylene cyanol, 0.025% [w/v] SDS and Milli-Q water. Samples after PEX reactions were always separated on a 12.5% PAGE (acrylamide/bisacrylamide 19:1,25% urea) under denaturing conditions in 1X TBE buffer (42 mA, 1 h). Samples after PCR reactions were always separated with a 3% agarose gel (Serva) in 0.5X TBE buffer (120 V, 2 h) using 6X DNA Gel Loading Dye (ThermoFisher Scientific). Modified ssONs were purified on HPLC C18 column (Waters X Bridge BEH C18 2.5 μm, 4.6 x 150 mm). PEX and PCR products were purified by QIAquick Nucleotide Removal Kit and QIAquick PCR Purification Kit, respectively (Qiagen). The PCR reactions were performed in a C1000 Touch thermal cycler (Biorad). The pH values were determined using inoLab pH 720. Other chemicals were of analytical grade.

Table S4. List of oligonucleotides used in this study

ON Name	Size (nt)	Sequence in 5´→3´ direction with primer regions underlined
**Oligo1A**[a]	19	CCCTCCCATGCCGCCCATG
**Oligo1T**[a]	19	CCCACCCATGCCGCCCATG
**Oligo1C**[a]	19	CCCGCCCATGCCGCCCATG
**Oligo1G**[a]	19	AAACCCCATGCCGCCCATG
**Prb4basII**[a]	31	CTAGCATGAGCTCAGTCCCATGCCGCCCATG
**NickMO1**[a]	35	CTAGCATGAGCTCAGTCGAGGACTCACTAGATCGG
**MO35**[a]	35	CAGTCTAGCATGAGCTCAGTCCCATGCCGCCCATG
**MO43**[a]	43	CATGAGCTCAGTCTAGCATGAGCTCAGTCCCATGCCGCCCATG
**MO47**[a]	47	CTAGCATGAGCTCAGTCTAGCATGAGCTCAGTCCCATGCCGCCCATG
**MO61**	61	GACATCATGAGAGACATCGCCTAGCATGAGCTCAGTAAGGAATACAGGTATTTTGTCCTTG
**MO77**[b,d]	77	GACATCATGAGAGACATCGCCTAGCATGAGCTCAGTCTAGCATGAGCTCAGTAAGGAATACAGGTATTTTGTCCTTG
**MO77OPP**	77	CAAGGACAAAATACCTGTATTCCTTACTGAGCTCATGCTAGACTGAGCTCATGCTAGGCGATGTCTCTCATGATGTC
**A77**	77	GACATCATGAGAGACATCGCACGTAATGCAAAGCTAAAATCGAAAACTGAAAAAGGAATACAGGTATTTTGTCCTTG
**T77**	77	GACATCATGAGAGACATCGCTACGTTGCATTTCAGTTTTGACTTTTAGCTTTAAGGAATACAGGTATTTTGTCCTTG
**C77**	77	GACATCATGAGAGACATCGCCAGTCCTGACCCGATCCCCTAGCCCCATGCCCAAGGAATACAGGTATTTTGTCCTTG

**G77**	77	GACATCATGAGAGACATCGCGACTGGTCAGGGCATGGGGTACGGGGATCGGGAAGGAATACAGGTATTTTGTCCTTG
**FVL-A**	98	GACATCATGAGAGACATCGCCTCTGGGCTAATAGGACTACTTCTAATCTGTAAGAGCAGATCCCTGGACAGGCAAGGAATACAGGTATTTTGTCCTTG
**MO120**	120	GACATCATGAGAGACATCGCCTAGCATGAGCTCAGTCTAGCATGAGCTCAGTCTAGCATGAGCTCAGTCTAGCATGAGCTCAGTCTAGCATGAGCAAGGAATACAGGTATTTTGTCCTTG
**MO150**	150	GACATCATGAGAGACATCGCCTAGCATGAGCTCAGTCTAGCATGAGCTCAGTCTAGCATGAGCTCAGTCTAGCATGAGCTCAGTCTAGCATGAGCTCAGTCTAGCATGAGCTCAGTCTAGCATGAAAGGAATACAGGTATTTTGTCCTTG
Primers		
**Prim248short**[c]	15	CATGGGCGGCATGGG
**PrimNick**	19	CCGATCTAGTGAGTCCTCG
**L20**[c]	20	GACATCATGAGAGACATCGC
**L20_Seq+**[e]	20+	$(N)_x$GACATCATGAGAGACATCGC
**Flank**[c]	20	CATTCGGCTGCTCTTGATTT
**Flank_Seq+**[e]	20+	$(N)_x$CATTCGGCTGCTCTTGATTT
**LT25TH**[c]	25	CAAGGACAAAATACCTGTATTCCTT
**Flank_LT25TH**[c]	45	CATTCGGCTGCTCTTGATTTCAAGGACAAAATACCTGTATTCCTT
**Flank_adapter**	53	TCGTCGGCAGCGTCAGATGTGTATAAGAGACAGCATTCGGCTGCTCTTGATTT
**L20_adapter**	54	GTCTCGTGGGCTCGGAGATGTGTATAAGAGACAGGACATCATGAGAGACATCGC
**F_index**	47	CAAGCAGAAGACGGCATACGAGAT[unique_8nt]GTCTCGTGGGCTCGG
**R_index**	51	AATGATACGGCGACCACCGAGATCTACAC[unique_8nt]TCGTCGGCAGCGTC

[a] 5´-biotinylated; [b] 5´-phosphorylated; [c] 5´-(6-FAM); [d] 3´-sC3; **[e]** extended at 5´-end with unknown sequence

Table S5. List of synthesized modified ssDNA / dsDNA

Name	Sequence in 5´→3´ (primer regions underlined)
**17ON_N^{ER}**	$C^{EAlk}C^{EAlk}G^{EiPr}A^{EIn}U^{EPh}C^{EAlk}U^{EPh}A^{EIn}G^{EiPr}U^{EPh}G^{EiPr}A^{EIn}G^{EiPr}U^{EPh}C^{EAlk}C^{EAlk}U^{EPh}$
**19ON_A^{EIn}**	CATGGGCGGCATGGGA^{EIn}GGG
**19ON_U^{EPh}**	CATGGGCGGCATGGGU^{EPh}GGG
**19ON_C^{EAlk}**	CATGGGCGGCATGGGC^{EAlk}GGG
**19ON_G^{EiPr}**	CATGGGCGGCATGGGG^{EiPr}TTT
**19ON_A^{AIn}**	CATGGGCGGCATGGGA^{AIn}GGG
**19ON_U^{APh}**	CATGGGCGGCATGGGU^{APh}GGG
**19ON_C^{AAlk}**	CATGGGCGGCATGGGC^{AAlk}GGG
**19ON_G^{AiPr}**	CATGGGCGGCATGGGG^{AiPr}TTT
**31ON_A^{EIn}**	CATGGGCGGCATGGGA^{EIn}CTGA^{EIn}GCTCA^{EIn}TGCTA^{EIn}G
**31ON_U^{EPh}**	CATGGGCGGCATGGGACU^{EPh}GAGCU^{EPh}CAU^{EPh}GCU^{EPh}AG
**31ON_C^{EAlk}**	CATGGGCGGCATGGGAC^{EAlk}TGAGC^{EAlk}TC^{EAlk}ATGC^{EAlk}TAG
**31ON_G^{EiPr}**	CATGGGCGGCATGGGACTG^{EiPr}AG^{EiPr}CTCATG^{EiPr}CTAG^{EiPr}
**31ON_A^{AIn}**	CATGGGCGGCATGGGA^{AIn}CTGA^{AIn}GCTCA^{Ain}TGCTA^{AIn}G
**31ON_U^{APh}**	CATGGGCGGCATGGGACU^{APh}GAGCU^{APh}CAU^{APh}GCU^{APh}AG
**31ON_C^{AAlk}**	CATGGGCGGCATGGGAC^{AAlk}TGAGC^{AAlk}TC^{AAlk}ATGC^{AAlk}TAG
**31ON_G^{AiPr}**	CATGGGCGGCATGGGACTG^{AiPr}AG^{AiPr}CTCATG^{AiPr}CTAG^{AiPr}
**31ON_N^{ER}**	CATGGGCGGCATGGG$A^{EIn}C^{EAlk}U^{EPh}G^{EiPr}A^{EIn}G^{EiPr}C^{EAlk}U^{EPh}C^{EAlk}A^{EIn}U^{EPh}G^{EiPr}C^{EAlk}U^{EPh}A^{EIn}G^{EiPr}$

31ON_N^{AR}
CATGGGCGGCATGGGA^{AIn}C^{AAlk}U^{APh}G^{AiPr}A^{AIn}G^{AiPr}C^{AAlk}U^{APh}C^{AAlk}A^{AIn}U^{APh}G^{AiPr}C^{AAlk}U^{APh}A^{AIn}G^{AiPr}

31ON_U^{APh}A^{EIn}C^{AAlk}G^{EiPr}
CATGGGCGGCATGGGA^{EIn}C^{AAlk}U^{APh}G^{EiPr}A^{EIn}G^{EiPr}C^{AAlk}U^{APh}C^{AAlk}A^{EIn}U^{APh}G^{EiPr}C^{AAlk}U^{APh}A^{EIn}G^{EiPr}

31ON_U^{EPh}A^{AIn}C^{EAlk}G^{AiPr}
CATGGGCGGCATGGGA^{AIn}C^{EAlk}U^{EPh}G^{AiPr}A^{AIn}G^{AiPr}C^{EAlk}U^{EPh}C^{EAlk}A^{AIn}U^{EPh}G^{AiPr}C^{EAlk}U^{EPh}A^{AIn}G^{AiPr}

31ON_U^{EPh}A^{EIn}C^{AAlk}G^{AiPr}
CATGGGCGGCATGGGA^{EIn}C^{AAlk}U^{EPh}G^{AiPr}A^{EIn}G^{AiPr}C^{AAlk}U^{EPh}C^{AAlk}A^{EIn}U^{EPh}G^{AiPr}C^{AAlk}U^{EPh}A^{EIn}G^{AiPr}

31ON_U^{APh}A^{AIn}C^{EAlk}G^{EiPr}
CATGGGCGGCATGGGA^{AIn}C^{EAlk}U^{APh}G^{EiPr}A^{AIn}G^{EiPr}C^{EAlk}U^{APh}C^{EAlk}A^{AIn}U^{APh}G^{EiPr}C^{EAlk}U^{APh}A^{AIn}G^{EiPr}

31ON_U^{APh}A^{EIn}C^{EAlk}G^{AiPr}
CATGGGCGGCATGGGA^{EIn}C^{EAlk}U^{APh}G^{AiPr}A^{EIn}G^{AiPr}C^{EAlk}U^{APh}C^{EAlk}A^{EIn}U^{APh}G^{AiPr}C^{EAlk}U^{APh}A^{EIn}G^{AiPr}

31ON_U^{EPh}A^{AIn}C^{AAlk}G^{EiPr}
CATGGGCGGCATGGGA^{AIn}C^{AAlk}U^{EPh}G^{EiPr}A^{AIn}G^{EiPr}C^{AAlk}U^{EPh}C^{AAlk}A^{AIn}U^{EPh}G^{EiPr}C^{AAlk}U^{EPh}A^{AIn}G^{EiPr}

35ON_N^{ER}
CATGGGCGGCATGGGA^{EIn}C^{EAlk}U^{EPh}G^{EiPr}A^{EIn}G^{EiPr}C^{EAlk}U^{EPh}C^{EAlk}A^{EIn}U^{EPh}G^{EiPr}C^{EAlk}U^{EPh}A^{EIn}G^{EiPr}A^{EIn}C^{EAlk}U^{EPh}G^{EiPr}

35ON_N^{AR}
CATGGGCGGCATGGGA^{AIn}C^{AAlk}U^{APh}G^{AiPr}A^{AIn}G^{AiPr}C^{AAlk}U^{APh}C^{AAlk}A^{AIn}U^{APh}G^{AiPr}C^{AAlk}U^{APh}A^{AIn}G^{AiPr}A^{AIn}C^{AAlk}U^{APh}G^{AiPr}

43ON_N^{ER}
CATGGGCGGCATGGGA^{EIn}C^{EAlk}U^{EPh}G^{EiPr}A^{EIn}G^{EiPr}C^{EAlk}U^{EPh}C^{EAlk}A^{EIn}U^{EPh}G^{EiPr}C^{EAlk}U^{EPh}A^{EIn}G^{EiPr}A^{EIn}C^{EAlk}U^{EPh}G^{EiPr}A^{EIn}G^{EiPr}C^{EAlk}U^{EPh}C^{EAlk}A^{EIn}U^{EPh}G^{EiPr}

47ON_N^{ER}
CATGGGCGGCATGGGA^{EIn}C^{EAlk}U^{EPh}G^{EiPr}A^{EIn}G^{EiPr}C^{EAlk}U^{EPh}C^{EAlk}A^{EIn}U^{EPh}G^{EiPr}C^{EAlk}U^{EPh}A^{EIn}G^{EiPr}A^{EIn}C^{EAlk}U^{EPh}G^{EiPr}A^{EIn}G^{EiPr}C^{EAlk}U^{EPh}C^{EAlk}A^{EIn}U^{EPh}G^{EiPr}C^{EAlk}U^{EPh}A^{EIn}G^{EiPr}

***98PCR_A^{EIn}**
CAAGGACAAAATACCTGTATTCCTTGCCTGTCCA^{EIn}GGGA^{EIn}TCTGCTCTTA^{EIn}CA^{EIn}GA^{EIn}TTA^{EIn}GA^{EIn}A^{EIn}GTA^{EIn}GTCCTA^{EIn}TTA^{EIn}GCCCA^{EIn}GA^{EIn}GGCGA^{EIn}TGTCTCTCA^{EIn}TGA^{EIn}TGTC

***98PCR_U^{EPh}**
CAAGGACAAAATACCTGTATTCCTTGCCU^{EPh}GU^{EPh}CCAGGGAU^{EPh}CU^{EPh}GCU^{EPh}CU^{EPh}U^{EPh}ACAGAU^{EPh}U^{EPh}AGAAGU^{EPh}AGU^{EPh}CCU^{EPh}AU^{EPh}U^{EPh}AGCCCAGAGGCGAU^{EPh}GU^{EPh}CU^{EPh}CU^{EPh}CAU^{EPh}GAU^{EPh}GU^{EPh}C

***98PCR_C^{EAlk}**
CAAGGACAAAATACCTGTATTCCTTGC^{EAlk}C^{EAlk}TGTC^{EAlk}C^{EAlk}AGGGATC^{EAlk}TGC^{EAlk}TC^{EAlk}TTAC^{EAlk}AGATTAGAAGTAGTC^{EAlk}C^{EAlk}TATTAGC^{EAlk}C^{EAlk}C^{EAlk}AGAGGC^{EAlk}GATGTC^{EAlk}TC^{EAlk}TC^{EAlk}ATGATGTC^{EAlk}

***98PCR_G^{EiPr}**
CAAGGACAAAATACCTGTATTCCTTG^{EiPr}CCTG^{EiPr}TCCAG^{EiPr}G^{EiPr}G^{EiPr}ATCTG^{EiPr}CTCTTACAG^{EiPr}ATTAG^{EiPr}AAG^{EiPr}TAG^{EiPr}TCCTATTAG^{EiPr}CCCAG^{EiPr}AG^{EiPr}G^{EiPr}CG^{EiPr}ATG^{EiPr}TCTCTCATG^{EiPr}ATG^{EiPr}TC

***98PCR_A^{AIn}**
CAAGGACAAAATACCTGTATTCCTTGCCTGTCCA^{AIn}GGGA^{AIn}TCTGCTCTTA^{AIn}CA^{AIn}GA^{AIn}TTA^{AIn}GA^{AIn}A^{AIn}GTA^{AIn}GTCCTA^{AIn}TTA^{AIn}GCCCA^{AIn}GA^{AIn}GGCGA^{AIn}TGTCTCTCA^{AIn}TGA^{AIn}TGTC

***98PCR_U^{APh}**
CAAGGACAAAATACCTGTATTCCTTGCCU^{APh}GU^{APh}CCAGGGAU^{APh}CU^{APh}GCU^{APh}CU^{APh}U^{APh}ACAGAU^{APh}U^{APh}AGAAGU^{APh}AGU^{APh}CCU^{APh}AU^{APh}U^{APh}AGCCCAGAGGCGAU^{APh}GU^{APh}CU^{APh}CU^{APh}CAU^{APh}GAU^{APh}GU^{APh}C

***98PCR_C^{AAlk}**
CAAGGACAAAATACCTGTATTCCTTGC^{AAlk}C^{AAlk}TGTC^{AAlk}C^{AAlk}AGGGATC^{AAlk}TGC^{AAlk}TC^{AAlk}TTAC^{AAlk}AGATTAGAAGTAGTC^{AAlk}C^{AAlk}TATTAGC^{AAlk}C^{AAlk}C^{AAlk}AGAGGC^{AAlk}GATGTC^{AAlk}TC^{AAlk}TC^{AAlk}ATGATGTC^{AAlk}

***98PCR_G^{AiPr}**
CAAGGACAAAATACCTGTATTCCTTG^{AiPr}CCTG^{AiPr}TCCAG^{AiPr}G^{AiPr}G^{AiPr}ATCTG^{AiPr}CTCTTACAG^{AiPr}ATTAG^{AiPr}AAG^{AiPr}TAG^{AiPr}TCCTATTAG^{AiPr}CCCAG^{AiPr}AG^{AiPr}G^{AiPr}CG^{AiPr}ATG^{AiPr}TCTCTCATG^{AiPr}ATG^{AiPr}TC

77ON_N^{ER}
CAAGGACAAAATACCTGTATTCCTTA^{EIn}C^{EAlk}U^{EPh}G^{EiPr}A^{EIn}G^{EiPr}C^{EAlk}U^{EPh}C^{EAlk}A^{EIn}U^{EPh}G^{EiPr}C^{EAlk}U^{EPh}A^{EIn}G^{EiPr}A^{EIn}C^{EAlk}U^{EPh}G^{EiPr}A^{EIn}G^{EiPr}C^{EAlk}U^{EPh}C^{EAlk}A^{EIn}U^{EPh}G^{EiPr}C^{EAlk}U^{EPh}A^{EIn}G^{EiPr}G^{EiPr}C^{EAlk}G^{EiPr}A^{EIn}U^{EPh}G^{EiPr}U^{EPh}C^{EAlk}U^{EPh}C^{EAlk}U^{EPh}C^{EAlk}A^{EIn}U^{EPh}G^{EiPr}A^{EIn}U^{EPh}G^{EiPr}U^{EPh}C^{EAlk}

77cON_N^{ER}
GACATCATGAGAGACATCGCC^{EAlk}U^{EPh}A^{EIn}G^{EiPr}C^{EAlk}A^{EIn}U^{EPh}G^{EiPr}A^{EIn}G^{EiPr}C^{EAlk}U^{EPh}C^{EAlk}A^{EIn}G^{EiPr}U^{EPh}C^{EAlk}U^{EPh}A^{EIn}G^{EiPr}C^{EAlk}A^{EIn}U^{EPh}G^{EiPr}A^{EIn}G^{EiPr}C^{EAlk}U^{EPh}C^{EAlk}A^{EIn}G^{EiPr}U^{EPh}A^{EIn}A^{EIn}G^{EiPr}G^{EiPr}A^{EIn}A^{EIn}U^{EPh}A^{EIn}C^{EAlk}A^{EIn}G^{EiPr}G^{EiPr}U^{EPh}A^{EIn}U^{EPh}U^{EPh}U^{EPh}U^{EPh}G^{EiPr}U^{EPh}C^{EAlk}C^{EAlk}U^{EPh}U^{EPh}G^{EiPr}

**97ON_N^{ER}**	CATTCGGCTGCTCTTGATTTCAAGGACAAAATACCTGTATTCCTTA^{EIn}C^{EAlk}U^{EPh}G^{EiPr}A^{EIn}G^{EiPr}C^{EAlk}U^{EPh}C^{EAlk}A^{EIn}U^{EPh}G^{EiPr}C^{EAlk}U^{EPh}A^{EIn}G^{EiPr}A^{EIn}C^{EAlk}U^{EPh}G^{EiPr}A^{EIn}G^{EiPr}C^{EAlk}U^{EPh}C^{EAlk}A^{EIn}U^{EPh}G^{EiPr}C^{EAlk}U^{EPh}A^{EIn}G^{EiPr}G^{EiPr}C^{EAlk}G^{EiPr}A^{EIn}U^{EPh}G^{EiPr}U^{EPh}C^{EAlk}U^{EPh}C^{EAlk}U^{EPh}C^{EAlk}A^{EIn}U^{EPh}G^{EiPr}A^{EIn}U^{EPh}G^{EiPr}U^{EPh}C^{EAlk}
**77ON_A_N^{ER}**	CAAGGACAAAATACCTGTATTCCTTA^{EIn}A^{EIn}A^{EIn}G^{EiPr}C^{EAlk}U^{EPh}A^{EIn}A^{EIn}A^{EIn}A^{EIn}G^{EiPr}U^{EPh}C^{EAlk}A^{EIn}A^{EIn}A^{EIn}A^{EIn}C^{EAlk}U^{EPh}G^{EiPr}A^{EIn}A^{EIn}A^{EIn}U^{EPh}G^{EiPr}C^{EAlk}A^{EIn}A^{EIn}C^{EAlk}G^{EiPr}U^{EPh}A^{EIn}G^{EiPr}C^{EAlk}G^{EiPr}A^{EIn}U^{EPh}G^{EiPr}U^{EPh}C^{EAlk}U^{EPh}C^{EAlk}U^{EPh}C^{EAlk}A^{EIn}U^{EPh}G^{EiPr}A^{EIn}U^{EPh}G^{EiPr}U^{EPh}C^{EAlk}
**77ON_U_N^{ER}**	CAAGGACAAAATACCTGTATTCCTTU^{EPh}U^{EPh}U^{EPh}C^{EAlk}A^{EIn}G^{EiPr}U^{EPh}U^{EPh}U^{EPh}U^{EPh}C^{EAlk}G^{EiPr}A^{EIn}U^{EPh}U^{EPh}U^{EPh}U^{EPh}A^{EIn}G^{EiPr}C^{EAlk}U^{EPh}U^{EPh}U^{EPh}G^{EiPr}C^{EAlk}A^{EIn}U^{EPh}U^{EPh}A^{EIn}C^{EAlk}G^{EiPr}U^{EPh}G^{EiPr}C^{EAlk}G^{EiPr}A^{EIn}U^{EPh}G^{EiPr}U^{EPh}C^{EAlk}U^{EPh}C^{EAlk}U^{EPh}C^{EAlk}A^{EIn}U^{EPh}G^{EiPr}A^{EIn}U^{EPh}G^{EiPr}U^{EPh}C^{EAlk}
**77ON_C_N^{ER}**	CAAGGACAAAATACCTGTATTCCTTC^{EAlk}C^{EAlk}C^{EAlk}G^{EiPr}A^{EIn}U^{EPh}C^{EAlk}C^{EAlk}C^{EAlk}C^{EAlk}G^{EiPr}U^{EPh}A^{EIn}C^{EAlk}C^{EAlk}C^{EAlk}C^{EAlk}A^{EIn}U^{EPh}G^{EiPr}C^{EAlk}C^{EAlk}C^{EAlk}U^{EPh}G^{EiPr}A^{EIn}C^{EAlk}C^{EAlk}A^{EIn}G^{EiPr}U^{EPh}C^{EAlk}G^{EiPr}C^{EAlk}G^{EiPr}A^{EIn}U^{EPh}G^{EiPr}U^{EPh}C^{EAlk}U^{EPh}C^{EAlk}U^{EPh}C^{EAlk}A^{EIn}U^{EPh}G^{EiPr}A^{EIn}U^{EPh}G^{EiPr}U^{EPh}C^{EAlk}
**77ON_G_N^{ER}**	CAAGGACAAAATACCTGTATTCCTTG^{EiPr}G^{EiPr}G^{EiPr}C^{EAlk}A^{EIn}U^{EPh}G^{EiPr}G^{EiPr}G^{EiPr}G^{EiPr}C^{EAlk}U^{EPh}A^{EIn}G^{EiPr}G^{EiPr}G^{EiPr}G^{EiPr}A^{EIn}U^{EPh}C^{EAlk}G^{EiPr}G^{EiPr}G^{EiPr}U^{EPh}C^{EAlk}A^{EIn}G^{EiPr}G^{EiPr}A^{EIn}C^{EAlk}U^{EPh}G^{EiPr}G^{EiPr}C^{EAlk}G^{EiPr}A^{EIn}U^{EPh}G^{EiPr}U^{EPh}C^{EAlk}U^{EPh}C^{EAlk}U^{EPh}C^{EAlk}A^{EIn}U^{EPh}G^{EiPr}A^{EIn}U^{EPh}G^{EiPr}U^{EPh}C^{EAlk}
**98ON_N^{ER}**	CAAGGACAAAATACCTGTATTCCTTG^{EiPr}C^{EAlk}C^{EAlk}U^{EPh}G^{EiPr}U^{EPh}C^{EAlk}G^{EiPr}A^{EIn}G^{EiPr}G^{EiPr}G^{EiPr}A^{EIn}U^{EPh}C^{EAlk}U^{EPh}G^{EiPr}C^{EAlk}U^{EPh}C^{EAlk}U^{EPh}U^{EPh}A^{EIn}C^{EAlk}A^{EIn}G^{EiPr}A^{EIn}U^{EPh}U^{EPh}A^{EIn}G^{EiPr}A^{EIn}A^{EIn}G^{EiPr}U^{EPh}A^{EIn}G^{EiPr}U^{EPh}C^{EAlk}C^{EAlk}U^{EPh}A^{EIn}U^{EPh}U^{EPh}A^{EIn}G^{EiPr}C^{EAlk}C^{EAlk}C^{EAlk}A^{EIn}G^{EiPr}A^{EIn}G^{EiPr}G^{EiPr}C^{EAlk}G^{EiPr}A^{EIn}U^{EPh}G^{EiPr}U^{EPh}C^{EAlk}U^{EPh}C^{EAlk}U^{EPh}C^{EAlk}A^{EIn}U^{EPh}G^{EiPr}A^{EIn}U^{EPh}G^{EiPr}U^{EPh}C^{EAlk}
**120ON_N^{ER}**	CAAGGACAAAATACCTGTATTCCTTG^{EiPr}C^{EAlk}U^{EPh}C^{EAlk}A^{EIn}U^{EPh}G^{EiPr}C^{EAlk}U^{EPh}A^{EIn}G^{EiPr}A^{EIn}C^{EAlk}U^{EPh}G^{EiPr}A^{EIn}G^{EiPr}C^{EAlk}U^{EPh}C^{EAlk}A^{EIn}U^{EPh}G^{EiPr}C^{EAlk}U^{EPh}A^{EIn}G^{EiPr}A^{EIn}C^{EAlk}U^{EPh}G^{EiPr}A^{EIn}G^{EiPr}C^{EAlk}U^{EPh}C^{EAlk}A^{EIn}U^{EPh}G^{EiPr}C^{EAlk}U^{EPh}A^{EIn}G^{EiPr}A^{EIn}C^{EAlk}U^{EPh}G^{EiPr}A^{EIn}G^{EiPr}C^{EAlk}U^{EPh}C^{EAlk}A^{EIn}U^{EPh}G^{EiPr}C^{EAlk}U^{EPh}A^{EIn}G^{EiPr}A^{EIn}C^{EAlk}U^{EPh}G^{EiPr}A^{EIn}G^{EiPr}C^{EAlk}U^{EPh}C^{EAlk}A^{EIn}U^{EPh}G^{EiPr}C^{EAlk}U^{EPh}A^{EIn}G^{EiPr}G^{EiPr}C^{EAlk}G^{EiPr}A^{EIn}U^{EPh}G^{EiPr}U^{EPh}C^{EAlk}U^{EPh}C^{EAlk}U^{EPh}C^{EAlk}A^{EIn}U^{EPh}G^{EiPr}A^{EIn}U^{EPh}G^{EiPr}U^{EPh}C^{EAlk}
**150ON_N^{ER}**	CAAGGACAAAATACCTGTATTCCTTU^{EPh}C^{EAlk}A^{EIn}U^{EPh}G^{EiPr}C^{EAlk}U^{EPh}A^{EIn}G^{EiPr}A^{EIn}C^{EAlk}U^{EPh}G^{EiPr}A^{EIn}G^{EiPr}C^{EAlk}U^{EPh}C^{EAlk}A^{EIn}U^{EPh}G^{EiPr}C^{EAlk}U^{EPh}A^{EIn}G^{EiPr}A^{EIn}C^{EAlk}U^{EPh}G^{EiPr}A^{EIn}G^{EiPr}C^{EAlk}U^{EPh}C^{EAlk}A^{EIn}U^{EPh}G^{EiPr}C^{EAlk}U^{EPh}A^{EIn}G^{EiPr}A^{EIn}C^{EAlk}U^{EPh}G^{EiPr}A^{EIn}G^{EiPr}C^{EAlk}U^{EPh}C^{EAlk}A^{EIn}U^{EPh}G^{EiPr}C^{EAlk}U^{EPh}A^{EIn}G^{EiPr}A^{EIn}C^{EAlk}U^{EPh}G^{EiPr}A^{EIn}G^{EiPr}C^{EAlk}U^{EPh}C^{EAlk}A^{EIn}U^{EPh}G^{EiPr}C^{EAlk}U^{EPh}A^{EIn}G^{EiPr}A^{EIn}C^{EAlk}U^{EPh}G^{EiPr}A^{EIn}G^{EiPr}C^{EAlk}U^{EPh}C^{EAlk}A^{EIn}U^{EPh}G^{EiPr}C^{EAlk}U^{EPh}A^{EIn}G^{EiPr}G^{EiPr}C^{EAlk}G^{EiPr}A^{EIn}U^{EPh}G^{EiPr}U^{EPh}C^{EAlk}U^{EPh}C^{EAlk}U^{EPh}C^{EAlk}A^{EIn}U^{EPh}G^{EiPr}A^{EIn}U^{EPh}G^{EiPr}U^{EPh}C^{EAlk}

*complementary strand is modified in the same manner using modification under study (primer regions are not modified)

2.1 PEX - Single incorporation (one modified dN^{R}TP)

Method: Reaction mixture (10 µL) contained one of Oligo 1A / Oligo 1T / Oligo 1C / Oligo 1G (3 µM, 0.75 µL), 5´-(6-FAM)-labelled prim248short primer (3 µM, 0.5 µL), natural dNTP (1 mM, Table S6), modified dN^{R}TP of study (4 mM, 0.5 µL), KOD XL DNA polymerase

(Table S6) and reaction buffer (10X, 1 µL) as supplied by the manufacturer. The reaction mixture was incubated for 30 minutes at 60 °C, stopped by addition of PAGE stop solution (10 µL) and denaturated for 3 minutes at 95 °C. Samples were analysed by PAGE and visualised using fluorescence imaging (Figure S1).

Table S6. Reaction condition specifications for single-incorporation

Entry	Oligo 1N templates	1 mM dNTP	KOD XL	Modified $dN^{R}TP$
1	**Oligo1A**	0.1 µL of dGTP	0.1 U	$\mathbf{dA^{EIn}}$ or $\mathbf{dA^{AIn}}$
2	**Oligo1T**	0.2 µL of dGTP	0.4 U	$\mathbf{dU^{EPh}}$ or $\mathbf{dU^{APh}}$
3	**Oligo1C**	0.1 µL of dGTP	0.4 U	$\mathbf{dC^{EAlk}}$ or $\mathbf{dC^{AAlk}}$
4	**Oligo1G**	0.2 µL of TTP	0.075 U	$\mathbf{dG^{EiPr}}$ or $\mathbf{dG^{AiPr}}$

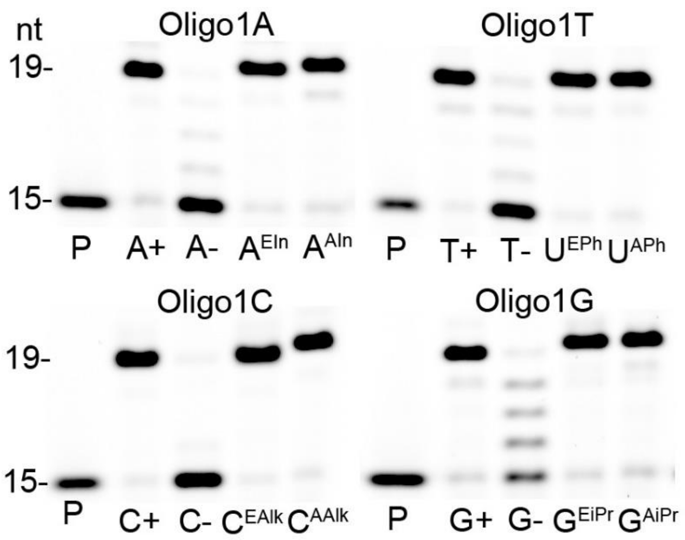

Figure S1. Denaturing PAGE analysis of PEX reaction with one modified $\mathbf{dN^{R}TP}$: (P) primer; Oligo1A template: (A+) dATP, dGTP; (A-) dGTP; (A^{EIn}) $\mathbf{dA^{EIn}TP}$, dGTP; (A^{AIn}) $\mathbf{dA^{AIn}TP}$ dGTP; Oligo 1T template: (T+) TTP, dGTP; (T-) dGTP; (U^{EPh}) $\mathbf{dU^{EPh}TP}$, dGTP; (U^{APh}) $\mathbf{dU^{APh}TP}$, dGTP; Oligo 1C template: (C+) dCTP, dGTP; (C-) dGTP; (C^{EAlk}) $\mathbf{dC^{EAlk}TP}$, dGTP; (C^{AAlk}) $\mathbf{dC^{AAlk}TP}$, dGTP; Oligo 1G template: (G+) dGTP, TTP; (G-) TTP; (G^{EiPr}) $\mathbf{dG^{EiPr}TP}$, TTP; (G^{AiPr}) $\mathbf{dG^{AiPr}TP}$, TTP.

2.2 PEX - Multiple incorporation (one modified $dN^{R}TP$)

Method: Reaction mixture (10 µL) contained prb4basII template (3 µM, 0.75 µL), 5´-(6-FAM)-labelled prim248short primer (3 µM, 0.5 µL), appropriate natural dNTPs (1 µL,

Table S7), modified $\mathbf{dN^{R}TP}$ of study (1 mM, 1 µL), KOD XL DNA polymerase (Table S7) and reaction buffer (10X, 1 µL) as supplied by the manufacturer. The reaction mixture was incubated for 30 minutes at 60 °C, stopped by addition of PAGE stop solution (10 µL) and denaturated for 3 minutes at 95 °C. Samples were analysed by PAGE and visualised using fluorescence imaging (Figure S2).

Table S7. Reaction condition specifications for multiple incorporation

Entry	natural dNTPs	c(dNTPs)	KOD XL	Modified $\mathbf{dN^{R}TP}$
1	TTP, dCTP, dGTP	0.5 mM	0.3 U	$\mathbf{dA^{EIn}}$ or $\mathbf{dA^{AIn}}$
2	dATP, dCTP, dGTP	0.5 mM	0.3 U	$\mathbf{dU^{EPh}}$ or $\mathbf{dU^{APh}}$
3	dATP, TTP, dCTP	0.5 mM	0.4 U	$\mathbf{dG^{EiPr}}$
4	dATP, TTP, dCTP	0.25 mM	0.4 U	$\mathbf{dG^{AiPr}}$
5	dATP, TTP, dGTP	0.25 mM	0.3 U	$\mathbf{dC^{EAlk}}$
6	dATP, TTP, dGTP	0.5 mM	0.4 U	$\mathbf{dC^{AAlk}}$

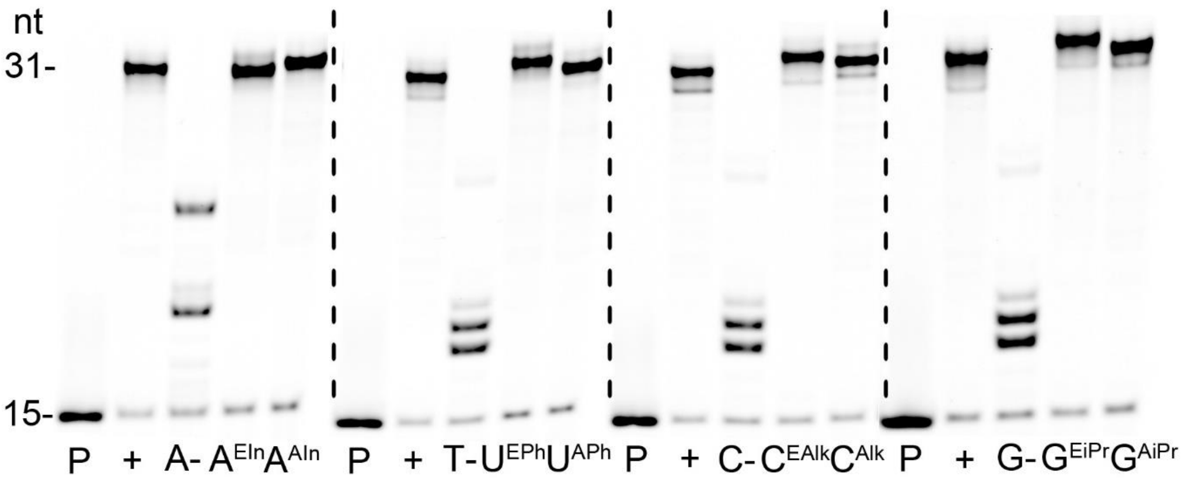

Figure S2. Denaturing PAGE analysis of PEX reaction with one modified $\mathbf{dN^{R}TP}$: (P) primer; (+) natural dNTPs; (A-) TTP, dCTP, dGTP; (A^{EIn}) $\mathbf{dA^{EIn}TP}$, TTP, dCTP, dGTP; (A^{AIn}) $\mathbf{dA^{AIn}TP}$, TTP, dCTP, dGTP; (T-) dATP, dCTP, dGTP; (U^{EPh}) dATP, $\mathbf{dU^{EPh}TP}$, dCTP, dGTP; (U^{APh}) dATP, $\mathbf{dU^{APh}TP}$, dCTP, dGTP; (C-) dATP, TTP, dGTP; (C^{EAlk}) dATP, TTP, $\mathbf{dC^{EAlk}TP}$, dGTP; (C^{AAlk}) dATP, TTP, $\mathbf{dC^{AAlk}TP}$, dGTP; (G-) dATP, TTP, dCTP; (G^{EiPr}) dATP, TTP, dCT, $\mathbf{dG^{EiPr}TP}$; (G^{AiPr}) dATP, TTP, dCTP, $\mathbf{dG^{AiPr}TP}$.

2.3 PEX - Multiple incorporation (four modified dN^{R}TPs in various combinations)

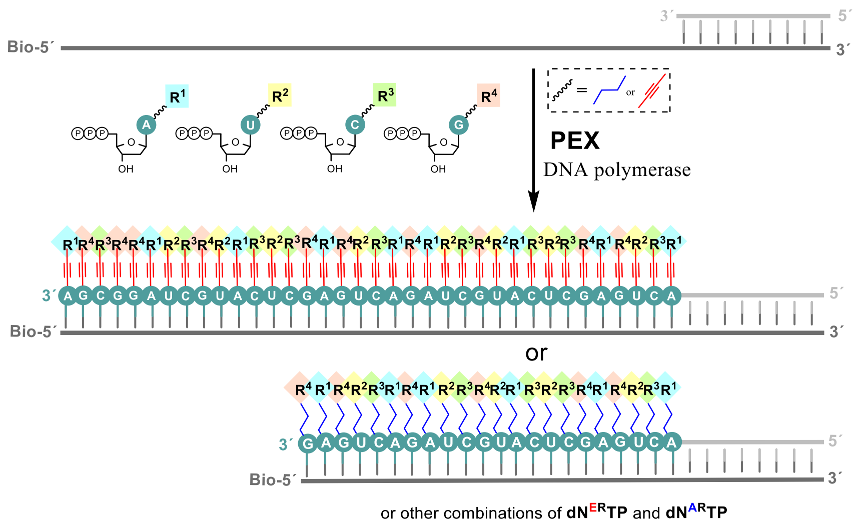

Scheme S4. Primer extension reaction with using set of four modified **dN^{R}TPs**.

Method: Reaction mixture (10 µL) contained prb4basII template (3 µM, 0.75 µL), 5´-(6-FAM)-labelled prim248short primer (3 µM, 0.5 µL), set of four modified **dN^{R}TPs** (2 mM, 1 µL, each), Vent (exo-) DNA polymerase (2 U) and reaction buffer (10X, 1 µL) as supplied by the manufacturer. Positive control contained 0.5 U of Vent (exo-) DNA polymerase and natural dNTPs (1 mM, 1 µL). The reaction mixture was incubated for 60 minutes at 60 °C, stopped by addition of PAGE stop solution (10 µL) and denaturated for 3 minutes at 95 °C. Samples were analysed by PAGE and visualised using fluorescence imaging (Figure S3).

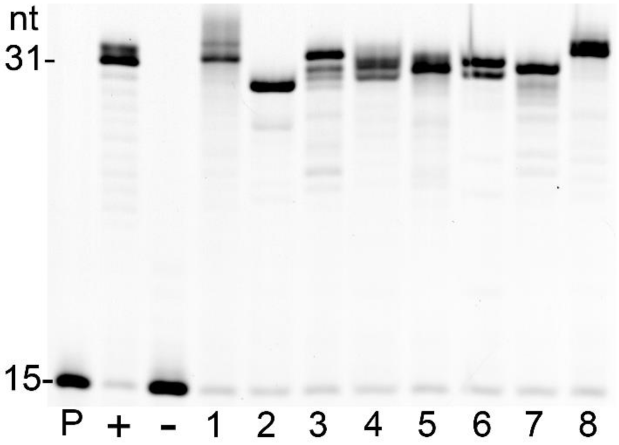

Figure S3. Denaturing PAGE analysis of PEX reactions with various combinations of four modified **dN^R^TPs**: (P) primer; (+) natural dNTPs; (-) no dNTPs; (1) **dA^{EIn}TP**, **dU^{EPh}TP**, **dC^{EAlk}TP**, **dG^{EiPr}TP**; (2) **dA^{AIn}TP**, **dU^{APh}TP**, **dC^{AAlk}TP**, **dG^{AiPr}TP**; (3) **dA^{EIn}TP**, **dU^{APh}TP**, **dC^{AAlk}TP**, **dG^{EiPr}TP**; (4) **dA^{AIn}TP**, **dU^{EPh}TP**, **dC^{EAlk}TP**, **dG^{AiPr}TP**; (5) **dA^{EIn}TP**, **dU^{EPh}TP**, **dC^{AAlk}TP**, **dG^{AiPr}TP**; (6) **dA^{AIn}TP**, **dU^{APh}TP**, **dC^{EAlk}TP**, **dG^{EiPr}TP**; (7) **dA^{EIn}TP**, **dU^{APh}TP**, **dC^{EAlk}TP**, **dG^{AiPr}TP**; (8) **dA^{AIn}TP**, **dU^{EPh}TP**, **dC^{AAlk}TP**, **dG^{EiPr}TP**.

2.4 PEX - Multiple incorporation (four modified dN^{R}TPs in various template length)

Method: Reaction mixture (10 µL) contained one of four templates (prb4basII / MO35 / MO43 / MO47) (3 µM, 0.75 µL), 5´-(6-FAM)-labelled prim248short primer (3 µM, 0.5 µL), set of four modified **dN^{R}TPs** (2 mM, 1 µL), Vent (exo-) DNA polymerase (2 U) and reaction buffer (10X, 1 µL) as supplied by the manufacturer. All positive controls contained 0.5 U of Vent (exo-) DNA polymerase and natural dNTPs (1 mM, 1 µL). The reaction mixture was incubated for 60 minutes at 60 °C, stopped by addition of PAGE stop solution (10 µL) and denaturated for 3 minutes at 95 °C. Samples were analysed by PAGE and visualised using fluorescence imaging (Figure S4).

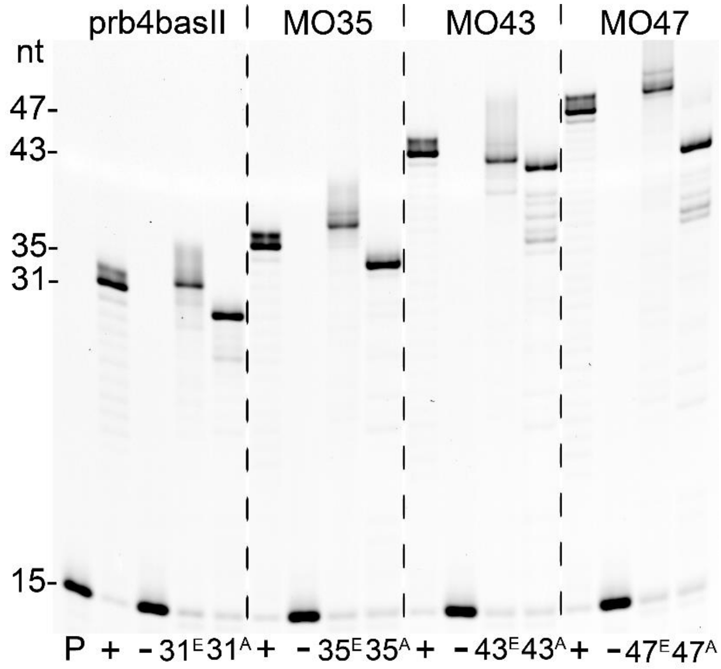

Figure S4. Denaturing PAGE analysis of PEX reactions with set of four modified **dN^{R}TPs** on various templates: (P) primer; (+) natural dNTPs; (-) no dNTPs; (31^{E}) **dA^{EIn}TP**, **dU^{EPh}TP**, **dC^{EAlk}TP**, **dG^{EiPr}TP**; (31^{A}) **dA^{AIn}TP**, **dU^{APh}TP**, **dC^{AAlk}TP**, **dG^{AiPr}TP**; (35^{E}) **dA^{EIn}TP**, **dU^{EPh}TP**, **dC^{EAlk}TP**, **dG^{EiPr}TP**; (35^{A}) **dA^{AIn}TP**, **dU^{APh}TP**, **dC^{AAlk}TP**, **dG^{AiPr}TP**; (43^{E}) **dA^{EIn}TP**, **dU^{EPh}TP**, **dC^{EAlk}TP**, **dG^{EiPr}TP**; (43^{A}) **dA^{AIn}TP**, **dU^{APh}TP**, **dC^{AAlk}TP**, **dG^{AiPr}TP**; (47^{E}) **dA^{EIn}TP**, **dU^{EPh}TP**, **dC^{EAlk}TP**, **dG^{EiPr}TP**; (43^{A}) **dA^{AIn}TP**, **dU^{APh}TP**, **dC^{AAlk}TP**, **dG^{AiPr}TP**.

2.5 General procedure for ssDNA generation via magnetoseparation

In PEX reactions undergoing magnetoseparation, 5´-biotinylated template and non-labelled prim248short primer was used (Table S4). In order to obtain sufficient amount of modified ssDNA for MALDI-TOF measurement, PEX reactions were five times scaled-up.

Method: 100 μL of Streptavidin magnetic beads (SMB) were washed three times with 200 μL of Binding buffer TEN100 (10 mM Tris, 1 mM EDTA, 100 mM NaCl, pH 7.5). The PEX reaction mixture was diluted to 50 μL with water, added to 100 μL of prewashed SMB (1:2) and incubated for 30 minutes (15 °C, 1400 rpm). SMB were captured on magnet (DynaMag-2, Invitrogen), washed successively three times with 200 μL of Washing buffer TEN500 (10 mM Tris, 1 mM EDTA, 500 mM NaCl, pH 7.5) and three times with 200 μL of water. Modified strand was then released by denaturation in 50μL of hot water (incubation for 2 minutes, 75 °C, 900 rpm), SMB bearing template strand was

immediately captured on magnet and water solution containing modified strand was taken out, evaporated and sent for MALDI-TOF measurement (overview of measured spectra

2.6 General procedure for ssDNA generation via λ-exonuclease digestion

Method: Proof of concept was performed on dsDNA obtained by annealing of 5´-(6-FAM)-labelled modified ssON **77ON_N^{ER}** (synthesis described below in section 2.9) with 5´-phosphorylated complementary strand (30 pmol of MO77 for each 20 µL of aPCR reaction). Achieved dsDNA was first purified by Qiaquick PCR purification kit using protocol provided by the manufacturer and set for λ-exonuclease digestion under following conditions: Reaction mixture (40 µL) contained dsDNA with modified **77ON_N^{ER}** strand (2 µM, 10 µL), λ-exonuclease (2.5-10 U) and reaction buffer (10X, 4 µL) as supplied by the manufacturer. Reaction mixture was incubated at 37°C for 30 / 60 or 120 minutes, analysed by agarose gel electrophoresis and visualised using fluorescence imaging (Figure S5).

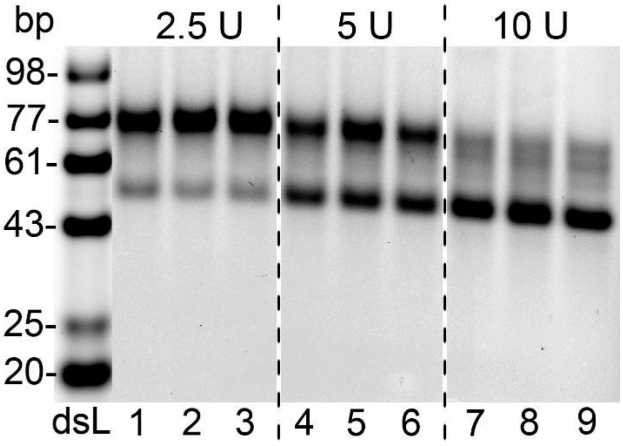

Figure S5. Agarose gel of λ-exonuclease digestion reaction: (lanes 1, 4, 7) 30 minutes; (lanes 2, 5, 8) 60 minutes; (lanes 3, 6, 9) 120 minutes; (dsL) double-stranded ladder.

2.7 MALDI-TOF measurements

Table S8. Overview of modified ssONs and their masses after magnetoseparation

ON Name	Mass calculated [Da]	Mass found [Da]	Figure number
**19ON_U^{EPh}**	6051.9	6053.1	Figure S23
**19ON_A^{EIn}**	6113.1	6114.0	Figure S24
**19ON_C^{EAlk}**	6016.9	6017.5	Figure S25
**19ON_G^{EiPr}**	5981.0	5981.5	Figure S26
**19ON_U^{APh}**	6055.9	6057.0	Figure S27
**19ON_A^{AIn}**	6116.9	6117.7	Figure S28
**19ON_C^{AAlk}**	6021.0	6021.6	Figure S29
**19ON_G^{AiPr}**	5985.1	5985.7	Figure S30
**31ON_U^{EPh}**	9961.3	9962.1	Figure S31
**31ON_A^{EIn}**	10169.5	10170.0	Figure S32
**31ON_C^{EAlk}**	9881.5	9882.0	Figure S33
**31ON_G^{EiPr}**	9877.5	9877.3	Figure S34
**31ON_U^{APh}**	9977.5	9978.2	Figure S35
**31ON_A^{AIn}**	10185.6	10186.1	Figure S36
**31ON_C^{AAlk}**	9897.6	9898.2	Figure S37
**31ON_G^{AiPr}**	9893.7	9894.0	Figure S38
**31ON_N^{ER}**	11037.9	11039.0	Figure S39
**31ON_N^{AR}**	11102.5	11102.9	Figure S40
**31ON_U^{APh}A^{EIn}C^{AAlk}G^{EiPr}**	11070.2	11071.4	Figure S41
**31ON_U^{EPh}A^{AIn}C^{EAlk}G^{AiPr}**	11070.2	11071.4	Figure S42
**31ON_U^{EPh}A^{EIn}C^{AAlk}G^{AiPr}**	11070.2	11070.8	Figure S43
**31ON_U^{APh}A^{AIn}C^{EAlk}G^{EiPr}**	11070.2	11071.4	Figure S44
**31ON_U^{APh}A^{EIn}C^{EAlk}G^{AiPr}**	11070.2	11071.3	Figure S45
**31ON_U^{EPh}A^{AIn}C^{AAlk}G^{EiPr}**	11070.2	11070.4	Figure S46
**35ON_ N^{AR}**	12709.6	12710.9	Figure S47
**35ON_ N^{ER}**	12628.9	12628.8	Figure S48
**43ON_ N^{ER}**	15810.8	16262.3	Figure S49
**47ON_ N^{ER}**	17401.8	17853.03	Figure S50

MALDI-TOF spectra of longer ONs (>50nt) were not carried. For these ONs, we performed Sanger and NGS sequencing (see section 2.11 and 2.12).

2.8 PCR – single incorporation (one modified dN^{R}TP)

Method: Reaction mixture (20 µL) contained FVL-A template (0.5 µM, 1 µL), 5´-(6-FAM)-labelled L20 and LT25TH primers (10 µM, 4 µL, each), natural dNTPs (3 mM, 1.5 µL), modified **dN^{R}TP** of study (4 mM, 4 µL), KOD XL DNA polymerase (Table S9) and reaction buffer (10X, 2 µL) as supplied by the manufacturer. Positive control contained 1.25 U of KOD XL DNA polymerase and natural dNTPs (1 mM, 3 µL). Some of the reaction also contained additives: 25 mM $MgSO_4$ or four-component mixture of 100% DMSO, 5% formamide, 0.75 M betaine, 50 mM TMAC (in ration 1:1:1:1) (Table S9). All reaction mixtures were under cycling protocol: 95 °C for 1 min, followed by 30 cycles at 95 °C for 1 min, 53 °C for 1 min, and 72 °C for 1 min, followed by a final elongation step at 75 °C for 5 min. Amplified products were analysed on agarose gel as well as by PAGE and visualised using fluorescence imaging (Figure S6).

Table S9. Reaction condition specifications for PCR

Entry	modified dN^{R}TP	KOD XL	25 mM $MgSO_4$	Additives mixture*	PCR product
1	**dA^{EIn}**	5 U	-	1 µL	**98PCR_A^{EIn}**
2	**dA^{AIn}**	5 U	1 µL	1 µL	**98PCR_A^{AIn}**
3	**dU^{EPh}**	5 U	1 µL	1 µL	**98PCR_U^{EPh}**
4	**dU^{APh}**	5 U	1 µL	1 µL	**98PCR_U^{APh}**
5	**dC^{EAlk}**	0.75 U	-	-	**98PCR_C^{EAlk}**
6	**dC^{AAlk}**	3.75 U	-	-	**98PCR_C^{AAlk}**
7	**dG^{EiPr}**	5 U	-	1 µL	**98PCR_G^{EiPr}**
8	**dG^{AiPr}**	3.75 U	-	-	**98PCR_G^{AiPr}**

*100% DMSO, 5% formamide, 0.75 M betaine, 50 mM TMAC (in ration 1:1:1:1)

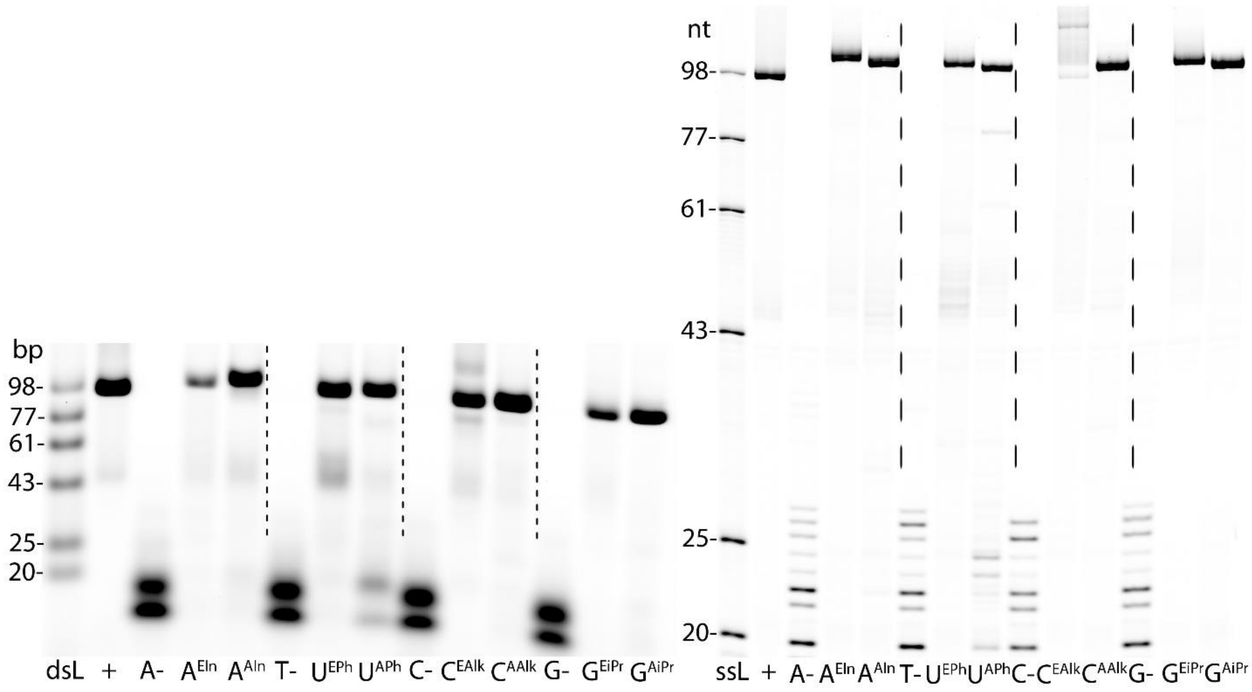

Figure S6. Agarose gel (left) and denaturing PAGE analysis (right) of PCR reactions with one modified **dN^{R}TP**: (+) natural dNTPs; (A-) TTP, dCTP, dGTP; (A^{Eln}) **dA^{Eln}TP**, TTP, dCTP, dGTP; (A^{Aln}) **dA^{Aln}TP**, TTP, dCTP, dGTP; (T-) dATP, dCTP, dGTP; (U^{EPh}) **dU^{EPh}TP**, dATP, dCTP, dGTP; (U^{APh}) **dU^{APh}TP**, dATP, dCTP, dGTP; (C-) dATP, TTP, dGTP; (C^{EAlk}) **dC^{EAlk}TP**, dATP, TTP, dGTP; (C^{AAlk}) **dC^{AAlk}TP**, dATP, TTP, dGTP; (G-) dATP, TTP, dCTP; (G^{EiPr}) **dG^{EiPr}TP**, dATP, TTP, dCTP; (G^{AiPr}) **dG^{AiPr}TP**, dATP, TTP, dCTP; (dsL) double-stranded ladder; (ssL) single-stranded ladder.

2.9 PCR – multiple incorporation (two, three and four modified dN^{R}TPs)

Method: Reaction mixture (20 µL) contained MO77 template (2.5 µM, 2 µL), 5´-(6-FAM)-labelled L20 and LT25TH primers (20 µM, 2 µL, each), modified **dN^{R}TP**s of study (2 mM, 2 µL), Vent (exo-) DNA polymerase and reaction buffer (10X, 2 µL) as supplied by the manufacturer. Negative controls were performed in absence of any dNTPs. All reaction mixtures were under cycling protocol: 95 °C for 1 min, followed by 50 cycles at 95 °C for 1 min, 50 °C for 1 min, and 70 °C for 2 min, followed by a final elongation step at 70 °C for 5 min. Amplified products were analysed on agarose gel and visualised using fluorescence imaging (Figure S7).

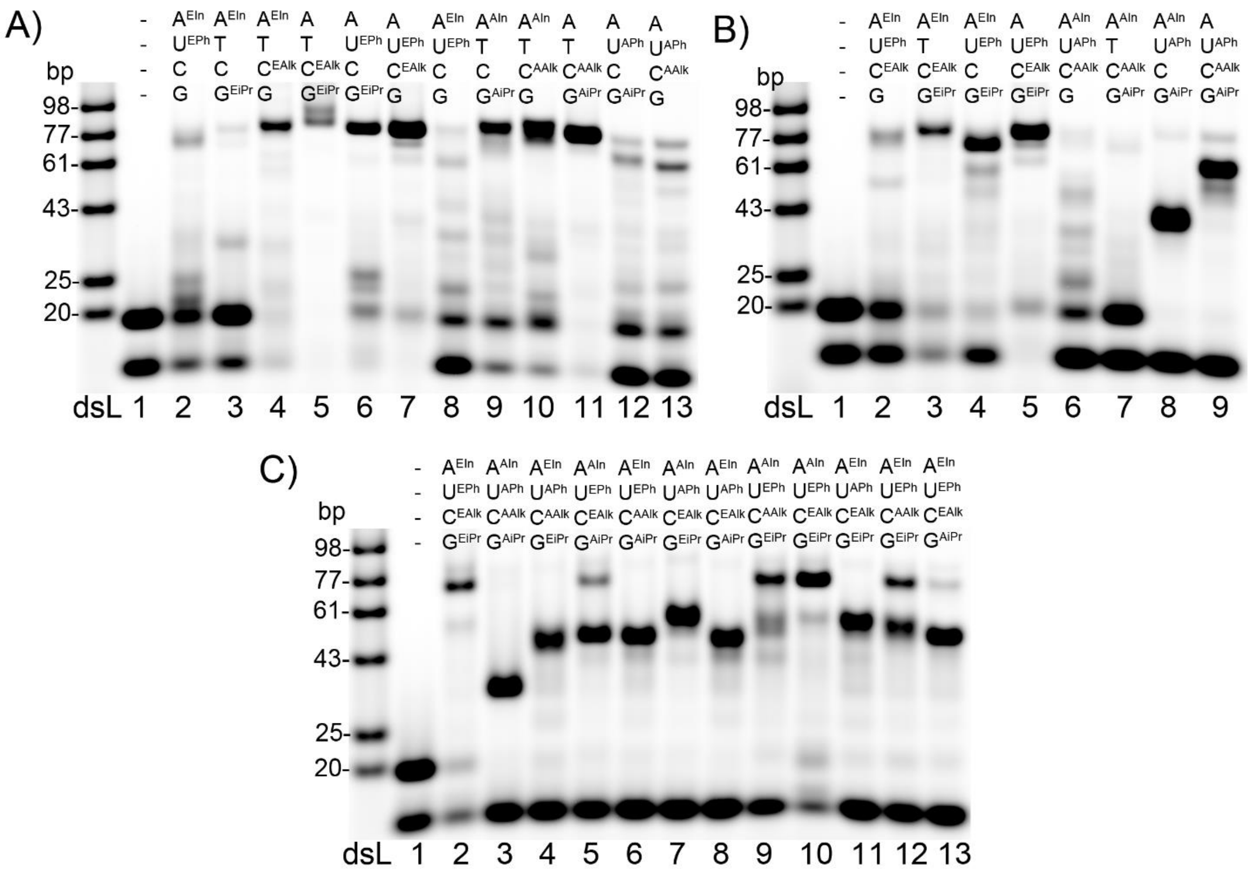

Figure S7. Agarose gels of various combinations of two (A), three (B) and four (C) modified **dN^{R}TP**s using 5´-(6-FAM)-labelled forward and reverse primer: (dsL), double-stranded ladder; (1) no dNTPs.

Method: Reaction mixture (20 µL) contained MO77 template (2.5 µM, 2 µL), 5´-Cy5-labelled L20 and 5´-(6-FAM)-labelled LT25TH primer (20 µM, 2 µL, each), combination of modified **dN^{R}TP**s of study (2 mM, 2 µL), Vent (exo-) DNA polymerase and reaction buffer (10X, 2 µL) as supplied by the manufacturer. All reaction mixtures were under cycling protocol: 95 °C for 1 min, followed by 50 cycles at 95 °C for 1 min, 50 °C for 1 min, and 70 °C for 2 min, followed by a final elongation step at 70 °C for 5 min. Amplified products were analysed on native agarose gel as well as denaturing PAGE and visualised using fluorescence imaging (Figure S8).

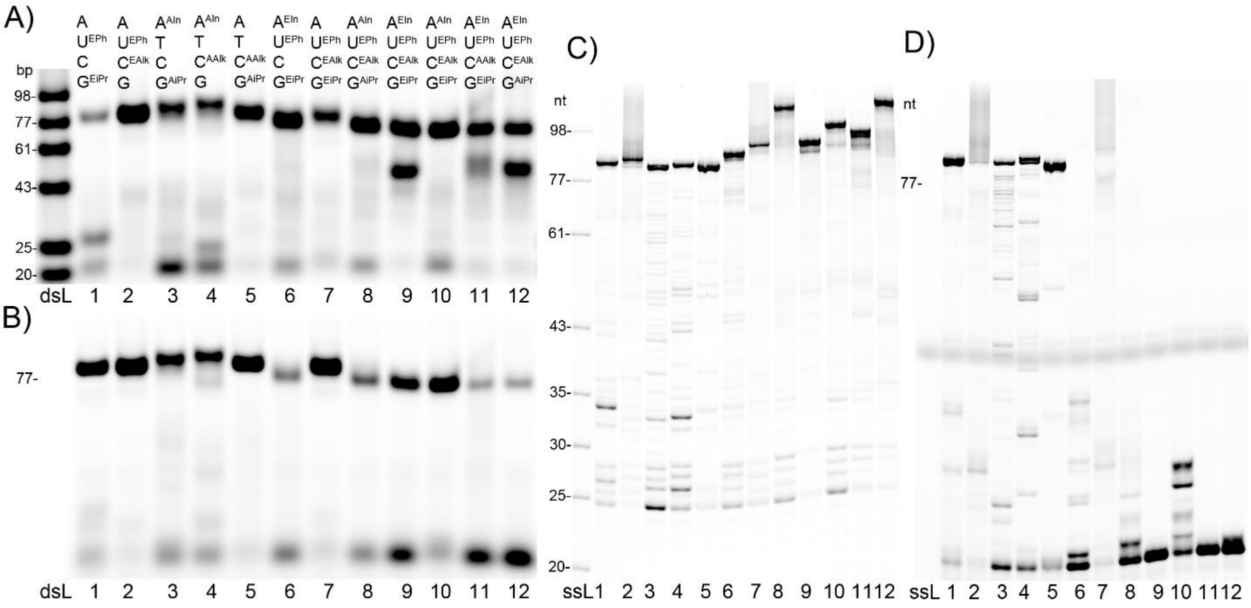

Figure S8. Full uncut native agarose gels (A,B) and denaturing PAGE (C,D) combinations of two (lanes 1-5), three (lanes 6-7) and four (lanes 8-12) modified **dN^{R}TP**s using 5´-(6-FAM)-labelled LT25TH reverse primer and 5´-Cy5-labelled L20 forward primer; (dsL) double-stranded ladder; (ssL) single-stranded ladder.

2.10 aPCR – multiple incorporation (four modified dN^{R}TPs)

aPCR using different template length

Method: Reaction mixture (20 µL) contained one of MO77 / FVL-A / MO120 / MO150 template (5 µM, 1 µL), 5´-(6-FAM)-labelled L20 and LT25TH primers (10 µM, 4 µL, each), ethynyl-modified **dN^{ER}TPs** (2 µL, Table S10), Vent (exo-) DNA polymerase (Table S10) and reaction buffer (10X, 2 µL) as supplied by the manufacturer. All positive controls contained 2.5 U of Vent (exo-) DNA polymerase and natural dNTPs (0.75 mM, 2 µL). All reaction mixtures were under cycling protocol: 95 °C for 1 min, followed by 50 cycles at 95 °C for 1 min, 50 °C for 1 min, and 70 °C for 2 min, followed by a final elongation step at 70 °C for 5 min. Samples were analysed on agarose gel as well as by PAGE and visualised using fluorescence imaging (Figure S9, S10).

Table S10. Reaction condition specifications for aPCR

Entry	Template	Vent (exo-)	**dN^{ER}TPs**	aPCR product
1	MO77	5 U	2 mM	**77ON_N^{ER}**
2	MO77OPP	5 U	2 mM	**77cON_N^{ER}**
3	FVL-A	5 U	4 mM	**98ON_N^{ER}**
4	MO120	10 U	4 mM	**120ON_N^{ER}**
5	MO150	10 U	4 mM	**150ON_N^{ER}**

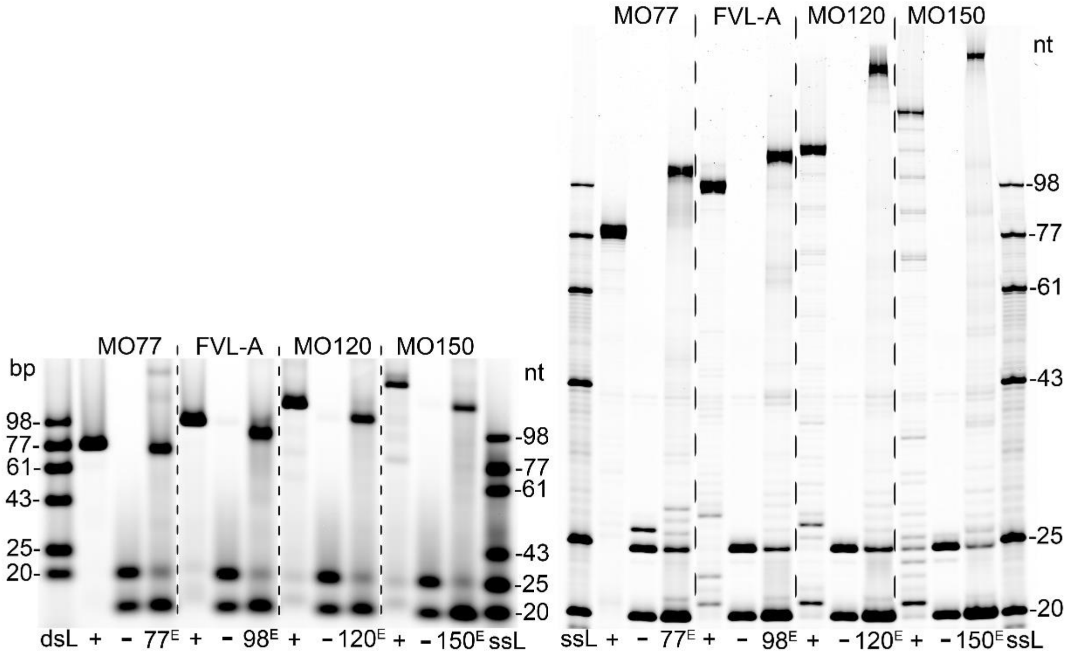

Figure S9. Agarose gel (left) and denaturing PAGE analysis (right) of aPCR reactions using ethynyl-modified **dN^{ER}TP**s, Vent (exo-) DNA polymerase and various templates: (+) natural dNTPs; (-) no dNTPs; (77^{E}) MO77, **dN^{ER}TP**s; (98^{E}) FVL-A, **dN^{ER}TP**s; (120^{E}) MO120, **dN^{ER}TP**s; (150^{E}) MO150, **dN^{ER}TP**s; (dsL) double-stranded ladder; (ssL) single-stranded ladder.

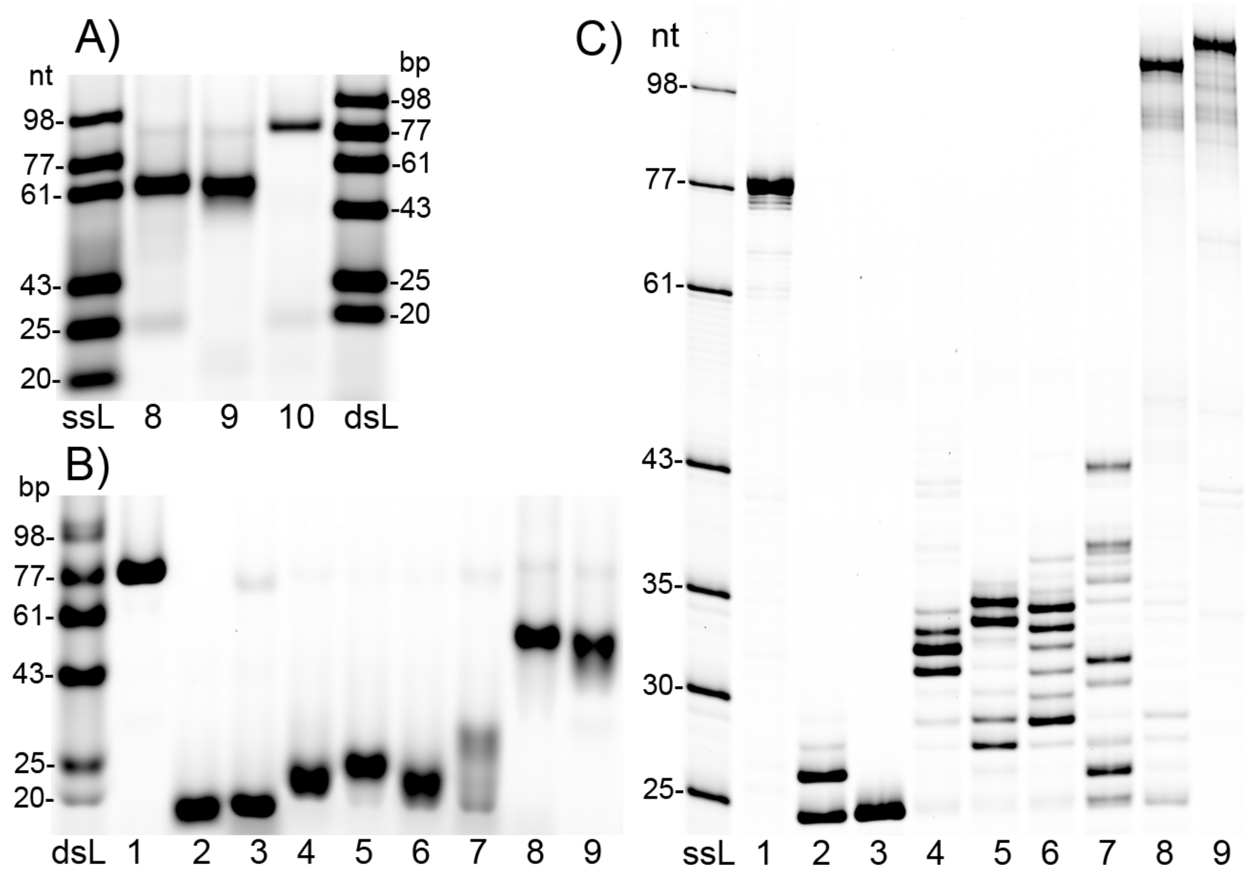

Figure S10. Agarose gels (A,B) and denaturing PAGE analysis (C) of PCR reactions using ethynyl-modified **dN^{ER}TP**s, Vent (exo-) DNA polymerase, MO77 template (lanes 1-8) or MO77OPP template (lane 9): (B,C): (1) natural dNTPs; (lanes 2-7) various negative controls: (2) no template; (3) no dNTPs; (4) natural dATP, TTP, dCTP; (5) natural dATP, TTP, dGTP; (6) natural dATP, dCTP, dGTP; (7) natural TTP, dCTP, dGTP; (8) **77ON_N^{ER}**; (9) **77cON_N^{ER}**; (10) **77DNA_dsN^{ER}** obtained by annealing of **77ON_N^{ER}** and **77cON_N^{ER}**; (dsL) double-stranded ladder; (ssL) single-stranded ladder.

aPCR using double-stranded template

Method: Reaction mixture (20 μL) contained MO77 and MO77OPP templates (2.5 μM, 2 μL, each), 5´-(6-FAM)-labelled L20 and 5´-Cy5-labelled LT25TH primer (20 μM, 2 μL, each), ethynyl-modified **dN^{ER}TPs** (2mM, 2 μL), Vent (exo-) DNA polymerase (5U) and reaction buffer (10X, 2 μL) as supplied by the manufacturer. All reaction mixtures were under cycling protocol: 95 °C for 1 min, followed by 50 cycles at 95 °C for 1 min, 50 °C for 1 min, and 70 °C for 2 min, followed by a final elongation step at 70 °C for 5 min.

Samples were analysed on denaturing PAGE and visualised using fluorescence imaging (Figure S11).

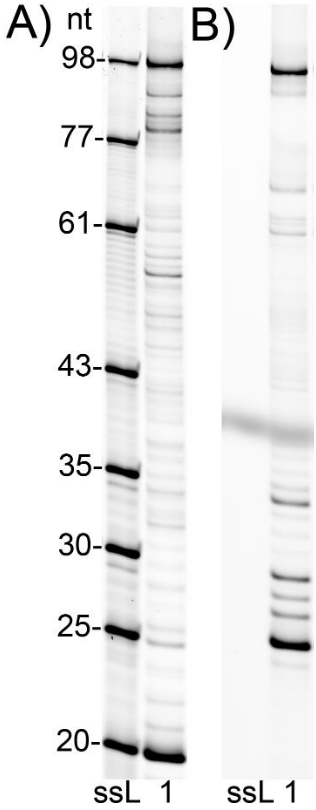

Figure S11. Denaturing PAGE of FAM-labelled **77cON_N^{ER}** (A) and Cy5-labelled **77ON_N^{ER}** (B) synthesized using non-modified MO77 and MO77OPP templates by PCR; (ssL) single-stranded ladder.

aPCR using different polymerase and different type of template

Method: Reaction mixture (20 μL) contained MO77 template (5 μM, 1 μL), 5´-(6-FAM)-labelled LT25TH primer (10 μM, 4 μL), ethynyl-modified **dN^{ER}TPs** (2 μL, Table S11), one of DNA polymerase (5 U, Table S11) and reaction buffer (10X, 2 μL) as supplied by the manufacturer. Since Vent (exo-) showed best results, conditions of this polymerase were applied for reactions with templates A77, T77, C77 and G77 to determine sequence dependency of this reaction. Amplified products were analysed on agarose gel and visualised using fluorescence imaging (Figure S12).

Table S11. Reaction condition specifications for aPCR

Entry / Lanes	template	**$dN^{ER}TPs$**	DNA polymerase	Amount of DNA polymerase	aPCR product
1	MO77	0.5 mM	KOD XL	5 U	**$77ON\_N^{ER}$**
2	MO77	2 mM	LongAmp	5 U	**$77ON\_N^{ER}$**
3	MO77	2 mM	Pwo	5 U	**$77ON\_N^{ER}$**
4	MO77	2 mM	Deep Vent (exo-)	5 U	**$77ON\_N^{ER}$**
5	MO77	2 mM	Vent (exo-)	5 U	**$77ON\_N^{ER}$**
6	A77	2 mM	Vent (exo-)	5 U	**$77ON\_U\_N^{ER}$**
7	T77	2 mM	Vent (exo-)	5 U	**$77ON\_A\_N^{ER}$**
8	C77	2 mM	Vent (exo-)	5 U	**$77ON\_G\_N^{ER}$**
9	G77	2 mM	Vent (exo-)	5 U	**$77ON\_C\_N^{ER}$**

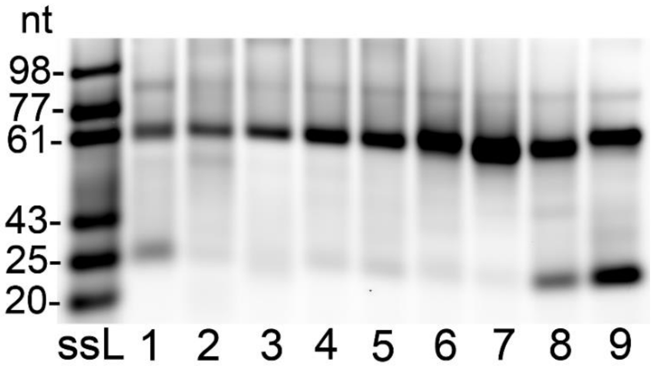

Figure S12. Agarose gel of aPCR reaction with ethynyl-modified **$dN^{ER}TP$**s using different DNA polymerases: (1) KOD XL; (2) LongAmp; (3) Pwo; (4) Deep Vent (exo-); (5-9) Vent (exo-); and different templates: (5) MO77; (6) A77; (7) T77; (8) C77; (9) G77; (ssL) single-stranded ladder.

2.11 Re-PCR of modified ssONs

aPCR scale-up and HPLC purification: In order to obtain sufficient amount of ethynyl-modified **$97ON\_N^{ER}$** for following re-PCR, aPCR reaction was scaled-up (50 reactions) using MO77 template having 3´-sC3 modification, Flank_LT25TH primer, Vent (exo-) DNA polymerase and ethynyl-modified **$dN^{ER}TPs$** under reaction conditions as described in section 2.9. Purification was performed by HPLC using Waters X Bridge column (0.6 mL/min, column heated for 60°C) with use of linear gradient starting from 0.1 M TEAB in H_2O continuing to 0.1 M TEAB in H_2O/AN (4:1) and to AN and followed by freeze-drying.

Re-PCR method: Reaction mixture (20 μL) contained modified **97ON_N^{ER}** as template (7.5 μM, 1 μL), 5´-(6-FAM)-labelled L20 and Flank primers (10 μM, 4 μL, each), natural dNTPs (2 μL, 4 mM), Vent (exo-) DNA polymerase (6 U) and reaction buffer (10X, 2 μL) as supplied by the manufacturer. Reaction mixtures were under cycling protocol: 95 °C for 1 min, followed by 30 cycles at 95 °C for 1 min, 55 °C for 1 min, and 70 °C for 2 min, followed by a final elongation step at 70 °C for 5 min. Samples were analysed on agarose gel as well as by PAGE and visualised using fluorescence imaging (Figure S13).

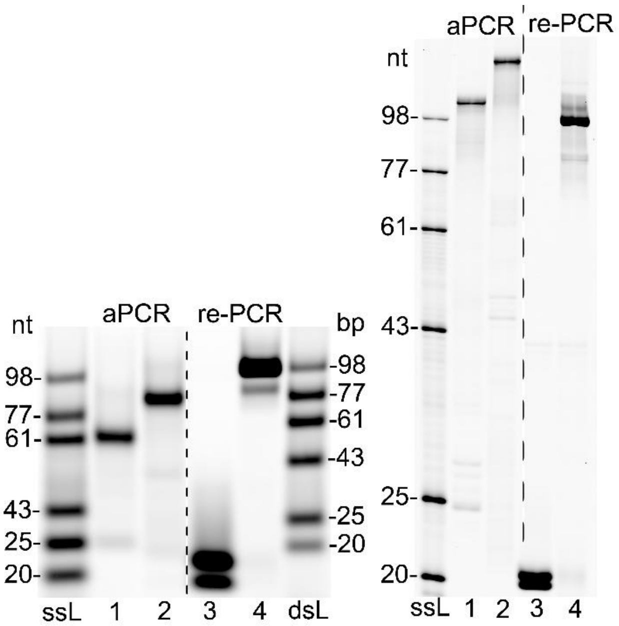

Figure S13. Agarose gel (left) and denaturing PAGE analysis (right) showing aPCR and re-PCR reactions: (1) **77ON_N^{ER}**; (2) **97ON_N^{ER};** (3) negative control, no dNTPs; (4) re-PCR of **97ON_N^{ER}**, natural dNTPs; (dsL) double-stranded ladder; (ssL) single-stranded ladder.

2.12 Sanger sequencing

In order to prepare dsDNA for Sanger sequencing, re-PCR of modified non-labelled **97ON_N^{ER}** was performed with L20 and Flank primers using reaction conditions as described in section 2.10. Resulting natural 97bp DNA was purified by Qiaquick PCR purification kit. dsDNA (50 ng/μL) was sent for Sanger sequencing using L20_Seq+ and Flank_Seq+ (5 μL, 5 μM, each) in order to improve sequencing results of such short DNA (Figure S14, S15).

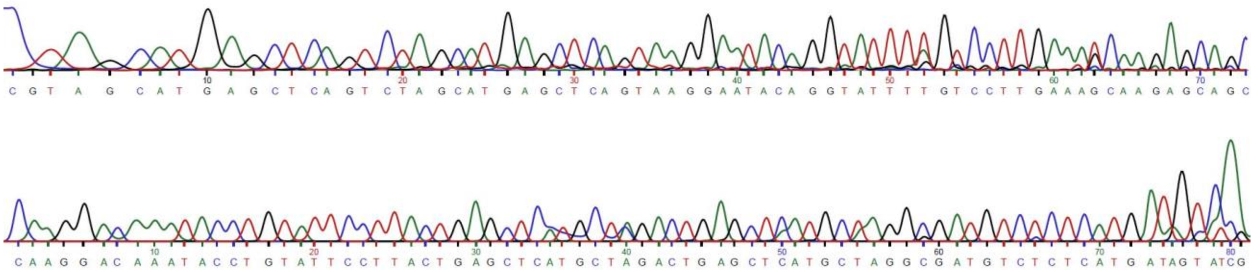

Figure S14. Raw data of Sanger sequencing of 97bp DNA (both strands) generated from modified **97ON_N^{ER}**.

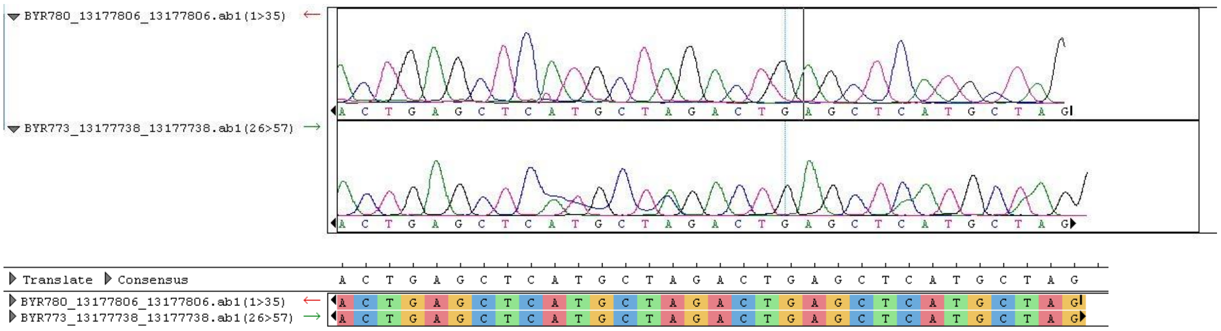

Figure S15. Alignment analysis of target sequence (modified region excluding primer sequences) obtained by Sanger sequencing of the 97bp DNA product generated from modified **97ON_N^{ER}**.

2.13 High-throughput Next-generation Sequencing (NGS)

NGS was performed using Nextera XT 2 Mid-Output cartridge and MiniSeq sequencing system (Illumina). In order to prepare dsDNA for sequencing, first, re-PCR of **97ON_N^{ER}** was performed to introduce adapter and then index sequences under the following protocols:

PCR with adapter primers: Reaction mixture (25 µL) contained modified non-labelled single-stranded **97ON_N^{ER}** (50 nM, 2 µL), L20_adapter and Flank_adapter primers (10 µM, 0.75 µL, each) and 2X KAPA HiFi Hotstart Master Mix (12.5 µL) as supplied by the manufacturer. The PCR reaction was performed according to the cycling protocol: 95 °C for 3 min, followed by 32 cycles at 98 °C for 20 sec, 64 °C for 15 sec, and 72 °C for 15 sec, followed by a final elongation step at 72 °C for 1 min. The resulting 164bp PCR

product was purified with AMPure XP magnetic beads using the protocol provided by the manufacturer, analysed by 2% agarose gel electrophoresis and visualized by GelRed staining (Figure S16).

PCR with index primers: Reaction mixture (25 µL) contained purified dsDNA from previous PCR step (5 µL, no quantification), Nextera F_index and R_index primers (10 µM, 2.5 µL, each) and 2X KAPA HiFi Hotstart Master Mix (12.5 µL) as supplied by the manufacturer. The PCR reaction was performed according to the cycling protocol: 95 °C for 3 min, followed by 8 cycles at 98 °C for 20 sec, 64 °C for 15 sec, and 72 °C for 15 sec, followed by a final elongation step at 72 °C for 1 min. The resulting 262bp PCR product was purified with AMPure XP magnetic beads using the protocol provided by the manufacturer and analysed by 2% agarose gel electrophoresis (Figure S16). The obtained dsDNA was quantified by qPCR, normalized to a final 10nM concentration and sequenced.

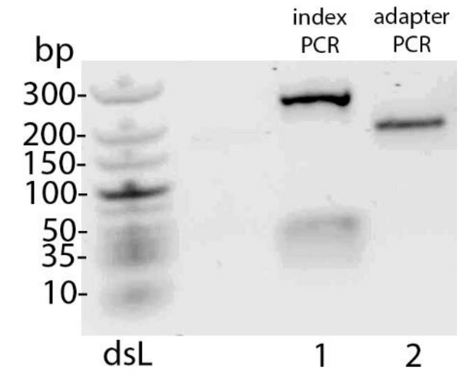

Figure S16. Agarose gel electrophoresis of PCR with adapter (lane 2) and index (lane 1) primers stained with GelRed.

Sequencing analysis was performed on Illumina MiniSeq (2×150 bp, paired-end reads). Raw data were processed with the Trimmomatic 0.36 tool (http://www.usadellab.org/cms/?page=trimmomatic) by means of quality trimming and filtering, paired-end reads were merged using fastq-join algorithm (https://github.com/brwnj/fastq-join). Sequencing was set up for two independent clustering resulting in two independent outputs of the same sample. Results are shown

as an alignment of unique sequences with frequency in 833 and 1048 total analyzed reads (Figure S17).

frequency	**ACTGAGCTCATGCTAGACTGAGCTCATGCTAG<u>GCGATGTCTCTCATGATGTC</u>**
81.03451 %	ACTGAGCTCATGCTAGACTGAGCTCATGCTAGGCGATGTCTCTCATGATGTC
1.477830 %	ACTGAGCTTATGCTAGACTGAGCTCATGCTAGGCGATGTCTCTCATGATGTC
0.738916 %	ACTGAGCTCATGCTAGACTGAGCTCATGC-AGGCGATGTCTCTCATGATGTC
0.738916 %	ACTGAGCTCATGCTAGACTGAGCTCATGCAAGGCGATGTCTCTCATGATGTC
0.615764 %	ACTGAGC-CATGCTAGACTGAGCTCATGCTAGGCGATGTCTCTCATGATGTC
0.492611 %	ACTGAGCTCATGCTAGACTGAGCTTATGCTAGGCGATGTCTCTCATGATGTC
0.492611 %	ACTGAGCTCATGCTAGACTGAGCTCATACTAGGCGATGTCTCTCATGATGTC
0.492611 %	ACTGAGCTCATGCTAGACTAAGCTCATGCTAGGCGATGTCTCTCATGATGTC
0.492611 %	ACTGAGCTAATGCTAGACTGAGCTCATGCTAGGCGATGTCTCTCATGATGTC
0.246305 %	ATTGAGCTCATGCTAGACTAAGCTCATGCTAGGCGATGTCTCTCATGATGTC
0.246305 %	ACTGAGCTCATG-TAGACTGAGCTCATGCTAGGCGATGTCTCTCATGATGTC
0.246305 %	ACTGAGCTCATGCTAGTCTGAGCTCATGCTAGGCGATGTCTCTCATGATGTC
0.246305 %	ACTGAGCTCATGCTAGACTG-GCTCATGCTAGGCGATGTCTCTCATGATGTC
0.246305 %	ACTGAGCTCATGCTAGACTGAGCT~~C~~CATGCTAGGCGATGTCTCTCATGATGTC
0.246305 %	ACTGAGCTCATGCTAGACTGAGCTCATGCTAG-CGATGTCTCTCATGATGTC
0.246305 %	ACTGAGCTCATGCTAGACTGAGCTCATGCTAAGCGATGTCTCTCATGATGTC
0.246305 %	ACTGAGCTCATGCTAGACTGAGC--ATGCTAGGCGATGTCTCTCATGATGTC
0.246305 %	ACTGAGCTCATGCTAGACTGAGCACATGCTAGGCGATGTCTCTCATGATGTC
0.246305 %	ACTGAGCTCATGCTAGACTGA-CTCATGCTAGGCGATGTCTCTCATGATGTC
0.246305 %	ACTGAGCCAATGCTAGACTGAGCTCATGCTAGGCGATGTCTCTCATGATGTC
0.246305 %	ACTGAGCACATGCTAGACTGAGCTCATGCTAGGCGATGTCTCTCATGATGTC

frequency	**ACTGAGCTCATGCTAGACTGAGCTCATGCTAG<u>GCGATGTCTCTCATGATGTC</u>**
75.90950 %	ACTGAGCTCATGCTAGACTGAGCTCATGCTAGGCGATGTCTCTCATGATGTC
1.376600 %	ACTGAGCTTATGCTAGACTGAGCTCATGCTAGGCGATGTCTCTCATGATGTC
0.983284 %	ACTGAGCTCATGCTAGACTGAGCTCATGCAAGGCGATGTCTCTCATGATGTC
0.983284 %	ACTGAGCTCATGCTAGACTGAGCACATGCTAGGCGATGTCTCTCATGATGTC
0.884956 %	ACTGAGCTCATGCTAGACTGAGCTCATGCTAAGCGATGTCTCTCATGATGTC
0.884956 %	ACTGAGCTAATGCTAGACTGAGCTCATGCTAGGCGATGTCTCTCATGATGTC
0.688299 %	ACTGAGCTCATGCTAAACTGAGCTCATGCTAGGCGATGTCTCTCATGATGTC
0.688299 %	ACTGAGCT-ATGCTAGACTGAGCTCATGCTAGGCGATGTCTCTCATGATGTC
0.589971 %	GCTGAGCTCATGCTAGACTGAGCTCATGCTAGGCGATGTCTCTCATGATGTC
0.589971 %	ACTGAGCTCATGCTAGACTGAGCTCATGC-AGGCGATGTCTCTCATGATGTC
0.589971 %	ACT-AGCTCATGCTAGACTGAGCTCATGCTAGGCGATGTCTCTCATGATGTC
0.393314 %	ACTGAGCTCATGCTAGACTGAGCTTATGCTAGGCGATGTCTCTCATGATGTC
0.393314 %	ACTGAGCTCATGCTAGACTAAGCTCATGCTAGGCGATGTCTCTCATGATGTC
0.393314 %	ACTGAGCACATGCTAGACTGAGCTCATGCTAGGCGATGTCTCTCATGATGTC
0.294985 %	ACTGAGCTC-TGCTAGACTGAGCTCATGCTAGGCGATGTCTCTCATGATGTC
0.294985 %	ACTGAGCTCATGCTATACTGAGCTCATGCTAGGCGATGTCTCTCATGATGTC
0.294985 %	ACTGAGCTCATGCTAGACTGAACTCATGCTAGGCGATGTCTCTCATGATGTC
0.294985 %	ACTGAGCTCATGCTAGACAAAGCTCATGCTAGGCGATGTCTCTCATGATGTC
0.294985 %	ACTGAGCTCAT-CTAGACTGAGCTCATGCTAGGCGATGTCTCTCATGATGTC
0.294985 %	ACTGAGC-CATGCTAGACTGAGCTCATGCTAGGCGATGTCTCTCATGATGTC
0.294985 %	ACTGAACTCATGCTAGACTGAGCTCATGCTAGGCGATGTCTCTCATGATGTC
0.294985 %	ACCGAGCTCATGCTAGACTGAGCTCATGCTAGGCGATGTCTCTCATGATGTC

Figure S17. Alignment of the most abundant (frequency over 0.2%) sequences obtained from NGS sequencing of PCR products generated from modified **97ON_N^{ER}**. First columns represent frequency of each unique sequence in 833 and 1048 reads, second column shows aligned sequences. Differences (point mutations) to original target sequence (in bold) are shown in red. Primer region is underlined.

2.14 NEAR - Nicking Enzyme Amplification Reaction

NEAR method: Reaction mixture (50 µL) contained NickMO1 template (5 µM, 1.25 µL), PrimNick primer (5 µM, 1.25 µL), set of four modified **dN^{ER}TPs** (2 mM, 4 µL), Vent (exo-) DNA polymerase (7 U), Nt.BstNBI Nickase (30 U), DNA polymerase reaction buffer (10X, 5 µL) and Nickase reaction buffer (10X, 2.5 µL) as supplied by the manufacturers. The reaction mixture was incubated for 3 h at 37 °C and stopped by cooling down to 4 °C. NEAR reaction mixture was purified using Illustra MicroSpin G-25 Columns (GE Healthcare) and directly used for radiolabeling.

Radiolabeling method: Reaction mixture (50 µL) contained filtrate obtained from NEAR reaction (40 µL), T4 Polynucleotide Kinase (20 U), [γ-^{32}P]-ATP (2 µL, 250 µCi, 9.25 MBq) and reaction buffer (10X, 5 µL) as supplied by the manufacturer. The reaction mixture was incubated for 1 h at 37 °C and stopped by cooling down to 4 °C. Samples were analysed by PAGE. The gel was dried (85°C, 50 min), autoradiographed and visualised by phosphorimaging. NEAR reaction resulted in **17ON_N^{ER}** instead of **16ON_N^{ER}**, since additional modified **dA^{EIn}TP** was added in untemplated fashion (Figure S18, S51).

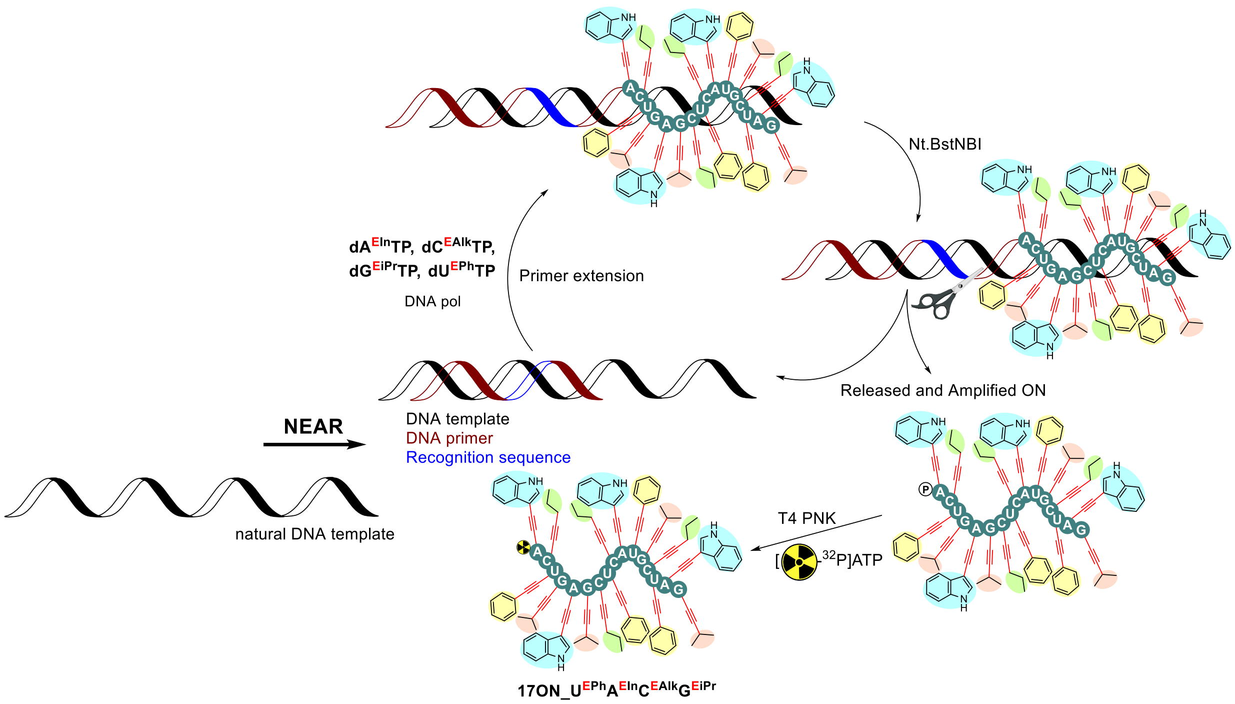

Scheme S5. Nicking enzyme amplification reaction (NEAR)

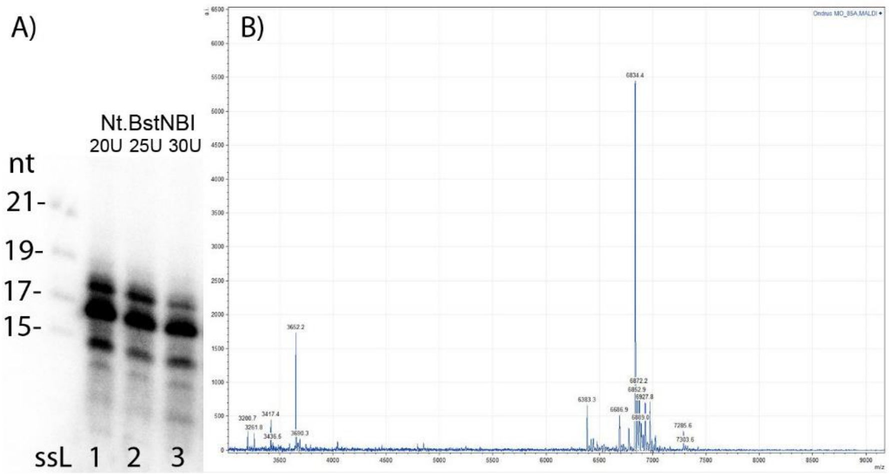

Figure S18. A) Denaturing PAGE analysis of NEAR reaction with modified **dN^{ER}TPs**: (ssL), single-stranded ladder, (1) **17ON_N^{ER}**; (2) **17ON_N^{ER}**; (3) **17ON_N^{ER}**; B) MALDI-TOF measurement of full-length **17ON_N^{ER}** NEAR product (Figure S51).

3) Experimental section – UV-VIS absorption and CD spectroscopy

UV-absorption measurements were performed on Cary 100 Bio UV/VIS Spectrophotometer with temperature controller (Varian). The spectra were recorded in 1 mm rectangular quartz cell, in temperature range 25 °C - 95 °C with temperature increment 1 °C / min under 260 nm detection and were obtained from three cycles (6 ramps in total). T_m values (in °C) were calculated using first negative derivative of intensity over temperature.

The circular dichroism (CD) measurements were performed on a Jasco-1500 spectropolarimeter equipped with Peltier thermostated holder PTC-517 (JASCO Inc. Easton, MD, USA). The spectra were recorded in temperature range 5 °C – 95 °C with temperature increment 5 °C in spectral range from 200 nm to 400 nm in 1 mm rectangular quartz cell with following experimental setup: standard instrument sensitivity, 1 nm bandwidth, a scanning speed of 10 nm/min, a response time of 8 s and one accumulation. The temperature of the sample was kept constant during each data accumulation and the same experimental setup was used for temperature increase and decrease. After baseline substraction the final data were recalculated on the concentration of nucleotides

and expressed as molar differential extinction Δε ($cm^{-1}mol^{-1}$). The melting temperatures were calculated using program Sigmaplot 12.5 (Systat software) when sigmoid fitting was applied.

In order to obtain sufficient amount of ethynyl- and alkyl-modified DNA to measure absorption, CD spectra and T_m values, synthesis of 31bp DNA (**31DNA** or **31DNA_N^{ER}** or **31DNA_N^{AR}**) was scaled-up by 100 PEX reactions using prb4basII template with natural and modified **dN^{ER}TPs** / **dN^{AR}TPs** as described in section 2.3. PEX products were purified by Qiaquick nucleotide removal kit (Qiagen) and diluted to 2 μM final concentration by TrisHCl buffer (10 mM, 1 mM EDTA, 65 mM NaCl, pH 8.0) and set for measurement (Figure 5A-B, S19).

In order to obtain ethynyl-modified **77DNA_N^{ER}**, aPCR reaction was scaled-up by 50 aPCR reactions using MO77 template and modified **dN^{ER}TPs** as described in section 2.9. aPCR product, **77ON_N^{ER}**, was annealed to its complementary sequence (30 pmol of MO77 for each 20 μL of aPCR reaction) directly in Vent (exo-) DNA polymerase buffer (Thermopol buffer). **77DNA_N^{ER}** was purified by Qiaquick PCR purification kit (Qiagen) using protocol provided by the manufacturer, dissolved in TrisHCl buffer (10 mM, 1 mM EDTA, 65 mM NaCl, pH 8.0) to 2 μM final concentration and set for T_m and CD measurements (Figure 5C-D, S19). Natural **77DNA** was prepeared using MO77 template with conditions describes for positive controls in section 2.9. Obtained DNA was purified by Qiaquick PCR purification kit (Qiagen) and dissolved in TrisHCl buffer (10 mM, 1 mM EDTA, 65 mM NaCl, pH 8.0) to 2 μM final concentration.

3.1 UV-VIS spectroscopy

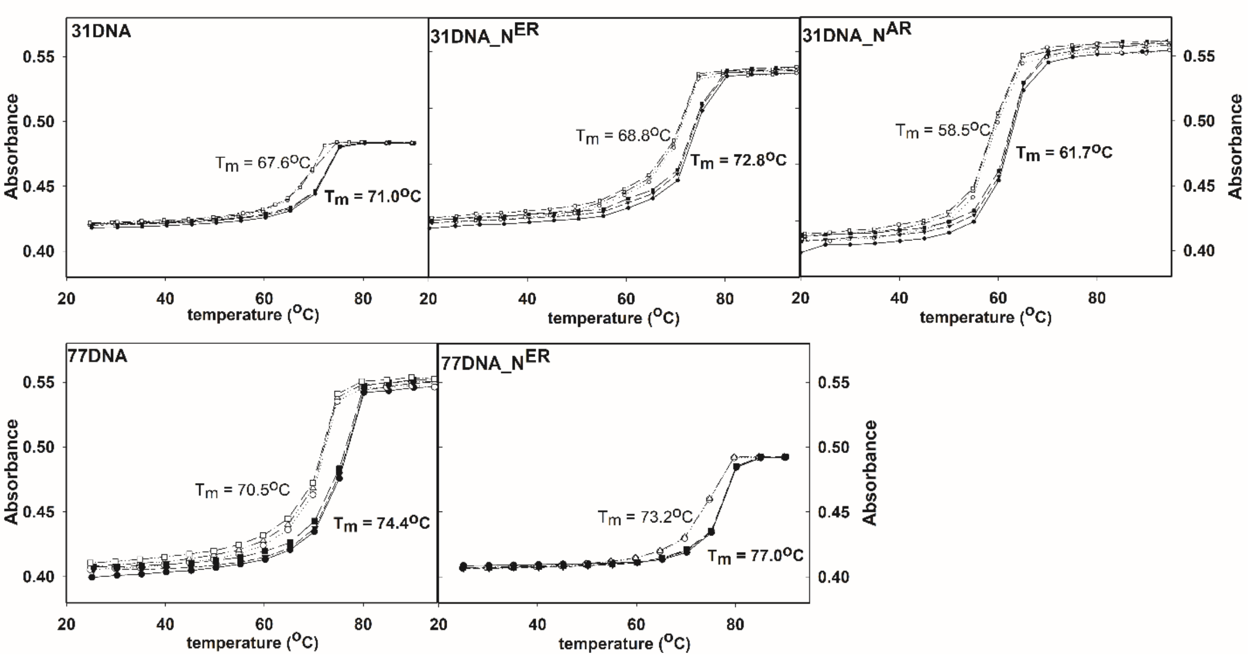

Figure S19. Melting curves with calculated melting temperatures (T_m) of **31DNA**, **31DNA_N^{ER}**, **31DNA_N^{AR}** and **77DNA**, **77DNA_N^{ER}** obtained from UV spectroscopy at 260 nm absorption.

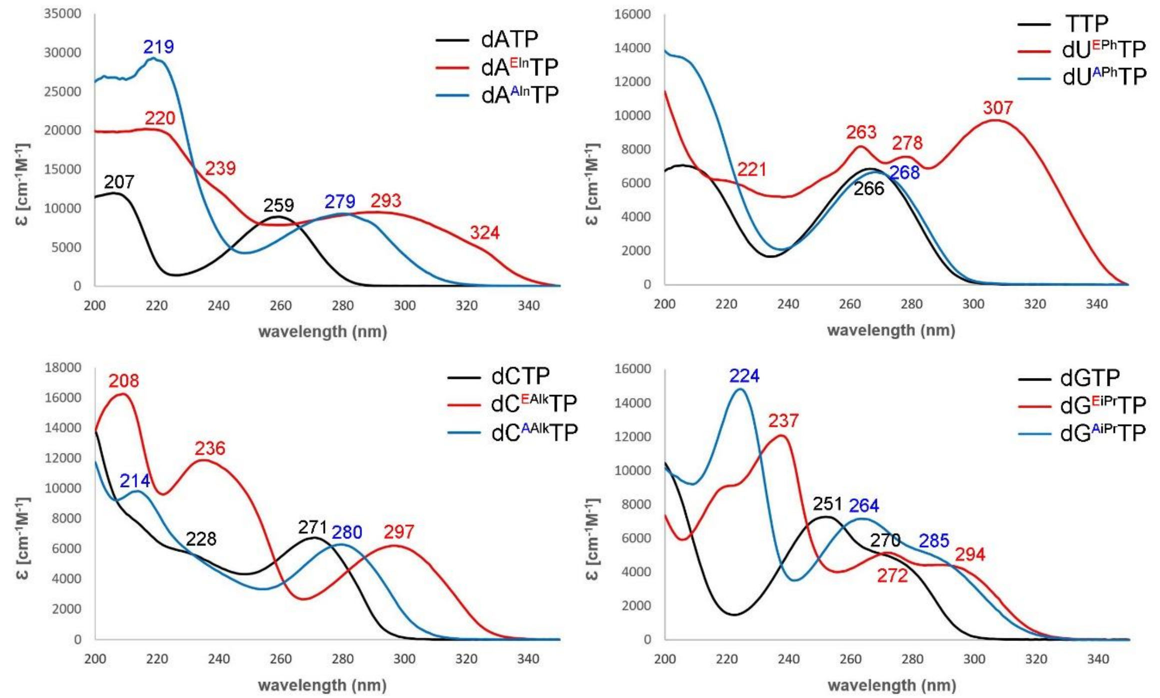

Figure S20. UV absorption spectra of natural and modified dNTP monomers recorded by UV-spectrophotometer in 200-350 nm range (in water at concentration 0.1 μM).

3.2 Circular dichroism (CD) spectroscopy

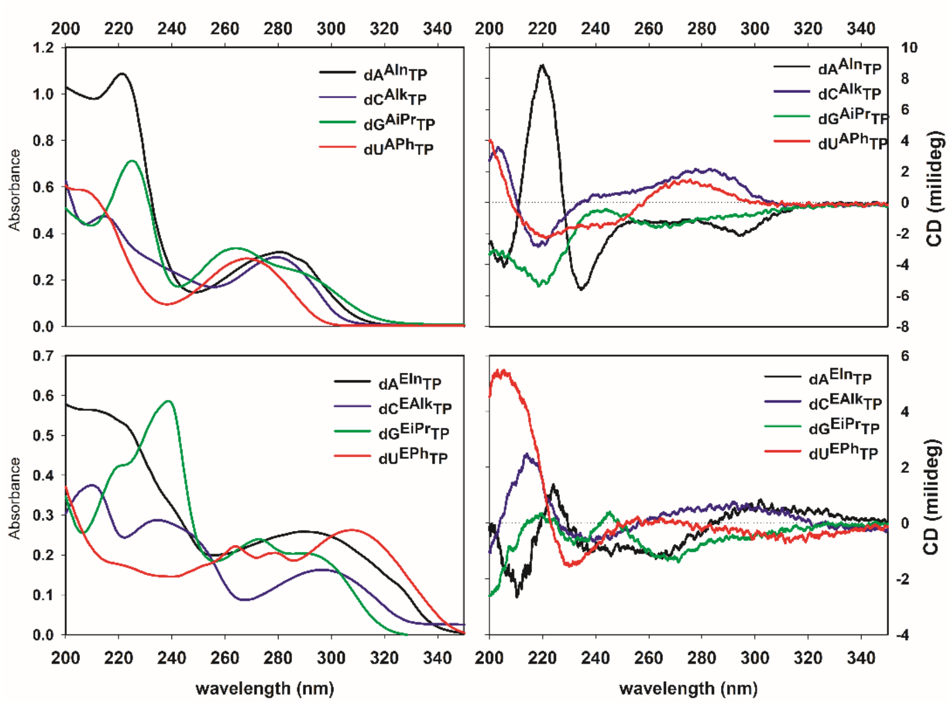

Figure S21. UV absorption and CD spectra of natural and modified dNTP monomers in 200-350nm range (in water at concentration 0.1 μM).

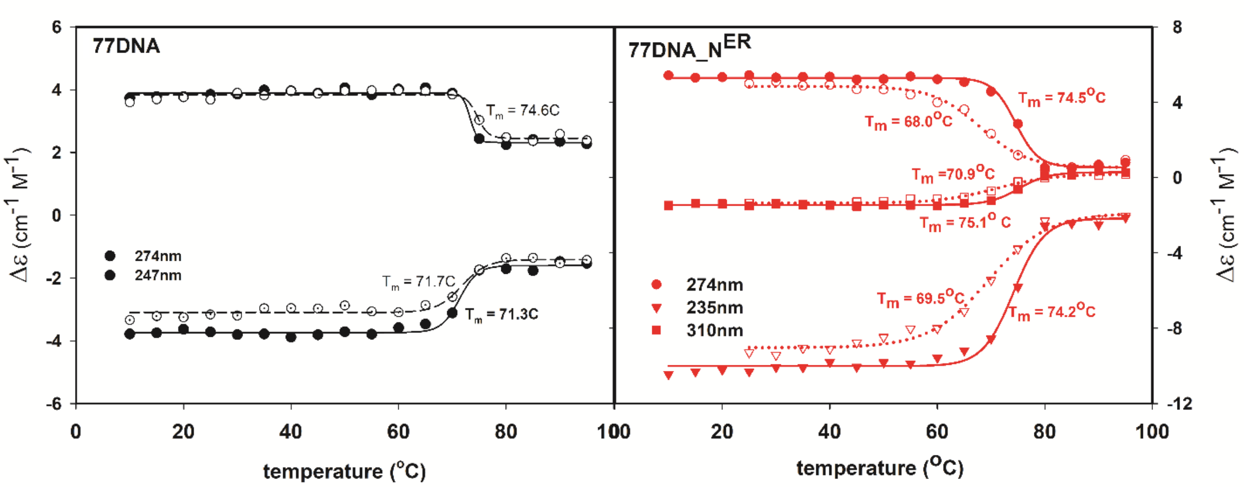

Figure S22. Melting curves with calculated melting temperatures (T_m) of **77DNA** and **77DNA_N^{ER}** obtained from CD spectroscopy under particular wavelengths.

4) Copies of MALDI-TOF mass spectra

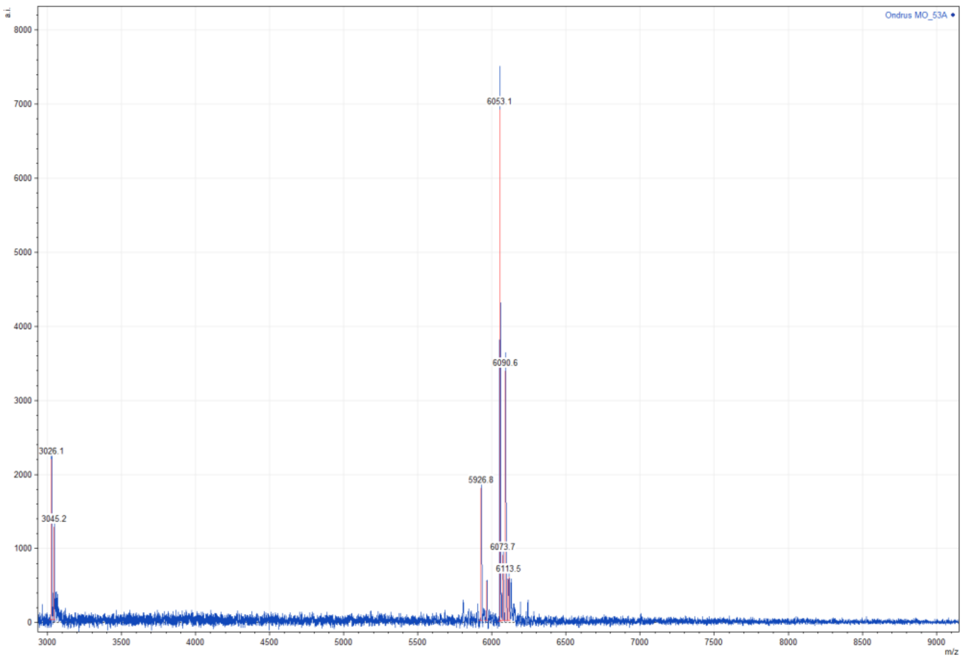

Figure S23. MALDI-TOF spectra of **19ON_U$^{\mathbf{EPh}}$**: calculated: 6051.9 Da; found: 6053.1 Da; Δ = 1.2.

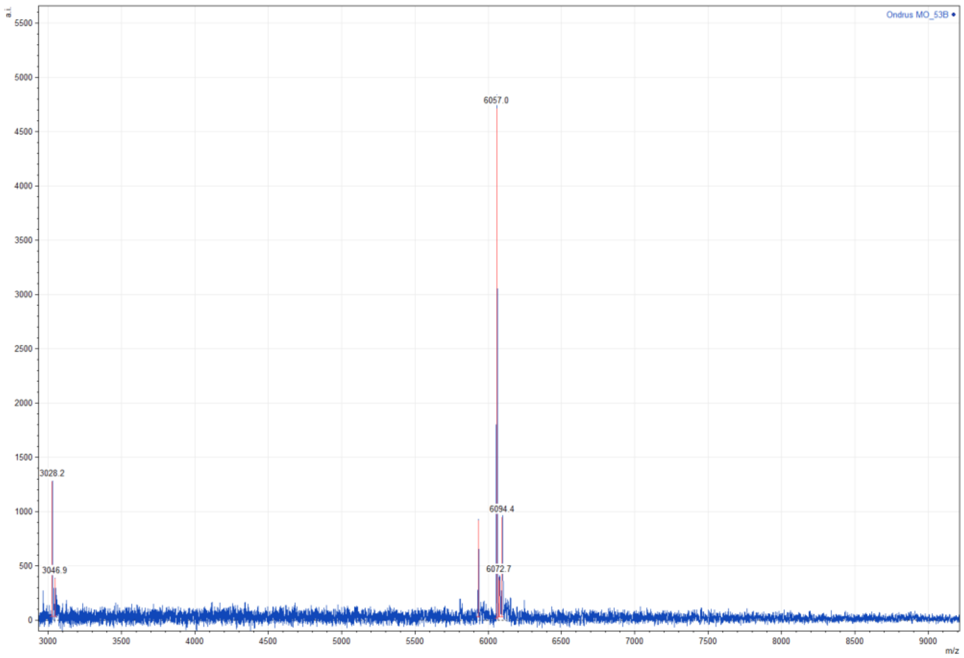

Figure S24. MALDI-TOF spectra of **19ON_U^{APh}**: calculated: 6055.9 Da; found: 6057.0 Da; Δ = 1.1.

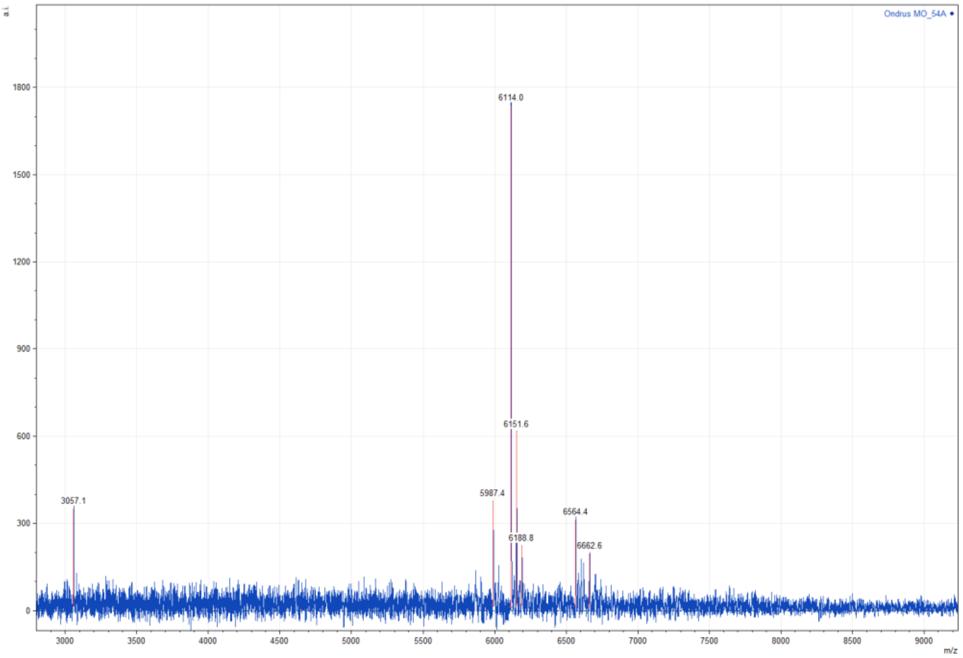

Figure S25. MALDI-TOF spectra of **19ON_A^{EIn}**: calculated: 6113.1 Da; found: 6114.0 Da; Δ = 1.1.

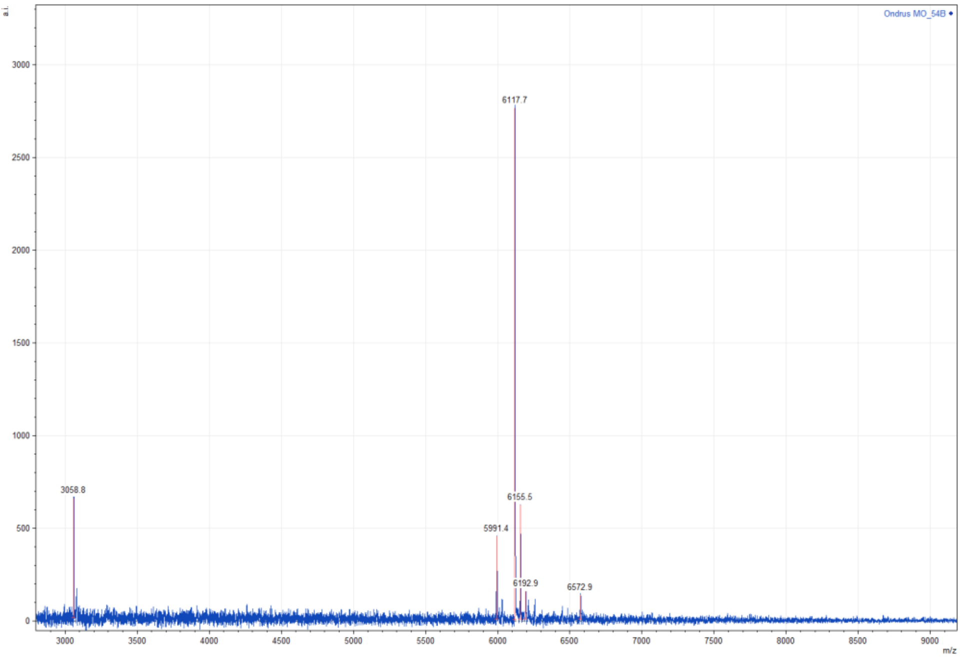

Figure S26. MALDI-TOF spectra of **19ON_A^{AIn}**: calculated: 6116.9 Da; found: 6117.7 Da; Δ = 0.8.

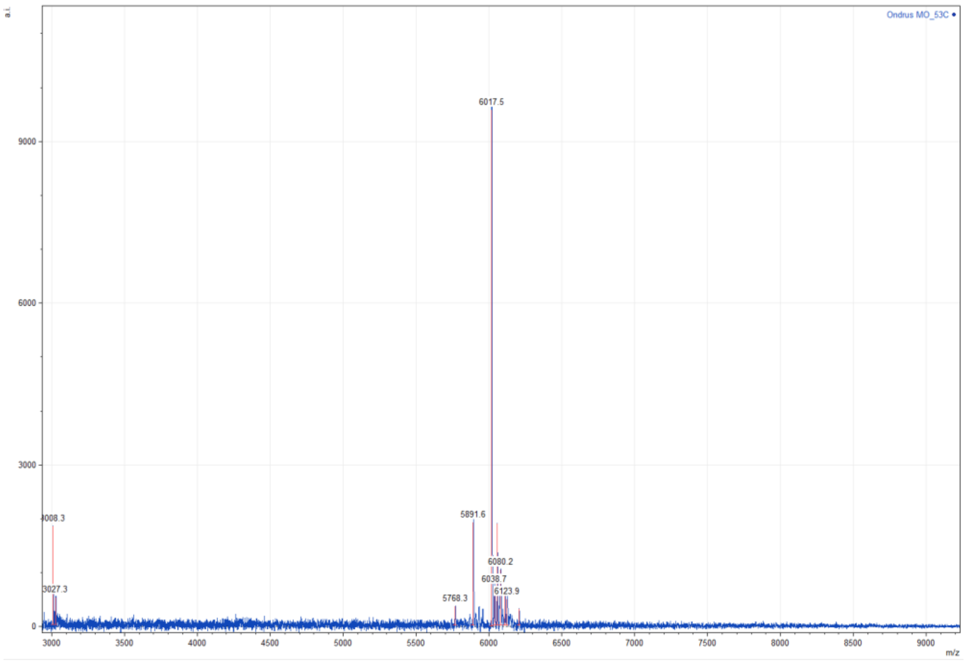

Figure S27. MALDI-TOF spectra of **19ON_C$^{\mathbf{EAlk}}$**: calculated: 6016.9 Da; found: 6017.5 Da; Δ = 0.6.

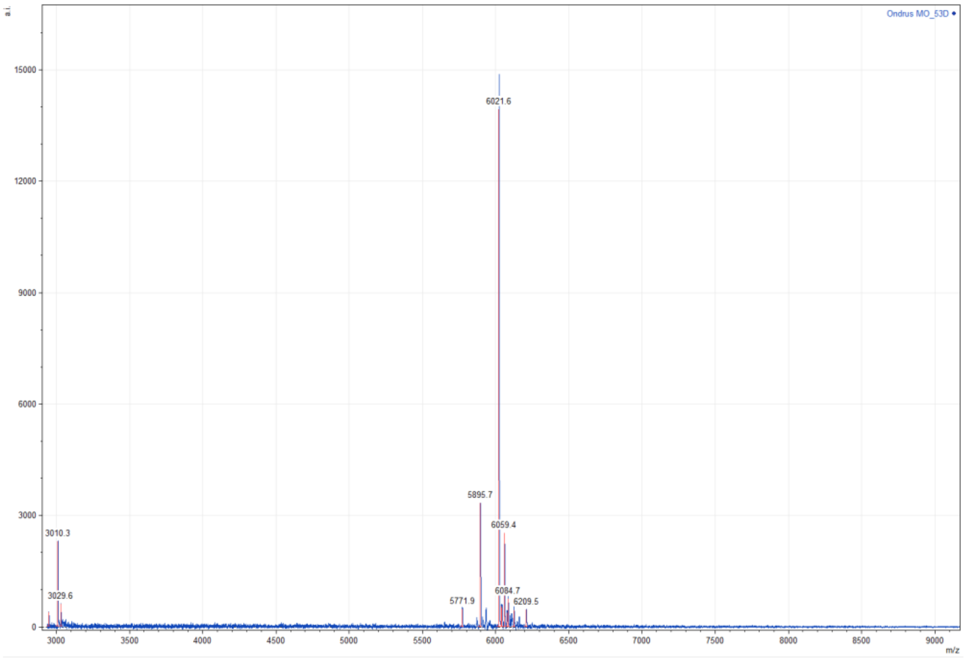

Figure S28. MALDI-TOF spectra of **19ON_C$^{\mathbf{AAlk}}$**: calculated: 6021.0 Da; found: 6021.6 Da; Δ = 0.6.

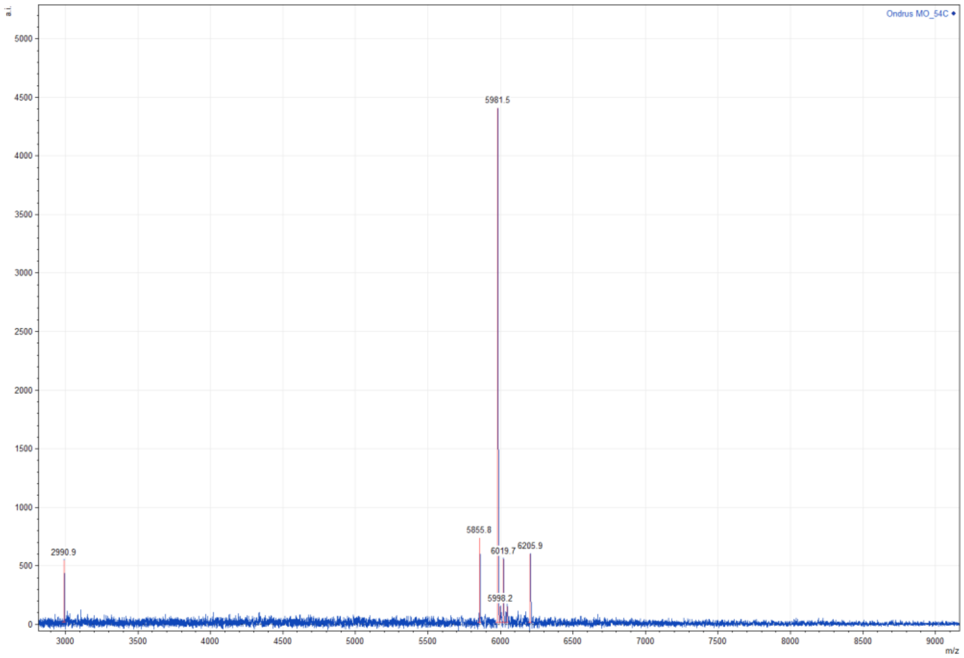

Figure S29. MALDI-TOF spectra of **19ON_G**$^{\mathbf{EiPr}}$: calculated: 5981.0 Da; found: 5981.5 Da; Δ = 0.5.

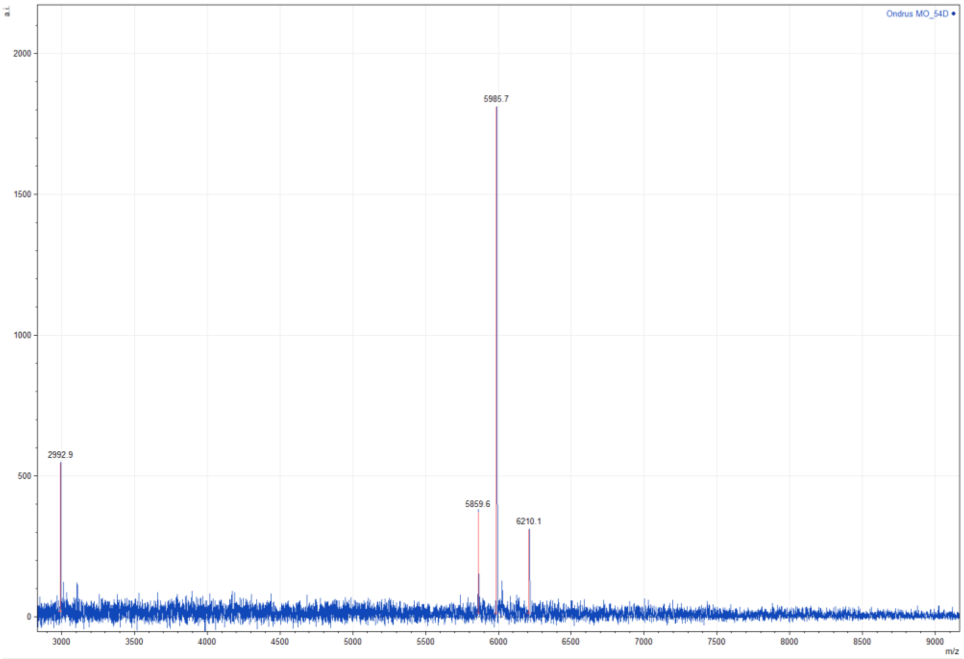

Figure S30. MALDI-TOF spectra of **19ON_G**$^{\mathbf{AiPr}}$: calculated: 5985.1 Da; found: 5985.7 Da; Δ = 0.6.

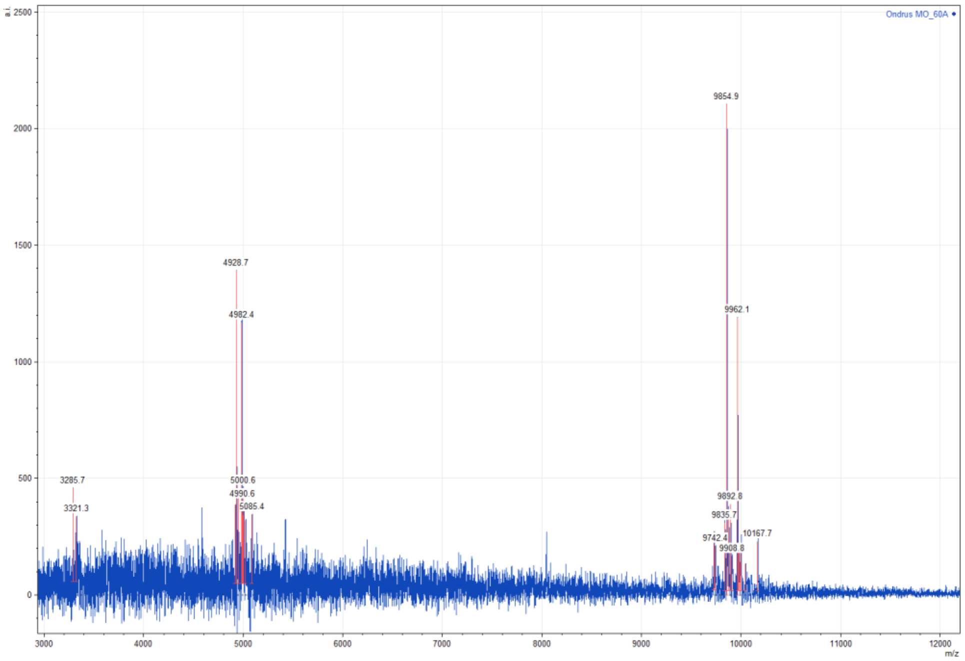

Figure S31. MALDI-TOF spectra of **31ON_U^{EPh}**: calculated: 9961.3 Da; found: 9962.1 Da; Δ = 0.8 (M = 9854.9 Da is template strand).

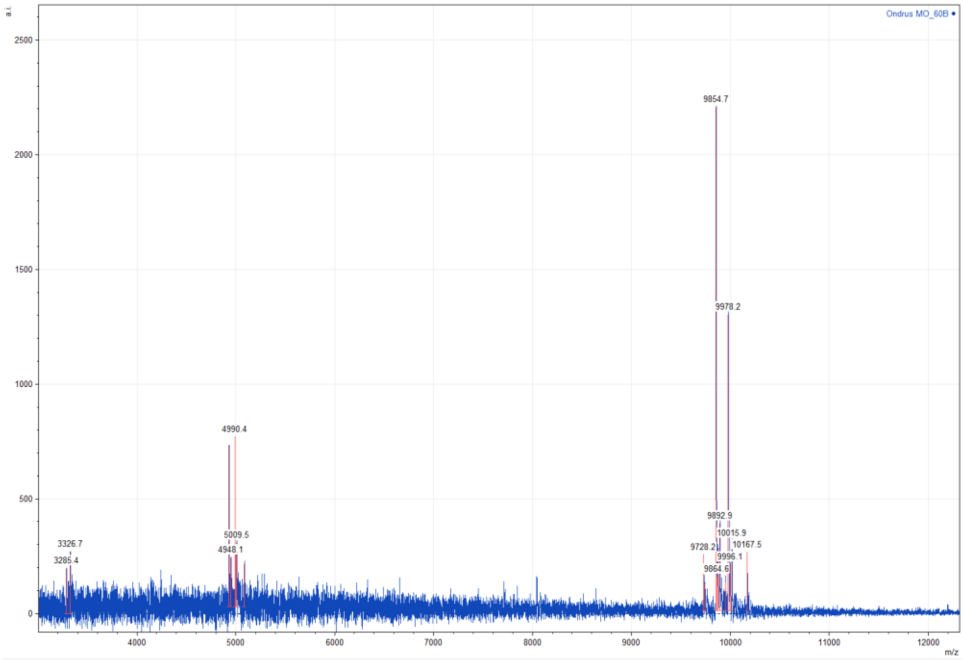

Figure S32. MALDI-TOF spectra of **31ON_U^{APh}**: calculated: 9977.5 Da; found: 9978.2 Da; Δ = 0.7 (M = 9854.7 Da is template strand).

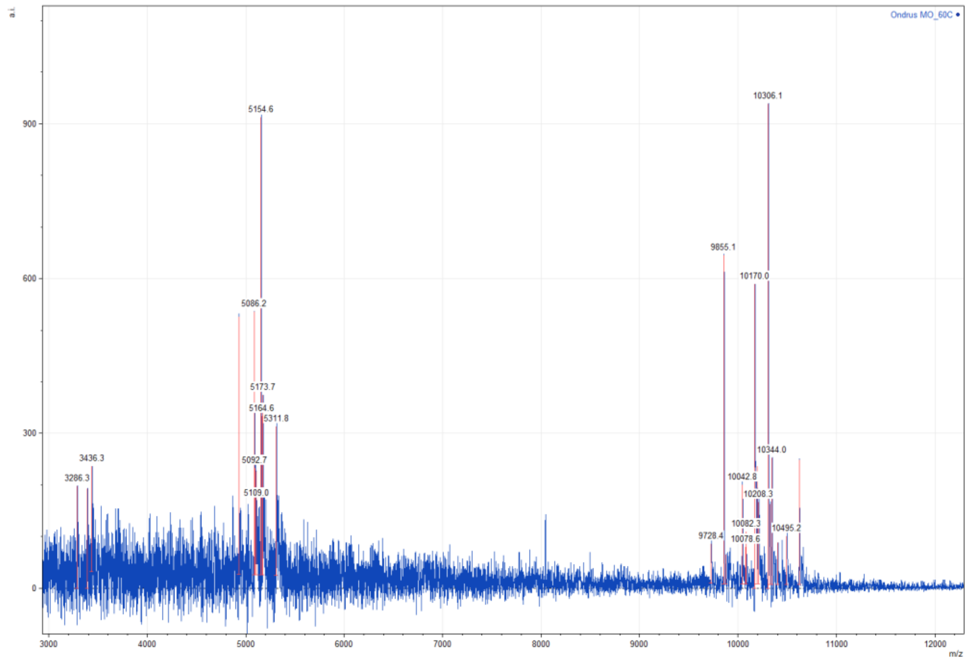

Figure S33. MALDI-TOF spectra of **31ON_A^{EIn}**: 10169.5 Da; found: 10170.0 Da; Δ = 0.5 (M = 9855.1 Da is template strand).

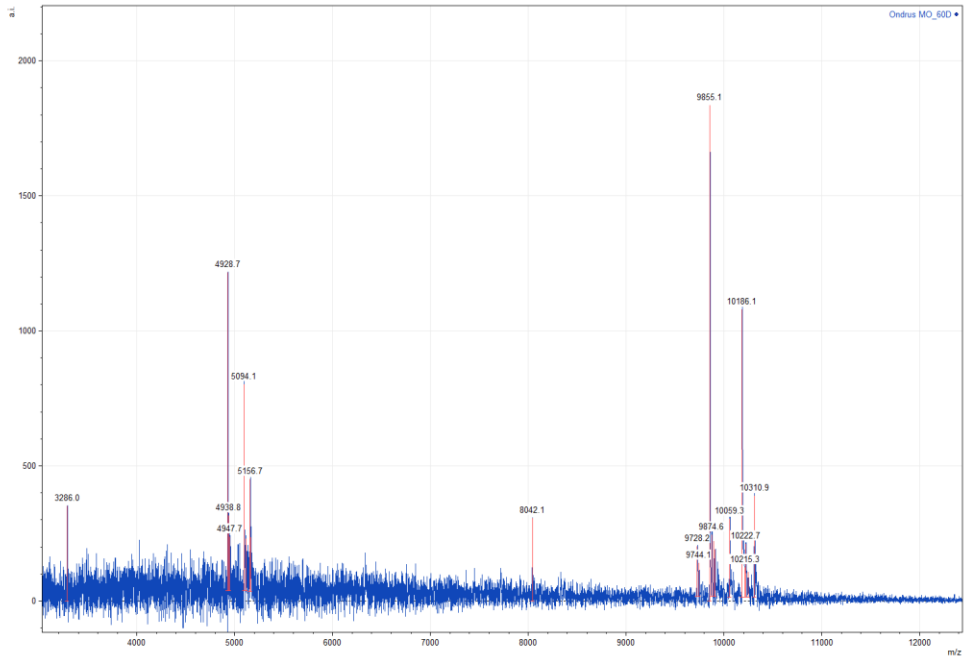

Figure S34. MALDI-TOF spectra of **31ON_A^{AIn}**: calculated: 10185.6 Da; found: 10186.1 Da; Δ = 0.5 (M = 9855.1 Da is template strand)

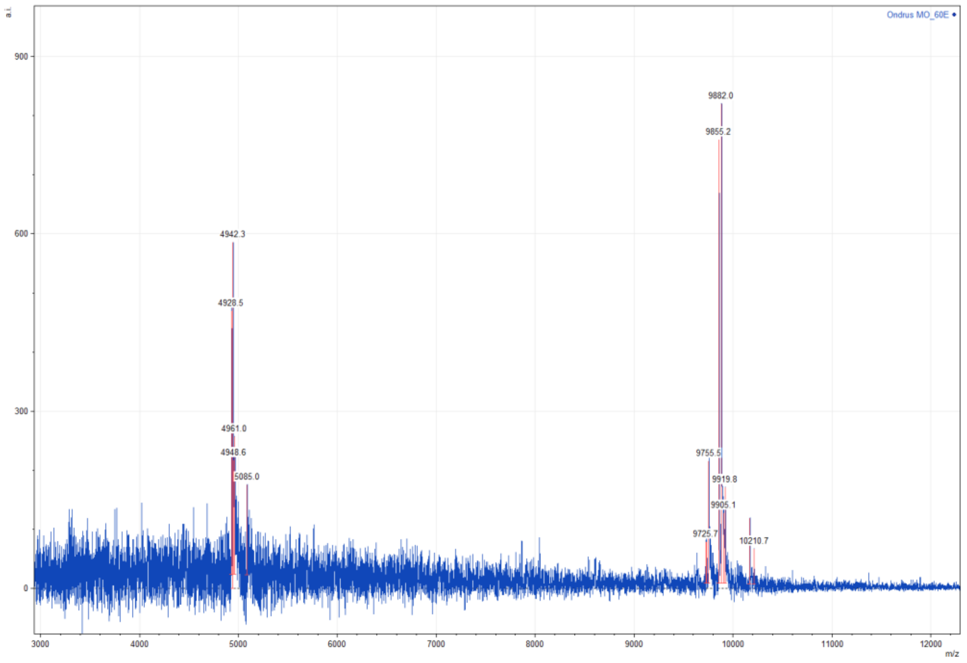

Figure S35. MALDI-TOF spectra of **31ON_C^{EAlk}**: calculated: 9881.5 Da; found: 9882.0 Da; Δ = 0.5 (M = 9855.2 Da is template strand).

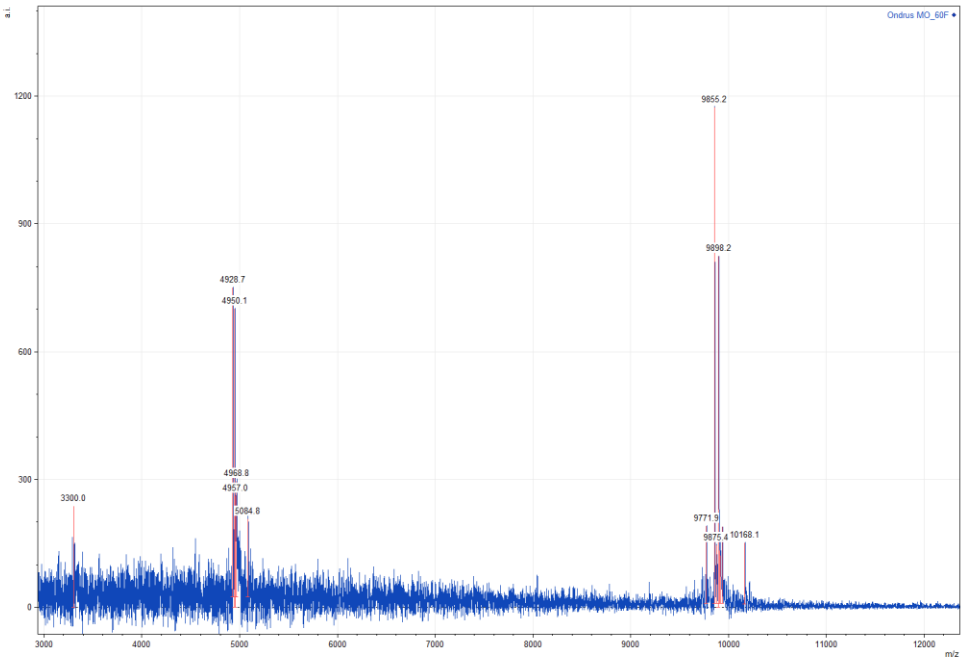

Figure S36. MALDI-TOF spectra of **31ON_C^{AAlk}**: calculated: 9897.6 Da; found: 9898.2 Da; Δ = 0.6 (M = 9855.2 Da is template strand).

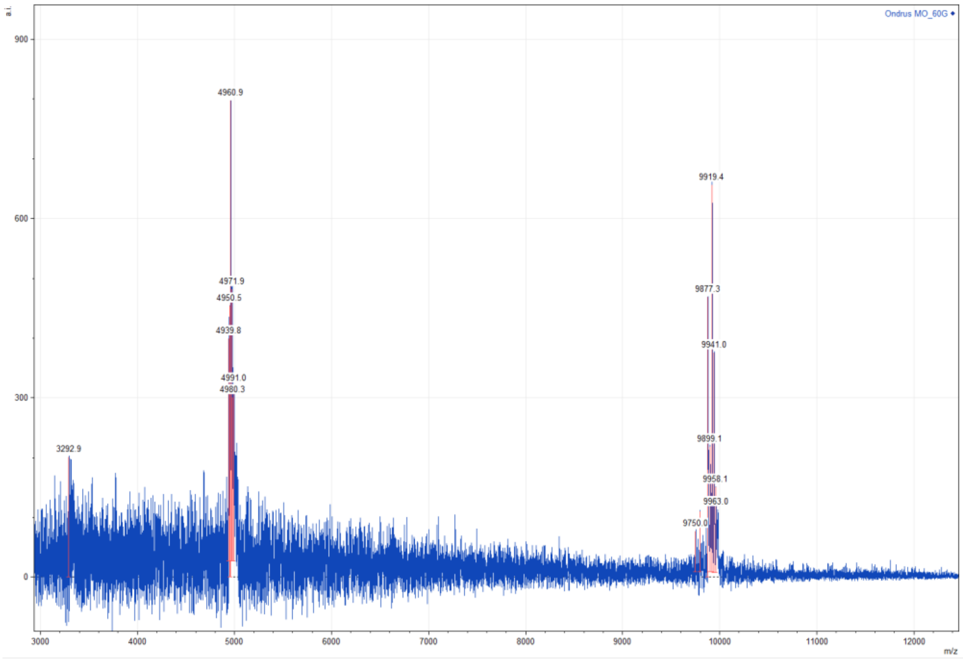

Figure S37. MALDI-TOF spectra of **31ON_G^{EiPr}**: calculated: 9877.5 Da; found: 9877.3 Da; Δ = 0.2 (M = 9919.4 Da is full product plus K).

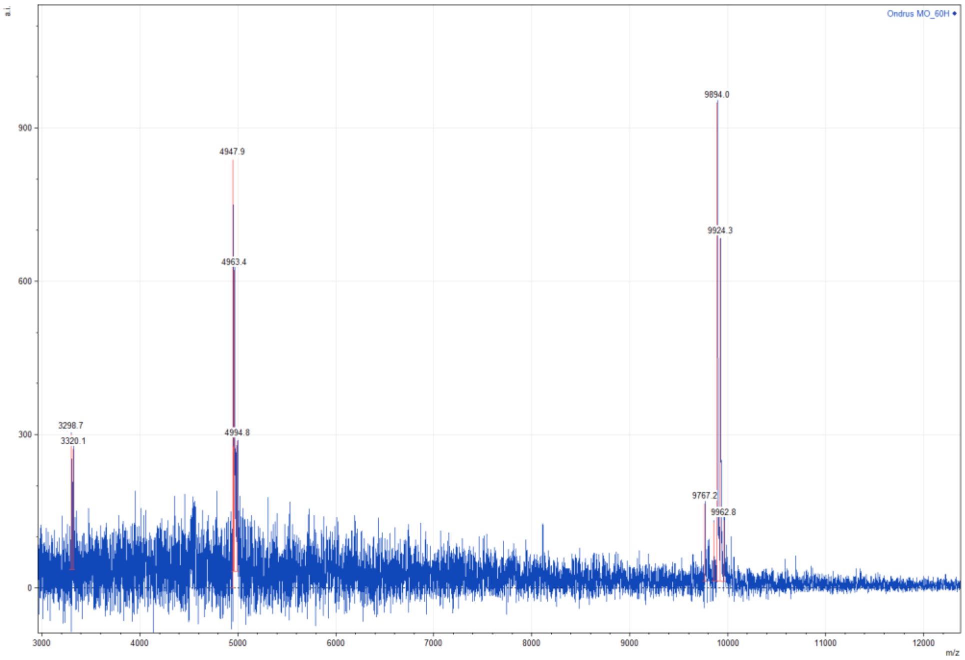

Figure S38. MALDI-TOF spectra of **31ON_G^{AiPr}**: calculated: 9893.7 Da; found: 9894.0 Da; Δ = 0.3.

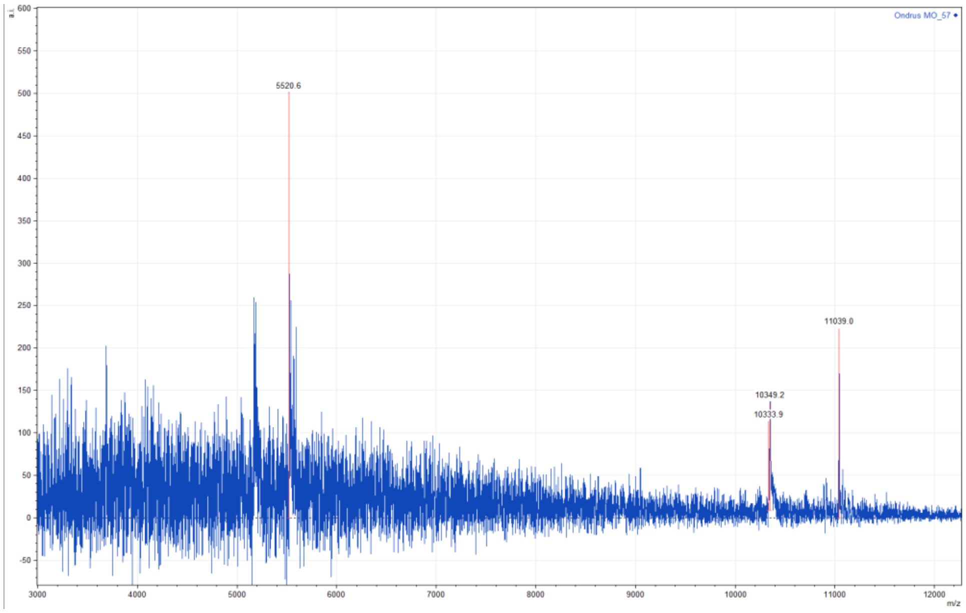

Figure S39. MALDI-TOF spectra of **31ON_N^{ER}**: calculated: 11037.9 Da; found: 11039.0 Da; Δ = 1.1.

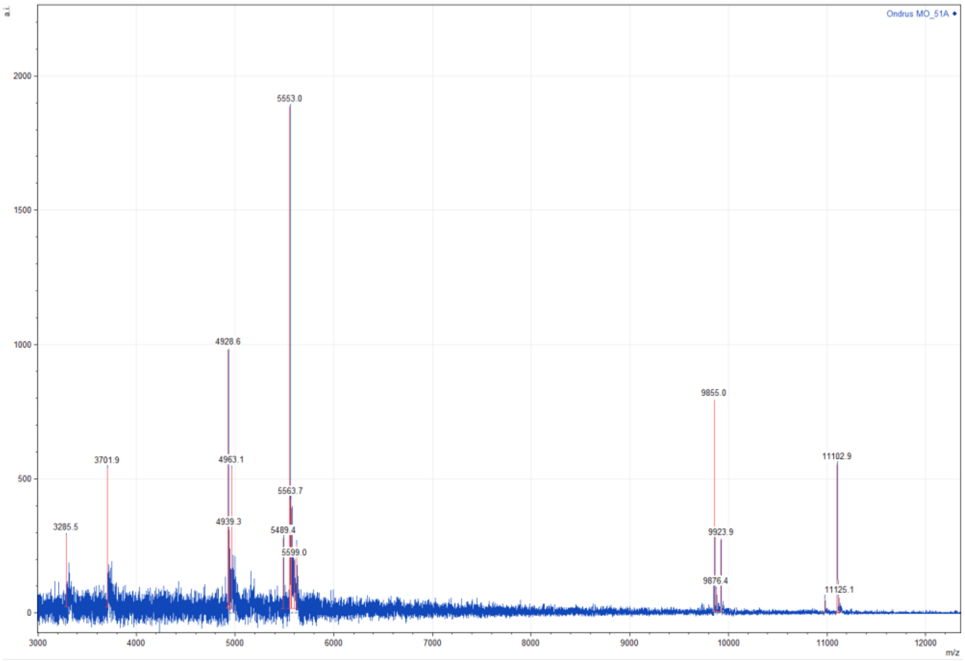

Figure S40. MALDI-TOF spectra of **31ON_N^{AR}**: calculated: 11102.5 Da; found: 11102.9 Da; Δ = 0.4 (M = 9855.0 Da is template strand).

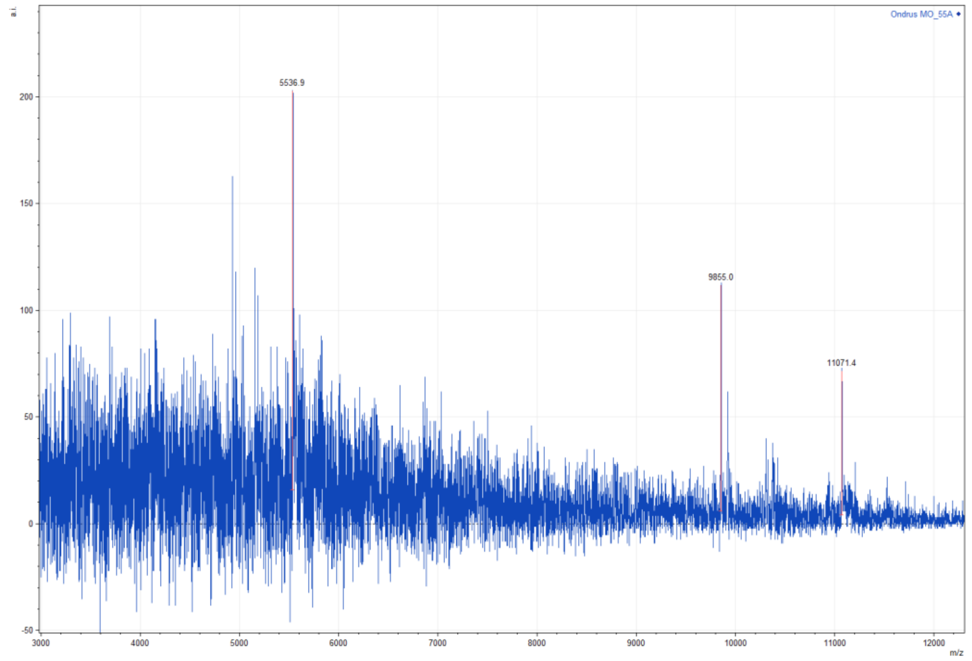

Figure S41. MALDI-TOF spectra of **31ON_U$^{\mathbf{APh}}$A$^{\mathbf{EIn}}$C$^{\mathbf{AAlk}}$G$^{\mathbf{EiPr}}$**: calculated: 11070.2 Da; found: 11071.4 Da; Δ = 1.6 (M = 9855.0 Da is template strand).

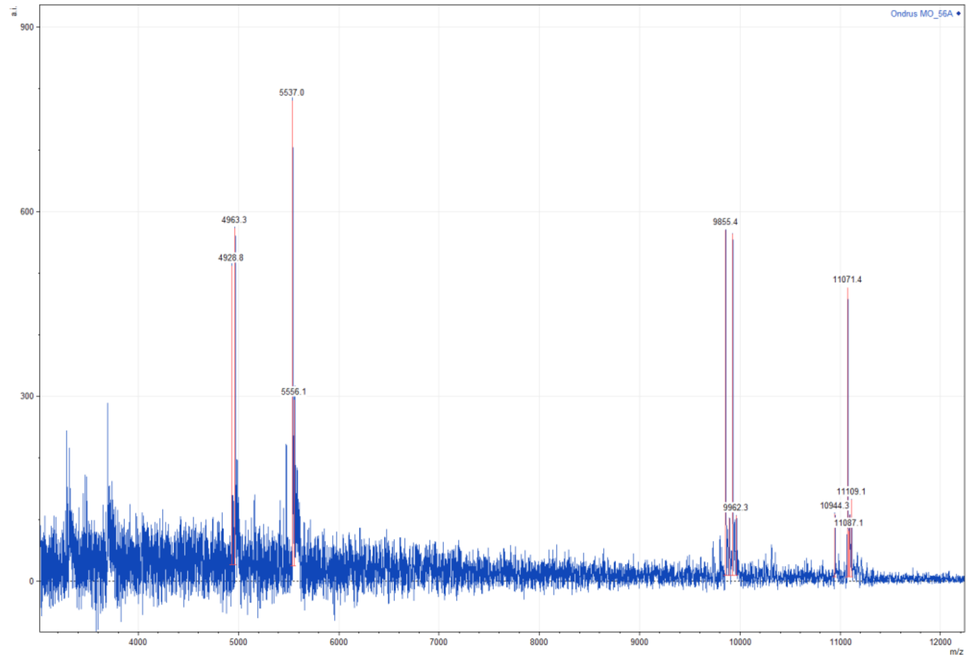

Figure S42. MALDI-TOF spectra of **31ON_U$^{\mathbf{EPh}}$A$^{\mathbf{AIn}}$C$^{\mathbf{EAlk}}$G$^{\mathbf{AiPr}}$**: calculated: 11070.2 Da; found: 11071.4 Da; Δ = 0.8 (M = 9855.4 Da is template strand).

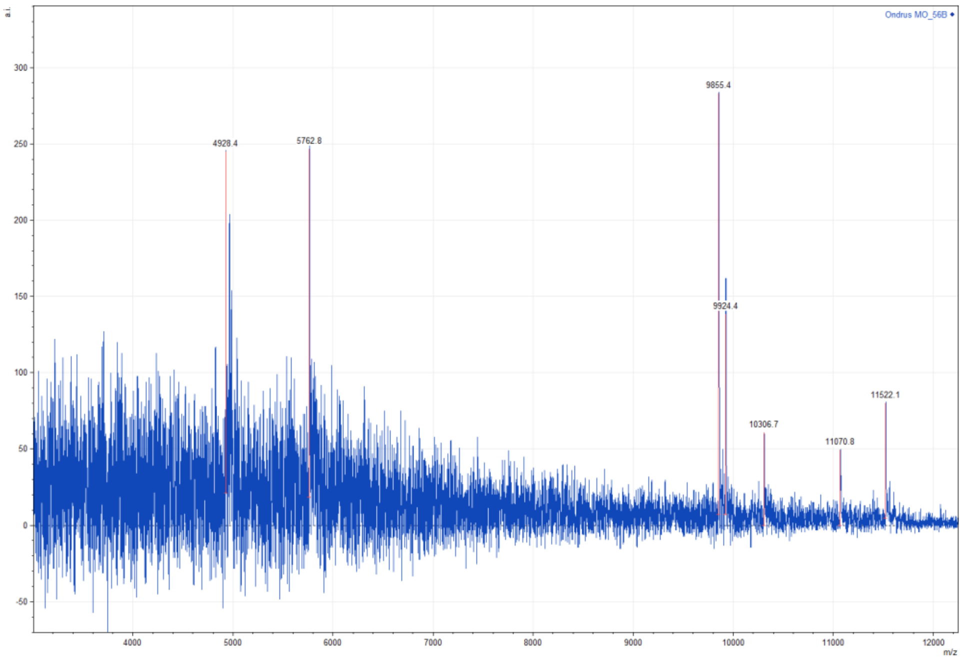

Figure S43. MALDI-TOF spectra of **31ON_U^{EPh}A^{Eln}C^{AAlk}G^{AiPr}**: calculated: 11070.2 Da; found: 11070.8 Da; Δ = 0.6 (M = 9855.4 Da is template strand, M = 11522.1 Da is full product plus modified **dA^{Eln}TP**).

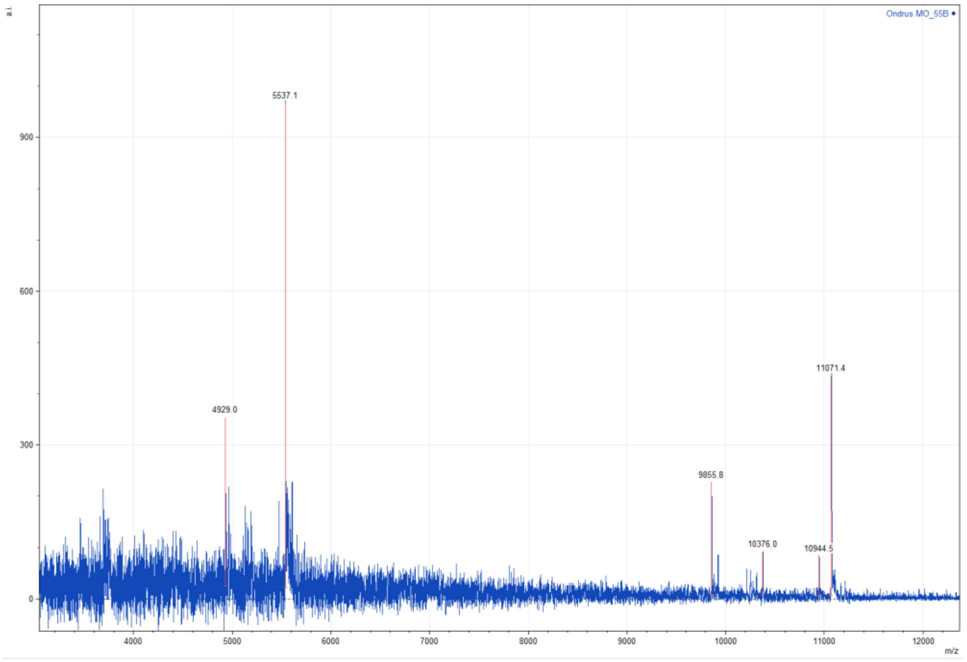

Figure S44. MALDI-TOF spectra of **31ON_U^{APh}A^{Aln}C^{EAlk}G^{EiPr}**: calculated: 11070.2 Da; found: 11071.4 Da; Δ = 1.2 (M = 9855.8 Da is template strand).

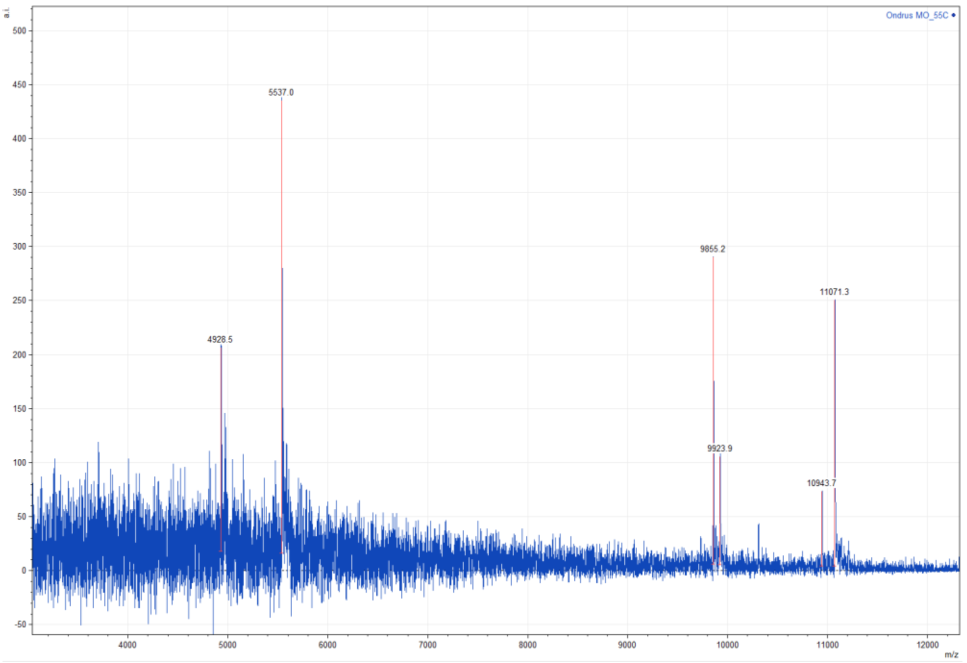

Figure S45. MALDI-TOF spectra of **31ON_U$^{\mathbf{APh}}$A$^{\mathbf{EIn}}$C$^{\mathbf{EAlk}}$G$^{\mathbf{AiPr}}$**: calculated: 11070.2 Da; found: 11071.3 Da; Δ = 1.1 (M = 9855.2 Da is template strand).

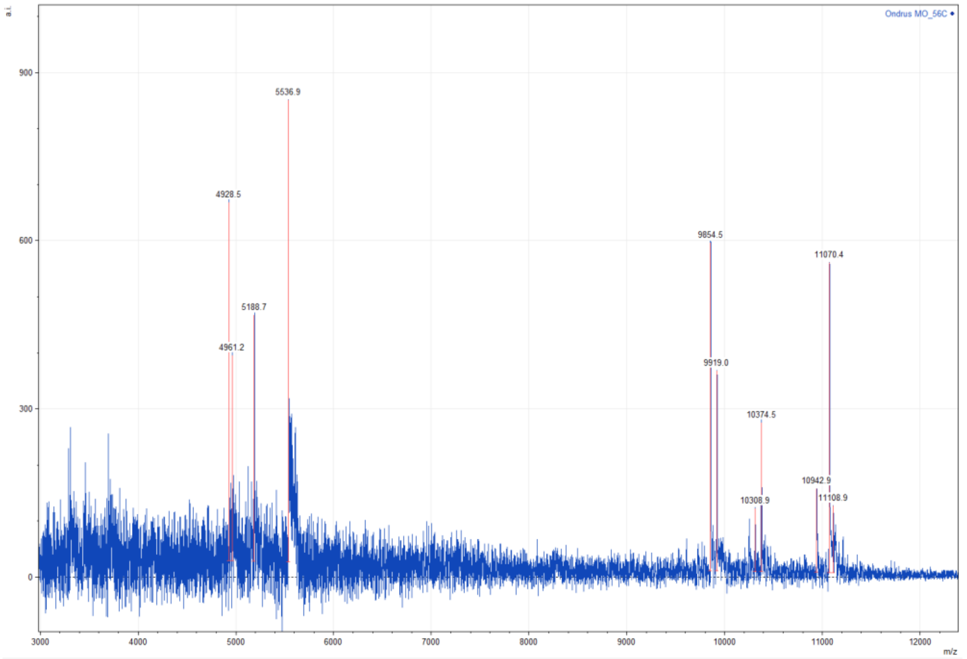

Figure S46. MALDI-TOF spectra of **31ON_U$^{\mathbf{EPh}}$A$^{\mathbf{AIn}}$C$^{\mathbf{AAlk}}$G$^{\mathbf{EiPr}}$**: calculated: 11070.2 Da; found: 11070.4 Da; Δ = 0.2 (M = 9854.5 Da is template strand).

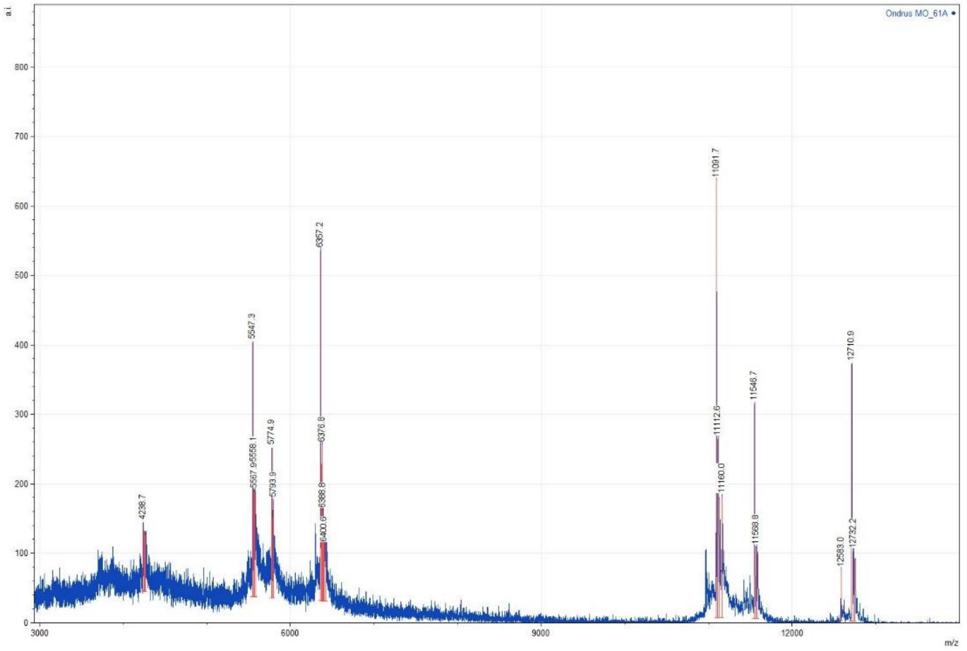

Figure S47. MALDI-TOF spectra of **35ON_N^{AR}**: calculated: 12709.6 Da; found: 12710.9 Da; Δ = 1.3 (M = 11091.7 Da is mass of template strand).

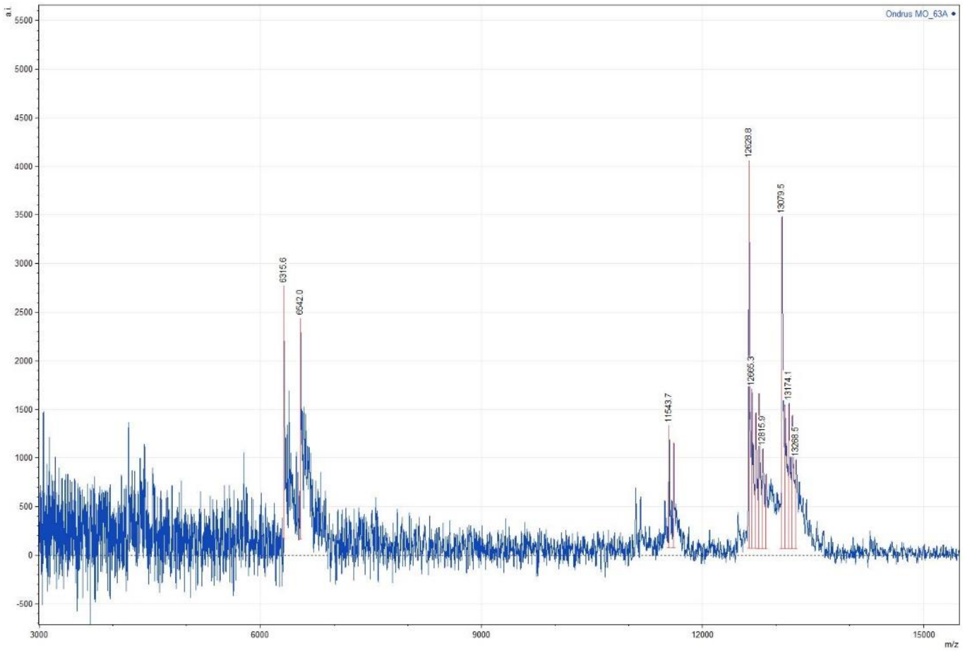

Figure S48. MALDI-TOF spectra of **35ON_N^{ER}**: calculated: 12628.9 Da; found: 12628.8 Da; Δ = 0.1 (M = 13079.5 Da is product with additional modified **dA^{EIn}TP**).

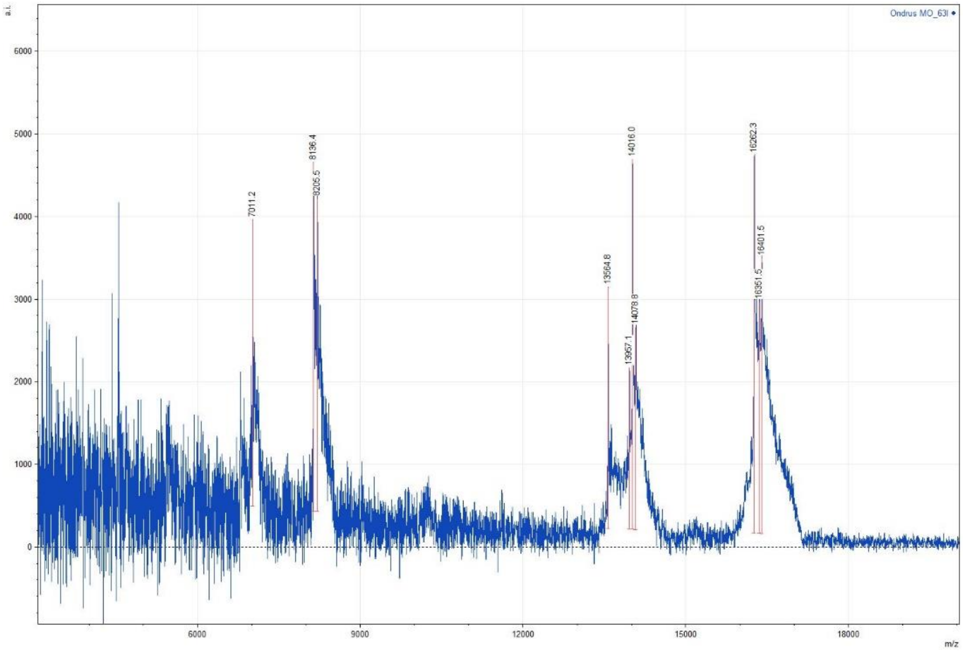

Figure S49. MALDI-TOF spectra of **43ON_N^{ER}**: calculated: 15810.8 Da; found: 16262.3 Da; Δ = 451.5 (M = 16262.3 Da correspond to product with additional modified **dA^{EIn}TP**; M = 13564.8 Da is mass of template strand).

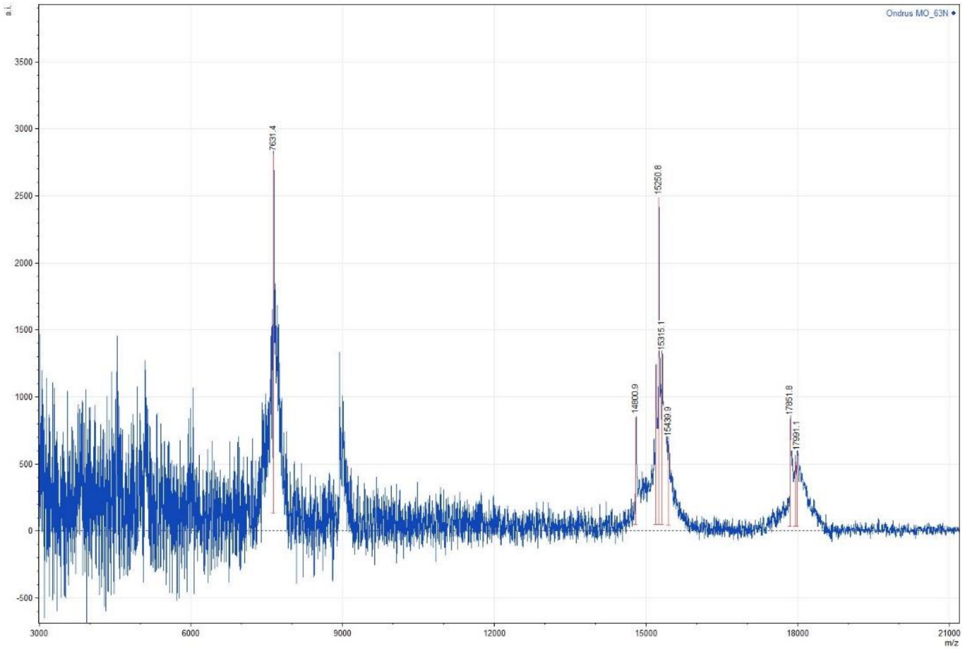

Figure S50. MALDI-TOF spectra of **47ON_N^{ER}**: calculated: 17401.8 Da; found: 17851.8 Da; Δ = 450.0 (M = 17851.8 Da is product with additional modified **dA^{EIn}TP**; M = 14800.9 Da is mass of template strand).

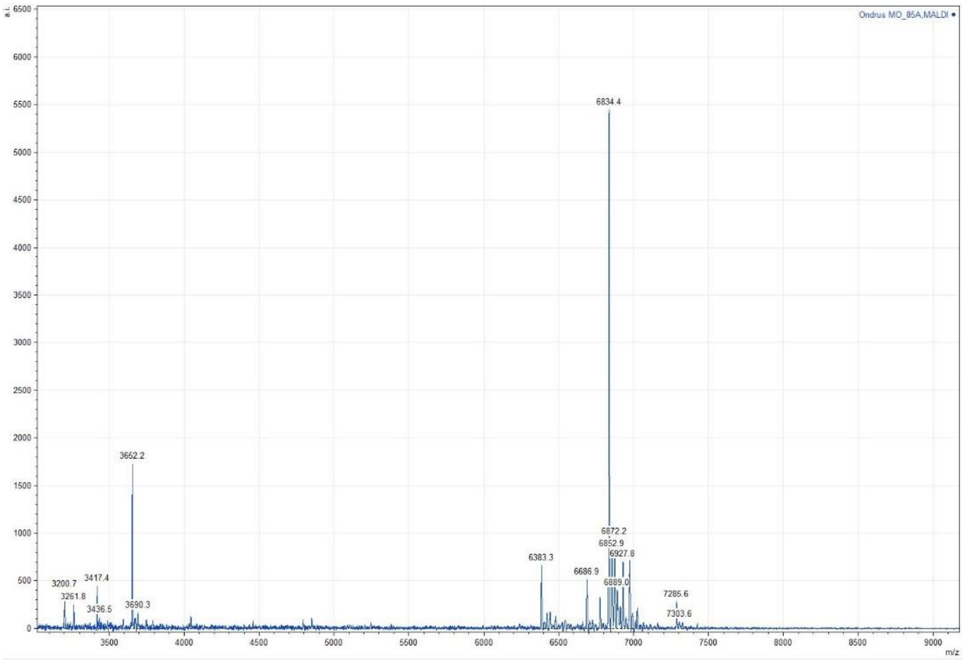

Figure S51. MALDI-TOF spectra of **17ON_N^{ER}**: calculated: 6380.8 Da; found: 6834.4 Da; Δ = 453.6 (M = 6834.4 Da is product with additional modified **dA^{EIn}TP**.

5) Copies of ^{1}H, ^{13}C and ^{31}P NMR spectra of prepared compounds

^{1}H and ^{13}C NMR spectra of compound **dU^{EPh}**:

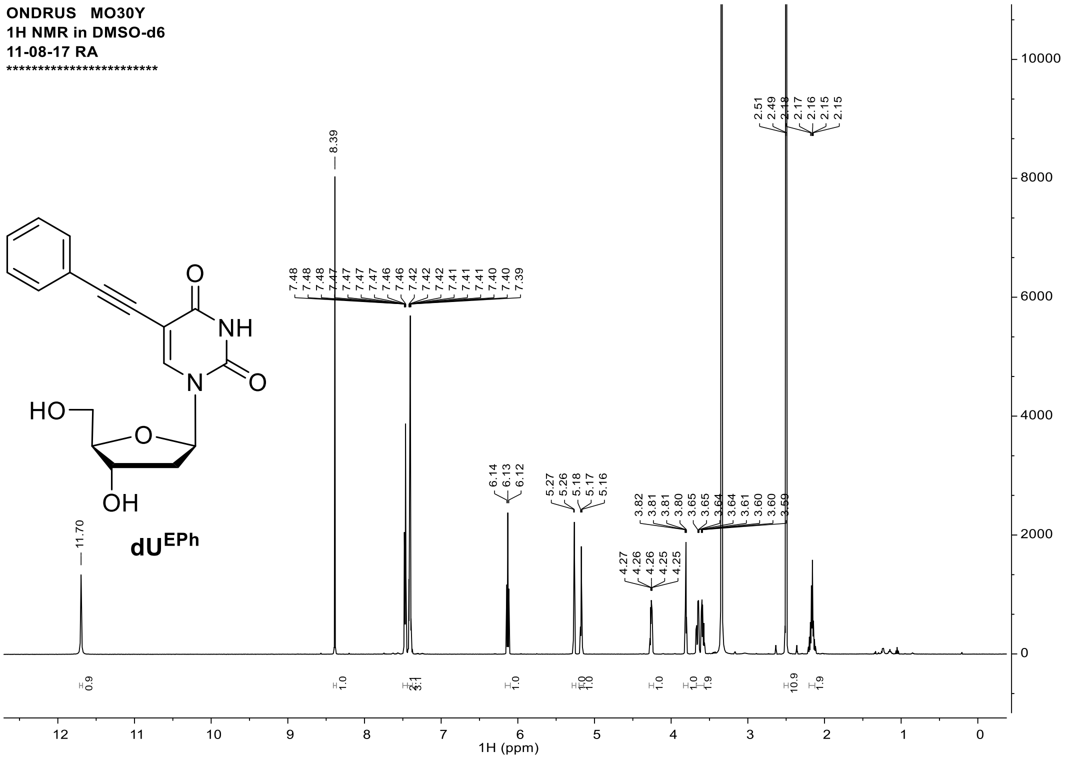

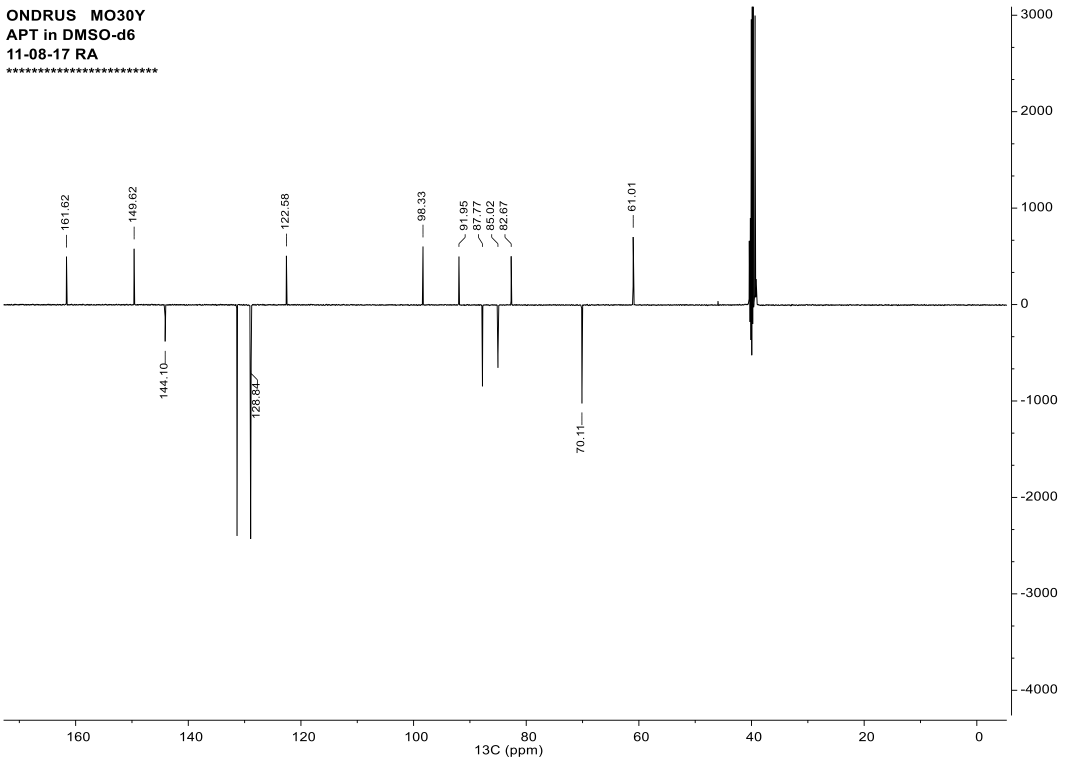

^{1}H, ^{13}C and ^{31}P NMR spectra of **dU^{EPh}TP**:

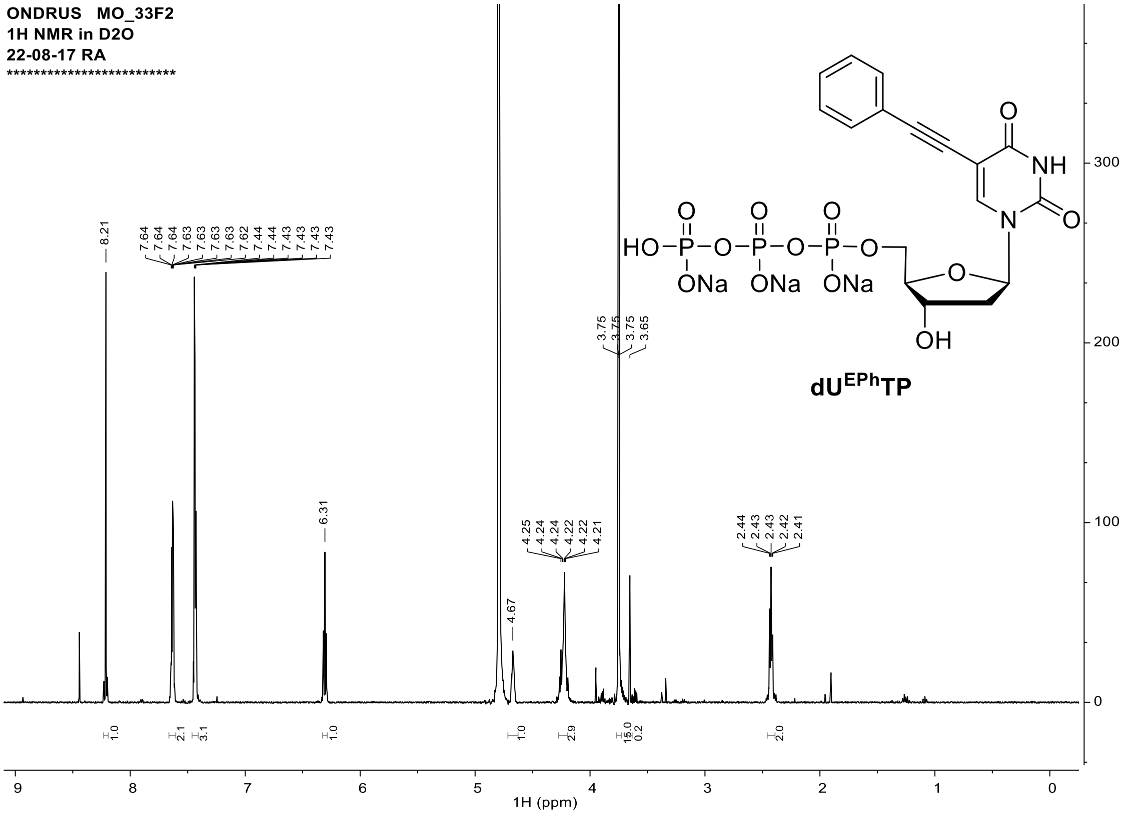

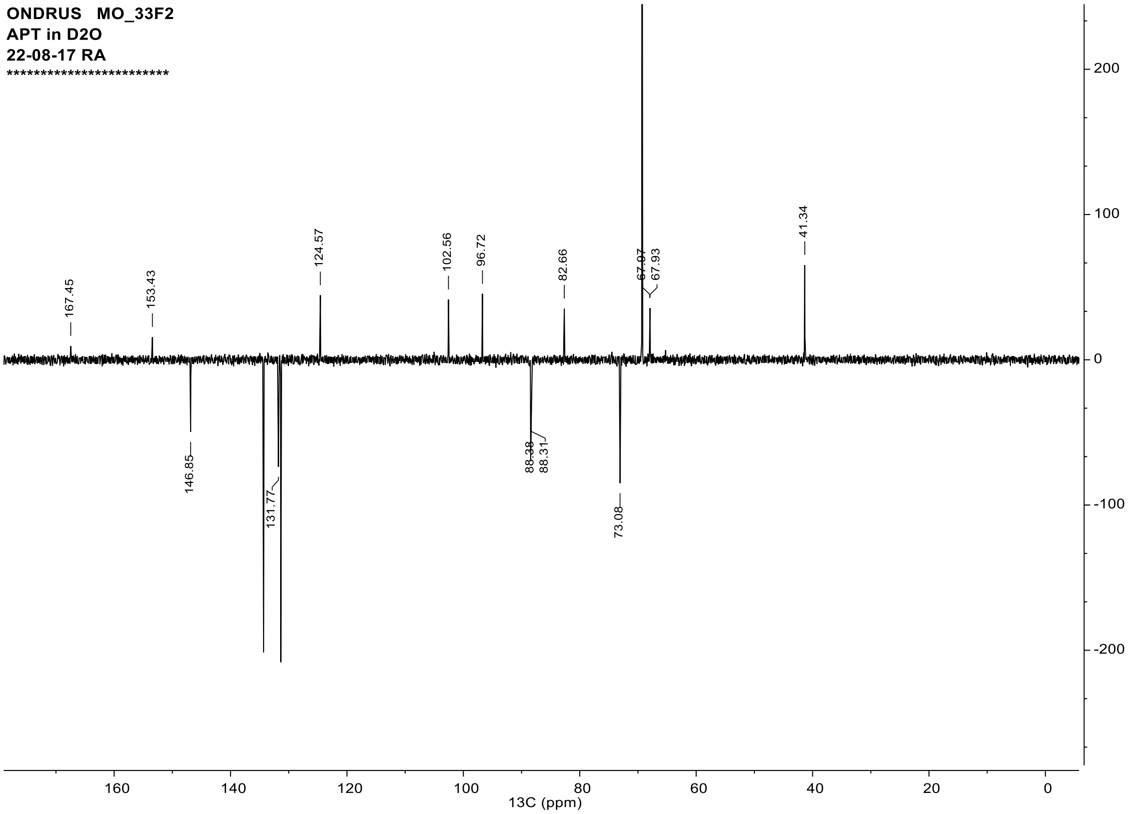

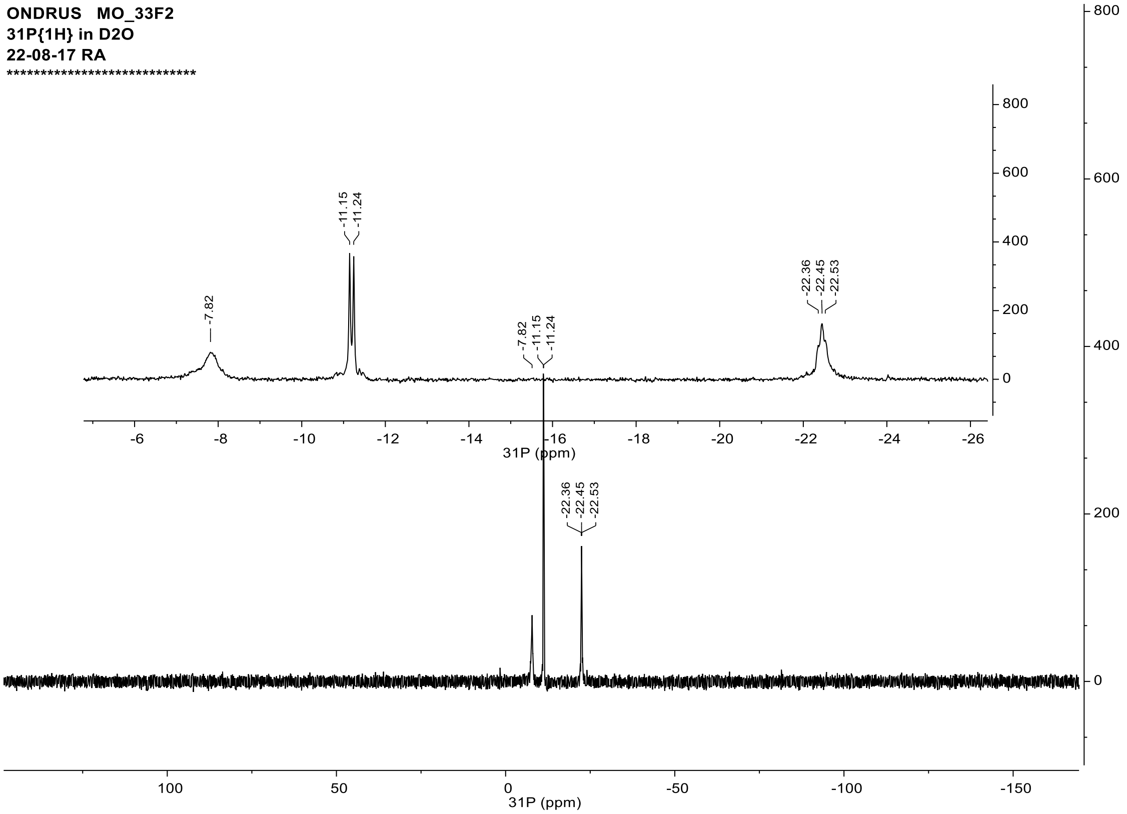

^{1}H and ^{13}C NMR spectra of compound **dU^{APh}**:

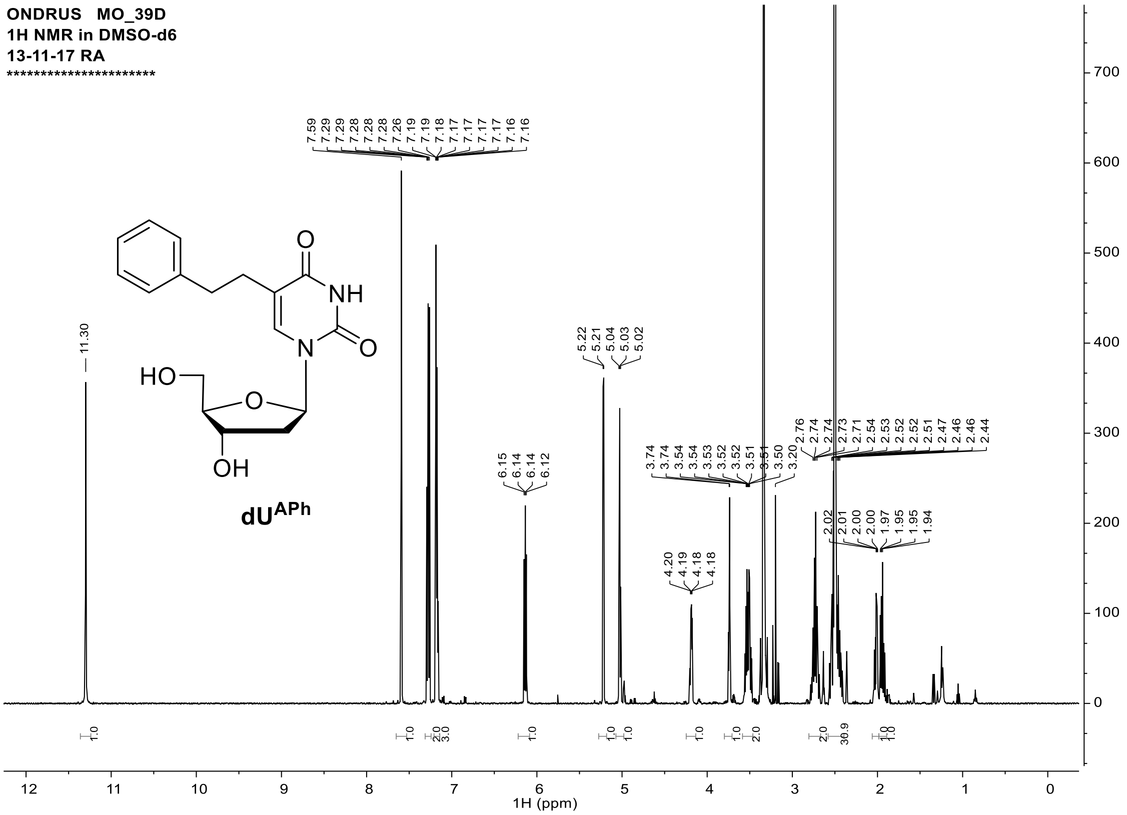

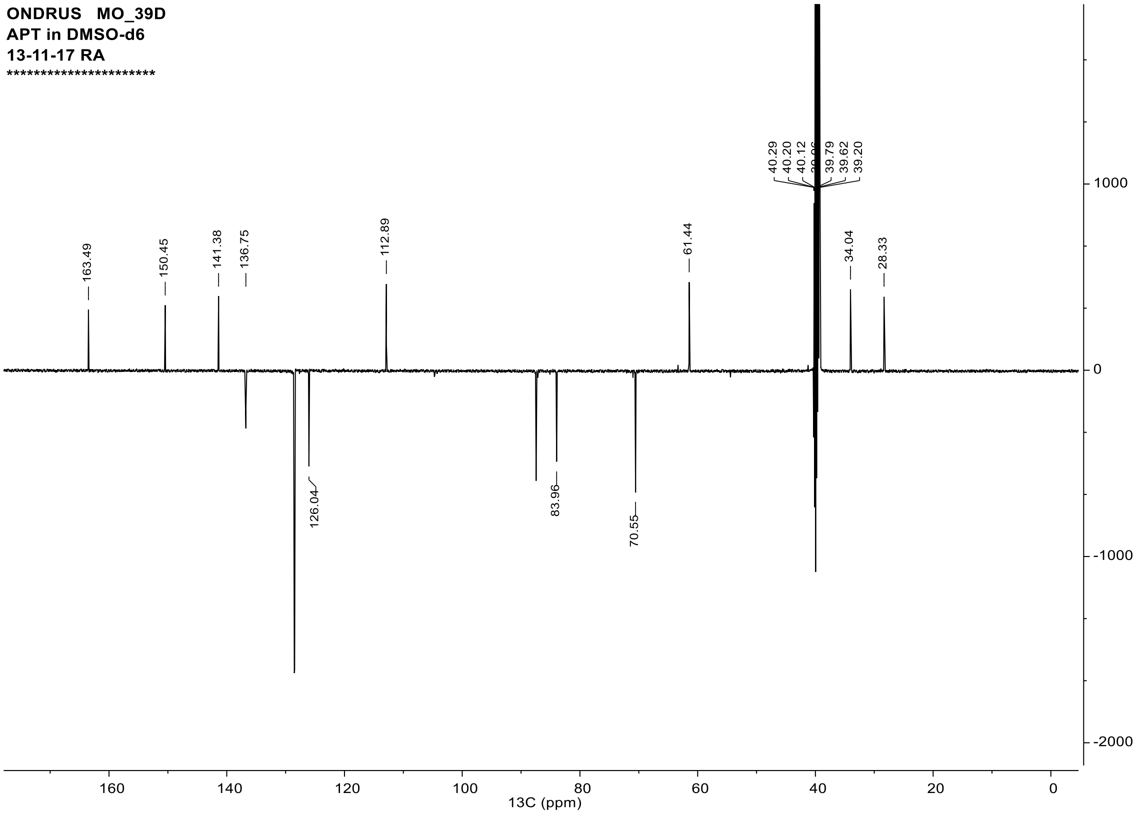

^{1}H, ^{13}C and ^{31}P NMR spectra of **dU^{APh}TP**:

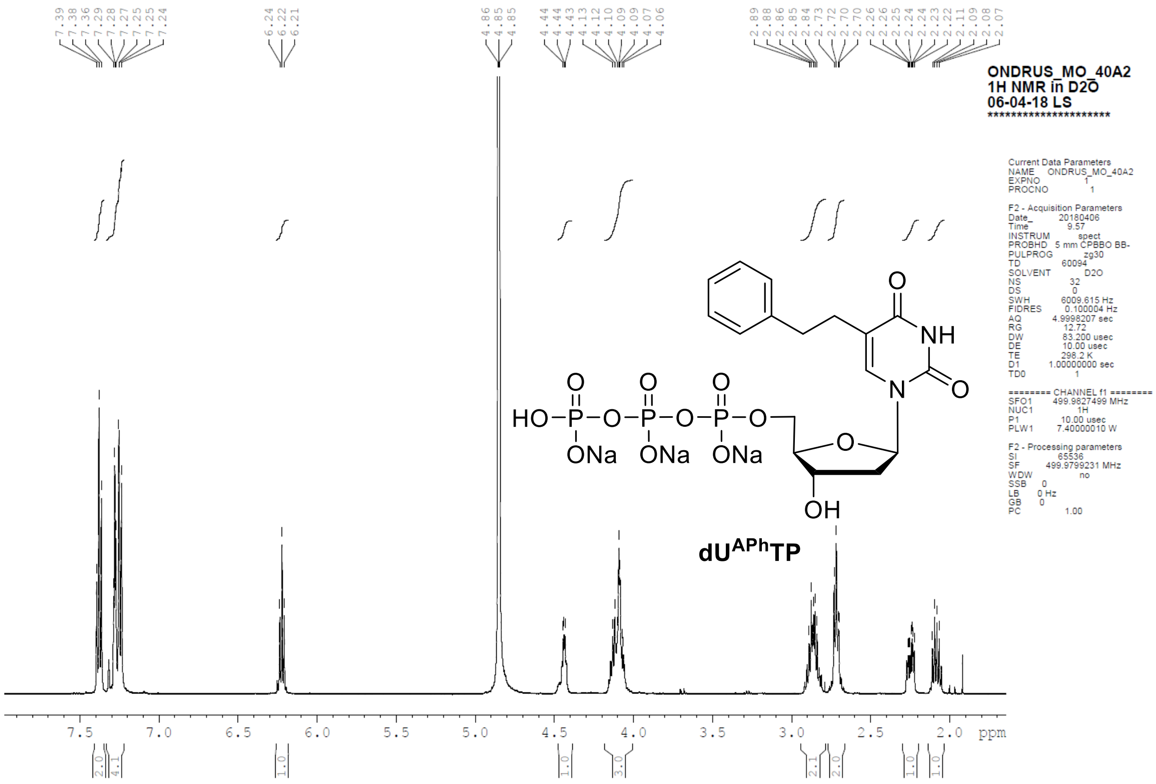

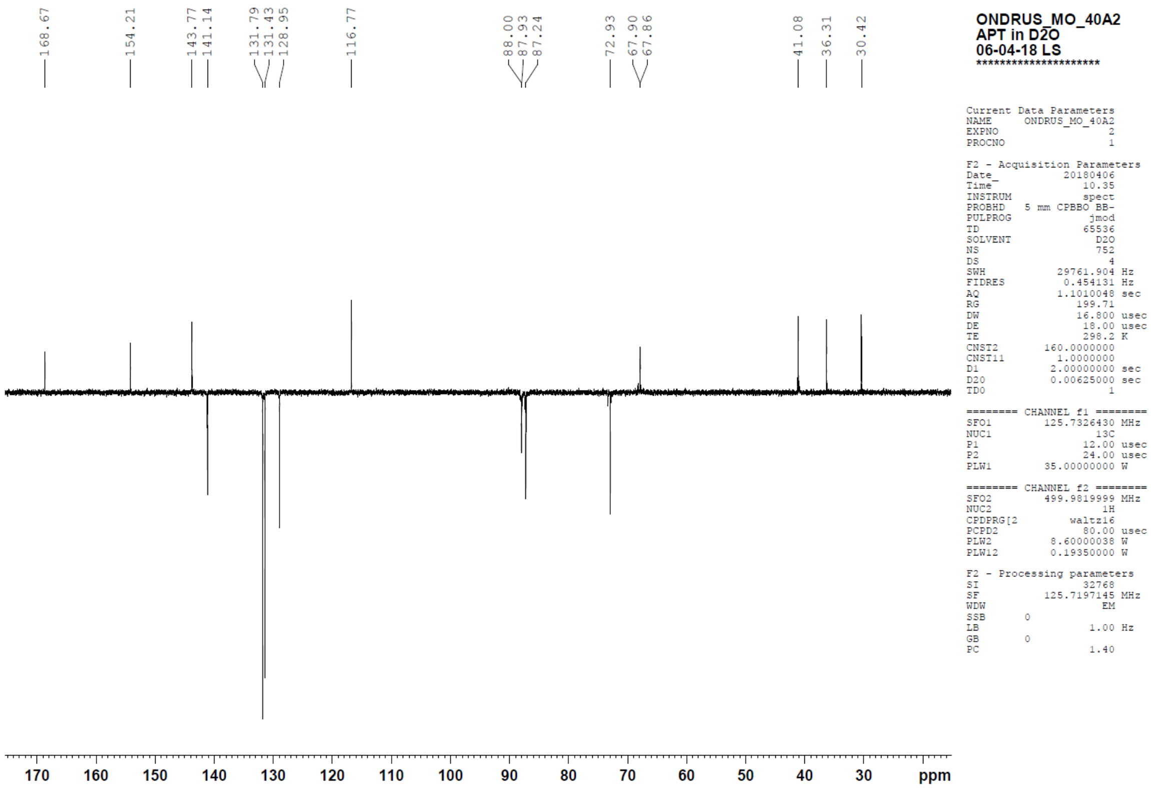
168.67
154.21
143.77
141.14
131.79
131.43
128.95
116.77
88.00
87.93
87.24
72.93
67.90
67.86
41.08
36.31
30.42
ONDRUS_MO_40A2
APT in D2O
06-04-18 LS
Current Data Parameters
NAME ONDRUS_MO_40A2
EXPNO 2
PROCNO 1
F2 - Acquisition Parameters
Date_ 20180406
Time 10.35
INSTRUM spect
PROBHD 5 mm CPBBO BB-
PULPROG jmod
TD 65536
SOLVENT D2O
NS 752
DS 4
SWH 29761.904 Hz
FIDRES 0.454131 Hz
AQ 1.1010048 sec
RG 199.71
DW 16.800 usec
DE 18.00 usec
TE 298.2 K
CNST2 160.0000000
CNST11 1.0000000
D1 2.00000000 sec
D20 0.00625000 sec
TD0 1
======== CHANNEL f1 ========
SFO1 125.7326430 MHz
NUC1 13C
P1 12.00 usec
P2 24.00 usec
PLW1 35.00000000 W
======== CHANNEL f2 ========
SFO2 499.9819999 MHz
NUC2 1H
CPDPRG[2 waltz16
PCPD2 80.00 usec
PLW2 8.60000038 W
PLW12 0.19350000 W
F2 - Processing parameters
SI 32768
SF 125.7197145 MHz
WDW EM
SSB 0
LB 1.00 Hz
GB 0
PC 1.40
170 160 150 140 130 120 110 100 90 80 70 60 50 40 30 ppm

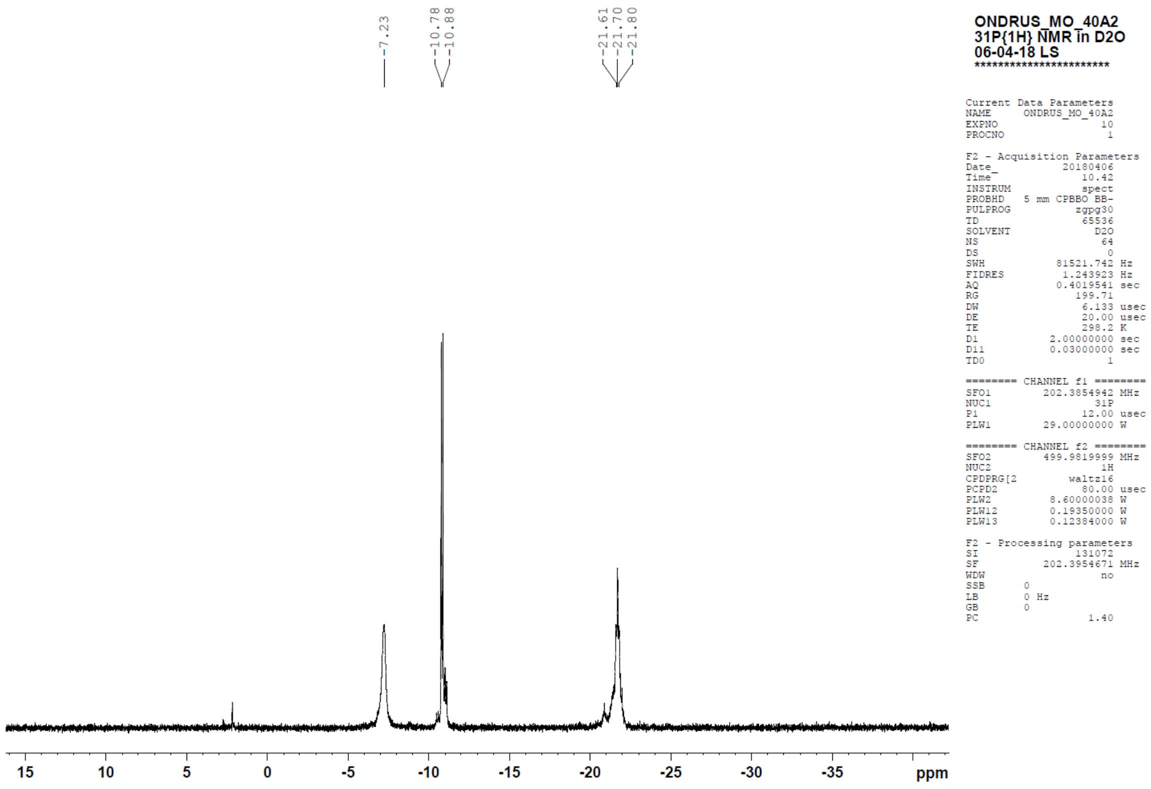
-7.23
-10.78
-10.88
-21.61
-21.70
-21.80
ONDRUS_MO_40A2
31P{1H} NMR in D2O
06-04-18 LS
Current Data Parameters
NAME ONDRUS_MO_40A2
EXPNO 10
PROCNO 1
F2 - Acquisition Parameters
Date_ 20180406
Time 10.42
INSTRUM spect
PROBHD 5 mm CPBBO BB-
PULPROG zgpg30
TD 65536
SOLVENT D2O
NS 64
DS 0
SWH 81521.742 Hz
FIDRES 1.243923 Hz
AQ 0.4019541 sec
RG 199.71
DW 6.133 usec
DE 20.00 usec
TE 298.2 K
D1 2.00000000 sec
D11 0.03000000 sec
TD0 1
======== CHANNEL f1 ========
SFO1 202.3854942 MHz
NUC1 31P
P1 12.00 usec
PLW1 29.00000000 W
======== CHANNEL f2 ========
SFO2 499.9819999 MHz
NUC2 1H
CPDPRG[2 waltz16
PCPD2 80.00 usec
PLW2 8.60000038 W
PLW12 0.19350000 W
PLW13 0.12384000 W
F2 - Processing parameters
SI 131072
SF 202.3954671 MHz
WDW no
SSB 0
LB 0 Hz
GB 0
PC 1.40
15 10 5 0 -5 -10 -15 -20 -25 -30 -35 ppm

^{1}H and ^{13}C NMR spectra of compound **dA^{Eln}**:

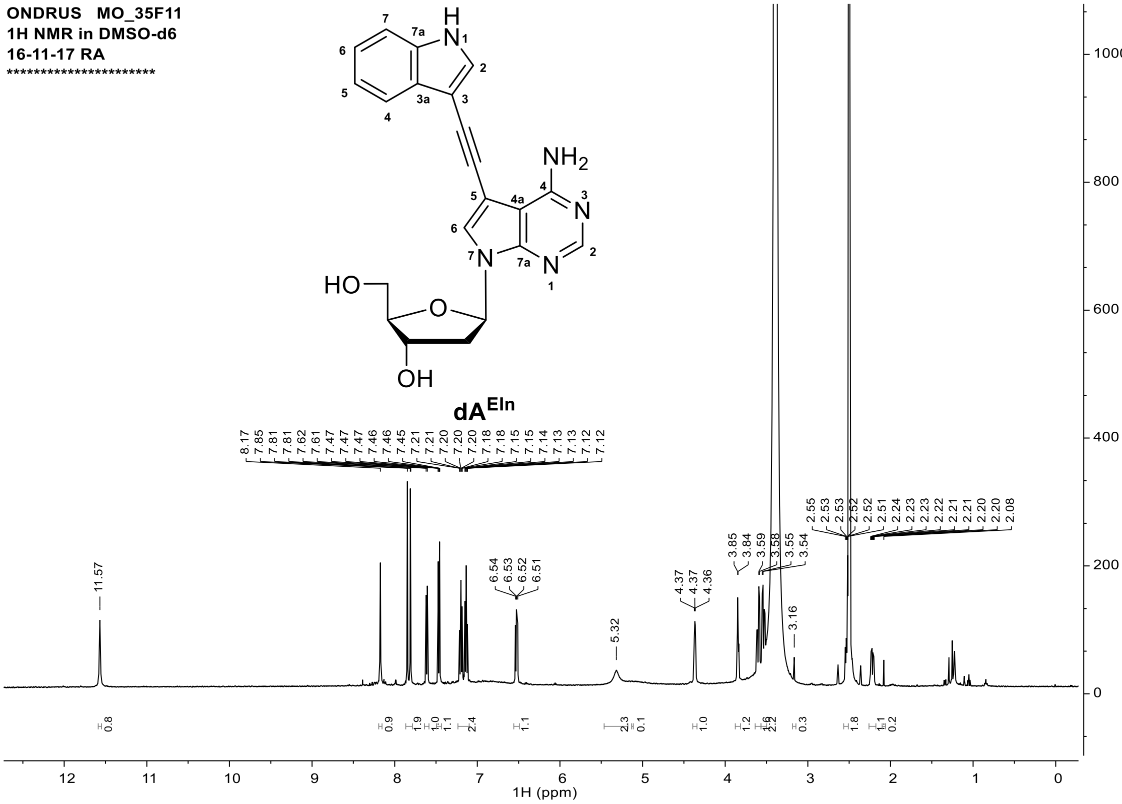

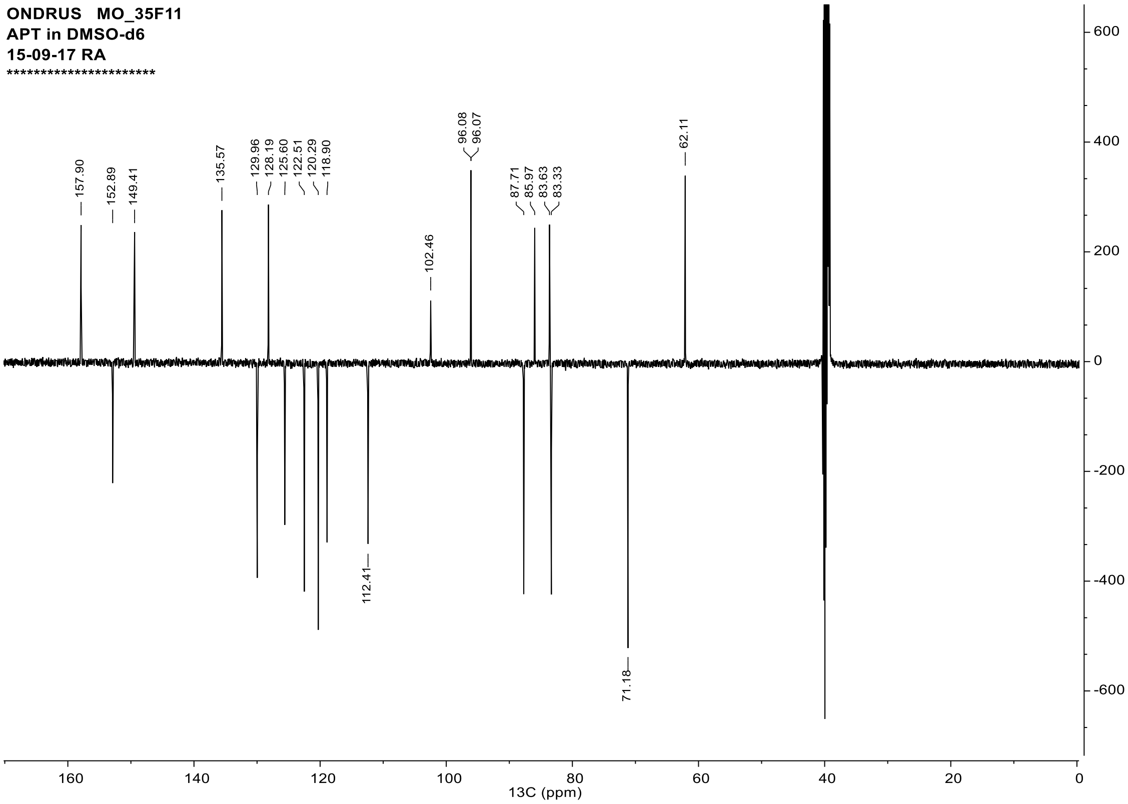

^{1}H, ^{13}C and ^{31}P NMR spectra of **dA^{EIn}TP**:

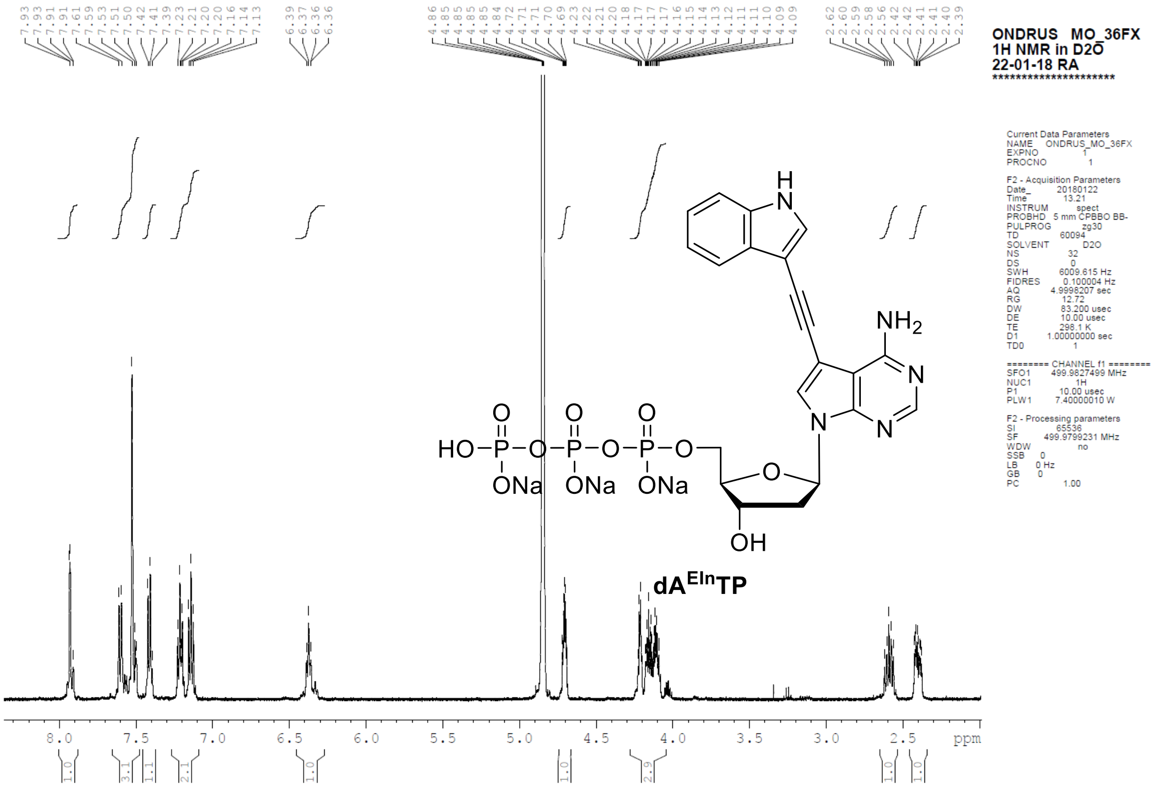

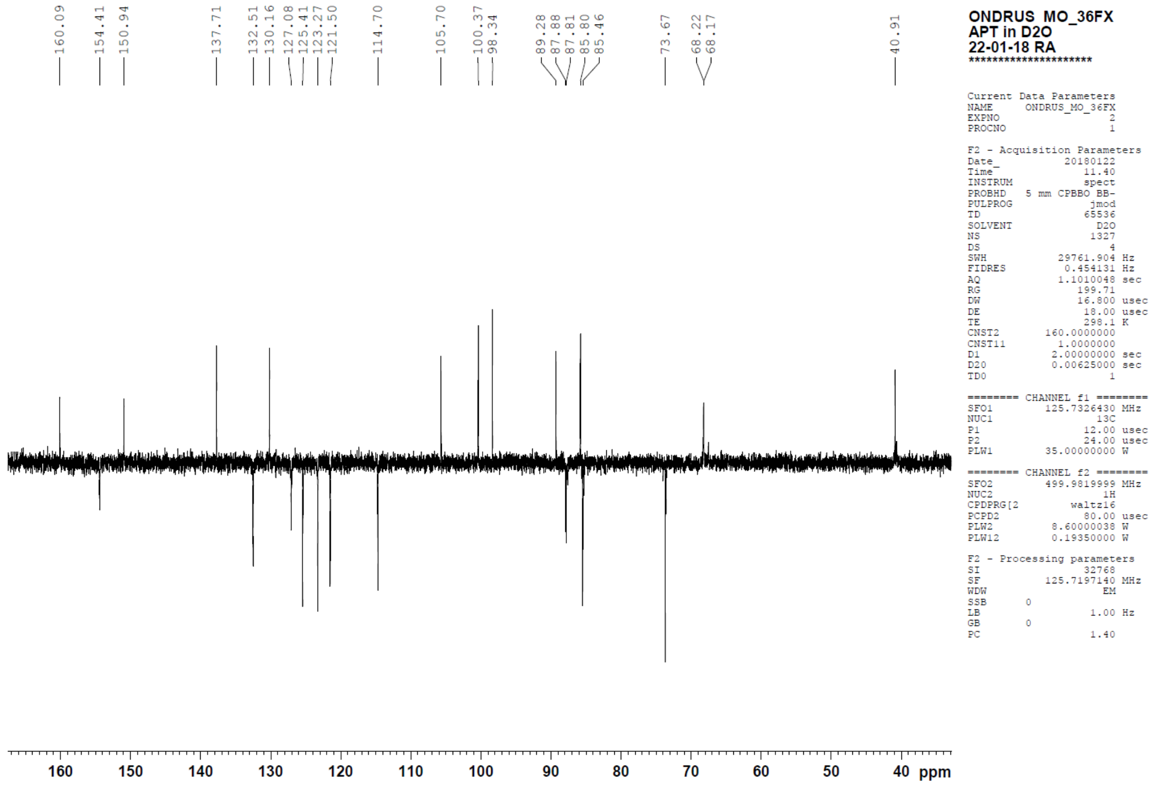

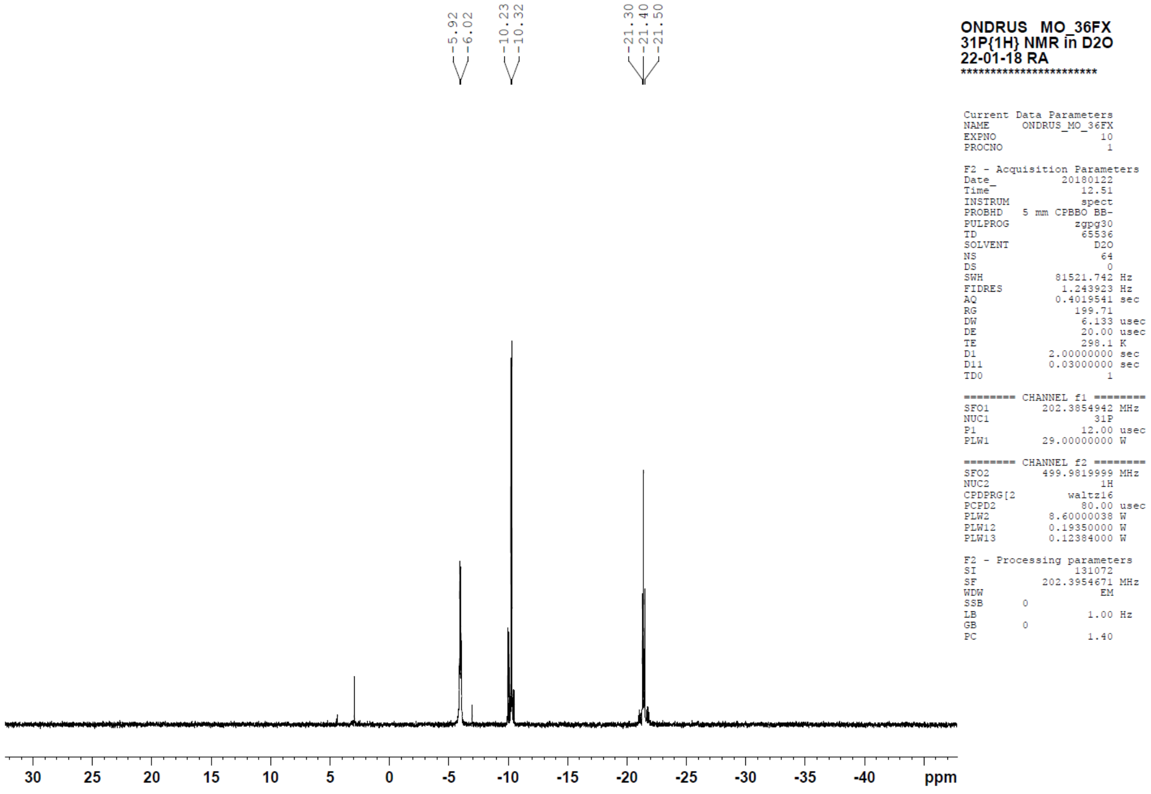

^{1}H and ^{13}C NMR spectra of **dA$^{\mathbf{Aln}}$**:

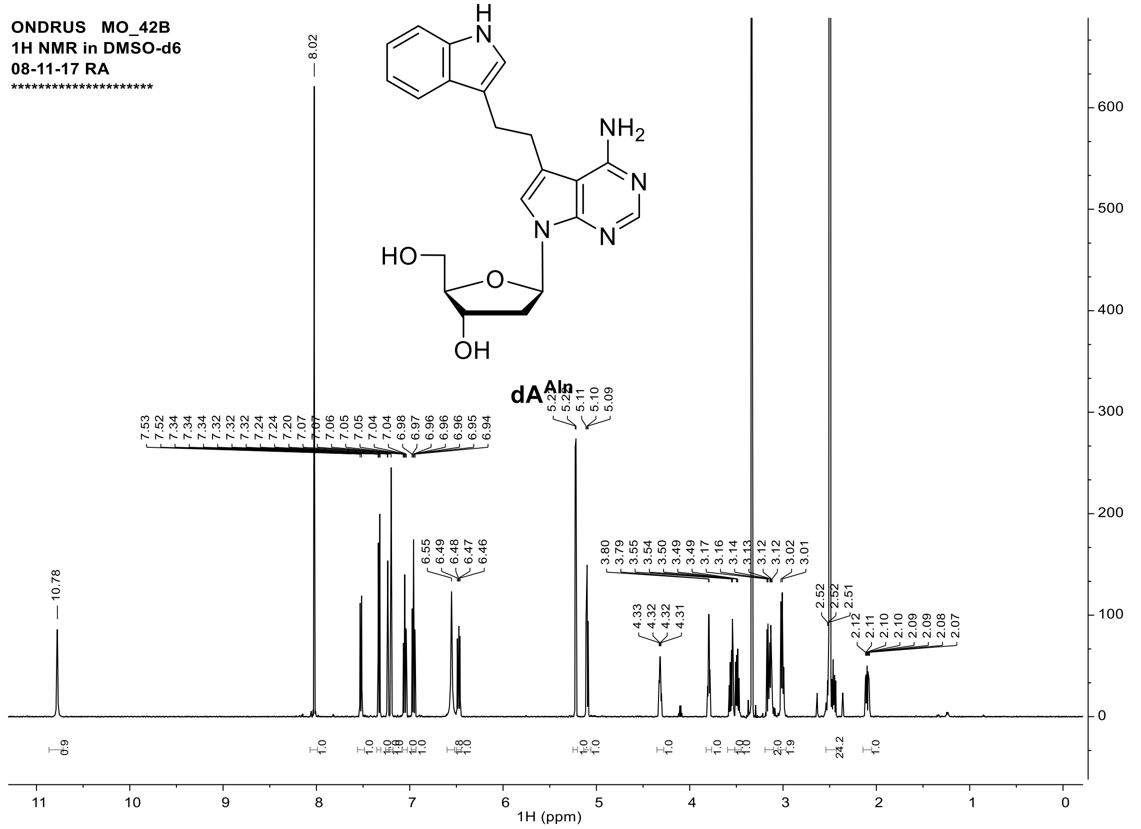

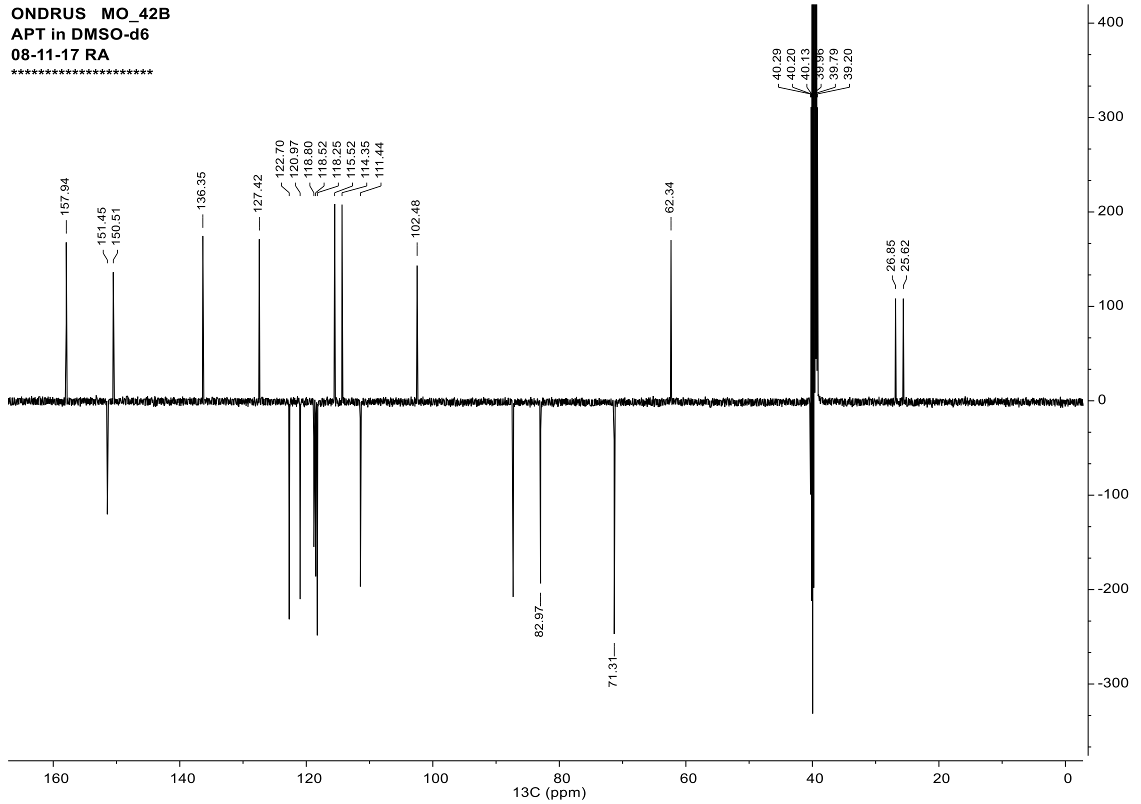

^{1}H, ^{13}C and ^{31}P NMR spectra of **dA^{Aln}TP**:

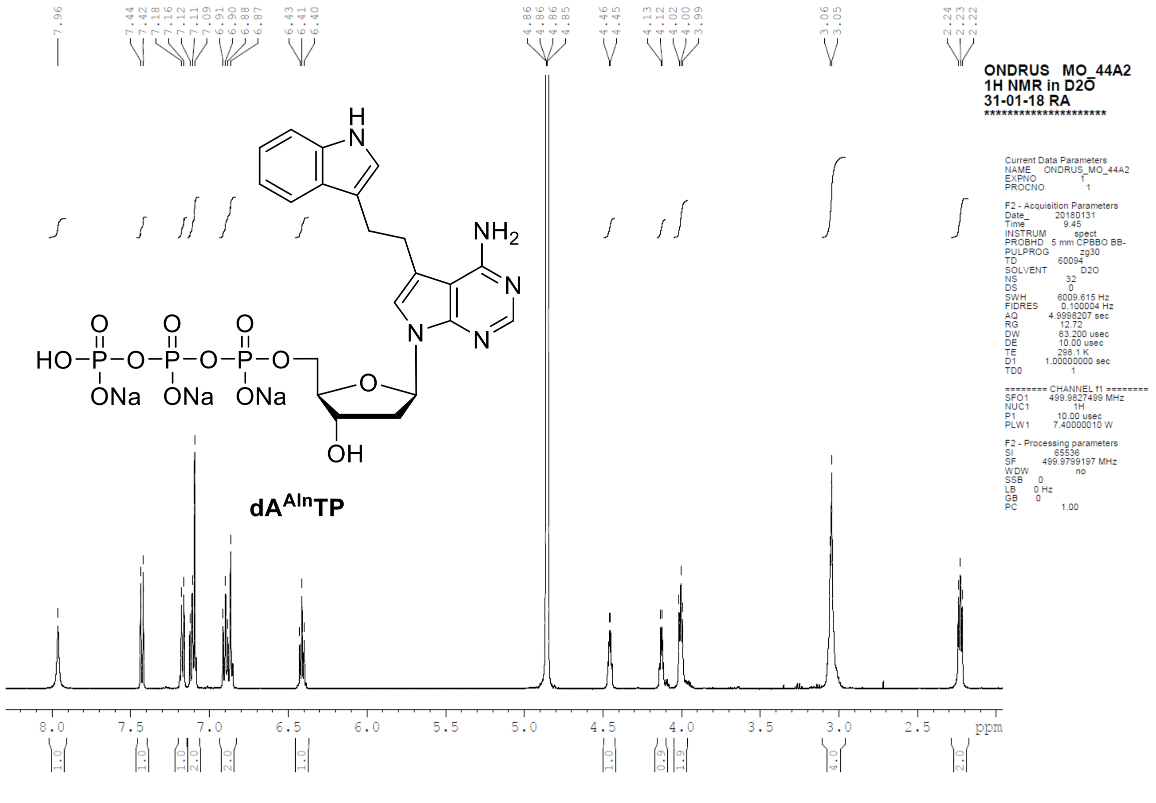

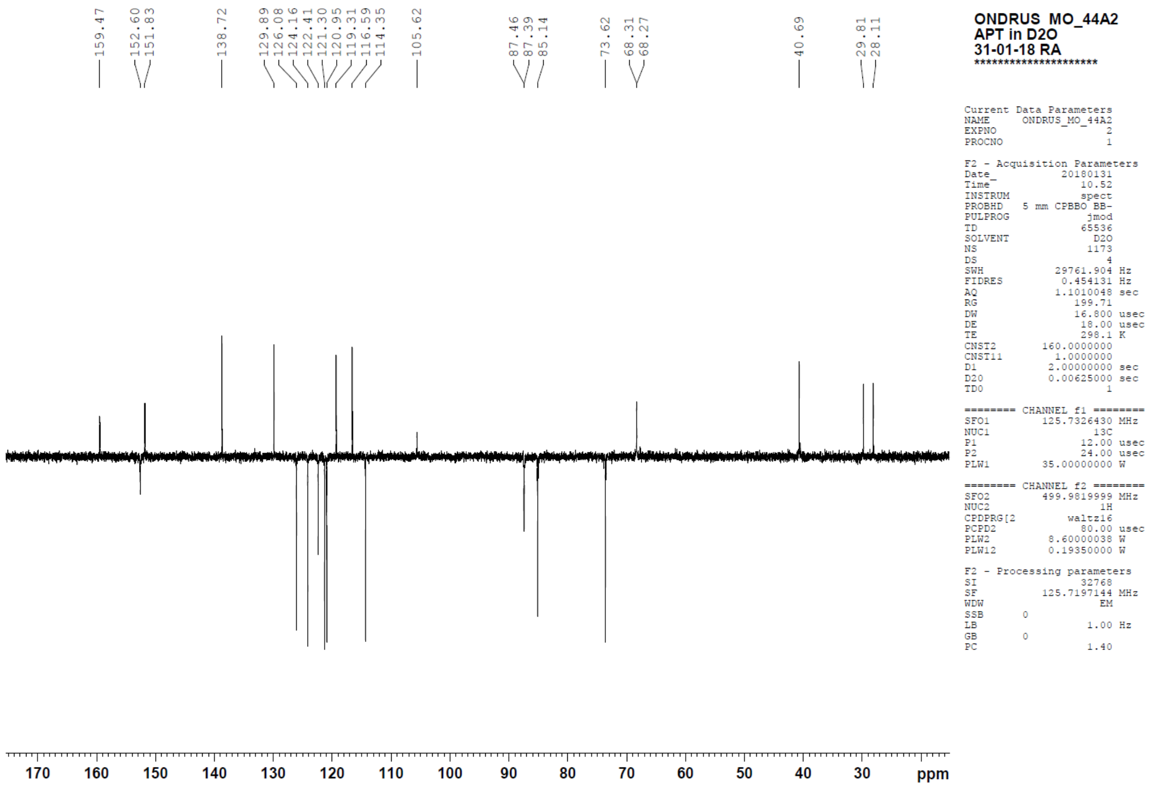

159.47
152.60
151.83
138.72
129.89
126.08
124.16
122.41
121.30
120.95
119.31
116.59
114.35
105.62
87.46
87.39
85.14
73.62
68.31
68.27
40.69
29.81
28.11
170 160 150 140 130 120 110 100 90 80 70 60 50 40 30 ppm
ONDRUS MO_44A2
APT in D2O
31-01-18 RA
Current Data Parameters
NAME ONDRUS_MO_44A2
EXPNO 2
PROCNO 1
F2 - Acquisition Parameters
Date_ 20180131
Time 10.52
INSTRUM spect
PROBHD 5 mm CPBBO BB-
PULPROG jmod
TD 65536
SOLVENT D2O
NS 1173
DS 4
SWH 29761.904 Hz
FIDRES 0.454131 Hz
AQ 1.1010048 sec
RG 199.71
DW 16.800 usec
DE 18.00 usec
TE 298.1 K
CNST2 160.0000000
CNST11 1.0000000
D1 2.00000000 sec
D20 0.00625000 sec
TD0 1
======== CHANNEL f1 ========
SFO1 125.7326430 MHz
NUC1 13C
P1 12.00 usec
P2 24.00 usec
PLW1 35.00000000 W
======== CHANNEL f2 ========
SFO2 499.9819999 MHz
NUC2 1H
CPDPRG[2 waltz16
PCPD2 80.00 usec
PLW2 8.60000038 W
PLW12 0.19350000 W
F2 - Processing parameters
SI 32768
SF 125.7197144 MHz
WDW EM
SSB 0
LB 1.00 Hz
GB 0
PC 1.40

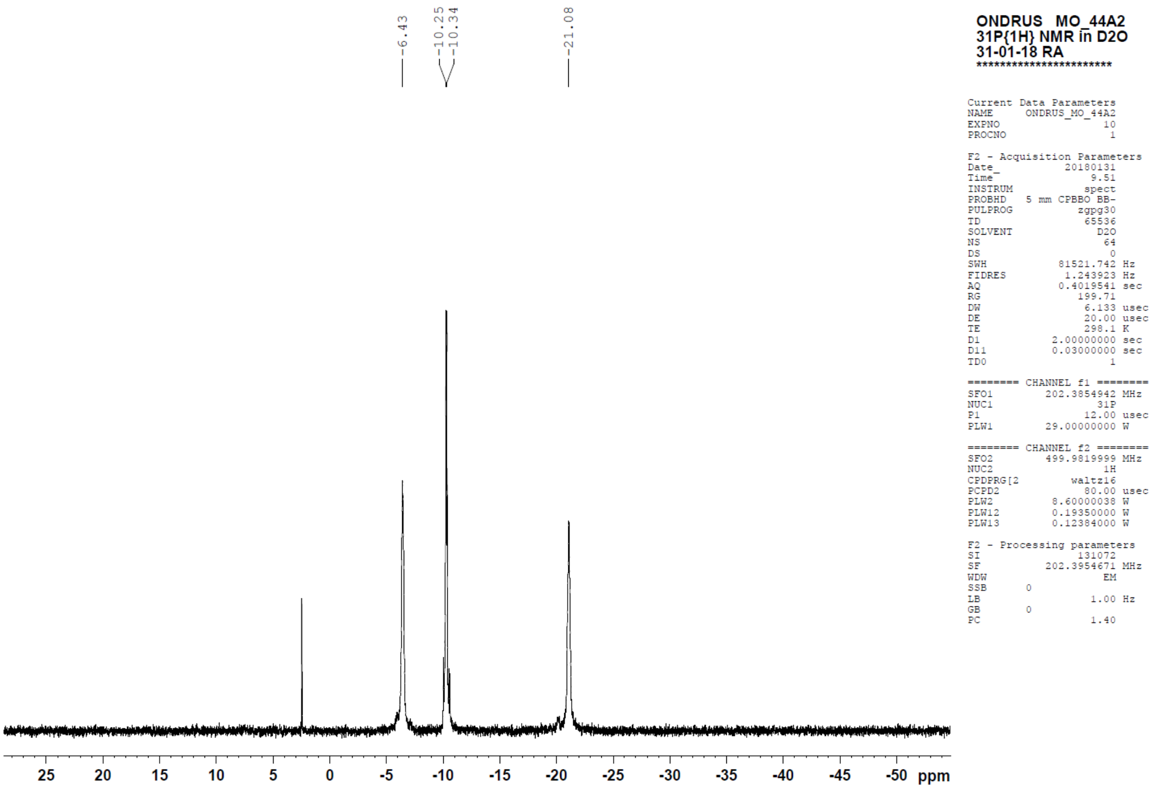

-6.43
-10.25
-10.34
-21.08
25 20 15 10 5 0 -5 -10 -15 -20 -25 -30 -35 -40 -45 -50 ppm
ONDRUS MO_44A2
31P{1H} NMR in D2O
31-01-18 RA
Current Data Parameters
NAME ONDRUS_MO_44A2
EXPNO 10
PROCNO 1
F2 - Acquisition Parameters
Date_ 20180131
Time 9.51
INSTRUM spect
PROBHD 5 mm CPBBO BB-
PULPROG zgpg30
TD 65536
SOLVENT D2O
NS 64
DS 0
SWH 81521.742 Hz
FIDRES 1.243923 Hz
AQ 0.4019541 sec
RG 199.71
DW 6.133 usec
DE 20.00 usec
TE 298.1 K
D1 2.00000000 sec
D11 0.03000000 sec
TD0 1
======== CHANNEL f1 ========
SFO1 202.3854942 MHz
NUC1 31P
P1 12.00 usec
PLW1 29.00000000 W
======== CHANNEL f2 ========
SFO2 499.9819999 MHz
NUC2 1H
CPDPRG[2 waltz16
PCPD2 80.00 usec
PLW2 8.60000038 W
PLW12 0.19350000 W
PLW13 0.12384000 W
F2 - Processing parameters
SI 131072
SF 202.3954671 MHz
WDW EM
SSB 0
LB 1.00 Hz
GB 0
PC 1.40

^{1}H and ^{13}C NMR spectra of compound **dC$^{\mathbf{EAlk}}$**:

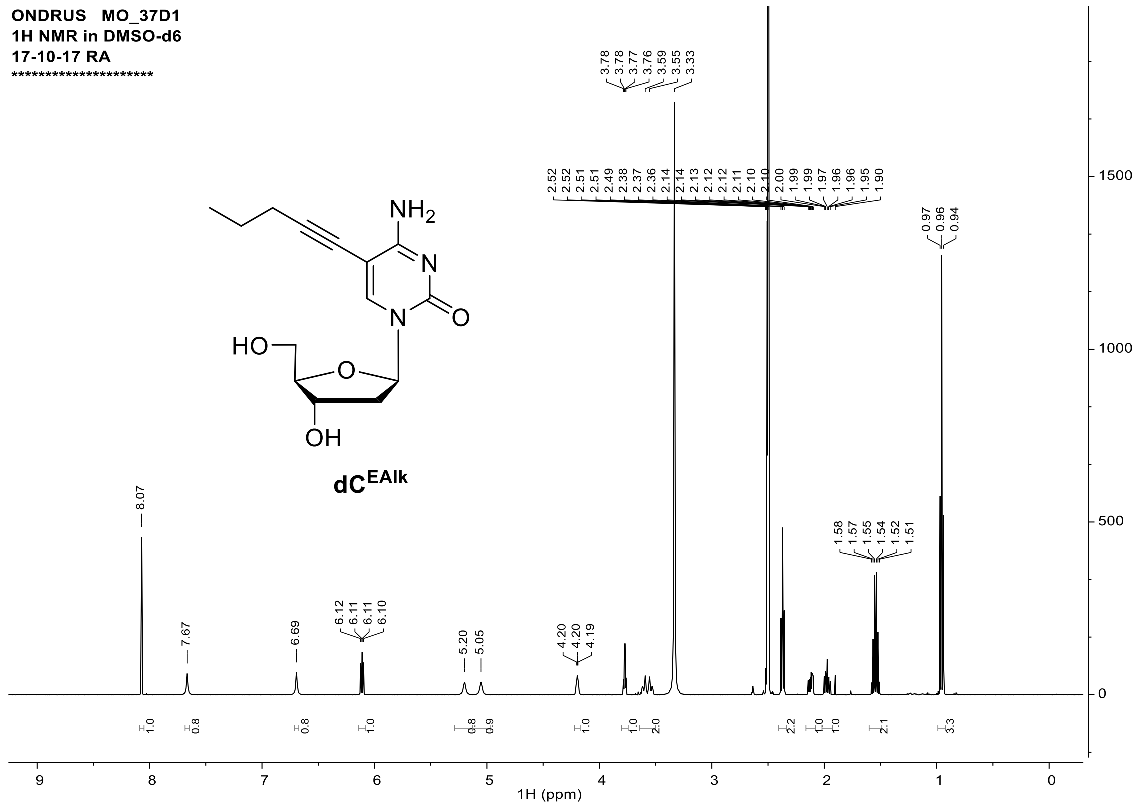

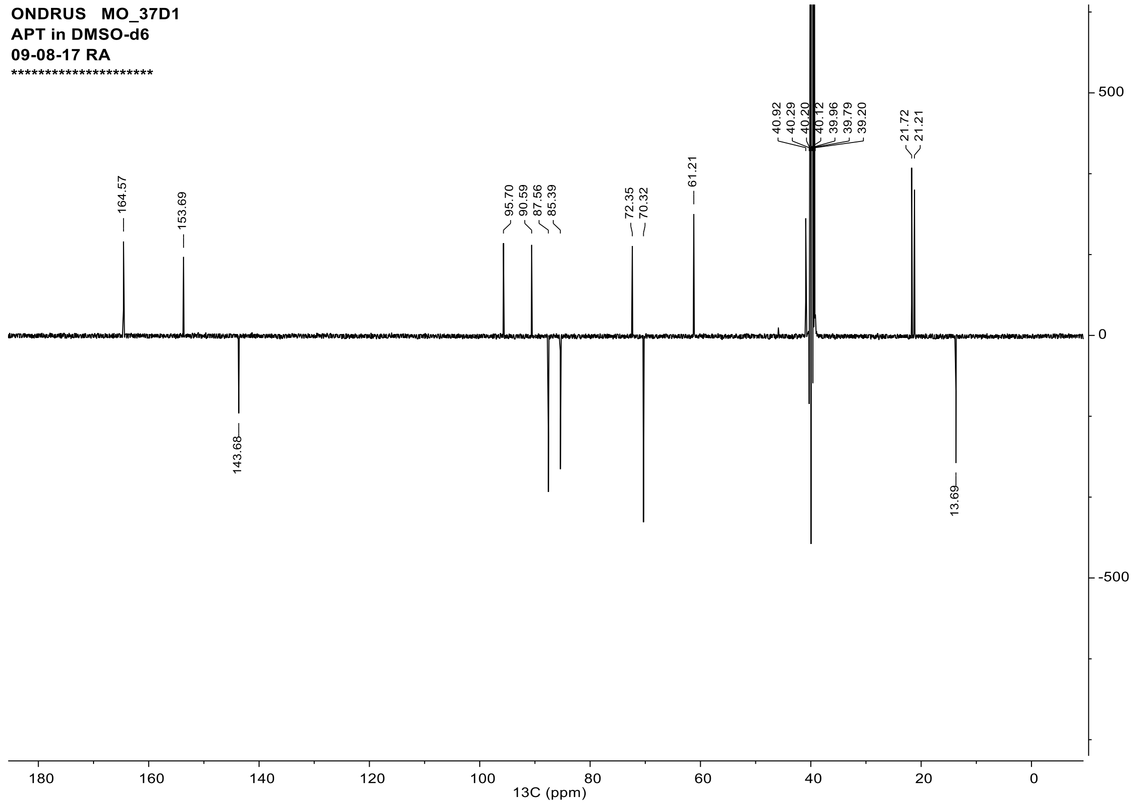

^{1}H, ^{13}C and ^{31}P NMR spectra of **dC^{EAlk}TP**:

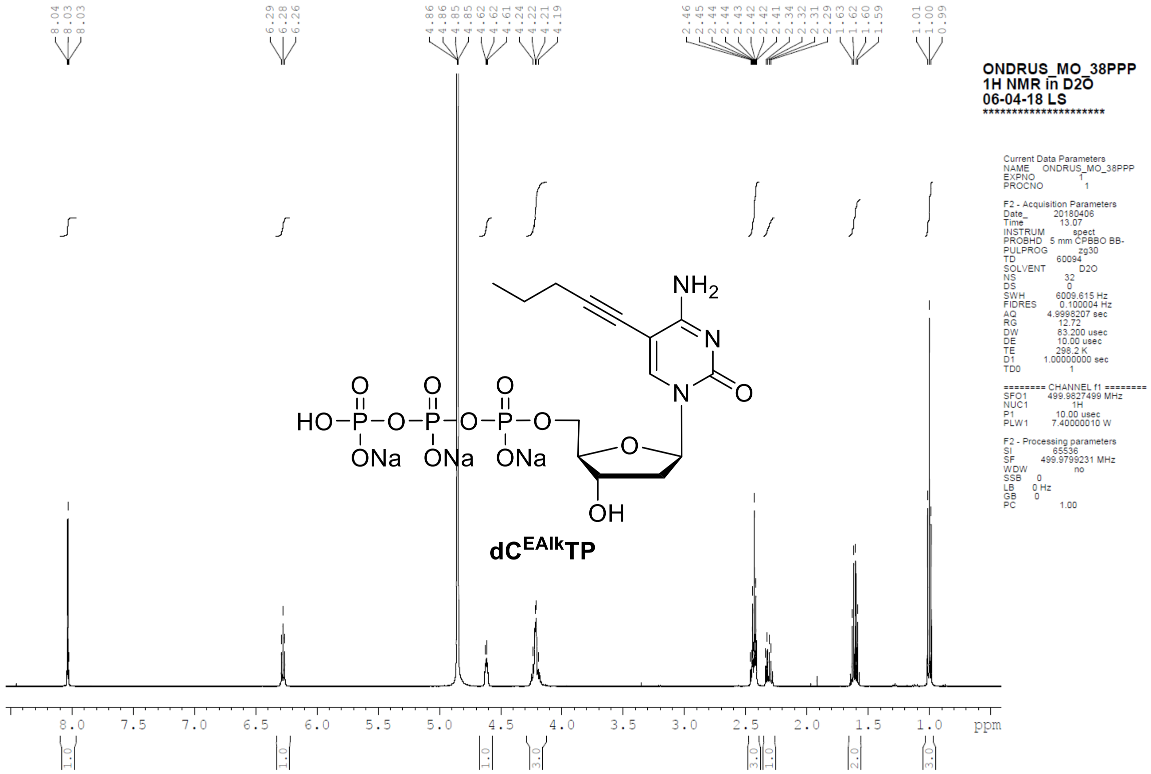

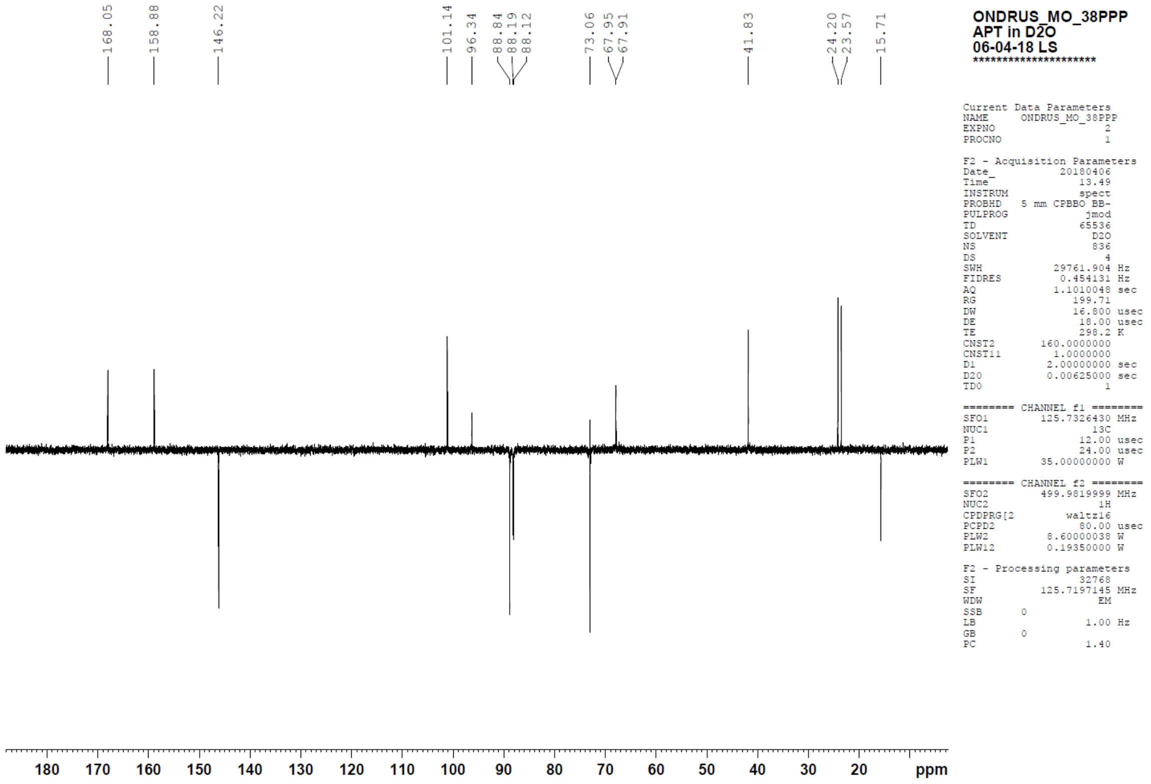

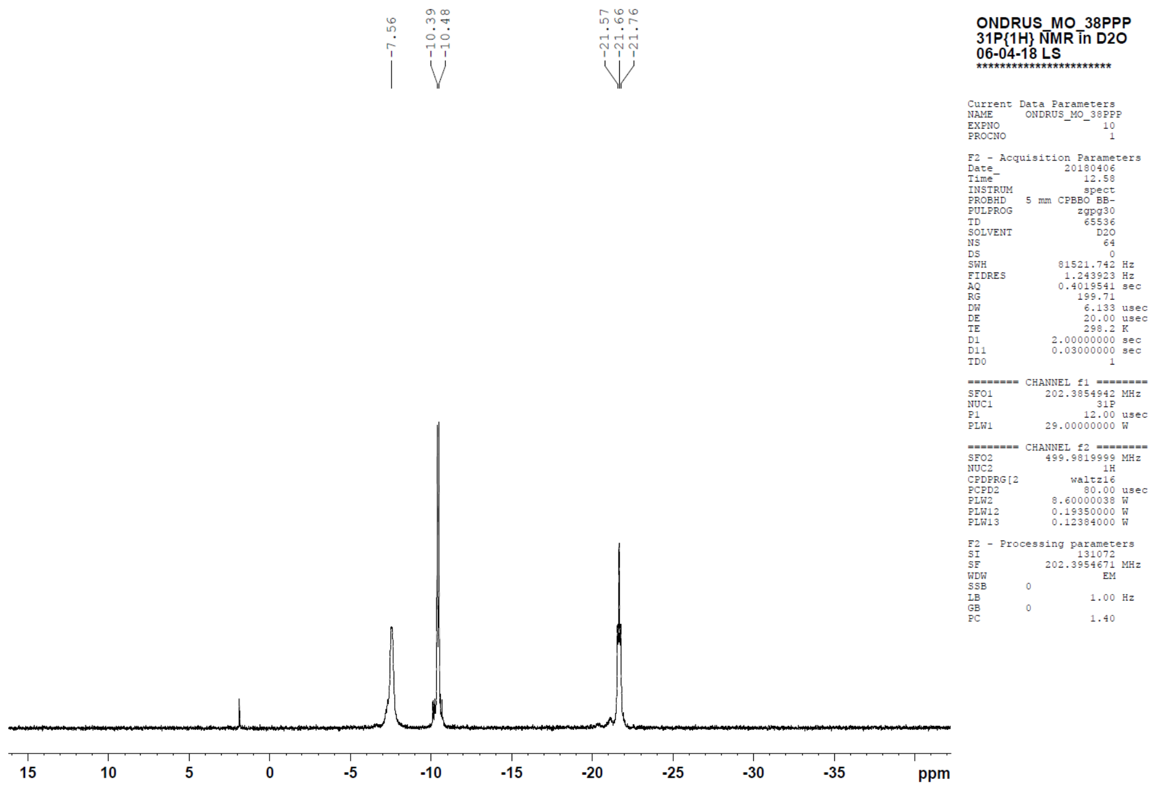

^{1}H and ^{13}C NMR spectra of compound **dC^{AAlk}**:

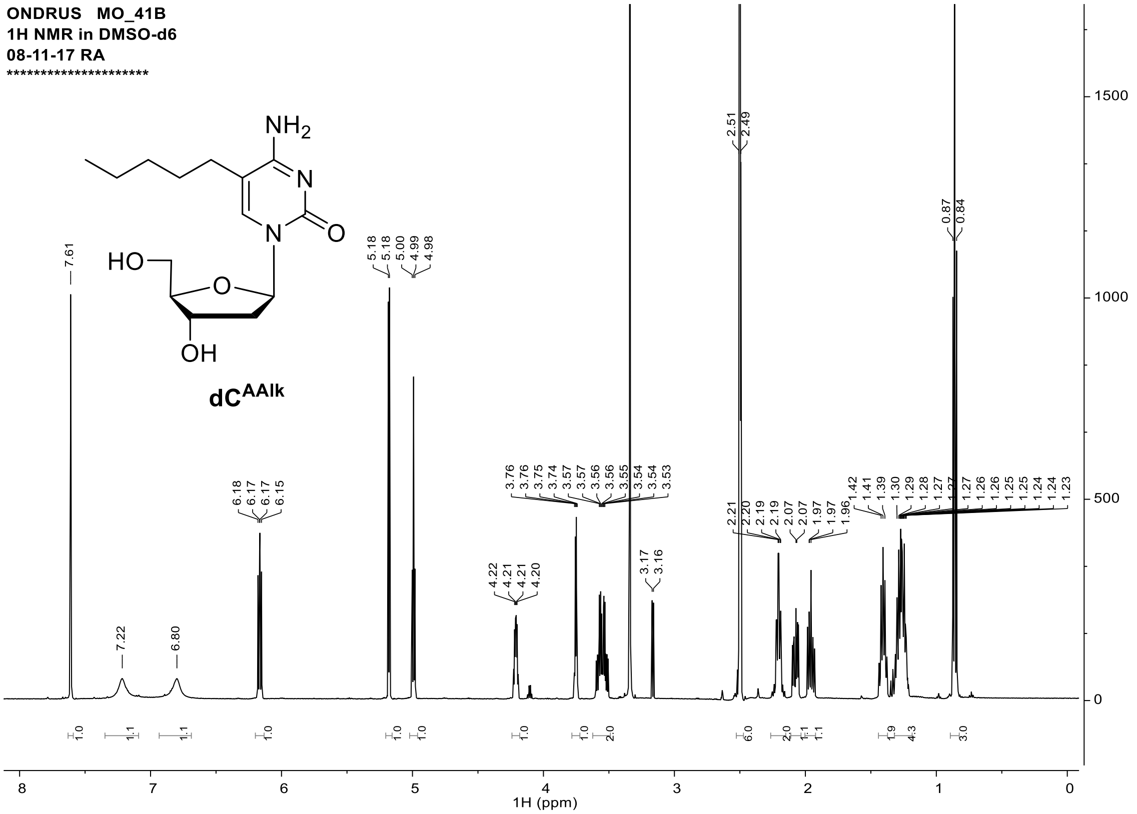

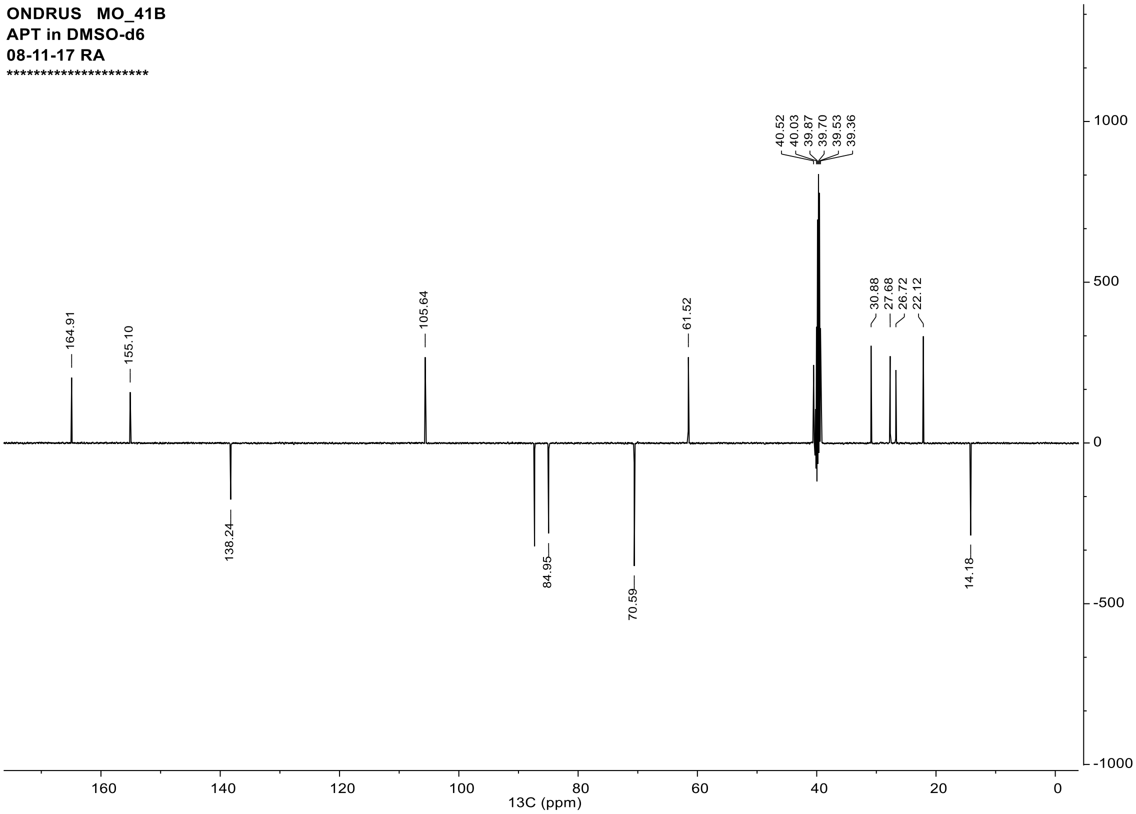

^1H, ^{13}C and ^{31}P NMR spectra of **dC^{AAlk}TP**:

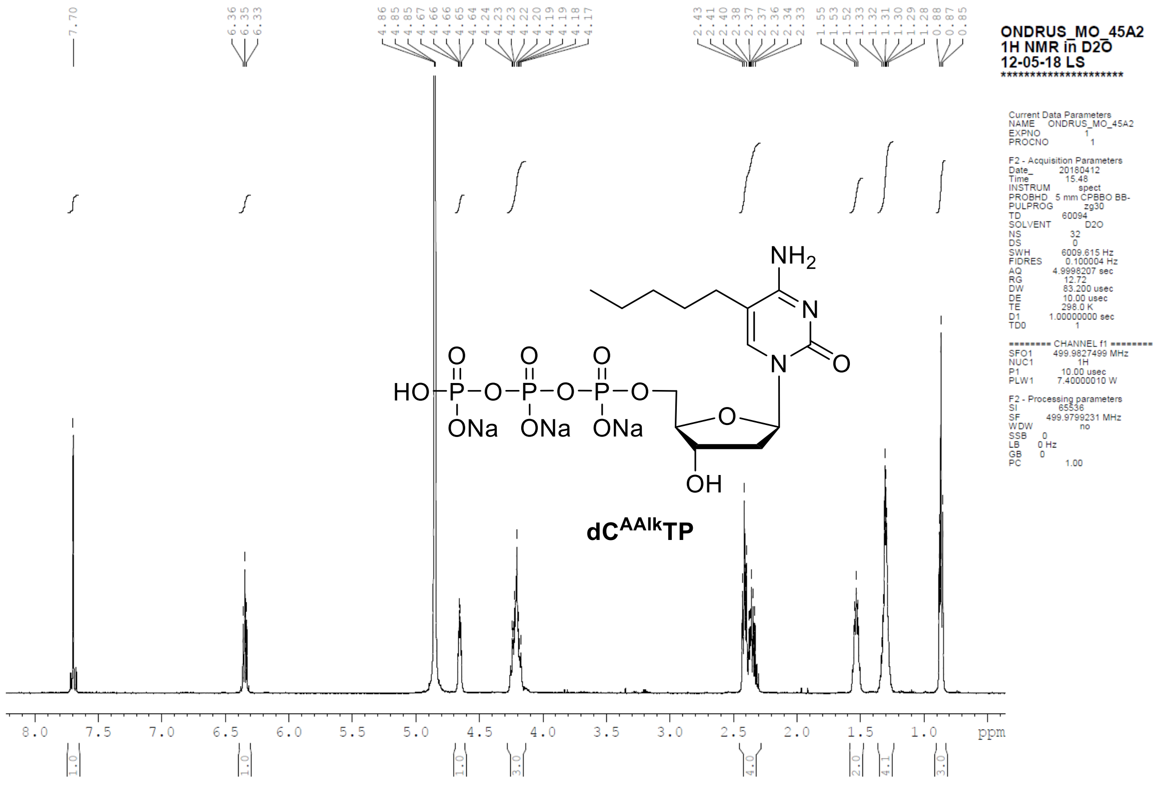

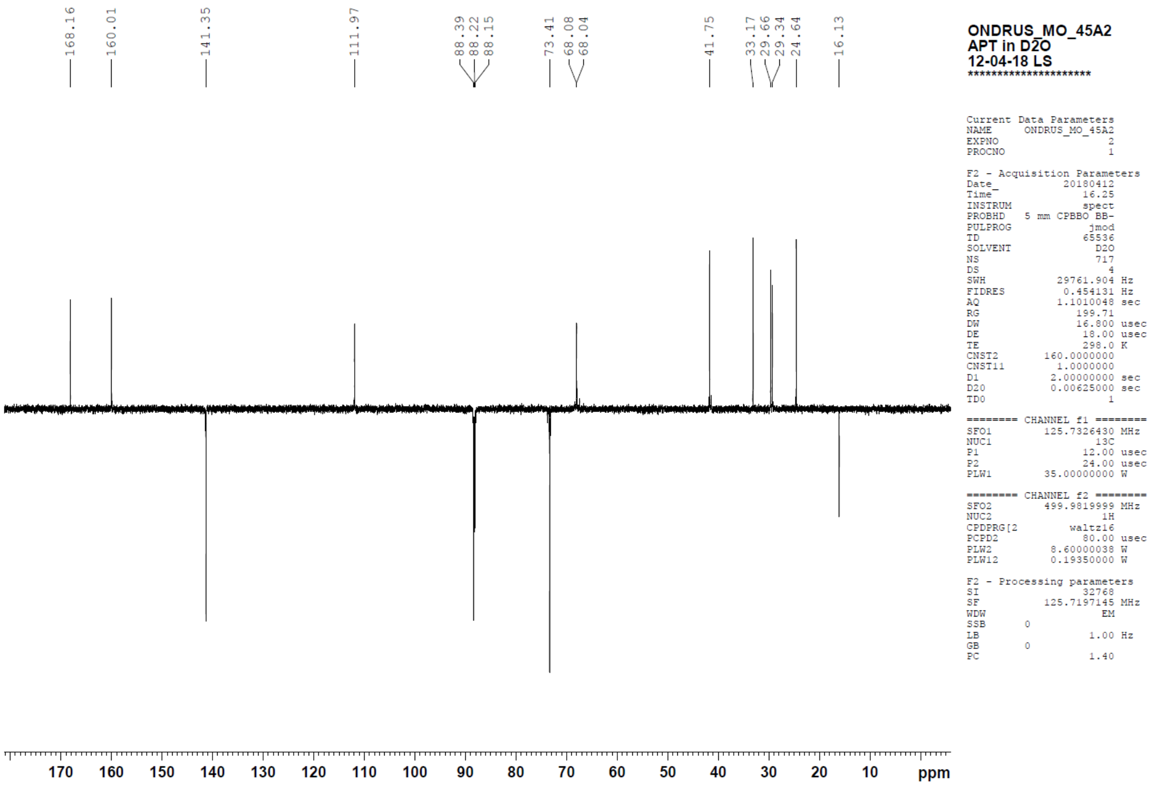
ONDRUS_MO_45A2
APT in D2O
12-04-18 LS
168.16
160.01
141.35
111.97
88.39
88.22
88.15
73.41
68.08
68.04
41.75
33.17
29.66
29.34
24.64
16.13
Current Data Parameters
NAME ONDRUS_MO_45A2
EXPNO 2
PROCNO 1
F2 - Acquisition Parameters
Date_ 20180412
Time 16.25
INSTRUM spect
PROBHD 5 mm CPBBO BB-
PULPROG jmod
TD 65536
SOLVENT D2O
NS 717
DS 4
SWH 29761.904 Hz
FIDRES 0.454131 Hz
AQ 1.1010048 sec
RG 199.71
DW 16.800 usec
DE 18.00 usec
TE 298.0 K
CNST2 160.0000000
CNST11 1.0000000
D1 2.00000000 sec
D20 0.00625000 sec
TD0 1
======== CHANNEL f1 ========
SFO1 125.7326430 MHz
NUC1 13C
P1 12.00 usec
P2 24.00 usec
PLW1 35.00000000 W
======== CHANNEL f2 ========
SFO2 499.9819999 MHz
NUC2 1H
CPDPRG[2 waltz16
PCPD2 80.00 usec
PLW2 8.60000038 W
PLW12 0.19350000 W
F2 - Processing parameters
SI 32768
SF 125.7197145 MHz
WDW EM
SSB 0
LB 1.00 Hz
GB 0
PC 1.40
170 160 150 140 130 120 110 100 90 80 70 60 50 40 30 20 10 ppm

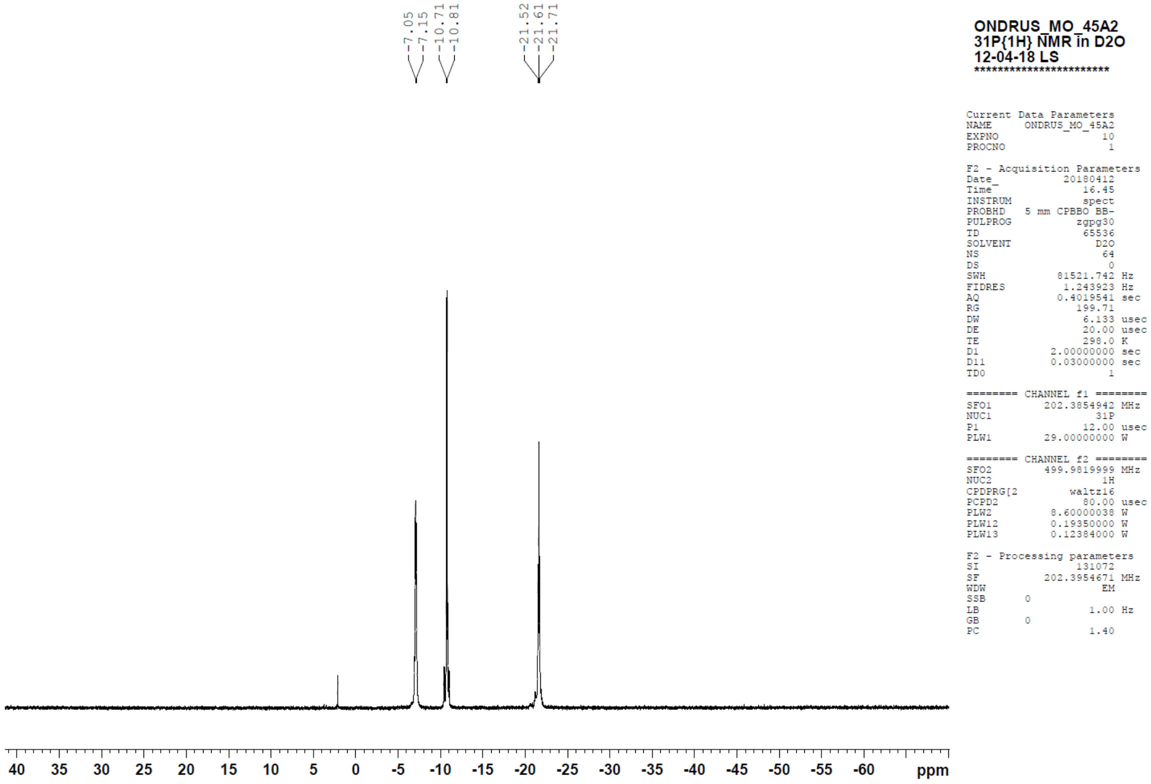
ONDRUS_MO_45A2
31P{1H} NMR in D2O
12-04-18 LS
-7.05
-7.15
-10.71
-10.81
-21.52
-21.61
-21.71
Current Data Parameters
NAME ONDRUS_MO_45A2
EXPNO 10
PROCNO 1
F2 - Acquisition Parameters
Date_ 20180412
Time 16.45
INSTRUM spect
PROBHD 5 mm CPBBO BB-
PULPROG zgpg30
TD 65536
SOLVENT D2O
NS 64
DS 0
SWH 81521.742 Hz
FIDRES 1.243923 Hz
AQ 0.4019541 sec
RG 199.71
DW 6.133 usec
DE 20.00 usec
TE 298.0 K
D1 2.00000000 sec
D11 0.03000000 sec
TD0 1
======== CHANNEL f1 ========
SFO1 202.3854942 MHz
NUC1 31P
P1 12.00 usec
PLW1 29.00000000 W
======== CHANNEL f2 ========
SFO2 499.9819999 MHz
NUC2 1H
CPDPRG[2 waltz16
PCPD2 80.00 usec
PLW2 8.60000038 W
PLW12 0.19350000 W
PLW13 0.12384000 W
F2 - Processing parameters
SI 131072
SF 202.3954671 MHz
WDW EM
SSB 0
LB 1.00 Hz
GB 0
PC 1.40
40 35 30 25 20 15 10 5 0 -5 -10 -15 -20 -25 -30 -35 -40 -45 -50 -55 -60 ppm

^{1}H and ^{13}C NMR spectra of compound **dG^{EiPr}**:

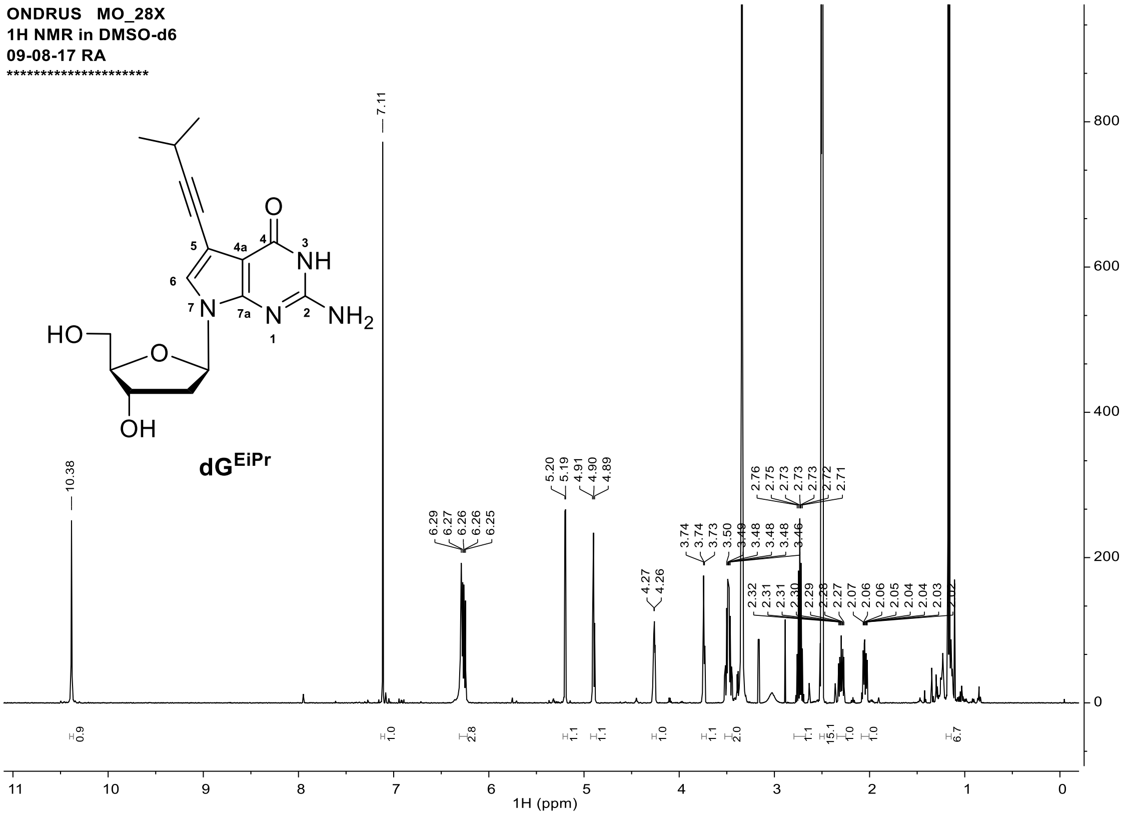

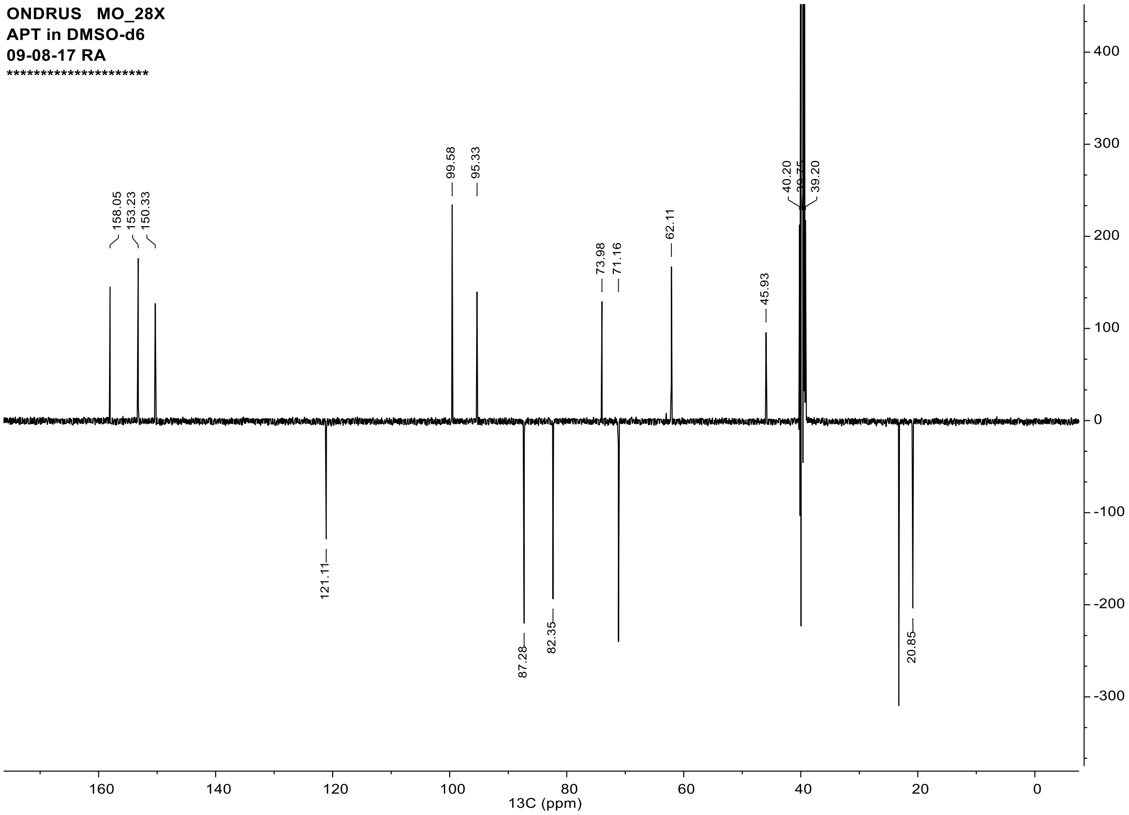

^{1}H, ^{13}C and ^{31}P NMR spectra of **dG^EiPr^TP**:

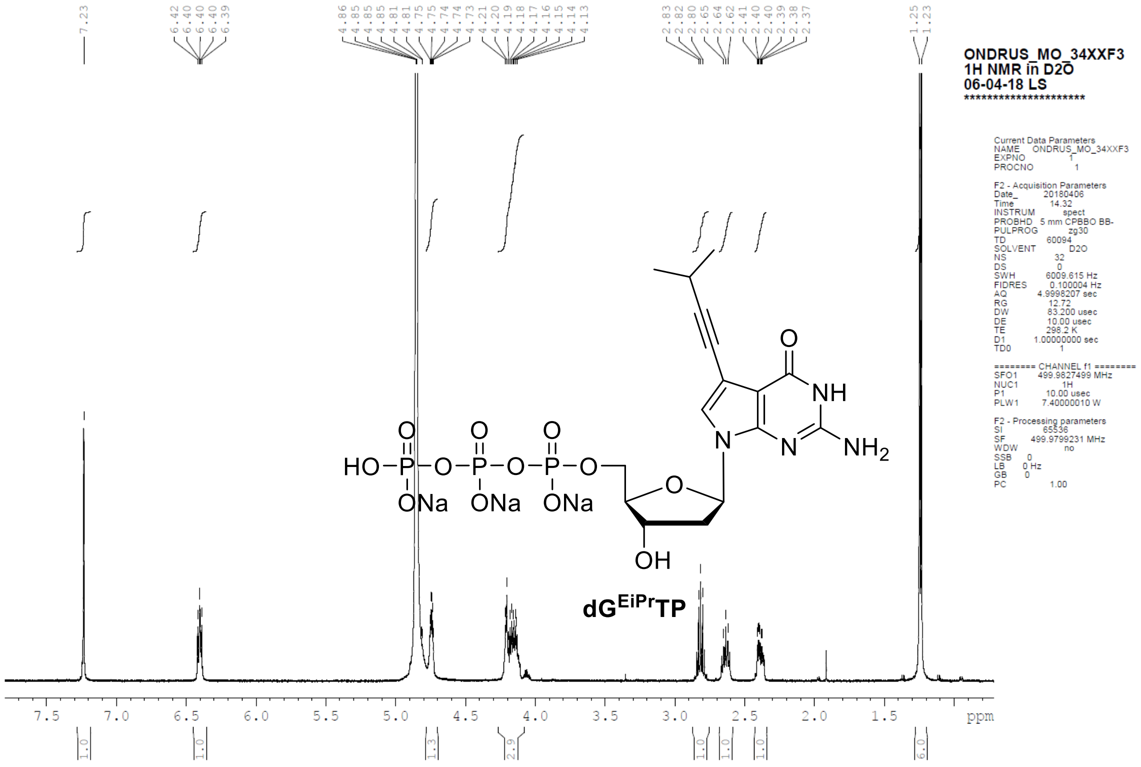

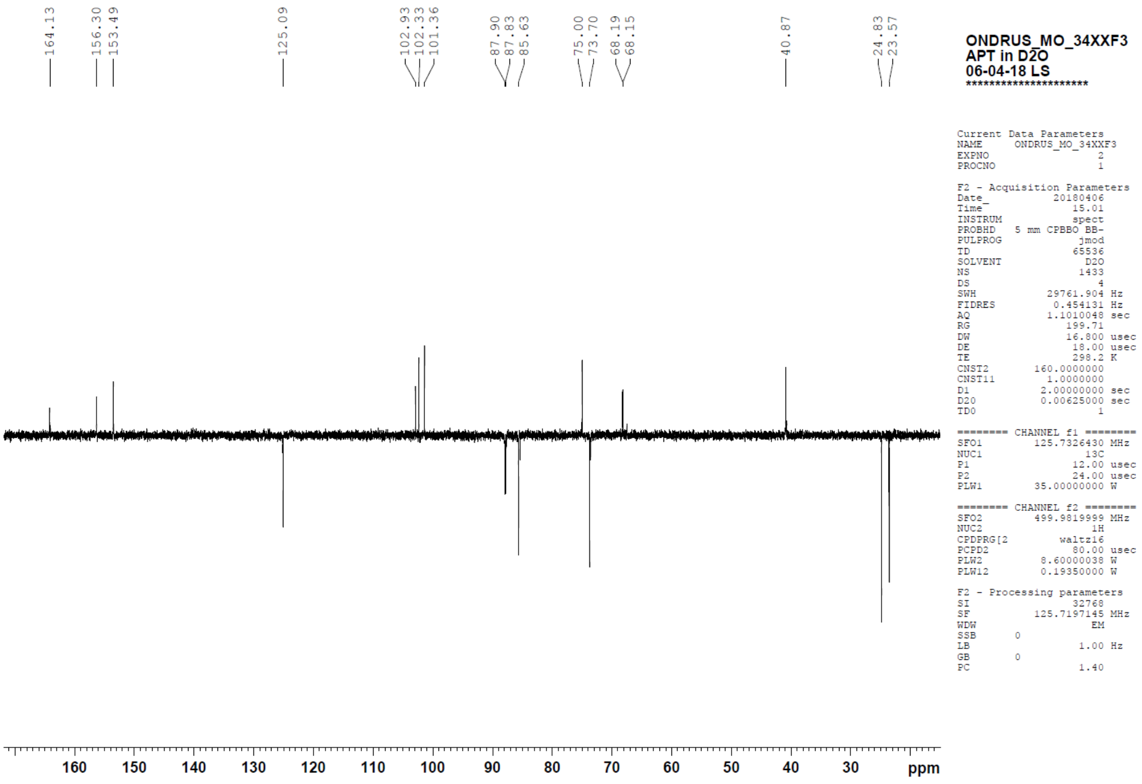

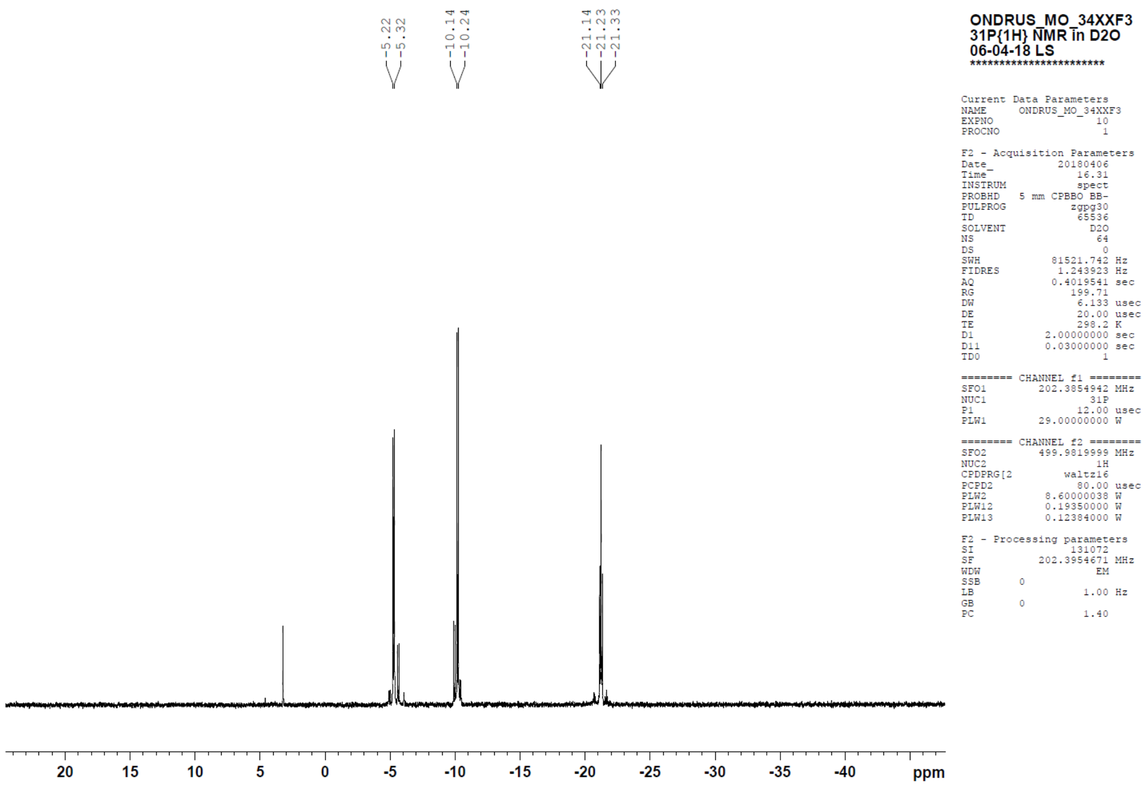

^{1}H, ^{13}C and ^{31}P NMR spectra of compound **dG^{AiPr}**:

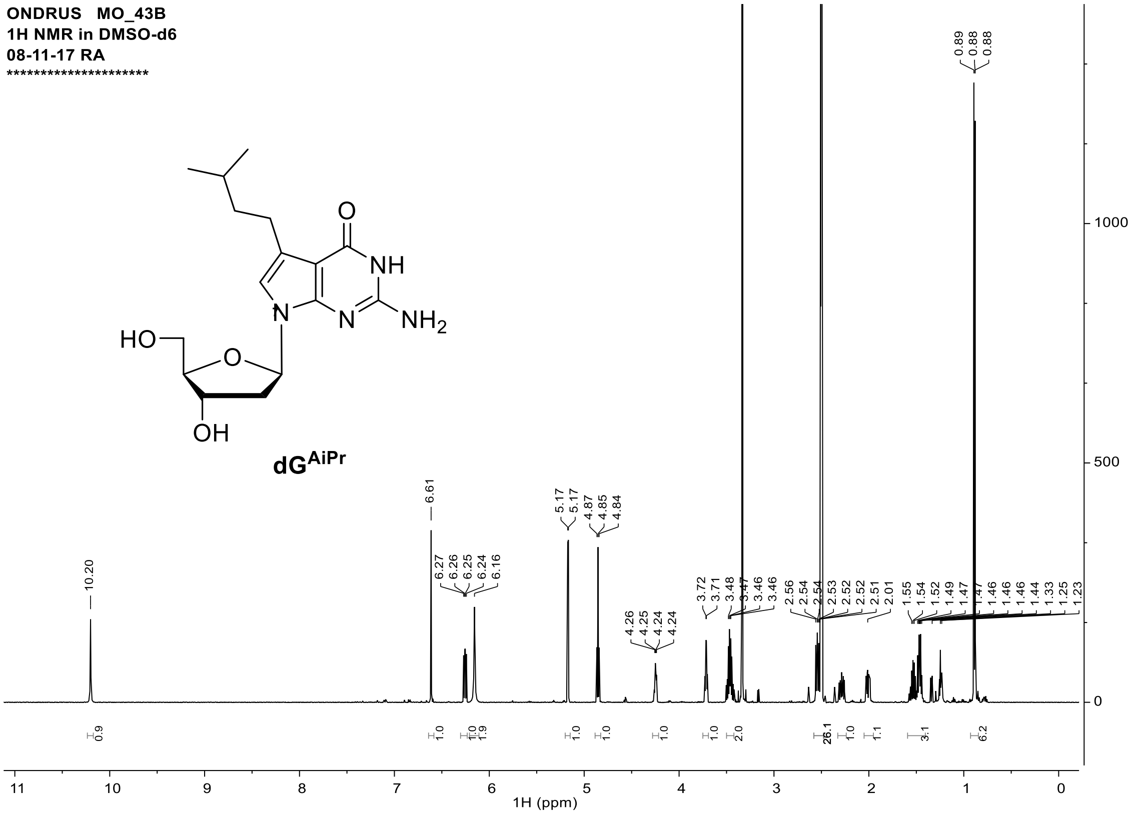

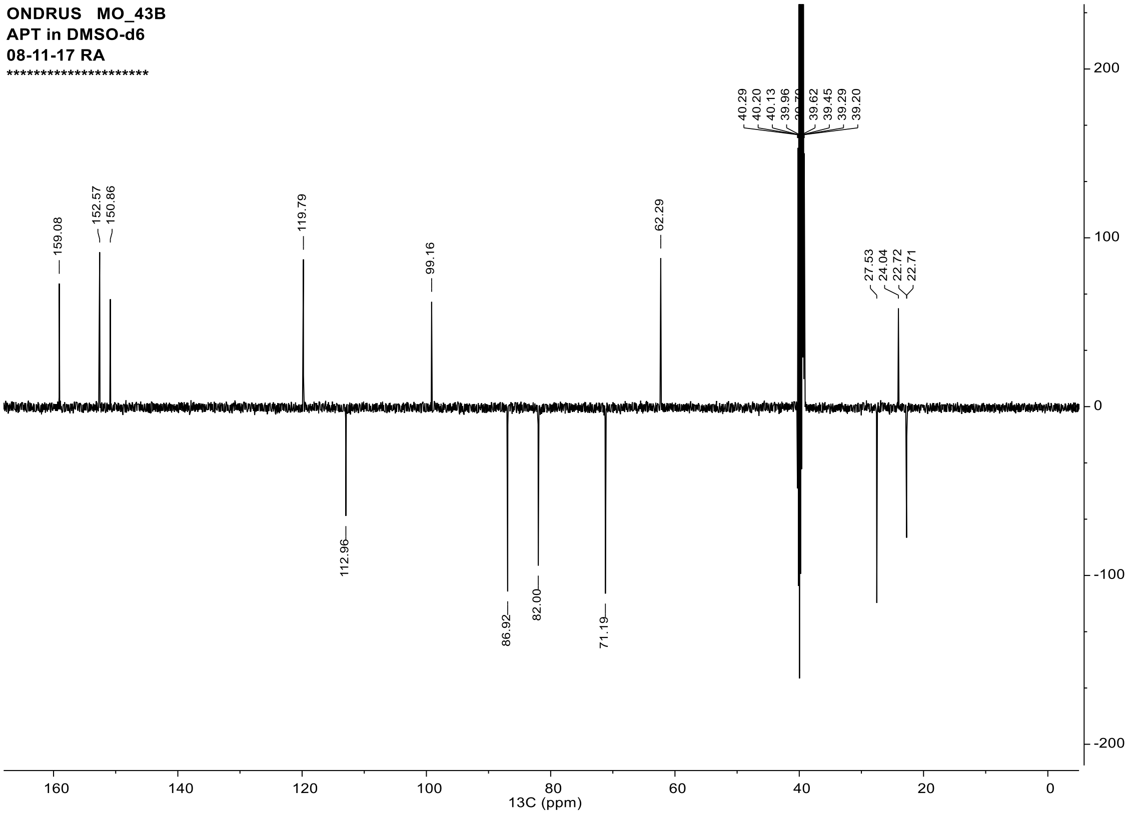

^1H, ^{13}C and ^{31}P NMR spectra of **dG^{AiPr}TP**:

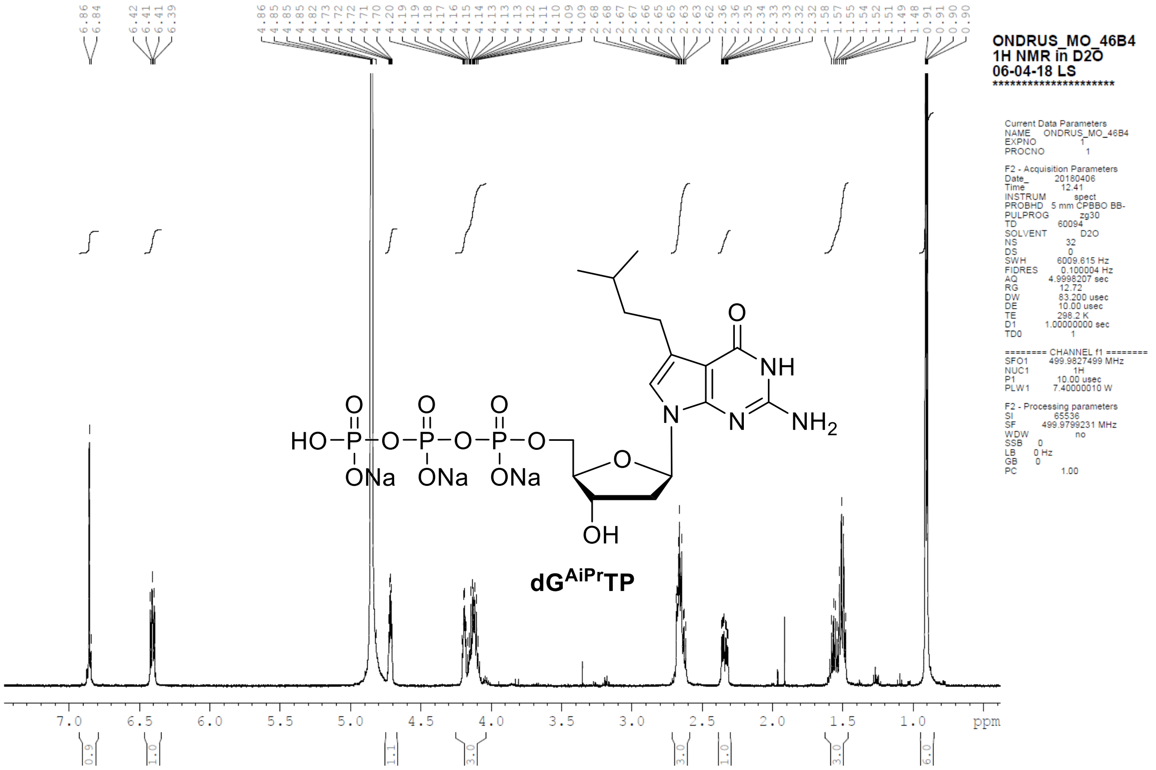

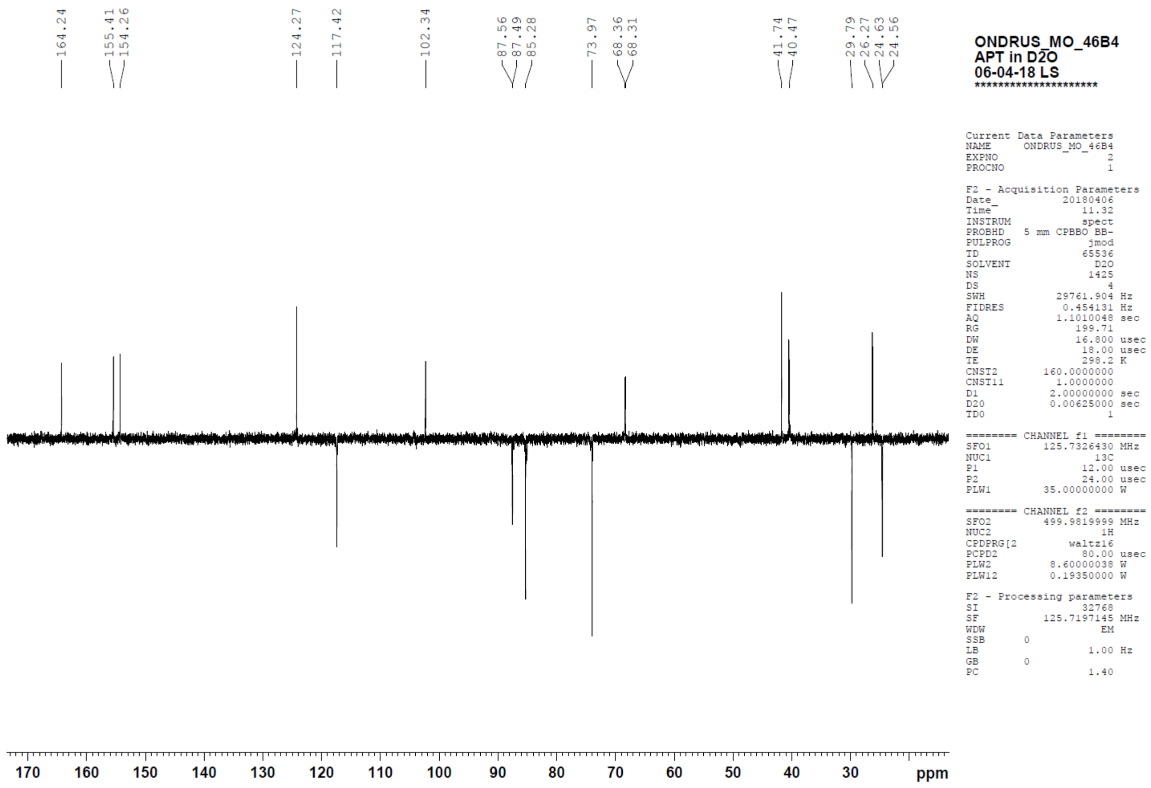

164.24
155.41
154.26
124.27
117.42
102.34
87.56
87.49
85.28
73.97
68.36
68.31
41.74
40.47
29.79
26.27
24.63
24.56
ONDRUS_MO_46B4
APT in D2O
06-04-18 LS
Current Data Parameters
NAME ONDRUS_MO_46B4
EXPNO 2
PROCNO 1
F2 - Acquisition Parameters
Date_ 20180406
Time 11.32
INSTRUM spect
PROBHD 5 mm CPBBO BB-
PULPROG jmod
TD 65536
SOLVENT D2O
NS 1425
DS 4
SWH 29761.904 Hz
FIDRES 0.454131 Hz
AQ 1.1010048 sec
RG 199.71
DW 16.800 usec
DE 18.00 usec
TE 298.2 K
CNST2 160.0000000
CNST11 1.0000000
D1 2.00000000 sec
D20 0.00625000 sec
TD0 1
======== CHANNEL f1 ========
SFO1 125.7326430 MHz
NUC1 13C
P1 12.00 usec
P2 24.00 usec
PLW1 35.00000000 W
======== CHANNEL f2 ========
SFO2 499.9819999 MHz
NUC2 1H
CPDPRG[2 waltz16
PCPD2 80.00 usec
PLW2 8.60000038 W
PLW12 0.19350000 W
F2 - Processing parameters
SI 32768
SF 125.7197145 MHz
WDW EM
SSB 0
LB 1.00 Hz
GB 0
PC 1.40
170 160 150 140 130 120 110 100 90 80 70 60 50 40 30 ppm

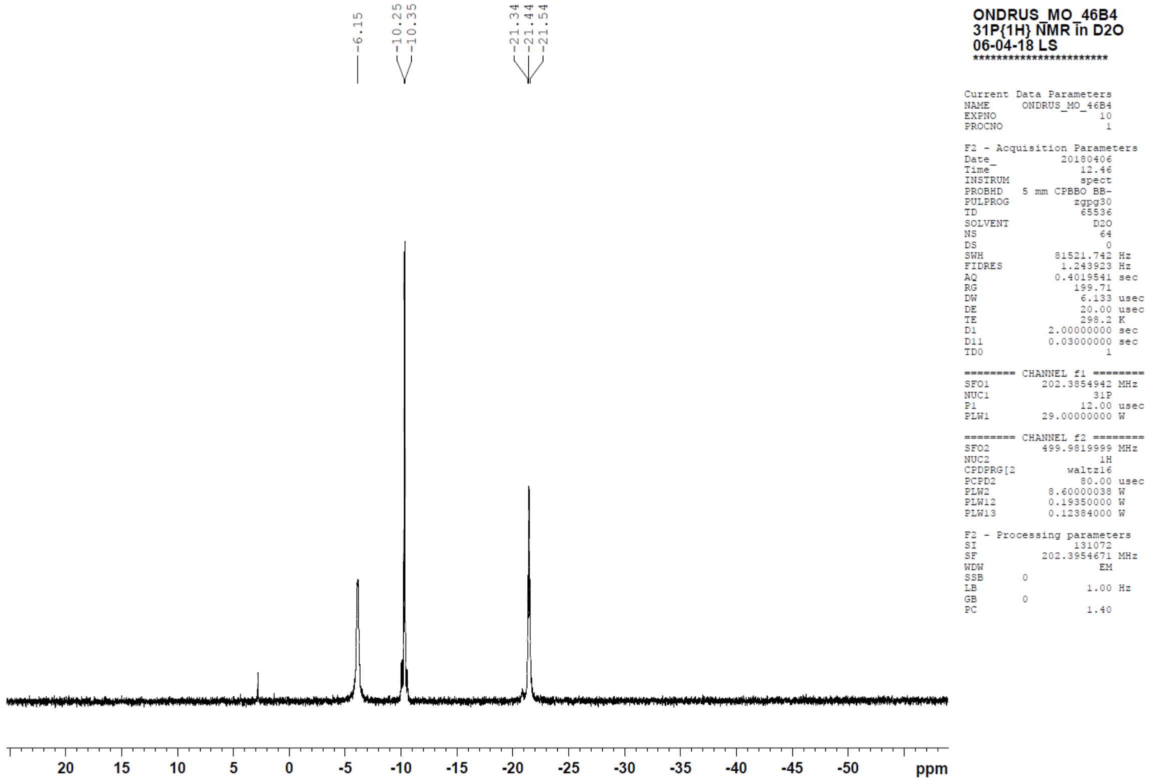

-6.15
-10.25
-10.35
-21.34
-21.44
-21.54
ONDRUS_MO_46B4
31P{1H} NMR in D2O
06-04-18 LS
Current Data Parameters
NAME ONDRUS_MO_46B4
EXPNO 10
PROCNO 1
F2 - Acquisition Parameters
Date_ 20180406
Time 12.46
INSTRUM spect
PROBHD 5 mm CPBBO BB-
PULPROG zgpg30
TD 65536
SOLVENT D2O
NS 64
DS 0
SWH 81521.742 Hz
FIDRES 1.243923 Hz
AQ 0.4019541 sec
RG 199.71
DW 6.133 usec
DE 20.00 usec
TE 298.2 K
D1 2.00000000 sec
D11 0.03000000 sec
TD0 1
======== CHANNEL f1 ========
SFO1 202.3854942 MHz
NUC1 31P
P1 12.00 usec
PLW1 29.00000000 W
======== CHANNEL f2 ========
SFO2 499.9819999 MHz
NUC2 1H
CPDPRG[2 waltz16
PCPD2 80.00 usec
PLW2 8.60000038 W
PLW12 0.19350000 W
PLW13 0.12384000 W
F2 - Processing parameters
SI 131072
SF 202.3954671 MHz
WDW EM
SSB 0
LB 1.00 Hz
GB 0
PC 1.40
20 15 10 5 0 -5 -10 -15 -20 -25 -30 -35 -40 -45 -50 ppm

6) References

1. Cho, J. H.; Prickett, C. D.; Shaughnessy, K. H., Efficient Sonogashira Coupling of Unprotected Halonucleosides in Aqueous Solvents Using Water-Soluble Palladium Catalysts. *Eur J Org Chem* **2010,** (19), 3678-3683.
2. Anderson, A. S.; Hwang, J. T.; Greenberg, M. M., Independent generation and reactivity of 2 '-deoxy-5-methyleneuridin-5-yl, a significant reactive intermediate produced from thymidine as a result of oxidative stress. *J Org Chem* **2000,** *65* (15), 4648-4654.
3. Rai, D.; Johar, M.; Manning, T.; Agrawal, B.; Kunimoto, D. Y.; Kumar, R., Design and studies of novel 5-substituted alkynylpyrimidine nucleosides as potent inhibitors of mycobacteria. *J Med Chem* **2005,** *48* (22), 7012-7017.
